# Genetic Structure Analysis of *Spirometra erinaceieuropaei* Isolates from Central and Southern China

**DOI:** 10.1371/journal.pone.0119295

**Published:** 2015-03-20

**Authors:** Xi Zhang, Jing Cui, Li Na Liu, Peng Jiang, Han Wang, Xin Qi, Xing Qi Wu, Zhong Quan Wang

**Affiliations:** Department of Parasitology, Medical College, Zhengzhou University, Zhengzhou, China; University of Innsbruck, AUSTRIA

## Abstract

**Background:**

Sparganosis caused by invasion of the plerocercoid larvae (spargana) of *Spirometra erinaceieuropaei* have increased in recent years in China. However, the population genetic structure regarding this parasite is still unclear. In this study, we used the sequences of two mitochondrial genes cytochrome *b* (*cytb*) and cytochrome *c* oxidase subunit I (*cox1*) to analyze genetic variation and phylogeographic structure of the *S*. *erinaceieuropaei* populations.

**Methodology/Principal Findings:**

A total of 88 *S*. *erinaceieuropaei* isolates were collected from naturally infected frogs in 14 geographical locations of China. The complete *cytb* and *cox1* genes of each sample was amplified and sequenced. Total 61 haplotypes were found in these 88 concatenated sequences. Each sampled population and the total population have high haplotype diversity (Hd), accompanied by very low nucleotide diversity (Pi). Phylogenetic analyses of haplotypes revealed two distinct clades (HeN+HuN+GZ-AS clade and GX+HN+GZ-GY clade) corresponding two sub-networks yielded by the median-joining network. Pairwise *F*_ST_ values supported great genetic differentiation between *S*. *erinaceieuropaei* populations. Both negative Fu’s *F*_S_ value of neutrality tests and unimodal curve of mismatch distribution analyses supported demographic population expansion in the HeN+HuN+GZ-AS clade. The BEAST analysis showed that the divergence time between the two clades took place in the early Pleistocene (1.16 Myr), and by Bayesian skyline plot (BSP) an expansion occurred after about 0.3 Myr ago.

**Conclusions:**

*S*. *erinaceieuropaei* from central and southern China has significant phylogeographic structure, and climatic oscillations during glacial periods in the Quaternary may affect the demography and diversification of this species.

## Introduction

*Spirometra erinaceieuropaei* (Cestoidea: Pseudophyllidea: Diphyllobothriidae) is one of the most important species of tapeworms [1]. Its plerocercoid larvae (spargana) can lodge in the subcutaneous tissues and sometimes invade the abdominal cavity, eye, and central nervous system of humans causing a serious parasitic zoonosis known as sparganosis [2]. Human infection results mainly from ingesting raw flesh of frogs and snakes infected with the plerocercoids, drinking raw water contaminated with cyclops harboring procercoids, or placing frog or snake flesh as poultice on open wound for treatment of skin ulcers or eye inflammations [3,4]. Sparganosis is distributed worldwide, but most cases occur in Eastern and Southeastern Asia [5,6]. China has the largest number of sparganosis cases in the world since 1999, with a total of approximately 1,000 instances of human sparganosis being reported in 27 out of 34 provinces, autonomous regions, or municipal districts [7]. In addition, the local cases have increased in recent years and sparganosis has even been termed as emerging enzootic diseases in several districts of China [8,9].

Sparganosis not only poses a serious threat to human health, but also causes significant economic losses [10,11]. Consequently, knowledge regarding the distribution of the pathogen of this disease, the genetic characteristics of its populations in relation to local environmental conditions is valuable for the prevention and control of sparganosis in humans. Unfortunately, insufficient studies on population genetics of *S*. *erinaceieuropaei* have been carried out to date. Thus, it becomes important to analyze the population genetics and demographic history of this tapeworm, so that we can get valuable clues about the population changes and genetic variation affecting the pathogenicity [12,13].

Due to high and rapid mutational rate, mitochondrial DNA (mtDNA) remains one of the most powerful and reliable tools for detecting population structure and inferring population differences [14,15]. Previous studies showed that within mtDNA, there are regions that diverge rapidly, while other regions that are highly conserved, making the different regions suitable for analysis of different taxonomic levels [16]. The structure and function of cytochrome *b* (*cytb*) and cytochrome *c* oxidase subunit I (*cox1*) genes have been verified in mtDNA sequences of cestodes and maintain a moderate evolutionary speed. Thus, *cytb* and *cox1* have been used to study the population structure and genetic differentiation of several tapeworm species [17–20]. The aim of this study was to investigate the genetic variability, population structure and divergence pattern among *S*. *erinaceieuropaei* populations from central and southern China based on *cytb* and *cox1* genes of mitochondrial DNA.

## Materials and Methods

### Ethics Statement

The performance of this study was strictly according to the recommendations of the Guide for the Care and Use of Laboratory Animals of the Ministry of Health, China, and our protocol was reviewed and approved by the Institutional Animal Care and Use Committee (IACUC) of Zhengzhou University (Permission No. SYXK 2012-0009). All the frog samples were collected from paddy fields after the permission of farm owners, with no specific permits being required by the authority for the collection of frog samples.

### Sample collection

**Fig 1:**
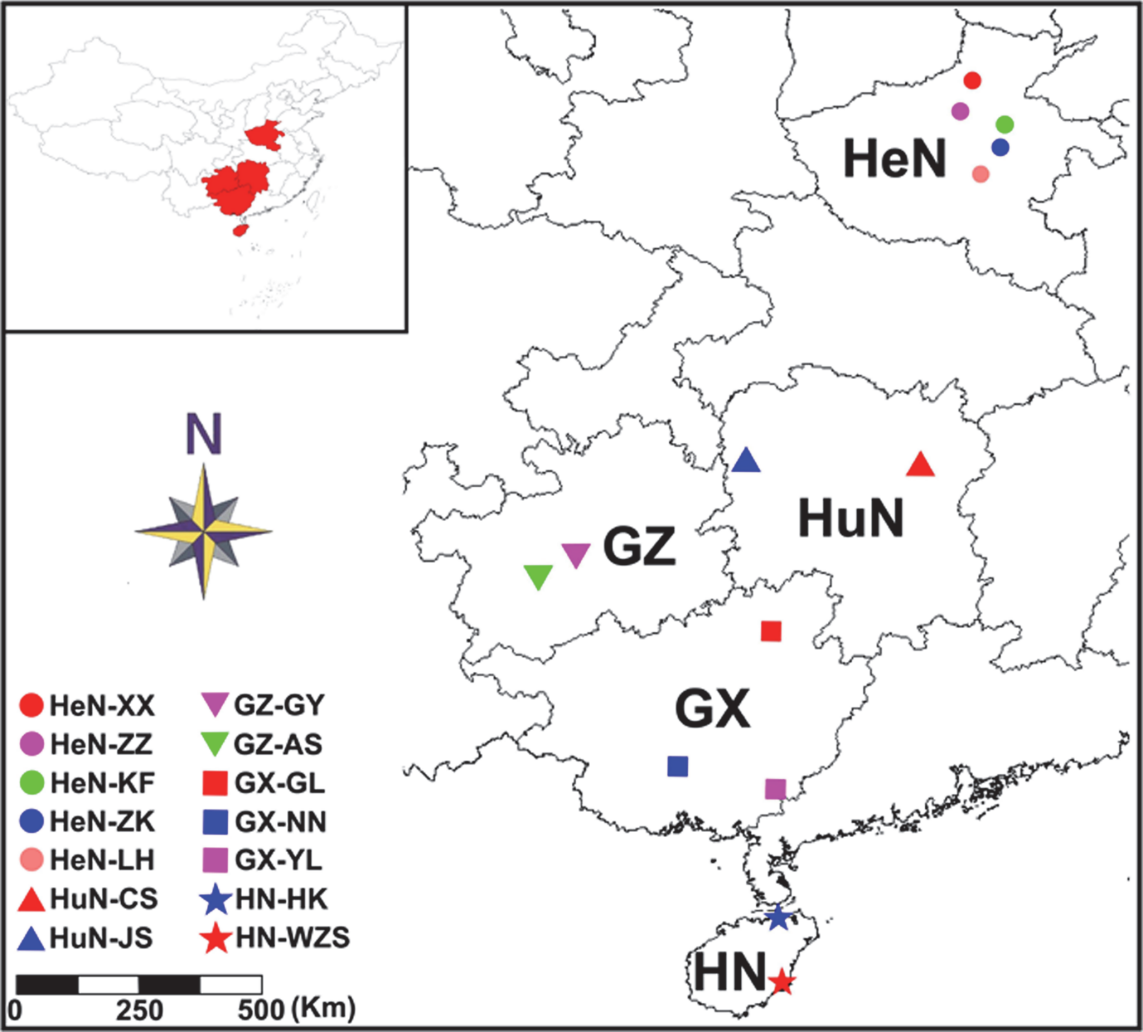
Map of collection localities for *Spirometra erinaceieuropaei* isolates. Geographic regions are designated as follows for China: Henan Province (HeN), Hunan Province (HuN), Guizhou Province (GZ), Guangxi Zhuang Autonomous Region (GX), Hainan Province (HN), Zhengzhou of Henan (HeN-ZZ), Kaifeng of Henan (HeN-KF), Luohe of Henan (HeN-LH), Zhoukou of Henan (HeN-ZK), Xinxiang of Henan (HeN-XX), Changsha of Hunan (HuN-CS), Jishou of Hunan (HuN-JS), Yulin of Guangxi (GX-YL), Guilin of Guangxi (GX-GL), Nanning of Guangxi (GX-NN), Guiyang of Guizhou (GZ-GY), Anshun of Guizhou (GZ-AS), Haikou of Hainan (HN-HK), Wuzhishan of Hainan (HN-WZS).

A total of 88 *S*. *erinaceieuropaei* isolates were collected from naturally infected frogs caught from a field site in fourteen geographical locations of China during May 2012 to August 2013 (Fig. 1 and S1 Table). Briefly, frogs were euthanized using ethyl-ether anesthesia, weighed, and skinned. Skeletal muscles and subcutaneous tissues were carefully and visually observed for the presence of spargana. These were removed from muscles or subcutaneous tissues and placed in a Petri dish containing physiological saline.

### DNA extraction, amplification and sequencing

Total genomic DNA was extracted from individual sample of plerocercoid using the Tiangen DNeasy Blood and Tissue Kit (Tiangen, China) following the manufacturer’s protocol. The whole sequences of *cytb* and *cox1* genes were amplified with Cob-F/R and Cox1-F/R two sets of primers respectively according to Yanagida et al. [21]. Polymerase chain reaction (25μl) was performed in 2mM MgCl2, 2.5μM of each primer, 2.5μl 10× rTaq buffer, 0.5mM of each deoxyribonucleoside triphosphate (dNTP), 1.25U of rTaq DNA polymerase (Takara, China), and 1μl of DNA sample in a thermocycler under the following conditions: after an initial denaturation at 94°C for 1 min, then 94°C for 30 s (denaturation); 58°C for 30 s (annealing); 72°C for 1 min (extension) for 30 cycles, followed by a final extension at 72°C for 5 min. These optimized amplification conditions for the specific and efficient amplification of individual DNA fragments were obtained after varying annealing and extension temperatures. One microlitre (5–10 ng) of genomic DNA was added to each PCR reaction. Samples without genomic DNA (no-DNA controls) were included in each amplification run, and in no case were amplicons detected in the no-DNA controls (not shown). PCR products were purified using the High Pure PCR Product Purification Kit (Takara, China) and sequenced in both directions using an automated sequencer (ABI Prism 3730 XL DNA Analyzer; ABI Prism, Foster City, CA) at the Genwiz Company (Beijing, China).

### Nucleotide polymorphism

Sequences of *cytb* and *cox1* genes were aligned using the computer program Clustal X 2.0 [22]. Molecular Evolutionary Genetics Analysis (MEGA) software version 5.0 [23] was employed to analyze the nucleotide composition, conserved sites, variable sites, parsimony-informative sites and singleton sites. The number of haplotypes, calculation of haplotype diversity (Hd) and nucleotide diversity (Pi) were performed using the program DnaSP v5.10.01 [24].

### Phylogenetic analysis

The phylogenetic relationship among haplotypes was estimated through three methods of maximum parsimony (MP), neighbor-joining (NJ) and Bayesian inference (BI), respectively. MP analysis was performed in PAUP*4b10 [25] using heuristic searches with TBR (tree bisection-reconnection) branch swapping and 2,000 random addition sequences. Confidence in each node was assessed by boot-strapping (2000 pseudo-replicates, heuristic search of 20 random addition replicates with TBR option). NJ analysis was also performed in PAUP*4b10 [25] using the Kimura two-parameter distance selected by Modeltest v3.7 [26] under the Akaike information criterion and the ‘heuristics’ search option with the ‘simple’ addition sequence and TBR swapping. BI analyses were performed in MrBayes v3.1 [27] with 5,000,000 generations, sampling trees every 100 generations. Stationarity was assessed using a convergence diagnostic. An average standard deviation of the split frequencies (ASDSF) < 0.03 were used as criteria of convergence between both runs. Four *Diphyllobothrium* species: *Diphyllobothrium nihonkaiense* (Genbank accession number of cytb/cox1: AB508837/AB015755), *D*. *latum* (AB522608/AB511963), *D*. *dendriticum* (AB522613/KC812045) and *D*. *ditremum* (AB522617/FM209182) were used as outgroup to root the resulting trees. We also used NETWORK v4.5.0.2 [28] to draw a median-joining network to analyze the relationships among detected haplotypes.

### Analyses of genetic structure

The partitions of genetic diversity within and among populations were analyzed through analysis of molecular variance (AMOVA) [29] using the software Arlequin v3.5.1.2 [30]. To estimate levels of genetic differentiation among the populations, a pairwise comparison test was performed based on Slatkin’s linearised *F*_ST_ [31] using Arlequin v3.5.1.2 [30]. The significance of *F*_ST_ evaluated was based on 10 000 random permutations (significance levels *p* = 0.05).

### Divergence time estimation

The approximate divergence times were estimated for the lineages of *S*. *erinaceieuropaei* with an uncorrelated log-normal relaxed molecular-clock model using the software BEAST v1.6.1 [32]. The substitution models were HKY+G for *cytb* and HKY+G+I for *cox1* following model selection by Modeltest v3.7 [26]. For the tree prior, a basic coalescent model assuming a constant population size over the time period considered was chosen according to the reduced effective population size resulting from the selfing mode of reproduction of *S*. *erinaceieuropaei*. Posterior distributions of the parameters, including the tree, were estimated via Markov chain Monte Carlo (MCMC) sampling. Two replicate MCMC runs were performed, with the tree and parameter values sampled every 1 000 steps over a total of 1 × 108 steps. The diagnostic software Tracer v1.5 [33] was used to assess convergence between runs, to estimate an appropriate number of samples to be discarded as burn-in, and to ensure that effective sample sizes (i.e., >500) were sufficient to provide reasonable estimates of model parameter variance. LogCombiner v1.6.1 [32] was used to combine both runs. The sampled tree with the maximum product of clade credibilities was viewed using FigTree v1.3.1 [34]. The molecular evolutionary rate were fixed to 0.0195 and 0.0225 substitutions per site per million year (Myr) for *cytb* and *cox1* respectively, according to the substitution rates for *cytb* and *cox1* calculated based on the *Taenia* tapeworms [19,35].

### Demographic history of population

We applied Neutrality tests through the program Arlequin v3.5.1.2 [30] as an assessment of possible population expansion. Under the assumption of neutrality, a population expansion produces a large negative value of Fu’s *F*_S_ test [36] and Tajima’s *D* [37]. Tajima’s *D* and Fu’s *F*_S_ are sensitive to bottleneck effects or population expansion, causing these values to be more significantly negative [38]. Fu’s *F*_S_ is particularly sensitive to recent population growth [36]. Population expansion events were determined through mismatch analysis [39] using Arlequin v3.5.1.2 [30] with the number of bootstrap replicates set to 5000. The validity of the expansion model was tested by using the sum of squared deviations (SSD) and Harpending’s raggedness index (Rag) between observed and expected mismatches. The Bayesian skyline plot (BSP) was used to estimate the demographic history of *S*. *erinaceieuropaei* using the program BEAST v1.6.1 [32]. A piecewise-constant skyline model was selected, and a relaxed uncorrelated log-normal molecular clock was used with the mutation rates of 1.95%/Myr for *cytb* and 2.25%/Myr for *cox*1 as suggested by Hoberg et al. [35] and Michelet et al. [19]. Tracer1.5 was used to reconstruct the demographic history through time.

## Results

### Genetic variation

The concatenated sequence alignment contained 88 sequences and 2676 positions, of which 1110 bp were sequenced for the *cytb* gene and 1566 bp for the *cox1* gene. The average base compositions of *cytb* and *cox1* were 46.1% and 46.0% (T), 18.6% and 18.3% (A), 23.5% and 23.6% (G), 11.8% and 12.1% (C) respectively, with AT-richness in the sequences. No insertions or deletions were detected. A total of 161 polymorphic sites were found, of which 150 were parsimony-informative and 11 were singleton-variable. These polymorphic sites identified 61 haplotypes within 88 isolates from fourteen localities (Table 1 and S1 Dataset.). Each sampled population and the total population have high Hd, accompanied by very low Pi (Table 1). The genetic divergence of *cytb* and *cox1* sequences of *S*. *erinaceieuropaei* collected from fourteen geographical locations ranged from 0 to 3.6% and 0 to 4.9%, respectively.

**Table 1:** Sampling haplotypes with frequencies and genetic diversities of *Spirometra erinaceieuropaei*.

Sample sites	SS	Haplotypes (Frequencies)	Pi±S.D.	Hd±S.D.
HeN-XX	4	Hap43(2), Hap44(1), Hap45(1)	0.00318±0.00157	0.833±0.222
HeN-ZZ	5	Hap50(1), Hap51(1), Hap52(2), Hap53(1)	0.00590±0.00120	0.900±0.161
HeN-KF	8	Hap35(5), Hap36(1), Hap37(1), Hap38(1)	0.00171±0.00067	0.643±0.184
HeN-ZK	5	Hap35(1), Hap46(1), Hap47(1), Hap48(1), Hap49(1)	0.00441±0.00087	1.000±0.126
HeN-LH	8	Hap35(1), Hap39(2), Hap40(3), Hap41(1), Hap42(1)	0.00226±0.00087	0.857±0.108
HuN-JS	6	Hap54(1), Hap55(2), Hap56(1), Hap57(1), Hap58(1)	0.00623±0.00099	0.933±0.122
HuN-CS	4	Hap59(2), Hap60(1), Hap61(1)	0.00343±0.00097	0.833±0.222
GZ-AS	6	Hap29(1), Hap30(1), Hap31(1), Hap32(1),Hap33(1), Hap34(1)	0.00600±0.00111	1.000±0.096
GZ-GY	6	Hap25(1), Hap26(1), Hap27(1), Hap28(3)	0.00685±0.00158	0.800±0.172
GX-GL	8	Hap1(1), Hap2(1), Hap3(1), Hap4(1), Hap5(3), Hap6(1)	0.00673±0.00178	0.893±0.111
GX-NN	8	Hap7(1), Hap8(2), Hap9(1), Hap10(1), Hap11(1), Hap12(2)	0.00690±0.00146	0.929±0.084
GX-YL	6	Hap10(2), Hap13(1), Hap14(1), Hap15(1),Hap16(1)	0.00800±0.00129	0.933±0.0122
HN-HK	5	Hap10(2), Hap17(1), Hap18(1), Hap19(1)	0.00845±0.00179	0.900±0.0161
HN-WZS	9	Hap10(1), Hap20(4), Hap21(1), Hap22(1),Hap23(1), Hap24(1)	0.00673±0.00127	0.833±0.0127
Total	88		0.01880±0.00044	0.985±0.005

SS, sampling size; Pi, nucleotide diversity; Hd, haplotype diversity; S.D., standard deviation.

### Phylogenetic diversity

**Fig 2:**
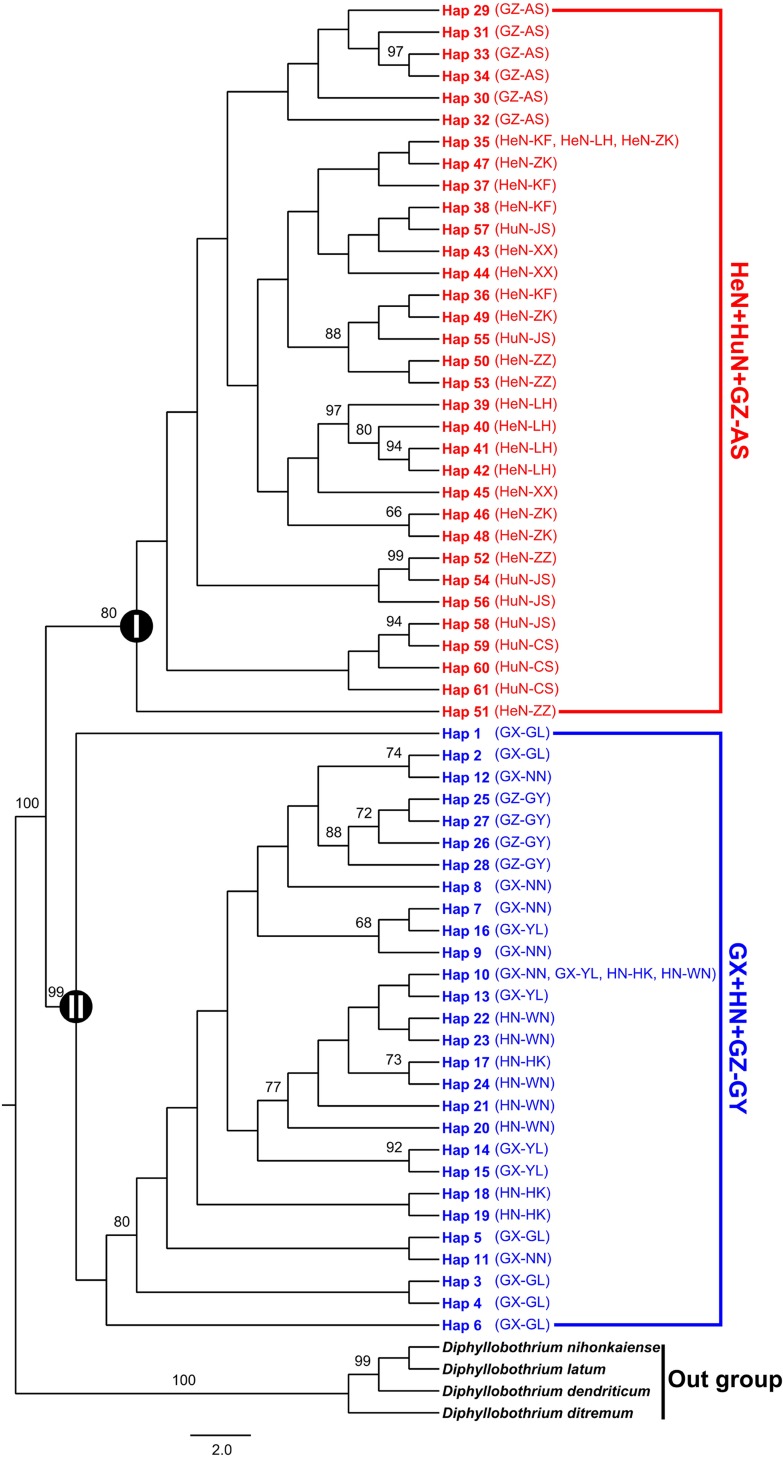
Maximum parsimony (MP) tree of the observed mtDNA haplotypes of *Spirometra erinaceieuropaei* isolates from central and southern China with four *Diphyllobothrium* species as outgroup. Numbers above branches represent the bootstrap values. Only bootstrap values above 60 are shown. Circled Roman numbers ‘I’ and ‘II’ refer to two main clades discussed in the text.

**Fig 3:**
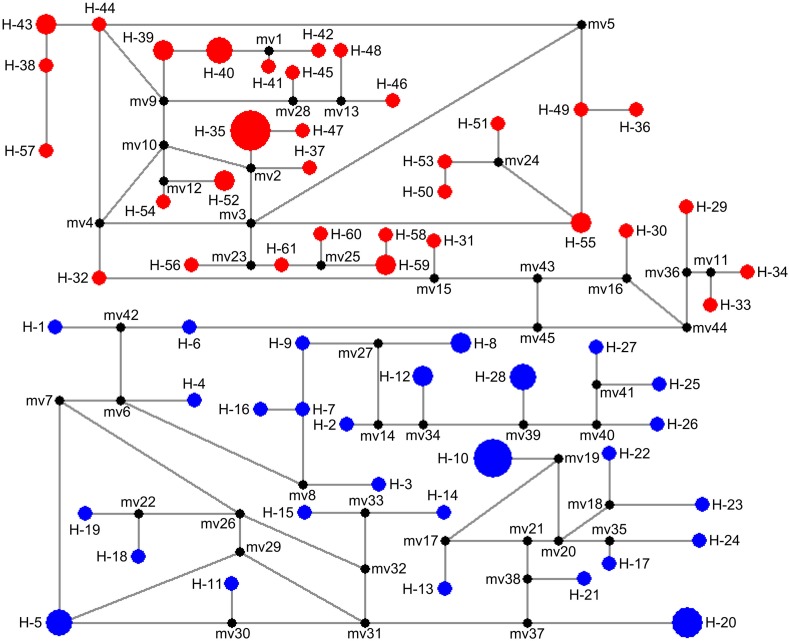
Median-joining network of mtDNA haplotypes of *Spirometra erinaceieuropaei* isolates from central and southern China. Each haplotype is represented by a circle, with the area of the circle proportional to its frequency. Samples from Clade I and Clade II are indicated by red and blue, respectively. Median vector (mv1-mv45) is indicated by black.

The 61 haplotypes of *S*. *erinaceieuropaei* formed two distinct clades (clade I and clade II) in all phylogenetic analyses based on three methods: maximum parsimony (Fig. 2), neighbor-joining (S1 Fig) and Bayesian inference (S2 Fig). Clade I included 46 individuals collected from Xinxiang (HeN-XX), Zhengzhou (HeN-ZZ), Kaifeng (HeN-KF), Zhoukou (HeN-ZK) and Luohe (HeN-LH) of Henan province in central China, Changsha (HuN-CS) and Jishou (HuN-JS) of Hunan province and Anshun (GZ-AS) of Guizhou province; Isolates within clade II came from Nanning (GX-NN), Guilin (GX-GL) and Yulin (GX-YL) of Guangxi Zhuang Autonomous Region, Haikou (HN-HK) and Wuzhishan (HN-WZS) of Hainan province and Guiyang (GZ-GY) of Guizhou province (Fig. 2, S1 and S2 Figs). In the haplotype median-joining network, the 61 haplotypes of *S*. *erinaceieuropaei* observed in the dataset also generated two subnetworks: clade I and clade II (Fig. 3).

### Population structure

Analysis of molecular variance indicated that most of the observed genetic variation occurs between the two clades (70.57%), whereas differentiation among fourteen endemic populations (HeN-XX, HeN-ZZ, HeN-KF, HeN-ZK, HeN-LH, HuN-JS, HuN-CS, GZ-AS, GZ-GY, GX-GL, GX-NN, GX-YL, HN-HK, HN-WZS) within groups only contributed 10.59% to the total population, and differentiation within fourteen endemic populations contributed 18.84% to the total population (Table 2). As described above, pairwise *F*_ST_ analyses were performed between specified regions (Table 3). In all estimated 105 pairwise *F*_ST_ values, only 9 were statistically insignificant. Pairwise *F*_ST_ values of regions within clade I (HeN+HuN+GZ-AS) and clade II (GX+HN+GZ-GY) were much lower than those between the two groups.

**Table 2:** Analysis of molecular variance (AMOVA) based on mtDNA sequences of the populations of *Spirometra erinaceieuropaei*.

Source of variation	d.f	Sum of squares	Variance components	Percentage of variation	Fixation Index (*F*_ST_)	*P*-value
Among groups	1	1245.998	27.57433	70.57	0.81159	0.0000
Among Populations within groups	12	397.037	4.13881	10.59		
Within populations	74	544.817	7.36239	18.84		
Total	87	2187.852	39.07553	100		

d.f = degrees of freedom.

**Table 3:** Estimates of pairwise *F*_ST_ of mtDNA sequences between *Spirometra erinaceieuropaei* populations.

Lable	Population	1	2	3	4	5	6	7	8	9	10	11	12	13	14
1	HeN-XX	0.0000													
2	HeN-ZZ	0.2530*	0.0000												
3	HeN-KF	0.2444*	0.3960**	0.0000											
4	HeN-ZK	0.0883	0.1727	0.0840	0.0000										
5	HeN-LH	0.5157**	0.5367**	0.6366**	0.4829**	0.0000									
6	HuN-JS	0.0988	-0.0393	0.1874**	0.0611	0.4337**	0.0000								
7	HuN-CS	0.4647*	0.3010**	0.5972**	0.3854**	0.6439**	0.1519	0.0000							
8	GZ-AS	0.3703**	0.3367**	0.4641**	0.3073**	0.5562**	0.2579**	0.3907**	0.0000						
9	GZ-GY	0.8067**	0.7730**	0.8596**	0.7883**	0.8421**	0.7707**	0.7968**	0.7580**	0.0000					
10	GX-GL	0.7968**	0.7611**	0.8445**	0.7819**	0.8429**	0.7664**	0.7927**	0.7585**	0.4609**	0.0000				
11	GX-NN	0.8082**	0.7842**	0.8520**	0.7942**	0.8448**	0.7852**	0.8107**	0.7714**	0.4087**	0.1997**	0.0000			
12	GX-YL	0.8021**	0.7686**	0.8572**	0.7893**	0.8529**	0.7743**	0.7996**	0.7637**	0.4862**	0.3198**	0.2028*	0.0000		
13	HN-HK	0.8023**	0.7598**	0.8620**	0.7877**	0.8567**	0.7684**	0.7967**	0.7596**	0.5010**	0.2551**	0.2889*	0.0319	0.0000	
14	HN-WZS	0.8126**	0.7789**	0.8560**	0.8028**	0.8564**	0.7847**	0.8075**	0.7755**	0.5865**	0.4235**	0.4298**	0.1694*	0.0914	0.0000

Significance of χ^2^

* *p*-values ≤ 0.05

** *p*-values ≤ 0.01.

### Divergence time

**Fig 4:**
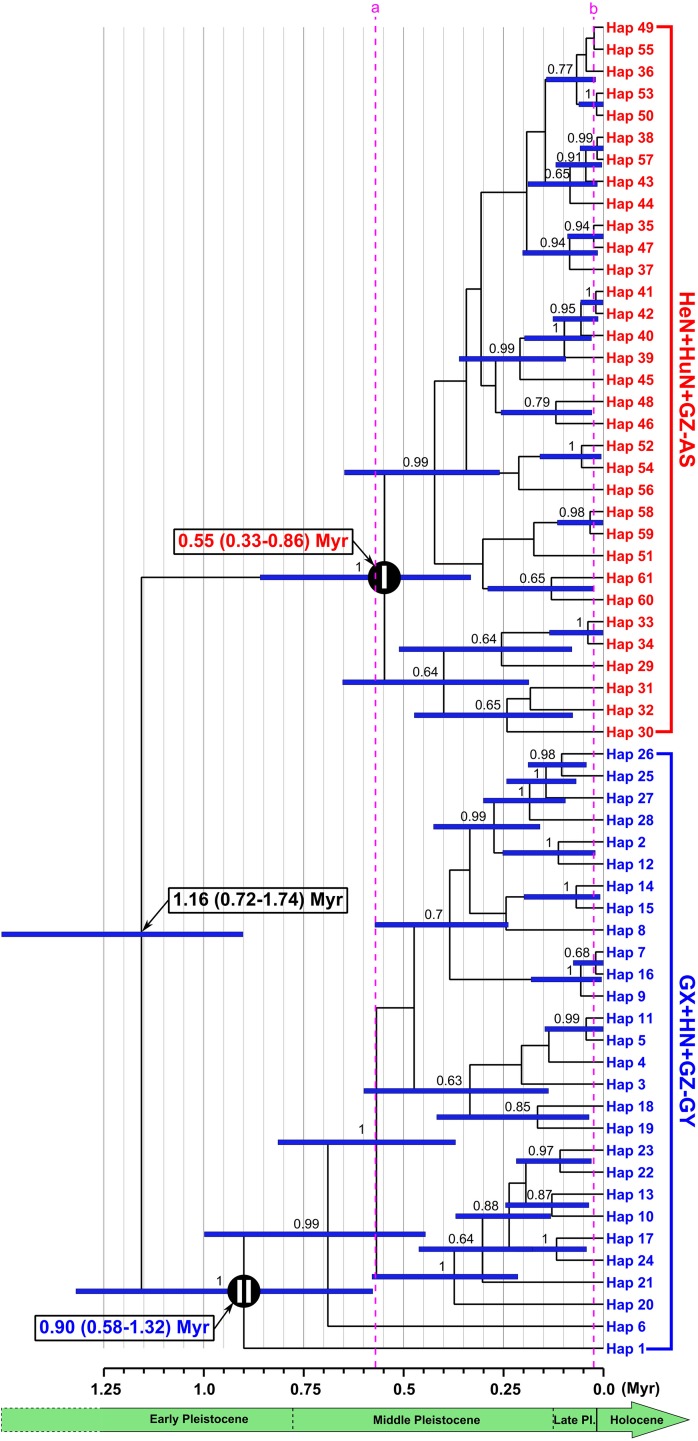
Phylogram of *Spirometra erinaceieuropaei* isolates from central and southern China with divergence time estimates based on mtDNA haplotypes. Blue bars at each node show 95% highest posterior density interval for the main nodes. Numbers above branches represent the Bayesian posterior probabilities. Only posterior probabilities above 0.6 are shown. Numbers in the square frames indicate the estimated age and 95% confidence intervals (shown in parenthesis). Circled Roman numbers ‘I’ and ‘II’ refer to two main clades discussed in the text. Dotted lines with lower case letters refer to divergence times discussed in the text.

According to the relaxed molecular clock analysis of the concatenated sequences of *cytb* and *cox1* genes (Fig. 4), the divergence time between clade I and clade II was calculated to have taken place in the early Pleistocene (1.16 Myr) with a 95% highest posterior density (HPD) of 0.72–1.74 Myr. The divergence time of the HeN+HuN+GZ-AS clade (clade I) was estimated to begin at 0.55 Myr (in the middle Pleistocene) with a 95% HPD of 0.33–0.86 Myr. The early branching of the GX+HN+GZ-GY clade (clade II) started in the early Pleistocene (0.90 Myr, with a 95% HPD of 0.58–1.32 Myr). Approximately 1.14 Myr elapsed from the earliest divergence of *S*. *erinaceieuropaei* lineage to the time when all major haplotypes are present.

### Demographic analysis

**Fig 5:**
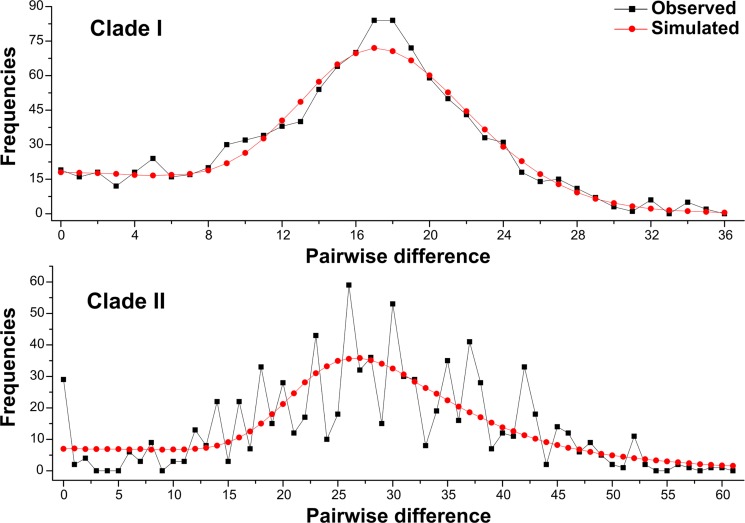
Mismatch distribution analysis for the Clade I and Clade II. The line charts represent the observed frequencies of pairwise differences among haplotypes.

**Fig 6:**
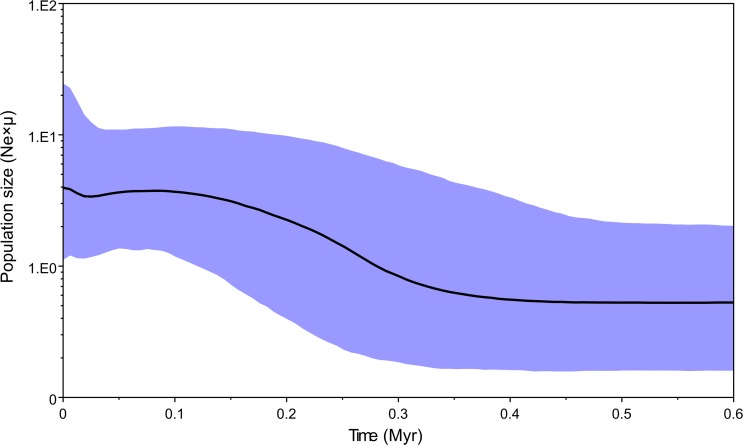
A Bayesian skyline plot derived from an alignment of mtDNA haplotypes of *Spirometra erinaceieuropaei* isolates in Clade I. The X-axis is in units of million years in the past and the Y-axis is Ne×μ (effective population size × mutation rate per site per generation). The median estimates are shown as thick solid lines, and the 95% HPD limits are shown by the blue areas.

The results of neutral test analyses were submitted in the Table 4. All values of Fu’s *F*_S_ of clade I, clade II and total population were negative, however, only the value of clade I was significant. Except clade I possessed a negative value of Tajima’s *D*, the values of Tajima’s *D* of both clade II and total population were positive, and all values of Tajima’s *D* were insignificant under significance levels *p* = 0.05. Mismatch distribution analyses showed a unimodal frequency distribution of pairwise differences in clade I (Fig. 5). All above results suggest demographic expansion in clade I. And a population expansion was identified after about 0.3 Myr (middle Pleistocene) by the result of BSP analysis of clade I (Fig. 6). However, both mismatch distribution analyses and the neutrality tests rejected a sudden population expansion in the clade II (Table 4 and Fig. 5).

**Table 4:** Mismatch and neutrality tests results of *Spirometra erinaceieuropaei* isolates from central and southern China.

Phylogroups	Neutrality Tests		Mismatch	
	Fu’s *F*_S_	Tajima’s *D*	SSD	Rag
Clade I	-7.75789*	-0.24064	0.00257 (0.80)	0.00541 (0.63)
Clade II	-0.78947	0.10978	0.00813 (0.17)	0.01905 (0.00)
Total population	-4.18213	1.95095	0.01084 (0.23)	0.00375 (0.05)

SSD = Sum of Squared deviation, Rag = Harpending's Raggedness index.

Significance * *p*-values ≤ 0.05. Number in parentheses is *P* value.

## Discussion

In our study, the AT-richness of *cytb* and *cox1* genes exceeded 64.7% and 64.3% respectively, which were consistent with the results of studies of other tapeworms such as the *Taenia* species [19,40] and the *Diphyllobothrium* species [21]. As suggested by Neigel and Avise [41], the nucleotide diversity (Pi) value is an important index to measure the level of genetic diversity, and Pi > 0.01 is considered to indicate a comparatively large variation in most animals. However, most Pi values were lower than 0.01 in this study, suggesting that there was low genetic variation of 88 isolates from 14 regions. The average genetic distances among 88 isolates from central and southern China are lower than 0.05. This is accord with the range of intraspecific genetic distance (0–0.05) reported by Nei et al. [42].

Some pioneering scientists have been concentrated their studies on the phylogeny of *S*. *erinaceieuropaei*. Okamoto et al. [43] evaluated the intraspecific variation of *S*. *erinaceieuropaei* and its phylogenetic relationship with *Diphyllobothrium* using partial sequences of the *cox1* gene, Dai et al. [44] examined sequence variability among and within three cestodes, *S*. *erinaceieuropaei*, *Taenia multiceps* and *T*. *hydatigena*, from different geographical origins in China based on partial *cox1*, *nad4* and ITS genes, Liu et al. [45] explored sequence variability in three mtDNA regions (*cox3*, *nad1* and *nad4*) in *S*. *erinaceieuropaei* spargana from different geographical regions in Hunan province of China, and Liang et al. [46] investigated sequence variability in *S*. *erinaceieuropaei* from dogs in Hunan province in China using partial *cox1* and a small subunit of ribosomal RNA (*rrnS*) region. All these studies supported the monophyly of *S*. *erinaceieuropaei* spargana isolates collected from different geographical locations or different hosts. Our analyses based on three phylogenetic inference methods (MP, NJ and BI) confirmed this conclusion too. In this study, *S*. *erinaceieuropaei* spargana isolates from central and southern China revealed in two distinct groups: HeN+HuN+GZ-AS group (clade I) and GX+HN+GZ-GY group (clade II) with high support values. The median-joining network yielded two subnetworks corresponding to the two clades in the phylogenetic tree (clade I and clade II), and there are no shared haplotypes between clades. Such distributions of haplotypes of *S*. *erinaceieuropaei* may be interpreted as being the result of population isolation [47].

The result obtained from AMOVA showed weak genetic variation within populations. The explanation may be that gene flow interrupts between the *S*. *erinaceieuropaei* isolates collected from different geographical locations [29]. Meanwhile, most pairwise *F*_ST_ values between *S*. *erinaceieuropaei* populations were higher than 0.25, indicating great genetic differentiation[48]. This evidenced that a long-term interruption of gene flow between two clades [49].

Estimation of divergence time from molecular data requires the selection of appropriate calibration information, such as fossil record or geological evidence [50]. Given a lack of fossils or geological events for *S*. *erinaceieuropaei*, we tended to use calibrating information based on substitution rates obtained from *Taenia* tapeworms for which there is a good fossil record or strong geological evidence dating a vicariance event [19,35]. Our data suggest that the *S*. *erinaceieuropaei* lineage separated 1.16 Myr ago, during early Pleistocene. This date is earlier than the divergence time between Asian lineage and African–American lineage of *Taenia solium* (in the middle Pleistocene) calculated using *cytb* and *cox1* genes [19,51]. The average age estimates of most of the haplotypes imply that they originated within a 0.88 Myr time window (∼0.02–0.90 Myr) about 0.26 Mya after the origin of *S*. *erinaceieuropaei*. And during about 0.57–0.02 Myr ago (between dotted line a and dotted line b in the Fig. 4), most of haplotypes appeared.

The divergence patterns of species can be driven by climatic fluctuations [52]. Tremendous climatic changes, particularly the Quaternary glaciations have made many plants and animals extinct and influenced the evolution and distribution of many plants and animals in China and its neighboring areas [53]. In our study, the neutrality test Fu’s *F*_S_ result of clade I was significantly negative, and mismatch distribution statistic support the assumption of population expansion of HeN+HuN+GZ-AS group in the past. The BEAST analysis showed that most of haplotypes appeared during the middle and late Pleistocene, coinciding with the Bayesian skyline plot (BSP) analysis: a population expansion occurred after about 0.3 Myr ago (in the middle Pleistocene). The climatic oscillations during glacial periods in the Quaternary may affected the demography and diversification of *S*. *erinaceieuropaei* from Henan and Hunan provinces in China. However, the demography and diversification pattern of GX+HN+GZ-GY group requires further research to clarify the definitive reason.

In conclusion, *Spirometra erinaceieuropaei* from central and southern China can be divided into two populations: the HeN+HuN+GZ-AS group and the GX+HN+GZ-GY group, and the two groups diverged from each other during the early Pleistocene. The climatic fluctuations in the Quaternary probably impacted the demography and diversification of the HeN+HuN+GZ-AS group.

## Supporting Information

S1 Dataset Data matrix of mtDNA haplotypes of *Spirometra erinaceieuropaei* used in this study. (DOC)

S1 Fig Neighbor-joining (NJ) tree of the observed mtDNA haplotypes of *Spirometra erinaceieuropaei* isolates from central and southern China with four *Diphyllobothrium* species as outgroup. Numbers above branches represent the bootstrap values. Only bootstrap values above 60 are shown. Circled Roman numbers ‘I’ and ‘II’ refer to two main clades discussed in the text.
(TIF)

S2 Fig Bayesian inference (BI) tree of the observed mtDNA haplotypes of *Spirometra erinaceieuropaei* isolates from central and southern China with four *Diphyllobothrium* species as outgroup. Numbers above branches represent the Bayesian posterior probabilities. Only posterior probabilities above 0.6 are shown. Circled Roman numbers ‘I’ and ‘II’ refer to two main clades discussed in the text.
(TIF)

S1 Table *Spirometra erinaceieuropaei* sampling and data summary for this study. (DOC)
