# Supplementary material for: Genetic Structure Analysis of Spirometra erinaceieuropaei Isolates from Central and Southern China
Source: PLoS One. 2015 Mar 20;10(3):e0119295. doi: 10.1371/journal.pone.0119295 (PMC4368571; doi:10.1371/journal.pone.0119295)
Supplement: S1 Dataset — (DOC) [file pone.0119295.s001.doc]

**File S1.** Data matrix of mtDNA haplotypes of *Spirometra erinaceieuropaei* used in this study.

>Hap_1

ATGGTTTTGTTGTTGCGTCGTAATGTGGTTGATTTGCCTACTAATTATTCTCTTAATTATTATTGATGTAGTGGTTTTATGATTTCGGCTTTTATGGTAGTTCAGGTAATCACTGGTGTGATTCTTTCACTTTTGTATGTGGCTGATTCAAGATTAAGTTTTCGTTGTGTTATGGATTTGAGAAAAGATTCTTTTTTTACTTGGGGAGTGCGCTATTGACACATTTGGGGGGTTAGTATTTTGTTTGTCCTGTTTTTTGTTCATATGGGACGTGCCTTATATTATTCAAGTTATACTAAGAAGGGGGTATGGAAAGTGGGGTTTATTTTATATCTTTTAACTATGGCCGAGGCTTTTTTGGGTTATATTTTACCTTGACATCAAATGTCATATTGGGCTGCTACTGTTTTGACGGCTATTGCCGAGAGTATTCCCTTAGTTGGTCCTACGGTGTTTAAGTATTTGGTGGGGGGTTTTTCTGTAACTAAAGTAACTTTGGTTCGTGTATTTTCAGCTCATGTTTGTTTAGGTTTTGTAATTTTAGGTTTAATGATTTTGCATCTTTTTTATTTGCATTCTTCCGGGTCCAATAACCCTTTATTTTCTTCTTTTGGGTATGGGGATGTTGTTTATTTCCACTCTTATTTCACCACTAAGGATTTTTTTTGTTTGGTCGTTCTATGCTGTATTTTGGTTGGATTTATGTGGTTGGTTCCTGATTTGGTGGTAGATACAGAGGGTTATCTAGAATCTGATCCTTTGGTGACTCCGGTGTCTATAAAGCCTGAGTGATATTTTTTGATTTATTATGCTATGCTTCGTTCTGTTGAGTCTAAGATAGGTGGTCTAGTGTTGGTGGCTAGATTATTATTTTTTATGTGGGTCCCAACTTTTAAAGACTCTAGTTCATATTTTGTTATTCGACAGGTGGTTTTTTGGAGTTTCGTTTGTCTTTTAGTTGGGTTGACTTATTTAGGCTCATGTCACCCTGAGTATCCTTATTTGGGTATTTGTCAGTTATTTTCGGTTGGTGCTGTAGCTTTTATGTTTATTTATAAGCTATTTTGATCGAGTTATACTAAGTTGGGTTTTAGTATTTTTTTAGGATAAATGGCTAAGTTTAGTTTTTTTAGTTGGCTTTTTACGCTAGATCATAAGCGTGTGGGTATGATTTATACTTTAATTGGGATATGATCAGGTTTTGTTGGGTTGAGTTTTAGGGTAATGATACGTGTTAATTTTGTTGAGCCTTATTTTAATGTAATTTCTTCGGACTGTTATAAATTTTTGATAACTAATCATGGTATTATTATGATTTTTTTTTTTTTGATGCCGGGGTTGATTGGGGGTTTGGGAAATTATTTAATTCCTTTATTATCCGGTTTACCAAATTTAAATTTACCCCGGTTGAAACCCTTGAAAGCAGGGTTGCTTTTCCCTTCTATTTTATTTCTGGGGTTAAGAATGGGTTGGGGTGCGGGAAAAGGGGGAACTTTTTACCCCCCTCTCTCTTCTTCCCTTTTTAGGAATAGCCGGGGGGCCAACCTTTTGAGGTTTTCTTTACATTTGGCGGGCCTTTCAAGTTTGTTGGGTTCAATAAATTTTATATGTACTCTTTATTCGGCTTTTGTTGATAATTTTGTGTCTCGAAGTTCTATTTTGTTGTGGTCTTATTTATTTACTTCTATCCTTTTATTGTTAACTATTCCTGTGTGGGCTGCTGCTATTACAATGTTGTTGTTTGATCGTAAATTTGGTTCGGCTTTTTTTGATCCTTTGGGTGGGGGTGATCCCGTTTTGTTCCAACATATGTTTTGGTTTTTTGGGCATCCAGAAGTTTATGTATTGATTTTGCCTGGTTTTGGTATGGTAAGTCATGTGTGTAGTAAATTAGGTTGTTCATATGATACTTTTGGTTTTTATGGTTTACTATTTGCTATGTTTTCTATAGTGTGTTTAGGTAGCGTTGTTTGGGGTCACCACATGTTTACTGTGGGGTTGGATGTGAAGACTGCTGTTTTCTTTAGTTCTGTGACCATGATTATTGGGGTTCCCACGGGTATAAAGGTGTTTTCTTGGCTTTATATGATTTTAAATAGTCGTGTTTCGTTGCGTGAGCCTGTGTTTTGATGGGTTTTATCTTTTATTGTGTTGTTTACTATGGGTGGTGTTACTGGTATAATTCTTTCTGCTTGTGTGTTGGATAAAATTTTGCATGACACGTGGTTTGTGGTGGCTCATTTTCATTATGTTATGTCTTTGGGTTCTTATATTAGGGTTATTATATTTTTTGTTTGGTGATGGCCTGTTATCACAGGGGTTAGCCTGAATAAGTATTTGTTACAGTGTCATTGTATAGTATCAAATGTGGGCTTTAATTTGTGTTTTTTTCCTATGCATTATTTTGGTATTTGTGGTTTACCTCGGCGTGTTTGTGTGTATGAGTCAGGGTACGCTTGAGTTAATATGCTTTGTTCAATAGGTTCTTTTGTTTCTGCCTTTAGTGGTTGCTTTTTTATTTTTATTTTATGGGAGTCTTTAGCTAAAAAGAATGTTGTTATAGGTTATTATGGTAGTTCTTCAACTTTGCTTAATTTGTGTTGATCGCCAGTGCCTTACCACAGTAATTTTTTTGTGCGCGGATTATTTGTTGATTATTCTGTATTGGCTTTTTAG

>Hap_2

ATGGTTTTGTTGTTGCGTCGTAATGTGGTTGATTTGCCTACTAATTATTCTCTTAATTATTATTGATGTAGTGGTTTTATGATTTCGGCTTTTATGGTAGTTCAGGTAATCACTGGTGTGATTCTTTCACTTTTGTATGTGGCTGATTCAAGATTAAGTTTTCGTTGTGTTATGGATTTGAGAAAAGATTCTTTTTTTACTTGGGGAGTGCGCTATTGACACATTTGGGGGGTTAGTATTTTGTTTGTCCTGTTTTTTGTTCATATGGGACGTGCCTTATATTATTCAAGTTATACTAAGAAGGGGGTATGGAAAGTGGGGTTTATTTTATATCTTTTAACTATGGCCGAGGCTTTTTTGGGTTATATTTTACCTTGACATCAAATGTCATATTGGGCTGCTACTGTTTTGACGGCTATTGCCGAGAGTATTCCCTTAGTCGGTCCTACGGTGTTTAAGTATTTGGTGGGGGGGTTTTCTGTAACCAAAGTAACTTTGGTTCGTGTATTTTCAGCTCATGTTTGTTTAGGTTTTGTAATTTTAGGTTTAATGATTTTGCATCTTTTTTATTTGCATTCTTCTGGGTCCAATAACCCTTTATTTTCTTCTTTTGGGTATGGGGATGTTGTTTATTTCCACTCTTATTTCACCACTAAGGATTTTTTTTGTTTGGTCGTTCTATGCTGTATTTTGGTTGGATTTATGTGGTTGGTTCCTGATTTGGTGGTAGATACGGAGGGTTATCTAGAATCTGATCCTCTGGTGACTCCGGTGTCTATAAAGCCTGAGTGATATTTTTTGATTTATTATGCTATGCTTCGTTCTGTTGAGTCTAAGATAGGTGGTCTAGTGTTGGTGGCTAGATTATTATTTTTTATGTGGGTCCCAACTTTTAAAGACTCTAGTTCATATTTTGTTATTCGACAGGTGATTTTTTGGAGTTTCGTTTGTCTTTTTGTTGGGTTGACTTATTTAGGCTCATGTCACCCTGAGTACCCTTATTTGGGTATTTGTCAGTTATTTTCGGTTGGTGCTGTAGCTTTTATGTTTATTTATAAGCTATTTTGATCGAGTTATACTAAGTTGGGTTTTAGTATTTTTTTAGGATAAATGGCTAAGTTTAGTTTTTTTAGTTGGCTTTTTACGCTAGATCATAAGCGTGTGGGTATGATTTATACTTTAATTGGGATATGATCGGGTTTTGTTGGGTTGAGTTTTAGGGTAATGATACGTGTTAATTTTGTTGAGCCTTATTTTAATGTAATTTCTTCGGACTGTTATAAATTTTTGATAACTAATCATGGTATTATTATGATTTTTTTTTTTTTGATGCCCGGGTTGATGGGGGGTTTGGGAAATTATTTGATTCCTTTATTATCCGGTTTACCAAATTTAAATTTACCCCGGTTGAAACCCTTGAAAGCAGGGTGGCTTTTCCCTCCTATTTTATTTCTGGGGTGAAGAAGGGGTTGGGGGGCGGGAAAAGGGGGGACTTTTTACCCCCCCCTCTCTTCTTCCCTTTTTAGCAATAGCCGGGGGGCCAACCTTTTGAGGTTTTCTTTACATTTGGCGGGTCTTTCAAGTTTGTTGGGTTCAATAAATTTTATATGTACTCTTTATTCGGCTTTTGTTGATAATTTTGTGTCTCGAAGTTCTATTTTGTTGTGATCTTATTTATTTACTCTAATCCTTTTATGGTTAACTATTCCTGTGTGGGCTGCTGCTATTACAATGTTGTTGTTTGATCGTAAATTTGGTTCGGCTTTTTTTGATCCTTTGGGTGGGGGTGATCCCGTTTTGTTTCAACATATGTTTTGGTTTTTTGGGCATCCGGAAGTTTATGTATTGATTTTGCCTGGTTTTGGTATGGTAAGTCATGTGTGTAGTAAATTAGGTTGTTCATATGATACTTTTGGTTTTTATGGTTTACTATTTGCTATGTTTTCTATAGTGTGTTTAGGTAGCGTGGTTTGGGGCCACCACATGTTTACTGTGGGGTTGGATGTGAAGACTGCTGTTTTCTTTAGTTCTGTGACCATGATTATTGGGGTTCCCACGGGTATAAAGGTGTTTTCTTGGCTTTATATGATTTTAAATAGTCGTGTTTCGTTGCGTGAGCCTGTGTTTTGATGGGTTTTATCTTTTATTGTGTTGTTTACTATGGGTGGTGTTACTGGTATAATTCTTTCTGCTTGTGTGTTGGATAAAATTTTGCATGACACGTGGTTTGTGGTGGCTCATTTTCATTATGTTATGTCTTTGGGTTCTTATATTAGGATTATTATATTTTTTGTTTGGTGATGGCCTGTTATCACAGGGGTTAGCCTGAATAAGTATTTGTTACAGTGTCATTGTATAGTATCAAATGTGGGTTTTAATTTGTGTTTTTTCCCTATGCATTATTTTGGTATTTGTGGTTTACCTCGGCGTGTTTGTGTGTATGAGTCAGGGTACGCTTGAGTTAATATGCTTTGTTCAATAGGTTCTTTTGTTTCTGCCTTTAGTGGTTGCTTTTTTATTTTTATTTTATGGGAGTCTTTAGCTAAAAAGAATGTTGTTATAGGTTATTATGGTAGTTCTTCAACTTTGCTTAATCTGTGTTGGTCGCCAGTGCCTTATCACAGTAATTTTTTTGTGCGCGGATTATTTGTTGATTATTCTGTATTGGCTTTTTAG

>Hap_3

ATGGTTTTGTTGTTGCGTCGTAATGTGGTTGATTTGCCTACTAATTATTCTCTTAATTATTATTGATGTAGTGGTTTTATGATTTCGGCTTTTATGGTAGTTCAGGTAATCACTGGTGTGATTCTTTCACTTTTGTATGTGGCTGATTCAAGATTAAGTTTTCGTTGTGTTATGGATTTGAGAAAAGATTCTTTTTTTACTTGGGGAGTGCGCTATTGACACATTTGGGGGGTTAGTATTTTGTTTGTCCTGTTTTTTGTTCATATGGGACGTGCCTTATATTATTCAAGTTATACTAAGAAGGGGGTATGGAAAGTGGGGTTTATTTTATATCTTTTAACTATGGCCGAGGCTTTTTTGGGTTATATTTTACCTTGACATCAAATGTCATATTGGGCTGCTACTGTTTTGACGGCTATTGCCGAGAGTATTCCCTTAGTCGGTCCTACGGTGTTTAAGTATTTGGTGGGGGGTTTTTCTGTAACCAAAGTAACTTTGGTTCGTGTATTTTCAGCTCATGTTTGTTTAGGTTTTGTAATTTTAGGTTTAATGATTTTGCATCTTTTTTATTTGCATTCTTCTGGGTCCAATAACCCTTTATTTTCTTCTTTTGGGTATGGGGATGTTGTTTATTTCCACTCTTATTTCACCACTAAGGATTTTTTTTGTTTGGTCGTTCTATGCTGTATTTTGGTTGGATTTATGTGGTTGGTTCCTGATTTGGTGGTAGATACGGAGGGTTATCTAGAATCTGATCCTCTGGTGACCCCGGTGTCTATAAAGCCTGAGTGATATTTTTTGATTTATTATGCTATGCTTCGTTCTGTTGAGTCTAAGATAGGTGGTCTAGTGTTGGTGGCTAGATTATTATTTTTTATGTGGGTCCCAACTTTTAAAGACTCTAGTTCATATTTTGTTATTCGACAGGTGATTTTTTGGAGTTTCGTTTGTCTTTTTGTTGGGTTGACTTATTTAGGCTCATGTCACCCTGAGTACCCTTATTTGGGAATTTGTCAGTTATTTTCGGTTGGTGCTGTAGCTTTTATGTTTATTTATAAGCTATTTTGATCGAGTTATACTAAGTTGGGTTTTAGTATTTTTTTAGGATAAATGGCTAAGTTTAGTTTTTTTAGTTGGCTTTTTACGCTAGATCATAAGCGTGTGGGTATGATTTATACTTTAATTGGGATATGATCGGGTTTTGTTGGGTTGAGTTTTAGGGTAATGATACGTGTTAATTTTGTTGAGCCTTATTTTAATGTAATTTCTTCGGACTGTTATAAATTTTTGATAACTAATCATGGTATTATTATGATTTTTTTTTTTTTGATGCCCGGGTTGATGGGGGGTTTGGGAAATTATTTGATTCCTTTATTACCCGGTTTACCAAATTTAAATTTACCCCGGTTGAAACCCTTGAAAGCAGGGTTGCTTTTTCCTTCTATTTTATTTCTGGGGTTGAGTAGGGGTTGGGGGGCGGGAAAAGGGGGAACTTTTTACCCCCCCCTCTCTTCTTCCCTTTTTAGCAATAGCCGGGGGGCCAATCTTTTGAGGTTTTCTTTACATTTGGCGGGCCTTTCAAGTTTGTTGGGTTCAATAAATTTTATATGTACTCTTTATTCGGCTTTTGTTGATAATTTTGTGTCTCGAAGTTCTATTTTGTTGTGGTCTTATTTATTTACTTCAATCCTTTTATTGTTAACTATTCCTGTGTGGGCTGCTGCTATTACAATGTTGTTGTTTGATCGTAAATTTGGTTCGGCTTTTTTTGATCCTTTGGGTGGGGGTGATCCCGTTTTGTTCCAACATATGTTTTGGTTTTTTGGGCATCCGGAAGTTTATGTATTGATTTTGCCTGGTTTTGGTATGGTAAGTCATGTGTGTAGTAAATTAGGTTGTTCATATGATACTTTTGGTTTTTATGGTTTACTATTTGCTATGTTTTCTATAGTGTGTTTAGGTAGCGTGGTTTGGGGCCACCACATGTTTACTGTGGGGTTGGATGTGAAGACTGCTGTTTTCTTTAGTTCTGTGACCATGATTATTGGGGTTCCCACGGGTATAAAGGTGTTTTCTTGGCTTTATATGATTTTAAATAGTCGTGTTTCGTTGCGTGAGCCTGTGTTTTGATGGGTTTTATCTTTTATTGTGTTGTTTACTATGGGTGGTGTTACTGGTATAATTCTTTCTGCTTGTGTGTTGGATAAAATTTTGCATGACACGTGGTTTGTGGTGGCTCATTTTCATTATGTTATGTCTTTGGGTTCTTATATTAGGATTATTATATTTTTTGTTTGGTGATGGCCTGTTATCACAGGGGTTAGCCTGAATAAGTATTTGTTACAGTGTCATTGTATAGTATCAAATGTGGGTTTTAATTTGTGTTTTTTCCCTATGCATTATTTTGGTATTTGTGGTTTACCTCGGCGTGTTTGTGTGTATGAGTCAGGGTACGCTTGAGTTAATATGCTTTGTTCAATAGGTTCTTTTGTTTCTGCCTTTAGTGGTTGCTTTTTTATTTTTATTTTATGGGAGTCTTTAGCTAAAAAGAATGTTGTTATAGGTTATTATGGTAGTTCTTCAACTTTGCTTAATCTGTGTTGGTCGCCAGTGCCTTATCACAGTAATTTTTTTGTGCGCGGATTATTTGTTGATTATTCTGTATTGGCTTTTTAG

>Hap_4

ATGGTTTTGTTGTTGCGTCGTAATGTGGTTGATTTGCCTACTAATTATTCTCTTAATTATTATTGATGTAGTGGTTTTATGATTTCGGCTTTTATGGTAGTTCAGGTAATCACTGGTGTGATTCTTTCACTTTTGTATGTGGCTGATTCAAGATTAAGTTTTCGTTGTGTTATGGATTTGAGAAAAGATTCTTTTTTTACTTGGGGAGTGCGCTATTGACACATTTGGGGGGTTAGTATTTTGTTTGTCCTGTTTTTTGTTCATATGGGACGTGCCTTATATTATTCAAGTTATACTAAGAAGGGGGTATGGAAAGTGGGGTTTATTTTATATCTTTTAACTATGGCCGAGGCTTTTTTGGGTTATATTTTACCTTGACATCAAATGTCATATTGGGCTGCTACTGTTTTGACGGCTATTGCCGAGAGTATTCCCTTAGTCGGTTCTACGGTGTTTAAGTATTTGGTGGGGGGTTTTTCTGTAACCAAAGTAACTTTGGTTCGTGTATTTTCAGCTCATGTTTGTTTAGGTTTTGTAATTTTAGGTTTAATGATTTTGCATCTTTTTTATTTGCATTCTTCTGGGTCCAATAACCCTTTATTTTCTTCTTTTGGGTATGGGGATGTTGTTTATTTCCACTCTTATTTCACCACTAAGGATTTTTTTTGTTTGGTCGTTCTATGCTGTATTTTGGTTGGATTTATGTGGTTGGTTCCTGATTTGGTGGTAGATACGGAGGGTTATCTAGAATCTGATCCTCTGGTGACTCCGGTGTCTATAAAGCCTGAGTGATATTTTTTGATTTATTATGCTATGCTTCGTTCTGTTGAGTCTAAGATAGGTGGTCTAGTGTTGGTGGCTAGATTATTATTTTTTATGTGGGTCCCAACTTTTAAAGACTCTAGTTCATATTTTGTTATTCGACAGGTGATTTTTTGGAGTTTCGTTTGTCTTTTTGTTGGGTTGACTTATTTAGGCTCATGTCACCCTGAGTACCCTTATTTGGGTATTTGTCAGTTATTTTCGGTTGGTGCTGTAGCTTTTATGTTTATTTATAAGCTATTTTGATCGAGTTATACTAAGTTGGGTTTTAGTATTTTTTTAGGATAAATGGCTAAGTTTAGTTTTTTTAGTTGGCTTTTTACGCTAGATCATAAGCGTGTGGGTATGATTTATACTTTAATTGGGATATGATCGGGTTTTGTTGGGTTGAGTTTTAGGGTAATGATACGTGTTAATTTTGTTGAGCCTTATTTTAATGTAATTTCTTCGGACTGTTATAAATTTTTGATAACTAATCATGGTATTATTATGATTTTTTTTTTTTTGATGCCCGGGTTGATGGGGGGTTTTGGAAATTATTTGATTCCTTTATTACCCGGTTTACCAAATTTAAATTTACCCCGGTTGAAACCCTTGAAAGCAGGGTTGCTTTTTCCTTCTATTTTATTTCTGGGGTTGAGTATGGGTTGGGGGGCGGGAAAAGGGGGAACTTTTTACCCCCCCCTCTCTTCTTCCCTTTTTAGCAATAGCCGGGGGGCCAATCTTTTGAGGTTTTCTTTACATTTGGCGGGCCTTTCAAGTTTGTTGGGTTCTATAAATTTTATATGTACTCTTTATTCGGCTTTTGTTGATAATTTTGTGTCTCGAAGTTCTATTTTGTTGTGGTCTTATTTATTTACTTCAATTCTTTTATTGTTAACTATTCCTGTGTGGGCTGCTGCTATTACAATGTTGTTGTTTGATCGTAAATTTGGTTCGGCTTTTTTTGATCCTTTGGGTGGGGGTGATCCCGTTTTGTTCCAACATATGTTTTGGTTTTTTGGGCATCCGGAAGTTTATGTATTGATTTTGCCTGGTTTTGGTATGGTAAGTCATGTGTGTAGTAAATTAGGTTGTTCATATGATACTTTTGGTTTTTATGGTTTACTATTTGCTATGTTTTCTATAGTCTGTTTAGGTAGCGTGGTTTGGGGCCACCACATGTTTACTGTGGGGTTGGATGTGAAGACTGCTGTTTTCTTTAGTTCTGTGACCATGATTATTGGGGTTCCCACGGGTATAAAGGTGTTTTCTTGGCTTTATATGATTTTAAATAGTCGTGTTTCGTTGCGTGAGCCTGTGTTTTGATGGGTTTTATCTTTTATTGTGTTGTTTACTATGGGTGGTGTTACTGGTATAATTCTTTCTGCTTGTGTGTTGGATAAAATTTTGCATGACACGTGGTTTGTGGTGGCTCATTTTCATTATGTTATGTCTTTGGGTTCTTATATTAGGATTATTATATTTTTTGTTTGGTGATGGCCTGTTATCACAGGGGTTAGCCTGAATAAGTATTTGTTACAGTGTCATTGTATAGTATCAAATGTGGGTTTTAATTTGTGTTTTTTCCCTATGCATTATTTTGGTATTTGTGGTTTACCTCGGCGTGTTTGTGTGTATGAGTCAGGGTACGCTTGAGTTAATATGCTTTGTTCAATAGGTTCTTTTGTTTCTGCCTTTAGTGGTTGCTTTTTTATTTTTATTTTATGGGAGTCTTTAGCTAAAAAGAATGTTGTTATAGGTTATTATGGTAGTTCTTCAACTTTGCTTAATCTGTGTTGGTCGCCAGTGCCTTATCACAGTAATTTTTTTGTGCGCGGATTATTTGTTGATTATTCTGTATTGGCTTTTTAG

>Hap_5

ATGGTTTTGTTGTTGCGTCGTAATGTGGTTGATTTGCCTACTAATTATTCTCTTAATTATTATTGATGTAGTGGTTTTATGATTTCGGCTTTTATGGTAGTTCAGGTAATCACTGGTGTGATTCTTTCACTTTTGTATGTGGCTGATTCAAGATTAAGTTTTCGTTGTGTTATGGATTTGAGAAAAGATTCTTTTTTTACTTGGGGAGTGCGCTATTGACACATTTGGGGGGTTAGTATTTTGTTTGTCCTGTTTTTTGTTCATATGGGACGTGCCTTATATTATTCAAGTTATACTAAGAAGGGGGTATGGAAAGTGGGGTTTATTTTATATCTTTTAACTATGGCCGAGGCTTTTTTGGGTTATATTTTACCTTGACATCAAATGTCATATTGGGCTGCTACTGTTTTGACGGCTATTGCCGAGAGTATTCCCTTAGTCGGTCCTACGGTGTTTAAGTATTTGGTGGGGGGTTTTTCTGTAACCAAAGTAACTTTGGTTCGTGTATTTTCAGCTCATGTTTGTTTAGGTTTTGTAATTTTAGGTTTAATGATTTTGCATCTTTTTTATTTGCATTCTTCTGGGTCCAATAACCCTTTATTTTCTTCTTTTGGGTATGGGGATGTTGTTTATTTCCACTCTTATTTCACCACTAAGGATTTTTTTTGTTTGGTCGTTCTATGCTGTATTTTGGTTGGATTTATGTGGTTGGTTCCTGATTTGGTGGTAGATACGGAGGGTTATCTAGAATCTGATCCTCTGGTGACTCCGGTGTCTATAAAGCCTGAGTGATATTTTTTGATTTATTATGCTATGCTTCGTTCTGTTGAGTCTAAGATAGGTGGTCTAGTGTTGGTGGCTAGATTATTATTTTTTATGTGGGTCCCAACTTTTAAAGACTCTAGTTCATATTTTGTTATTCGACAGGTGATTTTTTGGAGTTTCGTTTGTCTTTTTGTTGGGTTGACTTATTTAGGCTCATGTCACCCTGAGTACCCTTATTTGGGTATTTGTCAGTTATTTTCGGTTGGTGCTGTAGCTTTTATGTTTATTTATAAGCTATTTTGATCGAGTTATACTAAGTTGGGTTTTAGTATTTTTTTAGGATAAATGGCTAAGTTTAGTTTTTTTAGTTGGCTTTTTACGCTAGATCATAAGCGTGTGGGTATGATTTATACTTTAATTGGGATATGATCGGGTTTTGTTGGGTTGAGTTTTAGGGTAATGATACGTGTTAATTTTGTTGAGCCTTATTTTAATGTAATTTCTTCGGACTGTTATAAATTTTTGATAACTAATCATGGTATTATTATGATTTTTTTTTTTTTGATGCCCGGGTTGATGGGGGGTTTTGGAAATTATTTGATTCCTTTATTACCCGGTTTACCAAATTTAAATTTACCCCGGTTGAAACCCTTGAAAGCAGGGTTGCTTTTTCCTTCTATTTTATTTCTGGGGTTGAGTATGGGTTGGGGGGCGGGAAAAGGGGGAACTTTTTACCCCCCCCTCTCTTCTTCCCTTTTTAGCAATAGCCGGGGGGCCAATCTTTTGAGGTTTTCTTTACATTTGGCGGGCCTTTCAAGTTTGTTGGGTTCAATAAATTTTATATGTACTCTTTATTCGGCTTTTGTTGATAATTTTGTGTCTCGAAGTTCTATTTTGTTGTGGTCTTATTTATTTACTTCTATCCTTTTATTGTTAACTATTCCTGTGTGGGCTGCTGCTATTACAATGTTGTTGTTTGATCGTAAATTTGGTTCGGCTTTTTTTGATCCTTTGGGTGGGGGTGATCCCGTTTTGTTCCAACATATGTTTTGGTTTTTTGGGCATCCGGAAGTTTATGTATTGATTTTGCCTGGTTTTGGTATGGTAAGTCATGTGTGTAGTAAATTAGGTTGTTCATATGATACTTTTGGTTTTTATGGTTTACTGTTTGCTATGTTTTCTATAGTGTGTTTAGGTAGCGTGGTTTGGGGCCACCACATGTTTACTGTGGGGTTGGATGTGAAGACTGCTGTTTTCTTTAGTTCTGTGACCATGATTATTGGGGTTCCCACGGGTATAAAGGTGTTTTCTTGGCTTTATATGATTTTAAATAGTCGTGTTTCGTTGCGTGAGCCTGTGTTTTGATGGGTTTTATCTTTTATTGTGTTGTTTACTATGGGTGGTGTTACTGGTATAATTCTTTCTGCTTGTGTGTTGGATAAAATTTTGCATGACACGTGGTTTGTGGTGGCTCATTTTCATTATGTTATGTCTTTGGGTTCTTATATTAGGATTATTATATTTTTTGTTTGGTGATGGCCTGTTATCACAGGGGTTAGCCTGAATAAGTATTTGTTACAGTGTCATTGTATAGTATCAAATGTGGGTTTTAATTTGTGTTTTTTCCCTATGCATTATTTTGGTATTTGTGGTTTACCTCGGCGTGTTTGTGTGTATGAGTCAGGGTACGCTTGAGTTAATATGCTTTGTTCAATAGGTTCTTTTGTTTCTGCCTTTAGTGGTTGCTTTTTTATTTTTATTTTATGGGAGTCTTTAGCTAAAAAGAATGTTGTTATAGGTTATTATGGTAGTTCTTCAACTTTGCTTAATCTGTGTTGGTCGCCAGTGCCTTATCACAGTAATTTTTTTGTGCGCGGATTATTTGTTGATTATTCTGTATTGGCTTTTTAG

>Hap_6

ATGGTTTTGTTGTTGCGTCGTAATGTGGTTGATTTGCCTACTAATTATTCTCTTAATTATTATTGGTGTAGTGGTTTTATGATTTCGGCTTTTATGGTAGTTCAGGTAATCACTGGTGTGATTCTTTCACTTTTGTATGTGGCTGATTCAAGATTAAGTTTTCGTTGTGTTATGGATTTGAGAAAAGATTCTTTTTTTACTTGGGGAGTGCGCTATTGACACATTTGGGGGGTTAGTATTTTGTTTGTCCTGTTTTTTGTTCATATGGGTCGTGCCTTATATTATTCAAGTTATACTAAGAAGGGGGTATGGAAAGTGGGGTTTATTTTATATCTTTTAACTATGGCCGAGGCTTTTTTGGGTTATATTTTACCTTGACATCAAATGTCATATTGGGCTGCTACTGTTTTGACGGCTATTGCCGAGAGTATTCCCTTAGTCGGTCCTACGGTGTTTAAGTATTTGGTGGGGGGGTTTTCTGTAACCAAAGTAACTTTGGTTCGTGTATTTTCAGCTCATGTTTGTTTAGGTTTTGTAATTTTAGGTTTAATGATTTTGCATCTTTTTTATTTGCATTCTTCTGGGTCTAATAACCCTTTATTTTCTTCTTTTGGGTATGGGGATGTTGTTTATTTCCACTCTTATTTTACCACTAAGGATTTTTTTTGTTTGGTTGTTCTATGCTGTATTTTGGTTGGATTTATGTGGTTGGTTCCTGATTTGGTGGTAGATACGGAGGGTTATCTAGAATCTGATCCTTTGGTGACTCCGGTGTCTATAAAGCCTGAGTGATATTTTTTGATTTATTATGCTATGCTTCGTTCTGTTGAGTCTAAGATAGGTGGTCTAGTGTTGGTGGCTAGATTATTATTTTTTATGTGGGTCCCAACTTTTAAAGACTCTAGTTCATATTTTGTTATTCGACAGGTGATTTTTTGGAGTTTCGTTTGTCTTTTTGTTGGGTTGACTTATTTAGGCTCATGTCACCCTGAGTACCCTTATTTGGGTATTTGTCAGTTATTTTCGGTTGGTGCTGTAGCTTTTATGTTTATTTATAAGCTATTTTGATCGAGTTATACTAAGTTGGGTTTTAGTATTTTTTTAGGATAAATGGCTAAGTTTAGTTTTTTTAGTTGGCTTTTTACGCTAGATCATAAGCGTGTGGGTATATTTATACTTTAATTGGGATATGATCGGGTTTTGTTGGGTTGAGTTTTAGGGTAATGATACGTGTTAATTTTGTTGAGCCTTATTTTAATGTAATTTCTTCGGACTGTTATAAATTTTTGATAACTAATCATGGTATTATTATGATTTTTTTTTTTTTGATGCCCGGGTTGATGGGGGGTTTTGGAAATTATTTGATTCCTTTATTACCCGGTTTACCAAATTTAAATTTACCCCGGTTGAAACCCTTGAAAGCAGGGTTGCTTTTTCCTTCTATTTTATTTCTGGGGTTGAGTATGGGTTGGGGGGCGGGAAAAGGGGGAACTTTTTACCCCCCCCTCTCTTCTTCCCTTTTTAGCAATAGCCGGGGGGCCAATCTTTTGAGGTTTTCTTTACATTTGGCGGGCCTTTCAAGTTTGTTGGGTTCAATAAATTTTATATGTACTCTTTATTCGGCTTTTGTTGATAATTTTGTGTCTCGAAGTTCTATTTTGTTGTGGTCTTATTTATTTACTTCAATCCTTTTATTGTTAACTATTCCTGTGTGGGCTGCTGCTATTACAATGTTGTTGTTTGATCGTAAATTTGGTTCGGCTTTTTTTGATCCTTTGGGTGGGGGTGATCCCGTTTTGTTCCAACATATGTTTTGGTTTTTTGGGCATCCAGAAGTTTATGTATTGATTTTGCCTGGTTTTGGTATGGTAAGTCATGTGTGTAGTAAATTAGGTTGTTCATATGATACTTTTGGTTTTTATGGTTTACTATTTGCTATGTTTTCTATAGTGTGTTTAGGTAGCGTGGTTTGGGGTCACCACATGTTTACTGTGGGGTTGGATGTGAAGACTGCTGTTTTCTTTAGTTCTGTGACTATGATTATTGGGGTTCCCACGGGTATAAAGGTGTTTTCTTGGCTTTATATGATTTTAAATAGTCGTGTTTCGTTGCGTGAGCCTGTGTTTTGATGGGTTTTATCTTTTATTGTGTTGTTTACTATGGGTGGTGTTACTGGTATAATTCTTTCTGCTTGTGTGTTGGATAAAATTTTGCATGACACGTGGTTTGTGGTGGCTCATTTTCATTATGTTATGTCTTTGGGTTCTTATATTAGGGTTATTATATTTTTTGTTTGGTGATGGCCTGTTATCACAGGGGTTAGCTTGAATAAGTATTTGTTACAGTGTCATTGTATAGTATCAAATGTGGGCTTTAATTTGTGTTTTTTTCCTATGCATTATTTTGGTATTTGTGGTTTACCTCGGCGTGTTTGTGTGTATGAGTCAGGGTACGCTTGAGTTAATATGCTTTGTTCAATAGGTTCTTTTGTTTCTGCCTTTAGTGGTTGCTTTTTTATTTTTATTTTATGGGAGTCTTTAGCTAAAAAGAATGTTGTTATAGGTTATTATGGTAGTTCTTCAACTTTGCTTAATTTGTGTTGATCGCCAGTGCCTTACCACAGTAATTTTTTTGTGCGCGGATTATTTGTTGATTATTCTGTATTGGCTTTTTAG

>Hap_7

ATGGTTTTGTTGTTGCGTCGTAATGTGGTTGATTTGCCTACTAATTATTCTCTTAATTATTATTGATGTAGTGGTTTTATGATTTCGGCTTTTATGGTAGTTCAGGTAATCACTGGTGTGATTCTTTCACTTTTGTATGTGGCTGATTCAAGATTAAGTTTTCGTTGTGTTATGGATTTGAGAAAAGATTCTTTTTTTACTTGGGGAGTGCGCTATTGACACATTTGGGGGGTTAGTATTTTGTTTGTCCTGTTTTTTGTTCATATGGGACGTGCCTTATATTATTCAAGTTATACTAAGAAGGGGGTATGGAAAGTGGGGTTTATTTTATATCTTTTAACTATGGCCGAGGCTTTTTTGGGTTATATTTTACCTTGACATCAAATGTCATATTGGGCTGCTACTGTTTTGACGGCTATTGCCGAGAGTATTCCCTTAGTCGGTCCTACGGTGTTTAAGTATTTGGTGGGGGGTTTTTCTGTAACCAAAGTAACTTTGGTTCGTGTATTTTCAGCTCATGTTTGTTTAGGTTTTGTAATTTTAGGTTTAATGATTTTGCATCTTTTTTATTTGCATTCTTCTGGGTCCAATAACCCTTTATTTTCTTCTTTTGGGTATGGGGATGTTGTTTATTTCCACTCTTATTTCACCACTAAGGATTTTTTTTGTTTGGTCGTTCTATGCTGTATTTTGGTTGGATTTATGTGGTTGGTTCCTGATTTGGTGGTAGATACGGAGGGTTATCTAGAATCTGATCCTCTGGTGACCCCGGTGTCTATAAAGCCTGAGTGATATTTTTTGATTTATTATGCTATGCTTCGTTCTGTTGAGTCTAAGATAGGTGGTCTAGTGTTGGTGGCTAGATTATTATTTTTTATGTGGGTCCCAACTTTTAAAGACTCTAGTTCATATTTTGTTATTCGACAGGTGATTTTTTGGAGTTTCGTTTGTCTTTTTGTTGGGTTGACTTATTTAGGCTCATGTCACCCTGAGTACCCTTATTTGGGTATTTGTCAGTTATTTTCGGTTGGTGCTGTAGCTTTTATGTTTATTTATAAGCTATTTTGATCGAGTTATACTAAGTTGGGTTTTAGTATTTTTTTAGGATAAATGGCTAAGTTTAGTTTTTTTAGTTGGCTTTTTACGCTAGATCATAAGCGTGTGGGTATGATTTATACTTTAATTGGGATATGATCGGGTTTTGTTGGGTTGAGTTTTAGGGTAATGATACGTGTTAATTTTGTTGAGCCTTATTTTAATGTAATTTCTTCGGACTGTTATAAATTTTTGATAACTAATCATGGTATTATTATGATTTTTTTTTTTTTGATGCCCGGGTTGATGGGGGGTTTGGGAAATTATTTGATTCCTTTATTACCCGGTTTACCAAATTTAAATTTACCCCGGTTGAAACCCTTGAAAGCAGGGTGGCTTTTTCCTTCAATTTTATTTCTGGGGTTGAGTATGGGTTGGGGGGCGGGAAAAGGGGGGACTTTTTACCCCCCCCTCTCTTCTTCCCTTTTTAGCAATAGCCGGGGGGCCAATCTTTTGAGGTTTTCTTTACATTTGGCGGGTCTTTCAAGTTTGTTGGGTTCTATAAATTTTATATGTACTCTTTATTCGGCTTTTGTTGATAATTTTGTGTCTCGAAGTTCTATTTTGTTGTGATCTTATTTATTTACTCCAATCCTTTTATGGTTAACTATTCCTGTGTGGGCTGCTGCTATTACAATGTTGTTGTTTGATCGTAAATTTGGTTCGGCTTTTTTTGATCCTTTGGGTGGGGGTGATCCCGTTTTGTTTCAACATATGTTTTGGTTTTTTGGGCATCCGGAAGTTTATGTATTGATTTTGCCTGGTTTTGGTATGGTAAGTCATGTGTGTAGTAAATTAGGTTGTTCATATGATACTTTTGGTTTTTATGGTTTACTATTTGCTATGTTTTCTATAGTGTGTTTAGGTAGCGTGGTTTGGGGCCACCACATGTTTACTGTGGGGTTGGATGTGAAGACTGCTGTTTTCTTTAGTTCTGTGACCATGATTATTGGGGTTCCCACGGGTATAAAGGTGTTTTCTTGGCTTTATATGATTTTAAATAGTCGTGTTTCGTTGCGTGAGCCTGTGTTTTGATGGGTTTTATCTTTTATTGTGTTGTTTACTATGGGTGGTGTTACTGGTATAATTCTTTCTGCTTGTGTGTTGGATAAAATTTTGCATGACACGTGGTTTGTGGTGGCTCATTTTCATTATGTTATGTCTTTGGGTTCTTATATTAGGATTATTATATTTTTTGTTTGGTGATGGCCTGTTATCACAGGGGTTAGCCTGAATAAGTATTTGTTACAGTGTCATTGTATAGTATCAAATGTGGGTTTTAATTTGTGTTTTTTCCCTATGCATTATTTTGGTATTTGTGGTTTACCTCGGCGTGTTTGTGTGTATGAGTCAGGGTACGCTTGAGTTAATATGCTTTGTTCAATAGGTTCTTTTGTTTCTGCCTTTAGTGGTTGCTTTTTTATTTTTATTTTATGGGAGTCTTTAGCTAAAAAGAATGTTGTTATAGGTTATTATGGTAGTTCTTCAACTTTGCTTAATCTGTGTTGGTCGCCAGTGCCTTATCACAGTAATTTTTTTGTGCGCGGATTATTTGTTGATTATTCTGTATTGGCTTTTTAG

>Hap_8

ATGGTTTTGTTGTTGCGTCGTAATGTGGTTGATTTGCCTACTAATTATTCTCTTAATTATTATTGATGTAGTGGTTTTATGATTTCGGCTTTTATGGTAGTTCAGGTAATCACTGGTGTGATTCTTTCACTTTTGTATGTGGCTGATTCAAGATTAAGTTTTCGTTGTGTTATGGATTTGAGAAAAGATTCTTTTTTTACTTGGGGAGTGCGCTATTGACACATTTGGGGGGTTAGTATTTTGTTTGTCCTGTTTTTTGTTCATATGGGACGTGCCTTATATTATTCAAGTTATACTAAGAAGGGGGTATGGAAAGTGGGGTTTATTTTATATCTTTTAACTATGGCCGAGGCTTTTTTGGGTTATATTTTACCTTGACATCAAATGTCATATTGGGCTGCTACTGTTTTGACGGCTATTGCCGAGAGTATTCCCTTAGTCGGTCCTACGGTGTTTAAGTATTTGGTGGGGGGTTTTTCTGTAACCAAAGTAACTTTGGTTCGTGTATTTTCAGCTCATGTTTGTTTAGGTTTTGTAATTTTAGGTTTAATGATTTTGCATCTTTTTTATTTGCATTCTTCTGGGTCCAATAACCCTTTATTTTCTTCTTTTGGGTATGGGGATGTTGTTTATTTCCACTCTTATTTCACCACTAAGGATTTTTTTTGTTTGGTCGTTCTATGCTGTATTTTGGTTGGATTTATGTGGTTGGTTCCTGATTTGGTGGTAGATACGGAGGGTTATCTAGAATCTGATCCTCTGGTGACTCCGGTGTCTATAAAGCCTGAGTGATATTTTTTGATTTATTATGCTATGCTTCGTTCTGTTGAGTCTAAGATAGGTGGTCTAGTGTTGGTGGCTAGATTATTATTTTTTATGTGGGTCCCAACTTTTAAAGACTCTAGTTCATATTTTGTTATTCGACAGGTGATTTTTTGGAGTTTCGTTTGTCTTTTTGTTGGGTTGACTTATTTAGGCTCATGTCACCCTGAGTACCCTTATTTGGGAATTTGTCAGTTATTTTCGGTTGGTGCTGTAGCTTTTATGTTTATTTATAAGCTATTTTGATCGAGTTATACTAAGTTGGGTTTTAGTATTTTTTTAGGATAAATGGCTAAGTTTAGTTTTTTTAGTTGGCTTTTTACGCTAGATCATAAGCGTGTGGGTATGATTTATACTTTAATTGGGATATGATCGGGTTTTGTTGGGTTGAGTTTTAGGGTAATGATACGTGTTAATTTTGTTGAGCCTTATTTTAATGTAATTTCTTCGGACTGTTATAAATTTTTGATAACTAATCATGGTATTATTATGATTTTTTTTTTTTTGAGGCCCGGGTTGATGGGGGGTTTGGGAAATTATTTAATCCCTTTTTTACCGGGTTTACCAAATTTAATTTTCCCCCGGTTGAAACCCTTGAAAGCAGGGTGGCTTTTCCCTTCAATTTTATTTCTGGGGTGAAGAATGGGTTGGGGGGCGGGAAAAGGGGGAACTTTTTACCCCCCCCTCTCTTCTTCCCTTTTTAGCAATAGCCGGGGGGCCAATCTTTTAAGGTTTTCTTTACATTTGGCGGGTCTTTCAAGTTTGTTGGGTTCTATAAATTTTATATGTACTCTTTATTCGGCTTTTGTTGATAATTTTGTGTCTCGAAGTTCTATTTTGTTGTGATCTTATTTATTTACTTCAATCCTTTTATGGTTAACTATTCCTGTGTGGGCTGCTGCTATTACAATGTTGTTGTTTGATCGTAAATTTGGTTCGGCTTTTTTTGATCCTTTGGGTGGGGGTGATCCCGTTTTGTTTCAACATATGTTTTGGTTTTTTGGGCATCCGGAAGTTTATGTATTGATTTTGCCTGGTTTTGGTATGGTAAGTCATGTGTGTAGTAAATTAGGTTGTTCATATGATACTTTTGGTTTTTATGGTTTACTATTTGCTATGTTTTCTATAGTGTGTTTAGGTAGCGTGGTTTGGGGCCACCACATGTTTACTGTGGGGTTGGATGTGAAGACTGCTGTTTTCTTTAGTTCTGTGACCATGATTATTGGGGTTCCCACGGGTATAAAGGTGTTTTCTTGGCTTTATATGATTTTAAATAGTCGTGTTTCGTTGCGTGAGCCTGTGTTTTGATGGGTTTTATCTTTTATTGTGTTGTTTACTATGGGTGGTGTTACTGGTATAATTCTTTCTGCTTGTGTGTTGGATAAAATTTTGCATGACACGTGGTTTGTGGTGGCTCATTTTCATTATGTTATGTCTTTGGGTTCTTATATTAGGATTATTATATTTTTTGTTTGGTGATGGCCTGTTATCACAGGGGTTAGCCTGAATAAGTATTTGTTACAGTGTCATTGTATAGTATCAAATGTGGGTTTTAATTTGTGTTTTTTCCCTATGCATTATTTTGGTATTTGTGGTTTACCTCGGCGTGTTTGTGTGTATGAGTCAGGGTACGCTTGAGTTAATATGCTTTGTTCAATAGGTTCTTTTGTTTCTGCCTTTAGTGGTTGCTTTTTTATT

TTTATTTTATGGGAGTCTTTAGCTAAAAAGAATGTTGTTATAGGTTATTATGGTAGTTCTTCAACTTTGCTTAATCTGTGTTGGTCGCCAGTGCCTTATCACAGTAATTTTTTTGTGCGCGGATTATTTGTTGATTATTCTGTATTGGCTTTTTAG

>Hap_9

ATGGTTTTGTTGTTGCGTCGTAATGTGGTTGATTTGCCTACTAATTATTCTCTTAATTATTATTGATGTAGTGGTTTTATGATTTCGGCTTTTATGGTAGTTCAGGTAATCACTGGTGTGATTCTTTCACTTTTGTATGTGGCTGATTCAAGATTAAGTTTTCGTTGTGTTATGGATTTGAGAAAAGATTCTTTTTTTACTTGGGGAGTGCGCTATTGACACATTTGGGGGGTTAGTATTTTGTTTGTCCTGTTTTTTGTTCATATGGGACGTGCCTTATATTATTCAAGTTATACTAAGAAGGGGGTATGGAAAGTGGGGTTTATTTTATATCTTTTAACTATGGCCGAGGCTTTTTTGGGTTATATTTTACCTTGACATCAAATGTCATATTGGGCTGCTACTGTTTTGACGGCTATTGCCGAGAGTATTCCCTTAGTCGGTCCTACGGTGTTTAAGTATTTGGTGGGGGGTTTTTCTGTAACCAAAGTAACTTTGGTTCGTGTATTTTCAGCTCATGTTTGTTTAGGTTTTGTAATTTTAGGTTTAATGATTTTGCATCTTTTTTATTTGCATTCTTCTGGGTCCAATAACCCTTTATTTTCTTCTTTTGGGTATGGGGATGTTGTTTATTTCCACTCTTATTTCACCACTAAGGATTTTTTTTGTTTGGTCGTTCTATGCTGTATTTTGGTTGGATTTATGTGGTTGGTTCCTGATTTGGTGGTAGATACGGAGGGTTATCTAGAATCTGATCCTCTGGTGACCCCGGTGTCTATAAAGCCTGAGTGATATTTTTTGATTTATTATGCTATGCTTCGTTCTGTTGAGTCTAAGATAGGTGGTCTAGTGTTGGTGGCTAGATTATTATTTTTTATGTGGGTCCCAACTTTTAAAGACTCTAGTTCATATTTTGTTATTCGACAGGTGATTTTTTGGAGTTTCGTTTGTCTTTTTGTTGGGTTGACTTATTTAGGCTCATGTCACCCTGAGTACCCTTATTTGGGTATTTGTCAGTTATTTTCGGTTGGTGCTGTAGCTTTTATGTTTATTTATAAGCTATTTTGATCGAGTTATACTAAGTTGGGTTTTAGTATTTTTTTAGGATAAATGGCTAAGTTTAGTTTTTTTAGTTGGCTTTTTACGCTAGATCATAAGCGTGTGGGTATGATTTATACTTTAATTGGGATATGATCGGGTTTTGTTGGGTTGAGTTTTAGGGTAATGATACGTGTTAATTTTGTTGAGCCTTATTTTAATGTAATTTCTTCGGACTGTTATAAATTTTTGATAACTAATCATGGTATTATTATGATTTTTTTTTTTTTGATGCCCGGGTTGATGGGGGGTTTGGGAAATTATTTGATCCCTTTATTACCCGGTTTACCAAATTTAAATTTACCCCGGTTGAAACCCTTGAAAGCAGGGTGGCTTTTTCCTTCAATTTTATTTCTGGGGTTGAGTATGGGTTGGGGGGCGGGAAAAGGGGGGACTTTTTACCCCCCCCTCTCTTCTTCCCTTTTTAGCAATAGCCGGGGGGCCAATCTTTTGAGGTTTTCTTTACATTTGGCGGGTCTTTCAAGTTTGTTGGGTTCTATAAATTTTATATGTACTCTTTATTCGGCTTTTGTTGATAATTTTGTGTCTCGAAGTTCTATTTTGTTGTGATCTTATTTATTTACTCCAATCCTTTTATGGTTAACTATTCCTGTGTGGGCTGCTGCTATTACAATGTTGTTGTTTGATCGTAAATTTGGTTCGGCTTTTTTTGATCCTTTGGGTGGGGGTGATCCCGTTTTGTTTCAACATATGTTTTGGTTTTTTGGGCATCCGGAAGTTTATGTATTGATTTTGCCTGGTTTTGGTATGGTAAGTCATGTGTGTAGTAAATTAGGTTGTTCATATGATACTTTTGGTTTTTATGGTTTACTATTTGCTATGTTTTCTATAGTGTGTTTAGGTAGCGTGGTTTGGGGCCACCACATGTTTACTGTGGGGTTGGATGTGAAGACTGCTGTTTTCTTTAGTTCTGTGACCATGATTATTGGGGTTCCCACGGGTATAAAGGTGTTTTCTTGGCTTTATATGATTTTAAATAGTCGTGTTTCGTTGCGTGAGCCTGTGTTTTGATGGGTTTTATCTTTTATTGTGTTGTTTACTATGGGTGGTGTTACTGGTATAATTCTTTCTGCTTGTGTGTTGGATAAAATTTTGCATGACACGTGGTTTGTGGTGGCTCATTTTCATTATGTTATGTCTTTGGGTTCTTATATTAGGATTATTATATTTTTTGTTTGGTGATGGCCTGTTATCACAGGGGTTAGCCTGAATAAGTATTTGTTACAGTGTCATTGTATAGTATCAAATGTGGGTTTTAATTTGTGTTTTTTCCCTATGCATTATTTTGGTATTTGTGGTTTACCTCGGCGTGTTTGTGTGTATGAGTCAGGGTACGCTTGAGTTAATATGCTTTGTTCAATAGGTTCTTTTGTTTCTGCCTTTAGTGGTTGCTTTTTTATTTTTATTTTATGGGAGTCTTTAGCTAAAAAGAATGTTGTTATAGGTTATTATGGTAGTTCTTCAACTTTGCTTAATCTGTGTTGGTCGCCAGTGCCTTATCACAGTAATTTTTTTGTGCGCGGATTATTTGTTGATTATTCTGTATTGGCTTTTTAG

>Hap_10

ATGGTTTTGTTGTTGCGTCGTAATGTGGTTGATTTGCCTACTAATTATTCTCTTAATTATTATTGATGTAGTGGTTTTATGATTTCGGCTTTTATGGTAGTTCAGGTAATCACTGGTGTGGTTCTTTCACTTTTGTATGTGGCTGATTCAAGATTAAGTTTTCGTTGTGTTATGGATTTGAGAAAAGATTCTTTTTTTACTTGGGGAGTGCGCTATTGACACATTTGGGGGGTTAGTATTTTGTTTGTCCTGTTTTTTGTTCATATGGGACGTGCCTTATATTATTCAAGTTATACCAAGAAGGGGGTATGGAAAGTGGGGTTTATTTTATATCTTTTAACTATGGCCGAGGCTTTTTTGGGTTATATTCTACCTTGACATCAAATGTCATATTGGGCTGCTACTGTTTTGACGGCTATTGCCGAGAGTATTCCCTTAGTTGGTCCTACGGTGTTTAAGTATTTGGTGGGGGGTTTTTCTGTAACTAAAGTAACTTTGGTTCGTGTATTTTCAGCTCATGTTTGTTTAGGTTTTGTAATTTTAGGTTTAATGATTTTGCATCTTTTTTATTTGCATTCTTCTGGGTCCAATAACCCTTTATTTTCTTCTTTTGGGTATGGGGATGTTGTTTATTTTCACTCTTATTTCACCACTAAGGATTTTTTTTGTTTGGTTGTTCTATGCTGTATTTTGGTTGGGTTTATGTGGTTGGTTCCTGATTTGGTGGTAGATACGGAGGGTTATCTAGAATCTGATCCTTTGGTGACTCCGGTGTCTATAAAGCCTGAGTGATATTTTTTGATTTATTATGCTATGCTTCGTTCTGTTGAGTCTAAGATAGGTGGTCTAGTGTTGGTGGCTAGATTATTATTTTTTATGTGGGTCCCAACTTTTAAAGACTCTAGTTCATATTTTGTTATTCGACAGGTGATTTTTTGGAGTTTCGTTTGTCTTTTTGTTGGGTTGACTTATTTAGGCTCATGTCACCCTGAGTACCCTTATTTGGGTATTTGTCAGTTATTTTCGGTTGGTGCTGTAGCTTTTATGTTTATTTATAAGCTATTTTGATCGAGTTATACTAAGTTGGGTTTTAGTATTTTTTTAGGATAAATGGCTAAGTTTAGTTTTTTTAGTTGGCTTTTTACGCTAGATCATAAGCGTGTGGGTATGATTTATACTTTAATTGGGATATGATCGGGTTTTGTTGGGTTGAGTTTTAGGGTAATGATACGTGTTAATTTTGTTGAGCCTTATTTTAATGTAATTTCTTCGGACTGTTATAAATTTTTGATAACTAATCATGGTATTATTATGATTTTTTTTTTTTTGATGCCCGGGTTGATGGGGGGGTTGGGAAATTATTTGATTCCTTTATTATCCGGTTTACCAAATTTAAATTTACCCCGGTTGAAACCCTTGAAAGCAGGGTGGCTTTTTCCTTCTATTTTATTTCTGGGGTTGAGTATGGGTTGGGGGGCGGGAAAAGGGGGAACTTTTTACCCCCCCCTCTCTTCTTCCCTTTTTAGCAATAGCCGGGGGGCCAATCTTTTGAGGTTTTCTTTACTTTTGGCGGGCCTTTCAAGTTTGTTGGGTTCAATAAATTTTATATGTACTCTTTATTCGGCTTTTGTTGATAATTTTGTGTCTCGAAGTTCTATTTTGTTGTGATCTTATTTATTTACTTCAATCCTTTTATGGTTAACTATTCCTGTGTGGGCTGCTGCTATTACAATGTTGTTGTTTGATCGTAAATTTGGTTCGGCTTTTTTTGATCCTTTGGGTGGGGGTGATCCTGTTTTGTTTCAACATATGTTTTGATTTTTTGGGCATCCGGAAGTTTATGTATTGATTTTGCCCGGTTTTGGTATGGTAAGTCATGTGTGTAGTAAATTAGGTTGTTCATATGATACTTTTGGTTTTTATGGTTTACTGTTTGCTATGTTTTCTATAGTGTGTTTAGGTAGTGTGGTTTGGGGCCACCACATGTTTACTGTGGGGTTGGATGTGAAGACTGCTGTTTTCTTTAGTTCTGTGACCATGATTATTGGGGTTCCCACGGGTATAAAGGTGTTTTCTTGGCTTTATATGATTTTAAATAGTCGTGTTTCGTTGCGTGAGCCTGTGTTTTGATGGGTTTTATCTTTTATTGTGTTGTTTACTATGGGTGGTGTTACTGGTATAATTCTTTCTGCTTGTGTGTTGGATAAAATTTTGCATGACACGTGGTTTGTGGTGGCTCATTTTCATTATGTTATGTCTTTGGGTTCTTATATTAGGGTTATTATATTTTTTGTTTGGTGATGGCCTGTTATTACAGGGGTTAGCCTGAATAAGTATTTGTTACAGTGTCATTGTATAGTATCAAATGTGGGTTTTAATTTGTGTTTTTTCCCTATGCATTATTTTGGTATTTGTGGTTTACCTCGGCGTGTTTGTGTGTATGAGTCAGGGTACGCTTGAGTTAATATGCTTTGTTCAATAGGTTCTTTTGTTTCTGCCTTTAGTGGTTGCTTTTTTATTTTTATTTTATGGGAGTCTTTAGCTAAAAAGAATGTTGTTATAGGTTATTATGGTAATTCTTCAACTTTGCTTAATCTGTGTTGATCGCCAGTGCCTTATCACAGTAATTTTTTTGTGCGCGGATTATTTGTTGATTATTCTGTATTGGCTTTTTAG

>Hap_11

ATGGTTTTGTTGTTGCGTCGTAATGTGGTTGATTTGCCTACTAATTATTCTCTTAATTATTATTGATGTAGTGGTTTTATGATTTCGGCTTTTATGGTAGTTCAGGTAATCACTGGTGTGATTCTTTCACTTTTGTATGTGGCTGATTCAAGATTAAGTTTTCGTTGTGTTATGGATTTGAGAAAAGATTCTTTTTTTACTTGGGGAGTGCGCTATTGACACATTTGGGGGGTTAGTATTTTGTTTGTCCTGTTTTTTGTTCATATGGGACGTGCCTTATATTATTCAAGTTATACTAAGAAGGGGGTATGGAAAGTGGGGTTTATTTTATATCTTTTAACTATGGCCGAGGCTTTTTTGGGTTATATTTTACCTTGACATCAAATGTCATATTGGGCTGCTACTGTTTTGACGGCTATTGCCGAGAGTATTCCCTTAGTCGGTCCTACGGTGTTTAAGTATTTGGTGGGGGGTTTTTCTGTAACCAAAGTAACTTTGGTTCGTGTATTTTCAGCTCATGTTTGTTTAGGTTTTGTAATTTTAGGTTTAATGATTTTGCATCTTTTTTATTTGCATTCTTCTGGGTCCAATAACCCTTTATTTTCTTCTTTTGGGTATGGGGATGTTGTTTATTTCCACTCTTATTTCACCACTAAGGATTTTTTTTGTTTGGTCGTTCTATGCTGTATTTTGGTTGGATTTATGTGGTTGGTTCCTGATTTGGTGGTAGATACGGAGGGTTATCTAGAATCTGATCCTCTGGTGACTCCGGTGTCTATAAAGCCTGAGTGATATTTTTTGATTTATTATGCTATGCTTCGTTCTGTTGAGTCTAAGATAGGTGGTCTAGTGTTGGTGGCTAGATTATTATTTTTTATGTGGGTCCCAACTTTTAAAGACTCTAGTTCATATTTTGTTATTCGACAGGTGATTTTTTGGAGTTTCGTTTGTCTTTTTGTTGGGTTGACTTATTTAGGCTCATGTCACCCTGAGTACCCTTATTTGGGTATTTGTCAGTTATTTTCGGTTGGTGCTGTAGCTTTTATGTTTATTTATAAGCTATTTTGATCGAGTTATACTAAGTTGGGTTTTAGTATTTTTTTAGGATAAATGGCTAAGTTTAGTTTTTTTAGTTGGCTTTTTACGCTAGATCATAAGCGTGTGGGTATGATTTATACTTTAATTGGGATATGATCGGGTTTTGTTGGGTTGAGTTTTAGGGTAATGATACGTGTTAATTTTGTTGAGCCTTATTTTAATGTAATTTCTTCGGACTGTTATAAATTTTTGATAACTAATCATGGTATTATTATGATTTTTTTTTTTTTGATGCCCGGGTTGATGGGGGGTTTTGGAAATTATTTGATTCCTTTATTACCGGGTTTACCAAATTTAAATTTACCCCGGTTGAAACCCTTGAAAGCAGGGTTGCTTTTTCCTTCTATTTTATTTCTGGGGTTGAGTATGGGTTGGGGGGCGGGAAAAGGGGGAACTTTTTACCCCCCCCTCTCTTCTTCCCTTTTTAGCAATAGCCGGGGGGCCAATCTTTTGAGGTTTTCTTTACATTTGGCGGGCCTTTCAAGTTTGTTGGGTTCAATAAATTTTATATGTACTCTTTATTCGGCTTTTGTTGATAATTTTGTGTCTCGAAGTTCTATTTTGTTGTGGTCTTATTTATTTACTTCTATCCTTTTATGGTTAACTATTCCTGTGTGGGCTGCTGCTATTACAATGTTGTTGTTTGATCGTAAATTTGGTTCGGCTTTTTTTGATCCTTTGGGTGGGGGTGATCCCGTTTTGTTCCAACATATGTTTTGGTTTTTTGGGCATCCGGAAGTTTATGTATTGATTTTGCCTGGTTTTGGTATGGTAAGTCATGTGTGTAGTAAATTAGGTTGTTCATATGATACTTTTGGTTTTTATGGTTTACTGTTTGCTATGTTTTCTATAGTGTGTTTAGGTAGCGTGGTTTGGGGCCACCACATGTTTACTGTGGGGTTGGATGTGAAGACTGCTGTTTTCTTTAGTTCTGTGACCATGATTATTGGGGTTCCCACGGGTATAAAGGTGTTTTCTTGGCTTTATATGATTTTAAATAGTCGTGTTTCGTTGCGTGAGCCTGTGTTTTGATGGGTTTTATCTTTTATTGTGTTGTTTACTATGGGTGGTGTTACTGGTATAATTCTTTCTGCTTGTGTGTTGGATAAAATTTTGCATGACACGTGGTTTGTGGTGGCTCATTTTCATTATGTTATGTCTTTGGGTTCTTATATTAGGATTATTATATTTTTTGTTTGGTGATGGCCTGTTATCACAGGGGTTAGCCTGAATAAGTATTTGTTACAGTGTCATTGTATAGTATCAAATGTGGGTTTTAATTTGTGTTTTTTCCCTATGCATTATTTTGGTATTTGTGGTTTACCTCGGCGTGTTTGTGTGTATGAGTCAGGGTACGCTTGAGTTAATATGCTTTGTTCAATAGGTTCTTTTGTTTCTGCCTTTAGTGGTTGCTTTTTTATT

TTTATTTTATGGGAGTCTTTAGCTAAAAAGAATGTTGTTATAGGTTATTATGGTAGTTCTTCAACTTTGCTTAATCTGTGTTGGTCGCCAGTGCCTTATCACAGTAATTTTTTTGTGCGCGGATTATTTGTTGATTATTCTGTATTGGCTTTTTAG

>Hap_12

ATGGTTTTGTTGTTGCGTCGTAATGTGGTTGATTTGCCTACTAATTATTCTCTTAATTATTATTGATGTAGTGGTTTTATGATTTCGGCTTTTATGGTAGTTCAGGTAATCACTGGTGTGATTCTTTCACTTTTGTATGTGGCTGATTCAAGATTAAGTTTTCGTTGTGTTATGGATTTGAGAAAAGATTCTTTTTTTACTTGGGGAGTGCGCTATTGACACATTTGGGGGGTTAGTATTTTGTTTGTCCTGTTTTTTGTTCATATGGGACGTGCCTTATATTATTCAAGTTATACTAAGAAGGGGGTATGGAAAGTGGGGTTTATTTTATATCTTTTAACTATGGCCGAGGCTTTTTTGGGTTATATTTTACCTTGACATCAAATGTCATATTGGGCTGCTACTGTTTTGACGGCTATTGCCGAGAGTATTCCCTTAGTCGGTCCTACGGTGTTTAAGTATTTGGTGGGGGGGTTTTCTGTAACCAAAGTAACTTTGGTTCGTGTATTTTCAGCTCATGTTTGTTTAGGTTTTGTAATTTTAGGTTTAATGATTTTGCATCTTTTTTATTTGCATTCTTCTGGGTCCAATAACCCTTTATTTTCTTCTTTTGGGTATGGGGATGTTGTTTATTTCCACTCTTATTTCACCACTAAGGATTTTTTTTGTTTGGTCGTTCTATGCTGTATTTTGGTTGGATTTATGTGGTTGGTTCCTGATTTGGTGGTAGATACGGAGGGTTATCTAGAATCTGATCCTCTGGTGACTCCGGTGTCTATAAAGCCTGAGTGATATTTTTTGATTTATTATGCTATGCTTCGTTCTGTTGAGTCTAAGATAGGTGGTCTAGTGTTGGTGGCTAGATTATTATTTTTTATGTGGGTCCCAACTTTTAAAGACTCTAGTTCATATTTTGTTATTCGACAGGTGATTTTTTGGAGTTTCGTTTGTCTTTTTGTTGGGTTGACTTATTTAGGCTCATGTCACCCTGAGTACCCTTATTTGGGTATTTGTCAGTTATTTTCGGTTGGTGCTGTAGCTTTTATGTTTATTTATAAGCTATTTTGATCGAGTTATACTAAGTTGGGTTTTAGTATTTTTTTAGGATAAATGGCTAAGTTTAGTTTTTTTAGTTGGCTTTTTACGCTAGATCATAAGCGTGTGGGTATGATTTATACTTTAATTGGGATATGATCGGGTTTTGTTGGGTTGAGTTTTAGGGTAATGATACGTGTTAATTTTGTTGAGCCTTATTTTAATGTAATTTCTTCGGACTGTTATAAATTTTTGATAACTAATCATGGTATTATTATGATTTTTTTTTTTTTGATGCCCGGGTTGATGGGGGGTTTGGGAAATTATTTAATCCCTTTATTACCCGGTTTACCAAATTTAAATTTACCTCGGTTGAAACCCTTGAAAGCAGGGTGGCTTTTTCCTCCAATTTTATTTCTGGGGTGAAGAAGGGGTTGGGGGGCGGGAAAAGGGGGGACTTTTTACCCCCCCCTCTCTTCTTCCCTTTTTAGCAATAGCCGGGGGGCCAACCTTTTGAGGTTTTCTTTACATTTGGCGGGTCTTTCAAGTTTGTTGGGTTCTATAAATTTTATATGTACTCTTTATTCGGCTTTTGTTGATAATTTTGTGTCTCGAAGTTCTATTTTGTTGTGATCTTATTTATTTACTCTAATCCTTTTATGGTTAACTATTCCTGTGTGGGCTGCTGCTATTACAATGTTGTTGTTTGATCGTAAATTTGGTTCGGCTTTTTTTGATCCTTTGGGTGGGGGTGATCCCGTTTTGTTTCAACATATGTTTTGGTTTTTTGGGCATCCGGAAGTTTATGTATTGATTTTGCCTGGTTTTGGTATGGTAAGTCATGTGTGTAGTAAATTAGGTTGTTCATATGATACTTTTGGTTTTTATGGTTTACTATTTGCTATGTTTTCTATAGTGTGTTTAGGTAGCGTGGTTTGGGGCCACCACATGTTTACTGTGGGGTTGGATGTGAAGACTGCTGTTTTCTTTAGTTCTGTGACCATGATTATTGGGGTTCCCACGGGTATAAAGGTGTTTTCTTGGCTTTATATGATTTTAAATAGTCGTGTTTCGTTGCGTGAGCCTGTGTTTTGATGGGTTTTATCTTTTATTGTGTTGTTTACTATGGGTGGTGTTACTGGTATAATTCTTTCTGCTTGTGTGTTGGATAAAATTTTGCATGACACGTGGTTTGTGGTGGCTCATTTTCATTATGTTATGTCTTTGGGTTCTTATATTAGGATTATTATATTTTTTGTTTGGTGATGGCCTGTTATCACAGGGGTTAGCCTGAATAAGTATTTGTTACAGTGTCATTGTATAGTATCAAATGTGGGTTTTAATTTGTGTTTTTTCCCTATGCATTATTTTGGTATTTGTGGTTTACCTCGGCGTGTTTGTGTGTATGAGTCAGGGTACGCTTGAGTTAATATGCTTTGTTCAATAGGTTCTTTTGTTTCTGCCTTTAGTGGTTGCTTTTTTATTTTTATTTTATGGGAGTCTTTAGCTAAAAAGAATGTTGTTATAGGTTATTATGGTAGTTCTTCAACTTTGCTTAATCTGTGTTGGTCGCCAGTGCCTTATCACAGTAATTTTTTTGTGCGCGGATTATTTGTTGATTATTCTGTATTGGCTTTTTAG

>Hap_13

ATGGTTTTGTTGTTGCGTCGTAATGTGGTTGATTTGCCTACTAATTATTCTCTTAATTATTATTGATGTAGTGGTTTTATGATTTCGGCTTTTATGGTAGTTCAGGTAATCACTGGTGTGGTTCTTTCACTTTTGTATGTGGCTGATTCAAGATTAAGTTTTCGTTGTGTTATGGATTTGAGAAAAGATTCTTTTTTTACTTGGGGAGTGCGCTATTGACACATTTGGGGGGTTAGTATTTTGTTTGTCCTGTTTTTTGTTCATATGGGACGTGCCTTATATTATTCAAGTTATACCAAGAAGGGGGTATGGAAAGTGGGGTTTATTTTATATCTTTTAACTATGGCCGAGGCTTTTTTGGGTTATATTCTACCTTGACATCAAATGTCATATTGGGCTGCTACTGTTTTGACGGCTATTGCCGAGAGTATTCCCTTAGTTGGTCCTACGGTGTTTAAGTATTTGGTGGGGGGGTTTTCTGTAACTAAAGTAACTTTGGTTCGTGTATTTTCAGCTCATGTTTGTTTAGGTTTTGTAATTTTAGGTTTAATGATTTTGCATCTTTTTTATTTGCATTCTTCTGGGTCCAATAACCCTTTATTTTCTTCTTTTGGGTATGGGGATGTTGTTTATTTTCACTCTTATTTCACCACTAAGGATTTTTTTTGTTTGGTTGTTCTATGCTGTATTTTGGTTGGGTTTATGTGGTTGGTTCCTGATTTGGTGGTAGATACGGAGGGTTATCTAGAATCTGATCCTTTGGTGACTCCGGTGTCTATAAAGCCTGAGTGATATTTTTTGATTTATTATGCTATGCTTCGTTCTGTTGAGTCTAAGATAGGTGGTCTAGTGTTGGTGGCTAGATTATTATTTTTTATGTGGGTCCCAACTTTTAAAGACTCTAGTTCATATTTTGTTATTCGACAGGTGATTTTTTGGAGTTTCGTTTGTCTTTTTGTTGGGTTGACTTATTTAGGCTCATGTCACCCTGAGTACCCTTATTTGGGTATTTGTCAGTTATTTTCGGTTGGTGCTGTAGCTTTTATGTTTATTTATAAGCTATTTTGATCGAGTTATACTAAGTTGGGTTTTAGTATTTTTTTAGGATAAATGGCTAAGTTTAGTTTTTTTAGTTGGCTTTTTACGCTAGATCATAAGCGTGTGGGTATGATTTATACTTTAATTGGGATATGATCAGGTTTTGTTGGGTTGAGTTTTAGGGTAATGATACGTGTTAATTTTGTTGAGCCTTATTTTAATGTAATTTCTTCGGACTGTTATAAATTTTTGATAACTAATCATGGTATTATTATGATTTTTTTTTTTTTGATGCCCGGGTTGATTGGGGGGTTGGGAAATTATTTGATCCCTTTATTACCCGGTTTACCAAATTTAAATTTCCCCCGGTTGAAACCCTTGAAAGCAGGGTGGCTTTTCCCTTCTATTTTATTTCTGGGGTTGAGAATGGGTTTGGGGGCGGGAAAAGGGGGAACTTTTTACCCCCCCCTCTCTTCTTCCCTTTTTAGCAATAGCCGGGGGGCCAATCTTTTGATGTTTTCTTTACTTTTGGCGGGTCTTTCAAGTTTGTTGGGTTCAATAAATTTTATATGTACTCTTTATTCGGCTTTTGTTGATAATTTTGTGTCTCGAAGTTCTATTTTGTTGTGATCTTATTTATTTACTCCAATCCTTTTATGGTTAACTATTCCTGTGTGGGCTGCTGCTATTACAATGTTGTTGTTTGATCGTAAATTTGGTTCGGCTTTTTTTGATCCTTTGGGTGGGGGTGATCCTGTTTTGTTTCAACATATGTTTTGATTTTTTGGGCATCCGGAAGTTTATGTATTGATTTTGCCCGGTTTTGGTATGGTAAGTCATGTGTGTAGTAAATTAGGTTGTTCATATGATACTTTTGGTTTTTATGGTTTACTGTTTGCTATGTTTTCTATAGTGTGTTTAGGTAGTGTGGTTTGGGGCCACCACATGTTTACTGTGGGGTTGGATGTGAAGACTGCTGTTTTCTTTAGTTCTGTGACCATGATTATTGGGGTTCCCACGGGTATAAAGGTGTTTTCTTGGCTTTATATGATTTTAAATAGTCGTGTTTCGTTGCGTGAGCCTGTGTTTTGATGGGTTTTATCTTTTATTGTGTTGTTTACTATGGGTGGTGTTACTGGTATAATTCTTTCTGCTTGTGTGTTGGATAAAATTTTGCATGACACGTGGTTTGTGGTGGCTCATTTTCATTATGTTATGTCTTTGGGTTCTTATATTAGGGTTATTATATTTTTTGTTTGGTGATGGCCTGTTATTACAGGGGTTAGCCTGAATAAGTATTTGTTACAGTGTCATTGTATAGTATCAAATGTGGGTTTTAATTTGTGTTTTTTCCCTATGCATTATTTTGGTATTTGTGGTTTACCTCGGCGTGTTTGTGTGTATGAGTCAGGGTACGCTTGAGTTAATATGCTTTGTTCAATAGGTTCTTTTGTTTCTGCCTTTAGTGGTTGCTTTTTTATTTTTATTTTATGGGAGTCTTTAGCTAAAAAGAATGTTGTTATAGGTTATTATGGTAATTCTTCAACTTTGCTTAATCTGTGTTGATCGCCAGTGCCTTATCACAGTAATTTTTTTGTGCGCGGATTATTTGTTGATTATTCTGTATTGGCTTTTTAG

>Hap_14

ATGGTTTTGTTGTTGCGTCGTAATGTGGTTGATTTGCCTACTAATTATTCTCTTAATTATTATTGATGTAGTGGTTTTATGATTTCGGCTTTTATGGTAGTTCAGGTAATCACTGGTGTGATTCTTTCACTTTTGTATGTGGCTGATTCAAGATTAAGTTTTCGTTGTGTTATGGATTTGAGAAAAGATTCTTTTTTTACTTGGGGAGTGCGCTATTGACACATTTGGGGGGTTAGTATTTTGTTTGTCCTGTTTTTTGTTCATATGGGACGTGCCTTATATTATTCAAGTTATACTAAGAAGGGGGTATGGAAAGTGGGGTTTATTTTATATCTTTTAACTATGGCCGAGGCTTTTTTGGGTTATATTTTACCTTGACATCAAATGTCATATTGGGCTGCTACTGTTTTGACGGCTATTGCCGAGAGTATTCCCTTAGTCGGTTCTACGGTGTTTAAGTATTTGGTGGGGGGTTTTTCTGTAACCAAAGTAACTTTGGTTCGTGTATTTTCAGCTCATGTTTGTTTAGGTTTTGTAATTTTAGGTTTAATGATTTTGCATCTTTTTTATTTGCATTCTTCTGGGTCCAATAACCCTTTATTTTCTTCTTTTGGGTATGGGGATGTTGTTTATTTCCACTCTTATTTCACCACTAAGGATTTTTTTTGTTTGGTCGTTCTATGCTGTATTTTGGTTGGATTTATGTGGTTGGTTCCTGATTTGGTGGTAGATACGGAGGGTTATCTAGAATCTGATCCTCTGGTGACTCCGGTGTCTATAAAGCCTGAGTGATATTTTTTGATTTATTATGCTATGCTTCGTTCTGTTGAGTCTAAGATAGGTGGTCTAGTGTTGGTGGCTAGATTATTATTTTTTATGTGGGTCCCAACTTTTAAAGACTCTAGTTCATATTTTGTTATTCGACAGGTGATTTTTTGGAGTTTCGTTTGTCTTTTTGTTGGGTTGACTTATTTAGGCTCATGTCACCCTGAGTACCCTTATTTGGGTATTTGTCAGTTATTTTCGGTTGGTGCTGTAGCTTTTATGTTTATTTATAAGCTATTTTGATCGAGTTATACTAAGTTGGGTTTTAGTATTTTTTTAGGATAAATGGCTAAGTTTAGTTTTTTTAGTTGGCTTTTTACGCTAGATCATAAGCGTGTGGGTATGATTTATACTTTAATTGGGATATGATCGGGTTTTGTTGGGTTGAGTTTTAGGGTAATGATACGTGTTAATTTTGTTGAGCCTTATTTTAATGTAATTTCTTCGGACTGTTATAAATTTTTGATAACTAATCATGGTATTATTATGATTTTTTTTTTTTTGATGCCCGTGTTGATGGGGGGTTTTGGAAATTATTTAATTCCTTTATTACCCGGTTTACCAAATTTAAATTTCCCCCGGTTGAAACCCTTGAAAGCAGGGTGGCTTTTCCCTTCAATTTTATTTCTGGGGTTGAGAATGTGTTGGGGGGCGGGAAAAGGGGGAACTTTTTACCCCCCCCTCTCTTCTTCCCTTTTTAGCAATAGCCGGGGGGCCAATCTTTTGATGTTTTCTTTACTTTTGGCGGGCCTTTCAAGTTTGTTGGGTTCAATAAATTTTATATGAACTCTTTATTCGGCTTTTGTTGATAATTTTGTGTCTCGAAGTTCTATTTTGTTGTGATCTTATTTATTTACTCCAATCCTTTTATGGTTAACTATTCCTGTGTGGGCTGCTGCTATTACAATGTTGTTGTTTGATCGTAAATTTGGTTCGGCTTTTTTTGATCCTTTGGGTGGGGGTGATCCCGTTTTGTTTCAACATATGTTTTGGTTTTTTGGGCATCCGGAAGTTTATGTATTGATTTTGCCTGGTTTTGGTATGGTAAGTCATGTGTGTAGTAAATTAGGTTGTTCATATGATACTTTTGGTTTTTATGGTTTACTGTTTGCTATGTTTTCTATAGTGTGTTTAGGTAGCGTGGTTTGGGGCCACCACATGTTTACTGTGGGGTTGGATGTGAAGACTGCTGTTTTCTTTAGTTCTGTGACCATGATTATTGGGGTTCCCACGGGTATAAAGGTGTTTTCTTGGCTTTATATGATTTTAAATAGTCGTGTTTCGTTGCGTGAGCCTGTGTTTTGATGGGTTTTATCTTTTATTGTGTTGTTTACTATGGGTGGTGTTACTGGTATAATTCTTTCTGCTTGTGTGTTGGATAAAATTTTGCATGACACGTGGTTTGTGGTGGCTCATTTTCATTATGTTATGTCTTTGGGTTCTTATATTAGGATTATTATATTTTTTGTTTGGTGATGGCCTGTTATCACAGGGGTTAGCCTGAATAAGTATTTGTTACAGTGTCATTGTATAGTATCAAATGTGGGTTTTAATTTGTGTTTTTTCCCTATGCATTATTTTGGTATTTGTGGTTTACCTCGGCGTGTTTGTGTGTATGAGTCAGGGTACGCTTGAGTTAATATGCTTTGTTCAATAGGTTCTTTTGTTTCTGCCTTTAGTGGTTGCTTTTTTATTTTTATTTTATGGGAGTCTTTAGCTAAAAAGAATGTTGTTATAGGTTATTATGGTAATTCTTCAACTTTGCTTAATCTGTGTTGGTCGCCAGTGCCTTATCACAGTAATTTTTTTGTGCGCGGATTATTTGTTGATTATTCTGTATTGGCTTTTTAG

>Hap_15

ATGGTTTTGTTGTTGCGTCGTAATGTGGTTGATTTGCCTACTAATTATTCTCTTAATTATTATTGATGTAGTGGTTTTATGATTTCGGCTTTTATGGTAGTTCAGGTAATCACTGGTGTGATTCTTTCACTTTTGTATGTGGCTGATTCAAGATTAAGTTTTCGTTGTGTTATGGATTTGAGAAAAGATTCTTTTTTTACTTGGGGAGTGCGCTATTGACACATTTGGGGGGTTAGTATTTTGTTTGTCCTGTTTTTTGTTCATATGGGACGTGCCTTATATTATTCAAGTTATACCAAGAAGGGGGTATGGAAAGTGGGGTTTATTTTATATCTTTTAACTATGGCCGAGGCTTTTTTGGGTTATATTCTACCTTGACATCAAATGTCATATTGGGCTGCTACTGTTTTGACGGCTATTGCCGAGAGTATTCCCTTAGTTGGTCCTACGGTGTTTAAGTATTTGGTGGGGGGTTTTTCTGTAACTAAAGTAACTTTGGTTCGTGTATTTTCAGCTCATGTTTGTTTAGGTTTTGTAATTTTAGGTTTAATGATTTTGCATCTTTTTTATTTGCATTCTTCTGGGTCCAATAACCCTTTATTTTCTTCTTTTGGGTATGGGGATGTTGTTTATTTTCACTCTTATTTCACCACTAAGGATTTTTTTTGTTTGGTCGTTCTATGCTGTATTTTGGTTGGATTTATGTGGTTGGTTCCTGATTTGGTGGTAGATACGGAGGGTTATCTAGAATCTGATCCTTTGGTGACTCCGGTGTCTATAAAGCCTGAGTGATATTTTTTGATTTATTATGCTATGCTTCGTTCTGTTGAGTCTAAGATAGGTGGTCTAGTGTTGGTGGCTAGATTATTATTTTTTATGTGGGTCCCAACTTTTAAAGACTCTAGTTCATATTTTGTTATTCGACAGGTGATTTTTTGGAGTTTCGTTTGTCTTTTTGTTGGGTTGACTTATTTAGGCTCATGTCACCCTGAGTACCCTTATTTGGGTATTTGTCAGTTATTTTCGGTTGGTGCTGTAGCTTTTATGTTTATTTATAAGCTATTTTGATCGAGTTATACTAAGTTGGGTTTTAGTATTTTTTTAGGATAAATGGCTAAGTTTAGTTTTTTTAGTTGGCTTTTTACGCTAGATCATAAGCGTGTGGGTATGATTTATACTTTAATTGGGATATGATCGGGTTTTGTTGGGTTGAGTTTTAGGGTAATGATACGTGTTAATTTTGTTGAGCCTTATTTTAATGTAATTTCTTCGGACTGTTATAAATTTTTGATAACTAATCATGGTATTATTATGATTTTTTTTTTTTTGATGCCCGTGTTGATGGGGGGTTTTGGAAATTATTTAATTCCTTTATTACCCGGTTTACCAAATTTAAATTTCCCCCGGTTGAAACCCTTGAAAGCAGGGTGGCTTTTCCCTTCAATTTTATTTCTGGGGTTGAGAATGTGTTGGGGGGCGGGAAAAGGGGGAACTTTTTACCCCCCCCTCTCTTCTTCCCTTTTTAGCAATAGCCGGGGGGCCAATCTTTTGATGTTTTCTTTACTTTTGGCGGGCCTTTCAAGTTTGTTGGGTTCAATAAATTTTATATGAACTCTTTATTCGGCTTTTGTTGATAATTTTGTGTCTCGAAGTTCTATTTTGTTGTGATCTTATTTATTTACTCCAATCCTTTTATGGTTAACTATTCCTGTGTGGGCTGCTGCTATTACAATGTTGTTGTTTGATCGTAAATTTGGTTCGGCTTTTTTTGATCCTTTGGGTGGGGGTGATCCCGTTTTGTTTCAACATATGTTTTGGTTTTTTGGGCATCCGGAAGTTTATGTATTGATTTTGCCTGGTTTTGGTATGGTAAGTCATGTGTGTAGTAAATTAGGTTGTTCATATGATACTTTTGGTTTTTATGGTTTACTGTTTGCTATGTTTTCTATAGTGTGTTTAGGTAGCGTGGTTTGGGGCCACCACATGTTTACTGTGGGGTTGGATGTGAAGACTGCTGTTTTCTTTAGTTCTGTGACCATGATTATTGGGGTTCCCACGGGTATAAAGGTGTTTTCTTGGCTTTATATGATTTTAAATAGTCGTGTTTCGTTGCGTGAGCCTGTGTTTTGATGGGTTTTATCTTTTATTGTGTTGTTTACTATGGGTGGTGTTACTGGTATAATTCTTTCTGCTTGTGTGTTGGATAAAATTTTGCATGACACGTGGTTTGTGGTGGCTCATTTTCATTATGTTATGTCTTTGGGTTCTTATATTAGGATTATTATATTTTTTGTTTGGTGATGGCCTGTTATCACAGGGGTTAGCCTGAATAAGTATTTGTTACAGTGTCATTGTATAGTATCAAATGTGGGTTTTAATTTGTGTTTTTTCCCTATGCATTATTTTGGTATTTGTGGTTTACCTCGGCGTGTTTGTGTGTATGAGTCAGGGTACGCTTGAGTTAATATGCTTTGTTCAATAGGTTCTTTTGTTTCTGCCTTTAGTGGTTGCTTTTTTATTTTTATTTTATGGGAGTCTTTAGCTAAAAAGAATGTTGTTATAGGTTATTATGGTAATTCTTCAACTTTGCTTAATCTGTGTTGGTCGCCAGTGCCTTATCACAGTAATTTTTTTGTGCGCGGATTATTTGTTGATTATTCTGTATTGGCTTTTTAG

>Hap_16

ATGGTTTTGTTGTTGCGTCGTAATGTGGTTGATTTGCCTACTAATTATTCTCTTAATTATTATTGATGTAGTGGTTTTATGATTTCGGCTTTTATGGTAGTTCAGGTAATCACTGGTGTGATTCTTTCACTTTTGTATGTGGCTGATTCAAGATTAAGTTTTCGTTGTGTTATGGATTTGAGAAAAGATTCTTTTTTTACTTGGGGAGTGCGCTATTGACACATTTGGGGGGTTAGTATTTTGTTTGTCCTGTTTTTTGTTCATATGGGACGTGCCTTATATTATTCAAGTTATACTAAGAAGGGGGTATGGAAAGTGGGGTTTATTTTATATCTTTTAACTATGGCCGAGGCTTTTTTGGGTTATATTTTACCTTGACATCAAATGTCATATTGGGCTGCTACTGTTTTGACGGCTATTGCCGAGAGTATTCCCTTAGTCGGTCCTACGGTGTTTAAGTATTTGGTGGGGGGTTTTTCTGTAACCAAAGTAACTTTGGTTCGTGTATTTTCAGCTCATGTTTGTTTAGGTTTTGTAATTTTAGGTTTAATGATTTTGCATCTTTTTTATTTGCATTCTTCTGGGTCCAATAACCCTTTATTTTCTTCTTTTGGGTATGGGGATGTTGTTTATTTCCACTCTTATTTCACCACTAAGGATTTTTTTTGTTTGGTCGTTCTATGCTGTATTTTGGTTGGATTTATGTGGTTGGTTCCTGATTTGGTGGTAGATACGGAGGGTTATCTAGAATCTGATCCTCTGGTGACCCCGGTGTCTATAAAGCCTGAGTGATATTTTTTGATTTATTATGCTATGCTTCGTTCTGTTGAGTCTAAGATAGGTGGTCTAGTGTTGGTGGCTAGATTATTATTTTTTATGTGGGTCCCAACTTTTAAAGACTCTAGTTCATATTTTGTTATTCGACAGGTGATTTTTTGGAGTTTCGTTTGTCTTTTTGTTGGGTTGACTTATTTAGGCTCATGTCACCCTGAGTACCCTTATTTGGGTATTTGTCAGTTATTTTCGGTTGGTGCTGTAGCTTTTATGTTTATTTATAAGCTATTTTGATCGAGTTATACTAAGTTGGGTTTTAGTATTTTTTTAGGATAAATGGCTAAGTTTAGTTTTTTTAGTTGGCTTTTTACGCTAGATCATAAGCGTGTGGGTATGATTTATACTTTAATTGGGATATGATCGGGTTTTGTTGGGTTGAGTTTTAGGGTAATGATACGTGTTAATTTTGTTGAGCCTTATTTTAATGTAATTTCTTCGGACTGTTATAAATTTTTGATAACTAATCATGGTATTATTATGATTTTTTTTTTTTTGATCCCCGGGTTGATGGGGGGTTTGGGAAATTATTTGATTCCTTTATTACCCGGTTTACCAAATTTAAATTTACCCCGGTTGAAACCCTTGAAAGCAGGGTGGCTTTTTCCTTCAATTTTATTTCTGGGGTTGAGTATGGGTTGGGGGGCGGGAAAAGGGGGGACTTTTTACCCCCCCCTCTCTTCTTCCCTTTTTAGCAATAGCCGGGGGGCCAATCTTTTGAGGTTTTCTTTACATTTGGCGGGTCTTTCAAGTTTGTTGGGTTCTATAAATTTTATATGTACTCTTTATTCGGCTTTTGTTGATAATTTTGTGTCTCGAAGTTCTATTTTGTTGTGATCTTATTTATTTACTCCAATCCTTTTATGGTTAACTATTCCTGTGTGGGCTGCTGCTATTACAATGTTGTTGTTTGATCGTAAATTTGGTTCGGCTTTTTTTGATCCTTTGGGTGGGGGTGATCCCGTTTTGTTTCAACATATGTTTTGGTTTTTTGGGCATCCGGAAGTTTATGTATTGATTTTGCCTGGTTTTGGTATGGTAAGTCATGTGTGTAGTAAATTAGGTTGTTCATATGATACTTTTGGTTTTTATGGTTTACTATTTGCTATGTTTTCTATAGTGTGTTTAGGTAGCGTGGTTTGGGGCCACCACATGTTTACTGTGGGGTTGGATGTGAAGACTGCTGTTTTCTTTAGTTCTGTGACCATGATTATTGGGGTTCCCACGGGTATAAAGGTGTTTTCTTGGCTTTATATGATTTTAAATAGTCGTGTTTCGTTGCGTGAGCCTGTGTTTTGATGGGTTTTATCTTTTATTGTGTTGTTTACTATGGGTGGTGTTACTGGTATAATTCTTTCTGCTTGTGTGTTGGATAAAATTTTGCATGACACGTGGTTTGTGGTGGCTCATTTTCATTATGTTATGTCTTTGGGTTCTTATATTAGGATTATTATATTTTTTGTTTGGTGATGGCCTGTTATCACAGGGGTTAGCCTGAATAAGTATTTGTTACAGTGTCATTGTATAGTATCAAATGTGGGTTTTAATTTGTGTTTTTTCCCTATGCATTATTTTGGTATTTGTGGTTTACCTCGGCGTGTTTGTGTGTATGAGTCAGGGTACGCTTGAGTTAATATGCTTTGTTCAATAGGTTCTTTTGTTTCTGCCTTTAGTGGTTGCTTTTTTATTTTTATTTTATGGGAGTCTTTAGCTAAAAAGAATGTTGTTATAGGTTATTATGGTAGTTCTTCAACTTTGCTTAATCTGTGTTGGTCGCCAGTGCCTTATCACAGTAATTTTTTTGTGCGCGGATTATTTGTTGATTATTCTGTATTGGCTTTTTAG

>Hap_17

ATGGTTTTGTTGTTGCGTCGTAATGTGGTTGATTTGCCTACTAATTATTCTCTTAATTATTATTGATGTAGTGGTTTTATGATTTCGGCTTTTATGGTAGTTCAGGTAATCACTGGTGTGATTCTTTCACTTTTGTATGTGGCTGATTCAAGATTAAGTTTTCGTTGTGTTATGGATTTGAGAAAAGATTCTTTTTTTACTTGGGGAGTGCGCTATTGACACATTTGGGGGGTTAGTATTTTGTTTGTCCTGTTTTTTGTTCATATGGGACGTGCCTTATATTATTCAAGTTATACCAAGAAGGGGGTATGGAAAGTGGGGTTTATTTTATATCTTTTAACTATGGCCGAGGCTTTTTTGGGTTATATTCTACCTTGACATCAAATGTCATATTGGGCTGCTACTGTTTTGACGGCTATTGCCGAGAGTATTCCCTTAGTTGGTCCTACGGTGTTTAAGTATTTGGTGGGGGGTTTTTCTGTAACTAAAGTAACTTTGGTTCGTGTATTTTCAGCTCATGTTTGTTTAGGTTTTGTAATTTTAGGTTTAATGATTTTGCATCTTTTTTATTTGCATTCTTCTGGGTCCAATAACCCTTTATTTTCTTCTTTTGGGTATGGGGATGTTGTTTATTTTCACTCTTATTTCACCACTAAGGATTTTTTTTGTTTGGTTGTTCTATGCTGTATTTTGGTTGGGTTTATGTGGTTGGTTCCTGATTTGGTGGTAGATACGGAGGGTTATCTAGAATCTGATCCTTTGGTGACTCCGGTGTCTATAAAGCCTGAGTGATATTTTTTGATTTATTATGCTATGCTTCGTTCTGTTGAGTCTAAGATAGGTGGTCTAGTGTGGGTGGCTAGATTATTATTTTTTATGTGGGTCCCAACTTTTAAAGACTCTAGTTCATATTTTGTTATTCGACAGGTGATTTTTTGGAGTTTCGTTTGTCTTTTTGTTGGGTTGACTTATTTAGGCTCATGTCACCCTGAGTACCCTTATTTGGGTATTTGTCAGTTATTTTCGGTTGGTGCTGTAGCTTTTATGTTTATTTATAAGCTATTTTGATCGAGTTATACTAAGTTGGGTTTTAGTATTTTTTTAGGATAAATGGCTAAGTTTAGTTTTTTTAGTTGGCTTTTTACGCTAGATCATAAGCGTGTGGGTATGATTTATACTTTAATTGGGATATGATCGGGTTTTGTTGGGTTGAGTTTTAGGGTAATGATACGTGTTAATTTTGTTGAGCCTTATTTTAATGTAATTTCTTCGGACTGTTATAAATTTTTGATAACTAATCATGGTATTATTATGATTTTTTTTTTTTTGATGCCCGGGTTGATGGGGGGGTTGGGAAATTATTTGATTCCTTTATTATCCGGTTTACCAAATTTAAATTTACCCCGGTTGAAACCCTTGAAAGCAGGGTGGCTTTTTCCTTCTATTTTATTTCTGGGGTTGAGTAGGGGTTGGGGGGCGGGAAAAGGGGGAACTTTTTACCCCCCCCTCTCTTCTTCCCTTTTTAGCAATAGCCGGGGGGCCAATCTTTTGATGTTTTCTTTACTTTTGGCGGGCCTTTCAAGTTTGTGGGGTTCAATAAATTTTATATGTACTCTTTATTCGGCTTTTGTTGATAATTTTGTGTCTCGAAGTTCTATTTTGTTGTGGTCTTATTTATTTACTTCTATCCTTTTATTGTTAACTATTCCTGTGTGGGCTGCTGCTATTACAATGTTGTTGTTTGATCGTAAATTTGGTTCGGCTTTTTTTGATCCTTTGGGTGGGGGTGATCCTGTTTTGTTTCAACATATGTTTTGATTTTTTGGGCATCCAGAAGTTTATGTATTGATTTTGCCCGGTTTTGGTATGGTAAGTCATGTGTGTAGTAAATTAGGTTGTTCATATGATACTTTTGGTTTTTATGGTTTACTGTTTGCTATGTTTTCTATAGTGTGTTTAGGTAGCGTGGTTTGGGGTCACCACATGTTTACTGTGGGGTTGGATGTGAAGACTGCTGTTTTCTTTAGTTCTGTGACCATGATTATTGGGGTTCCCACGGGTATAAAGGTGTTTTCTTGGCTTTATATGATTTTAAATAGTCGTGTTTCGTTGCGTGAGCCTGTGTTTTGATGGGTTTTATCTTTTATTGTGTTGTTTACTATGGGTGGTGTTACTGGTATAATTCTTTCTGCTTGTGTGTTGGATAAAATTTTGCATGACACGTGGTTTGTGGTGGCTCATTTTCATTATGTTATGTCTTTGGGTTCTTATATTAGGGTTATTATATTTTTTGTTTGGTGATGGCCTGTTATTACAGGGGTTAGCCTGAATAAGTATTTGTTACAGTGTCATTGTATAGTATCAAATGTGGGTTTTAATTTGTGTTTTTTTCCTATGCATTATTTTGGTATTTGTGGTTTACCTCGGCGTGTTTGTGTGTATGAGTCAGGGTACGCTTGAGTTAATATGCTTTGTTCAATAGGTTCTTTTGTTTCTGCCTTTAGTGGTTGCTTTTTTATTTTTATTTTATGGGAGTCTTTAGCTAAAAAGAATGTTGTTATAGGTTATTATGGTAATTCTTCAACTTTGCTTAATTTGTGTTGATCGCCAGTGCCTTACCACAGTAATTTTTTTGTGCGCGGATTATTTGTTGATTATTCTGTATTGGCTTTTTAG

>Hap_18

ATGGTTTTGTTGTTGCGTCGTAATGTGGTTGATTTGCCTACTAATTATTCTCTTAATTATTATTGATGTAGTGGTTTTATGATTTCGGCTTTTATGGTAGTTCAGGTAATCACTGGTGTGATTCTTTCACTTTTGTATGTGGCTGATTCAAGATTAAGTTTTCGTTGTGTTATGGATTTGAGAAAAGATTCTTTTTTTACTTGGGGAGTGCGCTATTGACACATTTGGGGGGTTAGTATTTTGTTTGTCCTGTTTTTTGTTCATATGGGACGTGCCTTATATTATTCAAGTTATACTAAGAAGGGGGTATGGAAAGTGGGGTTTATTTTATATCTTTTAACTATGGCCGAGGCTTTTTTGGGTTATATTTTACCTTGACATCAAATGTCATATTGGGCTGCTACTGTTTTGACGGCTATTGCCGAGAGTATTCCCTTAGTCGGTCCTACGGTGTTTAAGTATTTGGTGGGGGGTTTTTCTGTAACCAAAGTAACTTTGGTTCGTGTATTTTCAGCTCATGTTTGTTTAGGTTTTGTAATTTTAGGTTTAATGATTTTGCATCTTTTTTATTTGCATTCTTCTGGGTCCAATAACCCTTTATTTTCTTCTTTTGGGTATGGGGATGTTGTTTATTTCCACTCTTATTTCACCACTAAGGATTTTTTTTGTTTGGTCGTTCTATGCTGTATTTTGGTTGGATTTATGTGGTTGGTTCCTGATTTGGTGGTAGATACGGAGGGTTATCTAGAATCTGATCCTCTGGTGACTCCGGTGTCTATAAAGGCTGACTGATATTTTTTGATTTATTATGCTATGCTTCGTTCTGTTGAGTCTAAGATAGGTGGTCTAGTGTGGGTGGCTAGATTATTATTTTTTATGTGGGTCCCAACTTTTAAAGACTCTACTTCATATTTTGTTATTCGACAGGTGATTTTTGGGAGGTTCGTTTGTCTTTTTGTTGGGGTGACTTATTTAGGCTCATGTCACCCTGAGTACCCTTATTTGGGTATTTGTCAGTTATTTTCGGTTGGTGCTGTAGCTTTTATGTTTATTTATAAGCTATTTTGATCGAGTTATACTAAGTTGGGTTTTAGTATTTTTTTAGGATAAATGGCTAAGTTTAGTTTTTTTAGTTGGCTTTTTACGCTAGATCATAAGCGTGTGGGTATGATTTATACTTTAATTGGGATATGATCGGGTTTTGTTGGGTTGAGTTTTAGGGTAATGATACGTGTTAATTTTGTTGAGCCTTATTTTAATGTAATTTCTTCGGACTGTTATAAATTTTTGATAACTAATCATGGTATTATTATGATTTTTTTTTTTTTGATGCCCGGGTTGATGGGGGGTTTTGGAAATTATTTGATTCCTTTATTATCCGGTTTACCAAATTTAAATTTACCCCGGTTGAAACCCTTGAAAGCAGGGTGGCTTTTTCCTTCTATTTTATTTCTGGGGTTGAGTATGGGTTGGGGGGCGGGAAAAGGGGGGACTTTTTACCCCCCCCTCTCTTCTTCCCTTTTTAGCAATAGCCGGGGGGCCAATCTTTTGAGGTTTTCTTTACTTTTGGCGGGCCTTTCAAGTTTGTGGGGTTCAATAAATTTTATATGTACTCTTTATTCGGCTTTTGTTGATAATTTTGTGTCTCGAAGTTCTATTTTGTTGTGGTCTTATTTATTTACTTCAATCCTTTTATTGTTAACTATTCCTGTGTGGGCTGCTGCTATTACAATGTTGTTGTTTGATCGTAAATTTGGTTCGGCTTTTTTTGATCCTTTGGGTGGGGGTGATCCCGTTTTGTTTCAACATATGTTTTGGTTTTTTGGGCATCCGGAAGTTTATGTATTGATTTTGCCTGGTTTTGGTATGGTAAGTCATGTGTGTAGTAAATTAGGTTGTTCATATGATACTTTTGGTTTTTATGGTTTACTGTTTGCTATGTTTTCTATAGTGTGTTTAGGTAGCGTGGTTTGGGGCCACCACATGTTTACTGTGGGGTTGGATGTGAAGACTGCTGTTTTCTTTAGTTCTGTGACCATGATTATTGGGGTTCCCACGGGTATAAAGGTGTTTTCTTGGCTTTATATGATTTTAAATAGTCGTGTTTCGTTGCGTGAGCCTGTGTTTTGATGGGTTTTATCTTTTATTGTGTTGTTTACTATGGGTGGTGTTACTGGTATAATTCTTTCTGCTTGTGTGTTGGATAAAATTTTGCATGACACGTGGTTTGTGGTGGCTCATTTTCATTATGTTATGTCTTTGGGTTCTTATATTAGGATTATTATATTTTTTGTTTGGTGATGGCCTGTTATCACAGGGGTTAGCCTGAATAAGTATTTGTTACAGTGTCATTGTATAGTATCAAATGTGGGTTTTAATTTGTGTTTTTTCCCTATGCATTATTTTGGTATTTGTGGTTTACCTCGGCGTGTTTGTGTGTATGAGTCAGGGTACGCTTGAGTTAATATGCTTTGTTCAATAGGTTCTTTTGTTTCTGCCTTTAGTGGTTGCTTTTTTATTTTTATTTTATGGGAGTCTTTAGCTAAAAAGAATGTTGTTATAGGTTATTATGGTAGTTCTTCAACTTTGCTTAATCTGTGTTGGTCGCCAGTGCCTTATCACAGTAATTTTTTTGTGCGCGGATTATTTGTTGATTATTCTGTATTGGCTTTTTAG

>Hap_19

ATGGTTTTGTTGTTGCGTCGTAATGTGGTTGATTTGCCTACTAATTATTCTCTTAATTATTATTGATGTAGTGGTTTTATGATTTCGGCTTTTATGGTAGTTCAGGTAATCACTGGTGTGATTCTTTCACTTTTGTATGTGGCTGATTCAAGATTAAGTTTTCGTTGTGTTATGGATTTGAGAAAAGATTCTTTTTTTACTTGGGGAGTGCGCTATTGACACATTTGGGGGGTTAGTATTTTGTTTGTCCTGTTTTTTGTTCATATGGGACGTGCCTTATATTATTCAAGTTATACTAAGAAGGGGGTATGGAAAGTGGGGTTTATTTTATATCTTTTAACTATGGCCGAGGCTTTTTTGGGTTATATTTTACCTTGACATCAAATGTCATATTGGGCTGCTACTGTTTTGACGGCTATTGCCGAGAGTATTCCCTTAGTCGGTCCTACGGTGTTTAAGTATTTGGTGGGGGGTTTTTCTGTAACCAAAGTAACTTTGGTTCGTGTATTTTCAGCTCATGTTTGTTTAGGTTTTGTAATTTTAGGTTTAATGATTTTGCATCTTTTTTATTTGCATTCTTCTGGGTCCAATAACCCTTTATTTTCTTCTTTTGGGTATGGGGATGTTGTTTATTTCCACTCTTATTTCACCACTAAGGATTTTTTTTGTTTGGTCGTTCTATGCTGTATTTTGGTTGGATTTATGTGGTTGGTTCCTGATTTGGTGGTAGATACGGAGGGTTATCTAGAATCTGATCCTCTGGTGACTCCGGTGTCTATAAAGCCTGAGTGATATTTTTTGATTTATTATGCTATGCTTCGTTCTGTTGAGTCTAAGATAGGTGGTCTAGTGTTGGTGGCTAGATTATTATTTTTTATGTGGGTCCCAACTTTTAAAGACTCTAGTTCATATTTTGTTATTCGACAGGTGATTTTTTGGAGTTTCGTTTGTCTTTTTGTTGGGTTGACTTATTTAGGCTCATGTCACCCTGAGTACCCTTATTTGGGTATTTGTCAGTTATTTTCGGTTGGTGCTGTAGCTTTTATGTTTATTTATAAGCTATTTTGATCGAGTTATACTAAGTTGGGTTTTAGTATTTTTTTAGGATAAATGGCTAAGTTTAGTTTTTTTAGTTGGCTTTTTACGCTAGATCATAAGCGTGTGGGTATGATTTATACTTTAATTGGGATATGATCGGGTTTTGTTGGGTTGAGTTTTAGGGTAATGATACGTGTTAATTTTGTTGAGCCTTATTTTAATGTAATTTCTTCGGACTGTTATAAATTTTTGATAACTAATCATGGTATTATTATGATTTTTTTTTTTTTGATGCCCGTGTTGATGGGGGGTTTGGGAAATTATTTGATTCCTTTATTATCCGGTTTACCAAATTTAAATTTACCTCGGTTGAAACCCTTGAAAGCAGGGTTGCTTTTTCCTTCTATTTTATTTCTGGGGTTGAGTATGGGTTGGGGGGCGGGTATAGGGGGGACTTTTTACCCCCCCCTCTCTTCTTCTCTTTTTAGCAATAGCCGGGGGGCCAATCTTTTGAGGTTTTCTTTACATTTGGCGGGCCTTTCAAGTTTGTTGGGTTCAATAAATTTTATATGTACTCTTTATTCGGCTTTTGTTGATAATTTTGTGTCTCGAAGTTCTATTTTGTTGTGGTCTTATTTATTTACTTCAATCCTTTTATTGTTAACTATTCCTGTGTGGGCTGCTGCTATTACAATGTTGTTGTTTGATCGTAAATTTGGTTCGGCTTTTTTTGATCCTTTGGGTGGGGGTGATCCCGTTTTGTTTCAACATATGTTTTGGTTTTTTGGGCATCCGGAAGTTTATGTATTGATTTTGCCTGGTTTTGGTATGGTAAGTCATGTGTGTAGTAAATTAGGTTGTTCATATGATACTTTTGGTTTTTATGGTTTACTGTTTGCTATGTTTTCTATAGTGTGTTTAGGTAGCGTGGTTTGGGGCCACCACATGTTTACTGTGGGGTTGGATGTGAAGACTGCTGTTTTCTTTAGTTCTGTGACCATGATTATTGGGGTTCCCACGGGTATAAAGGTGTTTTCTTGGCTTTATATGATTTTAAATAGTCGTGTTTCGTTGCGTGAGCCTGTGTTTTGATGGGTTTTATCTTTTATTGTGTTGTTTACTATGGGTGGTGTTACTGGTATAATTCTTTCTGCTTGTGTGTTGGATAAAATTTTGCATGACACGTGGTTTGTGGTGGCTCATTTTCATTATGTTATGTCTTTGGGTTCTTATATTAGGATTATTATATTTTTTGTTTGGTGATGGCCTGTTATCACAGGGGTTAGCCTGAATAAGTATTTGTTACAGTGTCATTGTATAGTATCAAATGTGGGTTTTAATTTGTGTTTTTTCCCTATGCATTATTTTGGTATTTGTGGTTTACCTCGGCGTGTTTGTGTGTATGAGTCAGGGTACGCTTGAGTTAATATGCTTTGTTCAATAGGTTCTTTTGTTTCTGCCTTTAGTGGTTGCTTTTTTATTTTTATTTTATGGGAGTCTTTAGCTAAAAAGAATGTTGTTATAGGTTATTATGGTAGTTCTTCAACTTTGCTTAATCTGTGTTGGTCGCCAGTGCCTTATCACAGTAATTTTTTTGTGCGCGGATTATTTGTTGATTATTCTGTATTGGCTTTTTAG

>Hap_20

ATGGTTTTGTTGTTGCGTCGTAATGTGGTTGATTTGCCTACTAATTATTCTCTTAATTATTATTGATGTAGTGGTTTTATGATTTCGGCTTTTATGGTAGTTCAGGTAATCACTGGTGTGATTCTTTCACTTTTGTATGTGGCTGATTCAAGATTAAGTTTTCGTTGTGTTATGGATTTGAGAAAAGATTCTTTTTTTACTTGGGGAGTGCGCTATTGACACATTTGGGGGGTTAGTATTTTGTTTGTCCTGTTTTTTGTTCATATGGGACGTGCCTTATATTATTCAAGTTATACTAAGAAGGGGGTATGGAAAGTGGGGTTTATTTTATATCTTTTAACTATGGCCGAGGCTTTTTTGGGTTATATTTTACCTTGACATCAAATGTCATATTGGGCTGCTACTGTTTTGACGGCTATTGCCGAGAGTATTCCCTTAGTTGGTCCTACGGTGTTTAAGTATTTGGTGGGGGGTTTTTCTGTAACTAAAGTAACTTTGGTTCGTGTATTTTCAGCTCATGTTTGTTTAGGTTTTGTAATTTTAGGTTTAATGATTTTGCATCTTTTTTATTTGCATTCTTCTGGGTCCAATAACCCTTTATTTTCTTCTTTTGGGTATGGGGATGTTGTTTATTTCCACTCTTATTTCACCACTAAGGATTTTTTTTGTTTGGTTGTTCTATGCTGTATTTTGGTTGGATTTATGTGGTTGGTTCCTGATTTGGTGGTAGATACGGAGGGTTATCTAGAATCTGATCCTTTGGTGACTCCGGTGTCTATAAAGCCTGAGTGATATTTTTTGATTTATTATGCTATGCTTCGTTCTGTTGAGTCTAAGATAGGTGGTCTAGTGTGGGTGGCTAGATTATTATTTTTTATGTGGGTCCCAACTTTTAAAGACTCTAGTTCATATTTTGTTATTCGACAGGTGATTTTTTGGAGTTTCGTTTGTCTTTTTGTTGGGTTGACTTATTTAGGCTCATGTCACCCTGAGTACCCTTATTTGGGTATTTGTCAGTTATTTTCGGTTGGTGCTGTAGCTTTTATGTTTATTTATAAGCTATTTTGATCGAGTTATACTAAGTTGGGTTTTAGTATTTTTTTAGGATAAATGGCTAAGTTTAGTTTTTTTAGTTGGCTTTTTACGCTAGATCATAAGCGTGTGGGTATGATTTATACTTTAATTGGGATATGATCGGGTTTTGTTGGGTTGAGTTTTAGGGTAATGATACGTGTTAATTTTGTTGAGCCTTATTTTAATGTAATTTCTTCGGACTGTTATAAATTTTTGATAACTAATCATGGTATTATTATGATTTTTTTTTTTTTGATGCCCGGGTTGATGGGGGGTTTTGGAAATTATTTGATCCCTTTATTACCCGGTTTACCAAATTTAAATTTACCCCGGTTGAAACCCTTGAAAGCAGGGTGGCTTTTTCCTTCTATTTTATTTCTGGGGTTGAGTATGGGTTTGGGGGCGGGAATAGGGGGAACTTTTTACCCCCCCCTCTCTTCTTCTCTTTTTAGCAATAGCCGGGGGGCCAATCTTTTGATGTTTTCTTTACTTTTGGCGGGCCTTTCAAGTTTGTTGGGTTCAATAAATTTTATATGTACTCTTTATTCGGCTTTTGTTGATAATTTTGTGTCTCGAAGTTCTATTTTGTTGTGGTCTTATTTATTTACTTCTATCCTTTTATGGTTAACTATTCCTGTGTGGGCTGCTGCTATTACAATGTTGTTGTTTGATCGTAAATTTGGTTCGGCTTTTTTTGATCCTTTGGGTGGGGGTGATCCTGTTTTGTTTCAACATATGTTTTGGTTTTTTGGGCATCCGGAAGTTTATGTATTGATTTTGCCTGGTTTTGGTATGGTAAGTCATGTGTGTAGTAAATTAGGTTGTTCATATGATACTTTTGGTTTTTATGGTTTACTGTTTGCTATGTTTTCTATAGTGTGTTTAGGTAGTGTGGTTTGGGGCCACCACATGTTTACTGTGGGGTTGGATGTGAAGACTGCTGTTTTCTTTAGTTCTGTGACCATGATTATTGGGGTTCCCACGGGTATAAAGGTGTTTTCTTGGCTTTATATGATTTTAAATAGTCGTGTTTCGTTGCGTGAGCCTGTGTTTTGATGGGTTTTATCTTTTATTGTGTTGTTTACTATGGGTGGTGTTACTGGTATAATTCTTTCTGCTTGTGTGTTGGATAAAATTTTGCATGACACGTGGTTTGTGGTGGCTCATTTTCATTATGTTATGTCTTTGGGTTCTTATATTAGGGTTATTATATTTTTTGTTTGGTGATGGCCTGTTATTACAGGGGTTAGCCTGAATAAGTATTTGTTACAGTGTCATTGTATAGTATCAAATGTGGGTTTTAATTTGTGTTTTTTCCCTATGCATTATTTTGGTATTTGTGGTTTACCTCGGCGTGTTTGTGTGTATGAGTCAGGGTACGCTTGAGTTAATATGCTTTGTTCAATAGGTTCTTTTGTTTCTGCCTTTAGTGGTTGCTTTTTTATTTTTATTTTATGGGAGTCTTTAGCTAAAAAGAATGTTGTTATAGGTTATTATGGTAATTCTTCAACTTTGCTTAATCTGTGTTGATCGCCAGTGCCTTATCACAGTAATTTTTTTGTGCGCGGATTATTTGTTGATTATTCTGTATTGGCTTTTTAG

>Hap_21

ATGGTTTTGTTGTTGCGTCGTAATGTGGTTGATTTGCCTACTAATTATTCTCTTAATTATTATTGATGTAGTGGTTTTATGATTTCGGCTTTTATGGTAGTTCAGGTAATCACTGGTGTGATTCTTTCACTTTTGTATGTGGCTGATTCAAGATTAAGTTTTCGTTGTGTTATGGATTTGAGAAAAGATTCTTTTTTTACTTGGGGAGTGCGCTATTGACACATTTGGGGGGTTAGTATTTTGTTTGTCCTGTTTTTTGTTCATATGGGACGTGCCTTATATTATTCAAGTTATACCAAGAAGGGGGTATGGAAAGTGGGGTTTATTTTATATCTTTTAACTATGGCCGAGGCTTTTTTGGGTTATATTCTACCTTGACATCAAATGTCATATTGGGCTGCTACTGTTTTGACGGCTATTGCCGAGAGTATTCCCTTAGTCGGTCCTACGGTGTTTAAGTATTTGGTGGGGGGTTTTTCTGTAACCAAAGTAACTTTGGTTCGTGTATTTTCAGCTCATGTTTGTTTAGGTTTTGTAATTTTAGGTTTAATGATTTTGCATCTTTTTTATTTGCATTCTTCTGGGTCCAATAACCCTTTATTTTCTTCTTTTGGGTATGGGGATGTTGTTTATTTCCACTCTTATTTCACCACTAAGGATTTTTTTTGTTTGGTCGTTCTATGCTGTATTTTGGTTGGATTTATGTGGTTGGTTCCTGATTTGGTGGTAGATACGGAGGGTTATCTAGAATCTGATCCTCTGGTGACTCCGGTGTCTATAAAGCCTGAGTGATATTTTTTGATTTATTATGCTATGCTTCGTTCTGTTGAGTCTAAGATAGGTGGTCTAGTGTTGGTGGCTAGATTATTATTTTTTATGTGGGTCCCAACTTTTAAAGACTCTAGTTCATATTTTGTTATTCGACAGGTGATTTTTTGGAGTTTCGTTTGTCTTTTTGTTGGGGTGACTTATTTAGGCTCATGTCACCCTGAGTACCCTTATTTGGGTATTTGTCAGTTATTTTCGGTTGGTGCTGTAGCTTTTATGTTTATTTATAAGCTATTTTGATCGAGTTATACTAAGTTGGGTTTTAGTATTTTTTTAGGATAAATGGCTAAGTTTAGTTTTTTTAGTTGGCTTTTTACGCTAGATCATAAGCGTGTGGGTATGATTTATACTTTAATTGGGATATGATCGGGTTTTGTTGGGTTGAGTTTTAGGGTAATGATACGTGTTAATTTTGTTGAGCCTTATTTTAATGTAATTTCTTCGGACTGTTATAAATTTTTGATAACTAATCATGGTATTATTATGATTTTTTTTTTTTTGATGCCCGGGTTGATGGGGGGGTTGGGAAATTATTTGATTCCTTTATTACCCGGTTTACCAAATTTAAATTTCCCTCGGTTGAAACCCTTGAAAGCAGGGTGGCTTTTCCCTCCTATTTTATTTCTGGGGTTGAGAAGGGGTTGGGGGGCGGGAAAAGGGGGGACTTTTTACCCCCCCCTCTCTTCTTCCCTTTTTAGCAATAGCCGGGGGGCCAATCTTTTGATGTTTTCTTTACTTTTGGCGGGCCTTTCAAGTTTGTGGGGTTCAATAAATTTTATATGTACTCTTTATTCGGCTTTTGTTGATAATTTTGTGTCTCGAAGTTCTATTTTGTTGTGATCTTATTTATTTACTTCTATCCTTTTATGGTTAACTATTCCTGTGTGGGCTGCTGCTATTACAATGTTGTTGTTTGATCGTAAATTTGGTTCGGCTTTTTTTGATCCTTTGGGTGGGGGTGATCCTGTTTTGTTTCAACATATGTTTTGATTTTTTGGGCATCCGGAAGTTTATGTATTGATTTTGCCCGGTTTTGGTATGGTAAGTCATGTGTGTAGTAAATTAGGTTGTTCATATGATACTTTTGGTTTTTATGGTTTACTGTTTGCTATGTTTTCTATAGTGTGTTTAGGTAGTGTGGTTTGGGGCCACCACATGTTTACTGTGGGGTTGGATGTGAAGACTGCTGTTTTCTTTAGTTCTGTGACCATGATTATTGGGGTTCCCACGGGTATAAAGGTGTTTTCTTGGCTTTATATGATTTTAAATAGTCGTGTTTCGTTGCGTGAGCCTGTGTTTTGATGGGTTTTATCTTTTATTGTGTTGTTTACTATGGGTGGTGTTACTGGTATAATTCTTTCTGCTTGTGTGTTGGATAAAATTTTGCATGACACGTGGTTTGTGGTGGCTCATTTTCATTATGTTATGTCTTTGGGTTCTTATATTAGGGTTATTATATTTTTTGTTTGGTGATGGCCTGTTATTACAGGGGTTAGCCTGAATAAGTATTTGTTACAGTGTCATTGTATAGTATCAAATGTGGGTTTTAATTTGTGTTTTTTCCCTATGCATTATTTTGGTATTTGTGGTTTACCTCGGCGTGTTTGTGTGTATGAGTCAGGGTACGCTTGAGTTAATATGCTTTGTTCAATAGGTTCTTTTGTTTCTGCCTTTAGTGGTTGCTTTTTTATTTTTATTTTATGGGAGTCTTTAGCTAAAAAGAATGTTGTTATAGGTTATTATGGTAATTCTTCAACTTTGCTTAATCTGTGTTGATCGCCAGTGCCTTATCACAGTAATTTTTTTGTGCGCGGATTATTTGTTGATTATTCTGTATTGGCTTTTTAG

>Hap_22

ATGGTTTTGTTGTTGCGTCGTAATGTGGTTGATTTGCCTACTAATTATTCTCTTAATTATTATTGATGTAGTGGTTTTATGATTTCGGCTTTTATGGTAGTTCAGGTAATCACTGGTGTGATTCTTTCACTTTTGTATGTGGCTGATTCAAGATTAAGTTTTCGTTGTGTTATGGATTTGAGAAAAGATTCTTTTTTTACTTGGGGAGTGCGCTATTGACACATTTGGGGGGTTAGTATTTTGTTTGTCCTGTTTTTTGTTCATATGGGACGTGCCTTATATTATTCAAGTTATACCAAGAAGGGGGTATGGAAAGTGGGGTTTATTTTATATCTTTTAACTATGGCCGAGGCTTTTTTGGGTTATATTCTACCTTGACATCAAATGTCATATTGGGCTGCTACTGTTTTGACGGCTATTGCCGAGAGTATTCCCTTAGTTGGTCCTACGGTGTTTAAGTATTTGGTGGGGGGTTTTTCTGTAACTAAAGTAACTTTGGTTCGTGTATTTTCAGCTCATGTTTGTTTAGGTTTTGTAATTTTAGGTTTAATGATTTTGCATCTTTTTTATTTGCATTCTTCTGGGTCCAATAACCCTTTATTTTCTTCTTTTGGGTATGGGGATGTTGTTTATTTTCACTCTTATTTCACCACTAAGGATTTTTTTTGTTTGGTTGTTCTATGCTGTATTTTGGTTGGGTTTATGTGGTTGGTTCCTGATTTGGTGGTAGATACGGAGGGTTATCTAGAATCTGATCCTTTGGTGACTCCGGTGTCTATAAAGCCTGAGTGATATTTTTTGATTTATTATGCTATGCTTCGTTCTGTTGAGTCTAAGATAGGTGGTCTAGTGTTGGTGGCTAGATTATTATTTTTTATGTGGGTCCCAACTTTTAAAGACTCTAGTTCATATTTTGTTATTCGACAGGTGATTTTTTGGAGTTTCGTTTGTCTTTTTGTTGGGGTGACTTATTTAGGCTCATGTCACCCTGAGTACCCTTATTTGGGTATTTGTCAGTTATTTTCGGTTGGTGCTGTAGCTTTTATGTTTATTTATAAGCTATTTTGATCGAGTTATACTAAGTTGGGTTTTAGTATTTTTTTAGGATAAATGGCTAAGTTTAGTTTTTTTAGTTGGCTTTTTACGCTAGATCATAAGCGTGTGGGTATGATTTATACTTTAATTGGGATATGATCGGGTTTTGTTGGGTTGAGTTTTAGGGTAATGATACGTGTTAATTTTGTTGAGCCTTATTTTAATGTAATTTCTTCGGACTGTTATAAATTTTTGATAACTAATCATGGTATTATTATGATTTTTTTTTTTTTGATGCCCGGGTTGATTGGGGGGTTTGGAAATTATTTGATTCCTTTATTATCCGGTTTACCAAATTTAAATTTACCTCGGTTGAAACCCTTGAAAGCAGGGTTGCTTTTTCCTTCTATTTTATTTCTGGGGTTGAGTATGTGTTTGGGGGCGGGTATAGGGGGAACTTTTTACCCCCCCCTCTCTTCTTCTCTTTTTAGCAATAGCCGGGGGGCCAATCTTTTGATGTTTTCTTTACATTTGGCGGGCCTTTCAAGTTTGTGGGGTTCAATAAATTTTATATGTACTCTTTATTCGGCTTTTGTTGATAATTTTGTGTCTCGAAGTTCTATTTTGTTGTGATCTTATTTATTTACTTCTATTCTTTTATGGTTAACTATTCCTGTGTGGGCTGCTGCTATTACAATGTTGTTGTTTGATCGTAAATTTGGTTCGGCTTTTTTTGATCCTTTGGGTGGGGGTGATCCTGTTTTGTTTCAACATATGTTTTGATTTTTTGGGCATCCGGAAGTTTATGTATTGATTTTGCCCGGTTTTGGTATGGTAAGTCATGTGTGTAGTAAATTAGGTTGTTCATATGATACTTTTGGTTTTTATGGTTTACTGTTTGCTATGTTTTCTATAGTGTGTTTAGGTAGTGTGGTTTGGGGCCACCACATGTTTACTGTGGGGTTGGATGTGAAGACTGCTGTTTTCTTTAGTTCTGTGACCATGATTATTGGGGTTCCCACGGGTATAAAGGTGTTTTCTTGGCTTTATATGATTTTAAATAGTCGTGTTTCGTTGCGTGAGCCTGTGTTTTGATGGGTTTTATCTTTTATTGTGTTGTTTACTATGGGTGGTGTTACTGGTATAATTCTTTCTGCTTGTGTGTTGGATAAAATTTTGCATGACACGTGGTTTGTGGTGGCTCATTTTCATTATGTTATGTCTTTGGGTTCTTATATTAGGGTTATTATATTTTTTGTTTGGTGATGGCCTGTTATTACAGGGGTTAGCCTGAATAAGTATTTGTTACAGTGTCATTGTATAGTATCAAATGTGGGTTTTAATTTGTGTTTTTTCCCTATGCATTATTTTGGTATTTGTGGTTTACCTCGGCGTGTTTGTGTGTATGAGTCAGGGTACGCTTGAGTTAATATGCTTTGTTCAATAGGTTCTTTTGTTTCTGCCTTTAGTGGTTGCTTTTTTATTTTTATTTTATGGGAGTCTTTAGCTAAAAAGAATGTTGTTATAGGTTATTATGGTAATTCTTCAACTTTGCTTAATCTGTGTTGATCGCCAGTGCCTTATCACAGTAATTTTTTTGTGCGCGGATTATTTGTTGATTATTCTGTATTGGCTTTTTAG

>Hap_23

ATGGTTTTGTTGTTGCGTCGTAATGTGGTTGATTTGCCTACTAATTATTCTCTTAATTATTATTGATGTAGTGGTTTTATGATTTCGGCTTTTATGGTAGTTCAGGTAATCACTGGTGTGGTTCTTTCACTTTTGTATGTGGCTGATTCAAGATTAAGTTTTCGTTGTGTTATGGATTTGAGAAAAGATTCTTTTTTTACTTGGGGAGTGCGCTATTGACACATTTGGGGGGTTAGTATTTTGTTTGTCCTGTTTTTTGTTCATATGGGACGTGCCTTATATTATTCAAGTTATACCAAGAAGGGGGTATGGAAAGTGGGGTTTATTTTATATCTTTTAACTATGGCCGAGGCTTTTTTGGGTTATATTCTACCTTGACATCAAATGTCATATTGGGCTGCTACTGTTTTGACGGCTATTGCCGAGAGTATTCCCTTAGTTGGTCCTACGGTGTTTAAGTATTTGGTGGGGGGTTTTTCTGTAACTAAAGTAACTTTGGTTCGTGTATTTTCAGCTCATGTTTGTTTAGGTTTTGTAATTTTAGGTTTAATGATTTTGCATCTTTTTTATTTGCATTCTTCTGGGTCCAATAACCCTTTATTTTCTTCTTTTGGGTATGGGGATGTTGTTTATTTTCACTCTTATTTCACCACTAAGGATTTTTTTTGTTTGGTTGTTCTATGCTGTATTTTGGTTGGGTTTATGTGGTTGGTTCCTGATTTGGTGGTAGATACGGAGGGTTATCTAGAATCTGATCCTTTGGTGACTCCGGTGTCTATAAAGCCTGAGTGATATTTTTTGATTTATTATGCTATGCTTCGTTCTGTTGAGTCTAAGATAGGTGGTCTAGTGTTGGTGGCTAGATTATTATTTTTTATGTGGGTCCCAACTTTTAAAGACTCTAGTTCATATTTTGTTATTCGACAGGTGATTTTTTGGAGTTTCGTTTGTCTTTTTGTTGGGGTGACTTATTTAGGCTCATGTCACCCTGAGTACCCTTATTTGGGTATTTGTCAGTTATTTTCGGTTGGTGCTGTAGCTTTTATGTTTATTTATAAGCTATTTTGATCGAGTTATACTAAGTTGGGTTTTAGTATTTTTTTAGGATAAATGGCTAAGTTTAGTTTTTTTAGTTGGCTTTTTACGCTAGATCATAAGCGTGTGGGTATGATTTATACTTTAATTGGGATATGATCGGGTTTTGTTGGGTTGAGTTTTAGGGTAATGATACGTGTTAATTTTGTTGAGCCTTATTTTAATGTAATTTCTTCGGACTGTTATAAATTTTTGATAACTAATCATGGTATTATTATGATTTTTTTTTTTTTGATGCCCGGGTTGATGGGGGGGTTGGGAAATTATTTGATTCCTTTATTACCCGGTTTACCAAATTTAAATTTACCCCGGTTGAAACCCTTGAAAGCAGGGTTGCTTTTTCCTTCTATTTTATTTCTGGGGTTGAGTATGTGTTGGGGGGCGGGAAAAGGGGGAACTTTTTACCCCCCCCTCTCTTCTTCTCTTTTTAGCAATAGCCGGGGGGCCAATCTTTTGATGTTTTCTTTACTTTTGGCGGGCCTTTCAAGTTTGTTGGGTTCAATAAATTTTATATGTACTCTTTATTCGGCTTTTGTTGATAATTTTGTGTCTCGAAGTTCTATTTTGTTGTGGTCTTATTTATTTACTTCTATCCTTTTATGGTTAACTATTCCTGTGTGGGCTGCTGCTATTACAATGTTGTTGTTTGATCGTAAATTTGGTTCGGCTTTTTTTGATCCTTTGGGTGGGGGTGATCCTGTTTTGTTTCAACATATGTTTTGATTTTTTGGGCATCCGGAAGTTTATGTATTGATTTTGCCCGGTTTTGGTATGGTAAGTCATGTGTGTAGTAAATTAGGTTGTTCATATGATACTTTTGGTTTTTATGGTTTACTGTTTGCTATGTTTTCTATAGTGTGTTTAGGTAGTGTGGTTTGGGGCCACCACATGTTTACTGTGGGGTTGGATGTGAAGACTGCTGTTTTCTTTAGTTCTGTGACCATGATTATTGGGGTTCCCACGGGTATAAAGGTGTTTTCTTGGCTTTATATGATTTTAAATAGTCGTGTTTCGTTGCGTGAGCCTGTGTTTTGATGGGTTTTATCTTTTATTGTGTTGTTTACTATGGGTGGTGTTACTGGTATAATTCTTTCTGCTTGTGTGTTGGATAAAATTTTGCATGACACGTGGTTTGTGGTGGCTCATTTTCATTATGTTATGTCTTTGGGTTCTTATATTAGGGTTATTATATTTTTTGTTTGGTGATGGCCTGTTATTACAGGGGTTAGCCTGAATAAGTATTTGTTACAGTGTCATTGTATAGTATCAAATGTGGGTTTTAATTTGTGTTTTTTCCCTATGCATTATTTTGGTATTTGTGGTTTACCTCGGCGTGTTTGTGTGTATGAGTCAGGGTACGCTTGAGTTAATATGCTTTGTTCAATAGGTTCTTTTGTTTCTGCCTTTAGTGGTTGCTTTTTTATTTTTATTTTATGGGAGTCTTTAGCTAAAAAGAATGTTGTTATAGGTTATTATGGTAATTCTTCAACTTTGCTTAATCTGTGTTGATCGCCAGTGCCTTATCACAGTAATTTTTTTGTGCGCGGATTATTTGTTGATTATTCTGTATTGGCTTTTTAG

>Hap_24

ATGGTTTTGTTGTTGCGTCGTAATGTGGTTGATTTGCCTACTAATTATTCTCTTAATTATTATTGATGTAGTGGTTTTATGATTTCGGCTTTTATGGTAGTTCAGGTAATCACTGGTGTGGTTCTTTCACTTTTGTATGTGGCTGATTCAAGATTAAGTTTTCGTTGTGTTATGGATTTGAGAAAAGATTCTTTTTTTACTTGGGGAGTGCGCTATTGACACATTTGGGGGGTTAGTATTTTGTTTGTCCTGTTTTTTGTTCATATGGGACGTGCCTTATATTATTCAAGTTATACTAAGAAGGGGGTATGGAAAGTGGGGTTTATTTTATATCTTTTAACTATGGCCGAGGCTTTTTTGGGTTATATTCTACCTTGACATCAAATGTCATATTGGGCTGCTACTGTTTTGACGGCTATTGCCGAGAGTATTCCCTTAGTCGGTCCTACGGTGTTTAAGTATTTGGTGGGGGGTTTTTCTGTAACTAAAGTAACTTTGGTTCGTGTATTTTCAGCTCATGTTTGTTTAGGTTTTGTAATTTTAGGTTTAATGATTTTGCATCTTTTTTATTTGCATTCTTCTGGGTCCAATAACCCTTTATTTTCTTCTTTTGGGTATGGGGATGTTGTTTATTTTCACTCTTATTTCACCACTAAGGATTTTTTTTGTTTGGTTGTTCTATGCTGTATTTTGGTTGGGTTTATGTGGTTGGTTCCTGATTTGGTGGTAGATACGGAGGGTTATCTAGAATCTGATCCTTTGGTGACTCCGGTGTCTATAAAGCCTGAGTGATATGTTTTGATTTATTATGCTATGCTTCGTTCTGTTGAGTCTAAGATAGGTGGTCTAGTGTGGGTGGCTAGATTATTATTTGTTATGTGGGTCCCAACTTTTAAAGACTCTAGTTCATATTTTGTTATTCTACAGGTGATTTTTTGGAGTTTCGTTTGTATTTTTGTTGGGTTGACTTATTTAGGCTCATGTCACCCTGAGTACCCTTATTTGGGTATTTGTCAGTTATTTTCGGTTGGTGCTGTAGCTTTTATGTTTATTTATAAGCTATTTTGATCGAGTTATACTAAGTTGGGTTTTAGTATTTTTTTAGGATAAATGGCTAAGTTTAGTTTTTTTAGTTGGCTTTTTACGCTAGATCATAAGCGTGTGGGTATGATTTATACTTTAATTGGGATATGATCGGGTTTTGTTGGGTTGAGTTTTAGGGTAATGATACGTGTTAATTTTGTTGAGCCTTATTTTAATGTAATTTCTTCGGACTGTTATAAATTTTTGATAACTAATCATGGTATTATTATGATTTTTTTTTTTTTGATGCCCGGGTTGATGGGGGGGTTTGGAAATTATTTGATTCCTTTATTACCCGGTTTACCAAATTTAAATTTACCCCGGTTGAAACCCTTGAAAGCAGGGTGGCTTTTTCCTTCTATTTTATTTCTGGGGTTGAGTAGGGGTTGGGGGGCGGGTAAAGGGGGAACTTTTTACCCCCCCCTCTCTTCTTCCCTTTTTAGCAATAGCCGGGGGGCCAATCTTTTGATGTTTTCTTTACATTTGGCGGGCCTTTCAAGTTTGTGGGGTTCAATAAATTTTATATGTACTCTTTATTCGGCTTTTGTTGATAATTTTGTGTCTCGAAGTTCTATTTTGTTGTGATCTTATTTATTTACTTCTATTCTTTTATGGTTAACTATTCCTGTGTGGGCTGCTGCTATTACAATGTTGTTGTTTGATCGTAAATTTGGTTCGGCTTTTTTTGATCCTTTGGGTGGGGGTGATCCTGTTTTGTTTCAACATATGTTTTGATTTTTTGGGCATCCAGAAGTTTATGTATTGATTTTGCCCGGTTTTGGTATGGTAAGTCATGTGTGTAGTAAATTAGGTTGTTCATATGATACTTTTGGTTTTTATGGTTTACTATTTGCTATGTTTTCTATAGTGTGTTTAGGTAGCGTGGTTTGGGGTCACCACATGTTTACTGTGGGGTTGGACGTGAAGACTGCTGTTTTCTTTAGTTCTGTGACCATGATTATTGGGGTTCCCACGGGTATAAAGGTGTTTTCTTGGCTTTATATGATTTTAAATAGTCGTGTTTCGTTGCGTGAGCCTGTGTTTTGATGGGTTTTATCTTTTATTGTGTTGTTTACTATGGGTGGTGTTACTGGTATAATTCTTTCTGCTTGTGTGTTGGATAAAATTTTGCATGACACGTGGTTTGTGGTGGCTCATTTTCATTATGTTATGTCTTTGGGTTCTTATATTAGGGTTATTATATTTTTTGTTTGGTGATGGCCTGTTATTACAGGGGTTAGCCTGAATAAGTATTTGTTACAGTGTCATTGTATAGTATCAAATGTGGGCTTTAATTTGTGTTTTTTTCCTATGCATTATTTTGGTATTTGTGGTTTACCTCGGCGTGTTTGTGTGTATGAGTCAGGGTACGCTTGAGTTAATATGCTTTGTTCAATAGGTTCTTTTGTTTCTGCCTTTAGTGGTTGCTTTTTTATTTTTATTTTATGGGAGTCTTTAGCTAAAAAGAATGTTGTTATAGGTTATTATGGTAGTTCTTCAACTTTGCTTAATTTGTGTTGATCGCCAGTGCCTTATCACAGTAATTTTTTTGTGCGCGGATTATTTGTTGATTATTCTGTATTGGCTTTTTAG

>Hap_25

ATGGTTTTGTTGTTGCGTCGTAATGTGGTTGATTTGCCTACTAATTATTCTCTTAATTATTATTGATGTAGTGGTTTTATGATTTCGGCTTTTATGGTAGTTCAGGTAATTACTGGTGTGATTCTTTCACTTTTGTATGTGGCTGATTCAAGATTAAGTTTTCGTTGTGTTATGGATTTGAGAAAAGATTCTTTTTTTACTTGGGGGGTGCGCTATTGACACATTTGGGGGGTTAGTATTTTGTTTGTCCTGTTTTTTGTTCATATGGGACGTGCCTTATATTATTCAAGTTATACTAAGAAGGGGGTATGGAAAGTGGGGTTTATTTTATATCTTTTAACTATGGCCGAGGCTTTTTTGGGTTATATTTTACCTTGACATCAAATGTCATATTGGGCTGCTACTGTTTTGACGGCTATTGCCGAGAGTATTCCCTTAGTCGGTCCTACGGTGTTTAAGTATTTGGTGGGGGGGTTTTCTGTAACCAAAGTAACTTTGGTTCGTGTATTTTCAGCTCATGTTTGTTTAGGTTTTGTAATTTTAGGTTTAATGATTTTGCATCTTTTTTATTTGCATTCTTCCGGGTCCAATAACCCTTTATTTTCTTCTTTTGGGTATGGGGATGTTGTTTATTTCCACTCTTATTTCACCACTAAGGATTTTTTTTGTTTGGTCGTTCTATGCTGTATTTTGGTTGGATTTATGTGGTTGGTTCCTGATTTGGTGGTAGATACGGAGGGTTATCTAGAGTCTGATCCTCTGGTGACTCCGGTGTCTATAAAGGCTGAGTGATATTTTTTGATTTATTATGCTATGCTTCCTTCTGTTGAGTCTAAGATAGGTGGTTTAGTGTTGGTGGCTAGATTATTATTTTTTATGTGGGTCCCAACTTTTAAAGACTCTAGTTCATATTTTGTTATTCGACAGGTGGTTTTTGGGAGTTTCGTTTGTCTTTTTGTTGGGTTGACTTATTTAGGCTCATGTCACCCTGAGTACCCTTATTTGGGTATTTGTCAGTTATTTTCGGTTGGTGCTGTAGCTTTTATGTTTATTTATAAGCTATTTTGATCGAGTTATACTAAGTTGGGTTTTAGTATTTTTTTAGGGTAAATGGCTAAGTTTAGTTTTTTTAGTTGGCTTTTTACGCTAGATCATAAGCGTGTGGGTATGATTTATACTTTAATTGGGATATGATCGGGTTTTGTTGGGTTGAGTTTTAGGGTAATGATACGTGTTAATTTTGTTGAGCCTTATTTTAATGTAATTTCTTCGGACTGTTATAAATTTTTGATAACTAATCATGGTATTATTATGATTTTTTTTTTTTTGATGCCCGGGTTGATTGGGGGTTTTGGAAATTATTTGATTCCTTTATTACCCGGTTTACCAAATTTAAATTTCCCCCGGTTGAAACCCTTGAAAGCAGGGTGGCTTTTCCCTCCTATTTTATTTCTGGGGTGGAGTAGGTGTTGGGGGGCGGGAAAAGGGGGGACTTTTTACCCCCCCCTCTCTTCTTCCCTTTTTAGCAATAGCCGGGGGGCCAATCTTTTGATGTTTTCTTTACTTTTGGCGGGTCTTTCAAGTTTGTTGGGTTCTATAAATTTTATATGTACTCTTTATTCGGCTTTTGTGGATAATTTTGTGTCTCGAAGTTCTATTTTGTTGTGATCTTATTTATTTACTTCAATTCTTTTATTGTTAACTATTCCTGTGTTGGCTGCTGCTATTACAATGTTGTTGTTTGATCGTAAATTTGGTTCGGCTTTTTTTGATCCTTTGGGTGGGGGTGATCCCGTTTTGTTTCAACATATGTTTTGGTTTTTTGGGCATCCGGAAGTTTATGTATTGATTTTGCCTGGTTTTGGTATGGTAAGTCATGTGTGTAGTAAATTAGGTTGTTCATATGATACTTTTGGTTTTTATGGTTTACTGTTTGCTATGTTTTCTATAGTGTGTTTAGGTAGCGTGGTTTGGGGCCACCACATGTTTACTGTGGGGTTGGATGTGAAGACTGCTGTTTTCTTTAGTTCTGTGACCATGATTATTGGGGTTCCCACGGGTATAAAGGTGTTTTCTTGGCTTTATATGATTTTAAATAGTCGTGTTTCGTTGCGTGAGCCTGTGTTTTGATGGGTTTTATCTTTTATTGTGTTGTTTACTATGGGTGGTGTTACTGGTATAATTCTTTCTGCTTGTGTGTTGGATAAAATTTTGCATGACACGTGGTTTGTGGTGGCTCATTTTCATTATGTTATGTCTTTGGGTTCTTATATTAGGATTATTATATTTTTTGTTTGGTGATGGCCTGTTATCACAGGGGTTAGCCTGAATAAGTATTTGTTACAGTGTCATTGTATAGTATCAAATGTGGGTTTTAATTTGTGTTTTTTCCCTATGCATTATTTTGGTATTTGTGGTTTACCTCGGCGTGTTTGTGTGTATGAGTCAGGGTACGCTTGAGTTAATATGCTTTGTTCAATAGGTTCTTTTGTTTCTGCCTTTAGTGGTTGCTTTTTTATTTTTATTTTATGGGAGTCTTTAGCTAAAAAGAATGTTGTTATAGGTTATTATGGTAGTTCTTCAACTTTGCTTAATCTGTGTTGGTCGCCAGTGCCTTATCACAGTAATTTTTTTGTGCGCGGATTATTTGTTGATTATTCTGTATTGGCTTTTTAG

>Hap_26

ATGGTTTTGTTGTTGCGTCGTAATGTGGTTGATTTGCCTACTAATTATTCTCTTAATTATTATTGATGTAGTGGTTTTATGATTTCGGCTTTTATGGTAGTTCAGGTAATCACTGGTGTGATTCTTTCACTTTTGTATGTGGCTGATTCAAGATTAAGTTTTCGTTGTGTTATGGATTTGAGAAAAGATTCTTTTTTTACTTGGGGGGTGCGCTATTGACACATTTGGGGGGTTAGTATTTTGTTTGTCCTGTTTTTTGTTCATATGGGACGTGCCTTATATTATTCAAGTTATACTAAGAAGGGGGTATGGAAAGTGGGGTTTATTTTATATCTTTTAACTATGGCCGAGGCTTTTTTGGGTTATATTTTACCTTGACATCAAATGTCATATTGGGCTGCTACTGTTTTGACGGCTATTGCCGAGAGTATTCCCTTAGTCGGTCCTACGGTGTTTAAGTATTTGGTGGGGGGGTTTTCTGTAACCAAAGTAACTTTGGTTCGTGTATTTTCAGCTCATGTTTGTTTAGGTTTTGTAATTTTAGGTTTAATGATTTTGCATCTTTTTTATTTGCATTCTTCCGGGTCCAATAACCCTTTATTTTCTTCTTTTGGGTATGGGGATGTTGTTTATTTCCACTCTTATTTCACCACTAAGGATTTTTTTTGTTTGGTCGTTCTATGCTGTATTTTGGTTGGATTTAGGTGGTTGGTTCCTGATTTGGTGGTAGATACGGAGGGTTATCTAGAGTCTGATCCTCTAGTGACTCCGGTGTCGATAAAGGCTGAGTGATATTTTTTGATTTATTATGCTATGCTTCCTTCTGTTGAGTCTAAGATAGGTGGTTTAGTGCTGGAGGCTAGATTATTATTTTTTATGTGGGTCCCAACTTTTAAAGACTCTACTTCATATTTTGTTATTCGACAGGTGGTTTTTTGGAGGTTCGTTTGTATTTTTGTTGGGTTGACTTATTTAGGCTCATGTCACCCTGAGTACCCTTATTTGGGTATTTGTCAGTTATTTTCGGTTGGTGCTGTAGCTTTTATGTTTATTTATAAGCTATTTTGATCGAGTTATACTAAGTTGGGTTTTAGTATTTTTTTAGGGTAAATGGCTAAGTTTAGTTTTTTTAGTTGGCTTTTTACGCTAGATCATAAGCGTGTGGGTATGATTTATACTTTAATTGGGATATGATCGGGTTTTGTTGGGTTGAGTTTTAGGGTAATGATACGTGTTAATTTTGTTGAGCCTTATTTTAATGTAATTTCTTCGGACTGTTATAAATTTTTGATAACTAATCATGGTATTATTATGATTTTTTTTTTTTTGATGCCCGGGTTGATGGGGGGTTTGGGAAATTATTTAATCCCTTTATTACCCGGTTTACCAAATTTAAATTTACCTCGGTTGAAACCCTTGAAAGCAGGGTGGCTTTTTCCTCCAATTTTATTCCTGGGGTGGAGAAGGGGTTGGGGGGCGGGAAAAGGGGGAACTTTTTACCCCCCCCTCTCTTCTTCCCTTTTTAGCAATAGCCGGGGGGCCAATCTTTTGATGTTTTCTTTACATTTGGCGGGTCTTTCTAGTTTGTTGGGTTCAATAAATTTTATATGTACTCTTTATTCGGCTTTTGTGGATAATTTTGTGTCTCGAAGTTCTATTTTGTTGTGATCTTATTTATTTACTTCAATCCTTTTATTGTTAACTATTCCTGTGTTGGCTGCTGCTATTACAATGTTGTTGTTTGATCGTAAATTTGGTTCGGCTTTTTTTGATCCTTTGGGTGGGGGTGATCCCGTTTTGTTTCAACATATGTTTTGGTTTTTTGGGCATCCGGAAGTTTATGTATTGATTTTGCCTGGTTTTGGTATGGTAAGTCATGTGTGTAGTAAATTAGGTTGTTCATATGATACTTTTGGTTTTTATGGTTTACTGTTTGCTATGTTTTCTATAGTGTGTTTAGGTAGCGTGGTTTGGGGCCACCACATGTTTACTGTGGGGTTGGATGTGAAGACTGCTGTTTTCTTTAGTTCTGTGACCATGATTATTGGGGTTCCCACGGGTATAAAGGTGTTTTCTTGGCTTTATATGATTTTAAATAGTCGTGTTTCGTTGCGTGAGCCTGTGTTTTGATGGGTTTTATCTTTTATTGTGTTGTTTACTATGGGTGGTGTTACTGGTATAATTCTTTCTGCTTGTGTGTTGGATAAAATTTTGCATGACACGTGGTTTGTGGTGGCTCATTTTCATTATGTTATGTCTTTGGGTTCTTATATTAGGATTATTATATTTTTTGTTTGGTGATGGCCTGTTATCACAGGGGTTAGCCTGAATAAGTATTTGTTACAGTGTCATTGTATAGTATCAAATGTGGGTTTTAATTTGTGTTTTTTCCCTATGCATTATTTTGGTATTTGTGGTTTACCTCGGCGTGTTTGTGTGTATGAGTCAGGGTACGCTTGAGTTAATATGCTTTGTTCAATAGGTTCTTTTGTTTCTGCCTTTAGTGGTTGCTTTTTTATTTTTATTTTATGGGAGTCTTTAGCTAAAAAGAATGTTGTTATAGGTTATTATGGTAGTTCTTCAACTTTGCTTAATCTGTGTTGGTCGCCAGTGCCTTATCACAGTAATTTTTTTGTGCGCGGATTATTTGTTGATTATTCTGTATTGGCTTTTTAG

>Hap_27

ATGGTTTTGTTGTTGCGTCGTAATGTGGTTGATTTGCCTACTAATTATTCTCTTAATTATTATTGATGTAGTGGTTTTATGATTTCGGCTTTTATGGTAGTTCAGGTAATCACTGGTGTGATTCTTTCACTTTTGTATGTGGCTGATTCAAGATTAAGTTTTCGTTGTGTTATGGATTTGAGAAAAGATTCTTTTTTTACTTGGGGGGTGCGCTATTGACACATTTGGGGGGTTAGTATTTTGTTTGTCCTGTTTTTTGTTCATATGGGACGTGCCTTATATTATTCAAGTTATACTAAGAAGGGGGTATGGAAAGTGGGGTTTATTTTATATCTTTTAACTATGGCCGAGGCTTTTTTGGGTTATATTTTACCTTGACATCAAATGTCATATTGGGCTGCTACTGTTTTGACGGCTATTGCCGAGAGTATTCCCTTAGTCGGTCCTACGGTGTTTAAGTATTTGGTGGGGGGGTTTTCTGTAACCAAAGTAACTTTGGTTCGTGTATTTTCAGCTCATGTTTGTTTAGGTTTTGTAATTTTAGGTTTAATGATTTTGCATCTTTTTTATTTGCATTCTTCCGGGTCCAATAACCCTTTATTTTCTTCTTTTGGGTATGGGGATGTTGTTTATTTCCACTCTTATTTCACCACTAAGGATTTTTTTTGTTTGGTCGTTCTATGCTGTATTTTGGTTGGATTTATGTGGTTGGTTCCTGATTTGGTGGTAGATACGGAGGGTTATCTAGAGTCTGATCCTCTGGTGACTCCGGTGTCTATAAAGCCTGAGTGATATTTTTTGATTTATTATGCTATGCTTCGTTCTGTTGAGTCTAAGATAGGTGGTTTAGTGTTGGTGGCTAGATTATTATTTTTTATGTGGGTCCCAACTTTTAAAGACTCTAGTTCATATTTTGTTATTCGACAGGTGATTTTTTGGAGTTTCGTTTGTCTTTTTGTTGGGTTGACTTATTTAGCCTCATGTCACCCTGAGTACCCTTATTTGGGTATTTGTCAGTTATTTTCGGTTGGTGCTGTAGCTTTTATGTTTATTTATAAGCTATTTTGATCGAGTTATACTAAGTTGGGTTTTAGTATTTTTTTAGGGTAAATGGCTAAGTTTAGTTTTTTTAGTTGGCTTTTTACGCTAGATCATAAGCGTGTGGGTATGATTTATACTTTAATTGGGATATGATCGGGTTTTGTTGGGTTGAGTTTTAGGGTAATGATACGTGTTAATTTTGTTGAGCCTTATTTTAATGTAATTTCTTCGGACTGTTATAAATTTTTGATAACTAATCATGGTATTATTATGATTTTTTTTTTTTTGATGCCCGGGTTGATTGGGGGTTTTGGAAATTATTTAATCCCTTTATTACCCGGTTTACCAAATTTAAATTTACCCCGGTTGAAACCCTTGAAAGCAGGGTGGCTTTTTCCTCCAATTTTATTTCTGGGGTGGAGAAGGTGTTGGGGTGCGGGAAAAGGGGGGACTTTTTACCCCCCCCTCTCTTCTTCCCTTTTTAGCAATAGCCGGGGGGCCAATCTTTTGATGTTTTCTTTACATTTGGCGGGTCTTTCTAGTTTGTTGGGTTCAATAAATTTTATATGTACTCTTTATTCGGCTTTTGTGGATAATTTTGTGTCTCGAAGTTCTATTTTGTTGTGATCTTATTTATTTACTTCAATCCTTTTATTGTTAACTATTCCTGTGTTGGCTGCTGCTATTACAATGTTGTTGTTTGATCGTAAATTTGGTTCGGCTTTTTTTGATCCTTTGGGTGGGGGTGATCCCGTTTTGTTTCAACATATGTTTTGGTTTTTTGGGCATCCGGAAGTTTATGTATTGATTTTGCCTGGTTTTGGTATGGTAAGTCATGTGTGTAGTAAATTAGGTTGTTCATATGATACTTTTGGTTTTTATGGTTTACTGTTTGCTATGTTTTCTATAGTGTGTTTAGGTAGCGTGGTTTGGGGCCACCACATGTTTACTGTGGGGTTGGATGTGAAGACTGCTGTTTTCTTTAGTTCTGTGACCATGATTATTGGGGTTCCCACGGGTATAAAGGTGTTTTCTTGGCTTTATATGATTTTAAATAGTCGTGTTTCGTTGCGTGAGCCTGTGTTTTGATGGGTTTTATCTTTTATTGTGTTGTTTACTATGGGTGGTGTTACTGGTATAATTCTTTCTGCTTGTGTGTTGGATAAAATTTTGCATGACACGTGGTTTGTGGTGGCTCATTTTCATTATGTTATGTCTTTGGGTTCTTATATTAGGATTATTATATTTTTTGTTTGGTGATGGCCTGTTATCACAGGGGTTAGCCTGAATAAGTATTTGTTACAGTGTCATTGTATAGTATCAAATGTGGGTTTTAATTTGTGTTTTTTCCCTATGCATTATTTTGGTATTTGTGGTTTACCTCGGCGTGTTTGTGTGTATGAGTCAGGGTACGCTTGAGTTAATATGCTTTGTTCAATAGGTTCTTTTGTTTCTGCCTTTAGTGGTTGCTTTTTTATTTTTATTTTATGGGAGTCTTTAGCTAAAAAGAATGTTGTTATAGGTTATTATGGTAGTTCTTCAACTTTGCTTAATCTGTGTTGGTCGCCAGTGCCTTATCACAGTAATTTTTTTGTGCGCGGATTATTTGTTGATTATTCTGTATTGGCTTTTTAG

>Hap_28

ATGGTTTTGTTGTTGCGTCGTAATGTGGTTGATTTGCCTACTAATTATTCTCTTAATTATTATTGATGTAGTGGTTTTATGATTTCGGCTTTTATGGTAGTTCAGGTAATCACTGGTGTGATTCTTTCACTTTTGTATGTGGCTGATTCAAGATTAAGTTTTCGTTGTGTTATGGATTTGAGAAAAGATTCTTTTTTTACTTGGGGAGTGCGCTATTGACACATTTGGGGGGTTAGTATTTTGTTTGTCCTGTTTTTTGTTCATATGGGACGTGCCTTATATTATTCAAGTTATACTAAGAAGGGGGTATGGAAAGTGGGGTTTATTTTATATCTTTTAACTATGGCCGAGGCTTTTTTGGGTTATATTTTACCTTGACATCAAATGTCATATTGGGCTGCTACTGTTTTGACGGCTATTGCCGAGAGTATTCCCTTAGTCGGTCCTACGGTGTTTAAGTATTTGGTGGGGGGGTTTTCTGTAACCAAAGTAACTTTGGTTCGTGTATTTTCAGCTCATGTTTGTTTAGGTTTTGTAATTTTAGGTTTAATGATTTTGCATCTTTTTTATTTGCATTCTTCCGGGTCCAATAACCCTTTATTTTCTTCTTTTGGGTATGGGGATGTTGTTTATTTCCACTCTTATTTCACCACTAAGGATTTTTTTTGTTTGGTCGTTCTATGCTGTATTTTGGTTGGATTTATGTGGTTGGTTCCTGATTTGGTGGTAGATACGGAGGGTTATCTAGAATCTGATCCTCTGGTGACTCCGGTGTCTATAAAGCCTGAGTGATATTTTTTGATTTATTATGCTATGCTTCGTTCTGTTGAGTCTAAGATAGGTGGTCTAGTGTTGGTGGCTAGATTATTATTTTTTATGTGGGTCCCAACTTTTAAAGACTCTAGTTCATATTTTGTTATTCGACAGGTGATTTTTTGGAGTTTCGTTTGTCTTTTTGTTGGGTTGACTTATTTAGGCTCATGTCACCCTGAGTACCCTTATTTGGGTATTTGTCAGTTATTTTCGGTTGGTGCTGTAGCTTTTATGTTTATTTATAAGCTATTTTGATCGAGTTATACTAAGTTGGGTTTTAGTATTTTTTTAGGGTAAATGGCTAAGTTTAGTTTTTTTAGTTGGCTTTTTACGCTAGATCATAAGCGTGTGGGTATGATTTATACTTTAATTGGGATATGATCGGGTTTTGTTGGGTTGAGTTTTAGGGTAATGATACGTGTTAATTTTGTTGAGCCTTATTTTAATGTAATTTCTTCGGACTGTTATAAATTTTTGATAACTAATCATGGTATTATTATGATTTTTTTTTTTTTGATGCCCGGGTTGATGGGGGGTTTGGGAAATTATTTAATCCCTTTATTACCCGGTTTACCAAATTTAAATTTACCCCGGTTGAAACCCTTGAAAGCAGGGTGGCTTTTTCCTCCAATTTTATTCCTGGGGTGGAGAAGGGGTTGGGGGGCGGGAAAAGGGGGGACTTTTTACCCCCCCCTCTCTTCTTCCCTTTTTAGCAATAGCCGGGGGGCCAATCTTTTGATGTTTTCTTTACATTTGGCGGGTCTTTCTAGTTTGTTGGGTTCAATAAATTTTATATGTACTCTTTATTCGGCTTTTGTGGATAATTTTGTGTCTCGAAGTTCTATTTTGTTGTGATCTTATTTATTTACTTCAATCCTTTTATTGTTAACTATTCCTGTGTTGGCTGCTGCTATTACAATGTTGTTGTTTGATCGTAAATTTGGTTCGGCTTTTTTTGATCCTTTGGGTGGGGGTGATCCCGTTTTGTTTCAACATATGTTTTGGTTTTTTGGGCATCCGGAAGTTTATGTATTGATTTTGCCTGGTTTTGGTATGGTAAGTCATGTGTGTAGTAAATTAGGTTGTTCATATGATACTTTTGGTTTTTATGGTTTACTGTTTGCTATGTTTTCTATAGTGTGTTTAGGTAGCGTGGTTTGGGGCCACCACATGTTTACTGTGGGGTTGGATGTGAAGACTGCTGTTTTCTTTAGTTCTGTGACCATGATTATTGGGGTTCCCACGGGTATAAAGGTGTTTTCTTGGCTTTATATGATTTTAAATAGTCGTGTTTCGTTGCGTGAGCCTGTGTTTTGATGGGTTTTATCTTTTATTGTGTTGTTTACTATGGGTGGTGTTACTGGTATAATTCTTTCTGCTTGTGTGTTGGATAAAATTTTGCATGACACGTGGTTTGTGGTGGCTCATTTTCATTATGTTATGTCTTTGGGTTCTTATATTAGGGTTATTATATTTTTTGTTTGGTGATGGCCTGTTATCACAGGGGTTAGCTTGAATAAGTATTTGTTACAGTGTCATTGTATAGTATCAAATGTGGGCTTTAATTTGTGTTTTTTTCCTATGCATTATTTTGGTATTTGTGGTTTACCTCGGCGTGTTTGTGTGTATGAGTCAGGGTACGCTTGAGTTAATATGCTTTGTTCAATAGGTTCTTTTGTTTCTGCCTTTAGTGGTTGCTTTTTTATTTTTATTTTATGGGAGTCTTTAGCTAAAAAGAATGTTGTTATAGGTTATTATGGTAGTTCTTCAACTTTGCTTAATTTGTGTTGATCGCCAGTGCCTTACCACAGTAATTTTTTTGTGCGTGGATTATTTGTTGATTATTCTGTATTGGCTTTTTAG

>Hap_29

ATGGTTTTGTTGTTGCGTCGTAATGTGGTTGATTTGCCTACTAATTATTCTCTTAATTATTATTGGTGTAGCGGGTTTATGATTTCGGCTTTTATGGTAGTTCAGGTAATTACTGGTGTGATTCTTTCACTTTTGTATGTGGCTGATTCAAGATTAAGTTTTCGTTGTGTTATGGATTTGAGAAAAGATTCTTTTTTTACTTGGGGGGTGCGTTATTGACACATCTGGGGGGTTAGTATTTTGTTTGTCCTGTTTTTTGTTCATATGGGTCGTGCCTTATATTATTCAAGTTATACTAAGAAGGGGGTATGGAAAGTGGGGTTTATTTTATATCTTTTAACTATGGCTGAGGCTTTTTTGGGTTATATTTTACCTTGACATCAAATGTCATATTGGGCTGCTACTGTTTTGACGGCTATTGCCGAGAGTATTCCCTTAGTTGGTCCTACGGTGTTTAAGTATTTGGTGGGGGGGTTTTCTGTAACTAAAGTAACTTTGGTTCGTGTATTTTCAGCTCATGTTTGTTTAGGTTTTGTAATTTTAGGTTTAATGATTTTGCATCTTTTTTATTTGCATTCTTCCGGGTCTAATAACCCTTTATTTTCTTCTTTTGGGTATGGAGATGTTGTTTATTTCCACTCTTATTTTACCACTAAGGATTTTTTTTGTTTGGTTGTTTTATGCTGTATTTTGGTTGGATTTAGGTGGTTGGTTCCTGATTTGGTGGTAGATACGGAGGGTTATCTAGAGTCTGATCCTTTAGTGACTCCGGTGTCTATAAAGCCTGAGTGATATTTTATGATTTATTATGCTATGCTTCGTTCTGTTGAGTCTAAGATAGGTGGTTTAGTGCTGGAGGCTAGATTATTATTTTTTATGTGGGTCCCAACTTTTAAAGATTCTAGTTCATATTTTGTTATTCGACAGGTGGTTTTTTGGGGTTTCGTTTGTCTTTTTGTTGGGTTGACTTATTTAGGCTCATGTCACCCTGAGTATCCTTATTTGGGTATTTGTCAGTTATTTTCGGTTGGTGCTGTAGCTTTTATGTTTATTTATAAGCTATTTTGATCGAGTTATACTAAGTTGGGTTTTAGTATTTTTTTAGGGTAAATGGCTAAGTTTAGTTTTTTTAGTTGGCTTTTTACGCTAGATCATAAGCGTGTGGGTATGATTTATACTTTAATTGGGATATGATCAGGTTTTGTTGGGTTGAGTTTTAGGGTAATGATACGTGTTAATTTCGTTGAGCCTTATTTTAATGTAATTTCTTCGGACTGTTATAAATTTTTGATAACTAATCATGGTATTATTATGATTTTTTTTTTTTTGAGGCCGGGGTTGATGGGGGGTTTTGGAAATTATTTAATCCCTTTATTACCCGGTTTACCAAATTTAAATTTCCCCCGGTTGAAACCCTTGAAAGCAGGGTGGCTTTTTCCTCCAATTTTATTCCTGGGGCGAAGTATGTGTTGGGGGGCGGGTATAGGGGGAACTTTTTACCCCCCTCTCTCCCCTTCTCTTTTTAGGGATAGCCGGGGGGTTAATCTTTTGATGTTTTCTTTACTTTTGGCGGGTCTTTCAAGTTTGTTGGGTTCAATAAATTTTATATGTACTCTTTACTCAGCTTTTGTTGATAATTTTGTGTCTCGTAGTTCTATTTTGTTGTGGTCTTATCTATTTACTTCTATTCTTTTATTGTTAACTATTCCTGTGTGGGCTGCTGCCATTACAATGTTGTTGTTTGATCGTAAATTTGGTTCGGCTTTTTTTGATCCTTTGGGTGGTGGTGATCCTGTTTTGTTTCAACATATGTTTTGGTTTTTTGGGCATCCAGAAGTTTATGTATTGATTTTGCCTGGTTTTGGTATGGTAAGTCATGTGTGTAGTAAATTAGGTTGTTCATATGATACTTTTGGTTTTTATGGTTTATTATTTGCTATGTTTTCTATAGTGTGTTTAGGTAGCGTGGTTTGGGGTCACCACATGTTTACTGTGGGGTTGGACGTGAAGACTGCTGTTTTCTTTAGTTCTGTGACTATGATTATTGGGGTTCCCACGGGTATAAAGGTGTTTTCTTGGCTTTATATGATTTTAAATAGTCGTGTTTCGTTGCGTGAGCCTGTGTTTTGATGGGTTTTATCCTTTATTGTGTTGTTTACTATGGGTGGTGTTACTGGTATAATTCTTTCTGCTTGTGTGTTGGATAAAATTTTGCATGACACGTGGTTTGTGGTGGCTCATTTTCATTATGTTATGTCTTTGGGTTCTTATATTAGGGTTATTATATTTTTTGTTTGGTGATGGCCTGTTATCACAGGGGTTAGCTTGAATAAGTATTTGTTACAGTGTCATTGTATAGTATCAAATGTGGGCTTTAATTTGTGTTTTTTTCCTATGCATTATTTTGGTATTTGTGGTTTACCTCGGCGTGTTTGTGTGTATGAGTCAGGGTACGCTTGAGTTAATATGCTTTGTTCAATAGGTTCTTTTGTTTCTGCCTTTAGTGGTTGCTTTTTTATTTTTATTTTATGGGAGTCTTTAGCTAAAAAGAATGTTGTTATAGGTTATTATGGTAGTTCTTCAACTTTGCTTAATTTGTGTTGATCGCCAGTGCCTTACCACAGTAATTTTTTTGTGCGTGGATTATTTGTTGATTATTCTGTATTGGCTTTTTAG

>Hap_30

ATGGTTTTGTTGTTGCGTCGTAATGTGGTTGATTTGCCTACTAATTATTCTCTTAATTATTATTGGTGTAGCGGGTTTATGATTTCGGCTTTTATGGTAGTTCAGGTAATTACTGGTGTGATTCTTTCACTTTTGTATGTGGCTGATTCAAGATTAAGTTTTCGTTGTGTTATGGATTTGAGAAAAGATTCTTTTTTTACTTGGGGGGTGCGTTATTGACACATCTGGGGGGTTAGTATTTTGTTTGTCCTGTTTTTTGTTCATATGGGTCGTGCCTTATATTATTCAAGTTATACTAAGAAGGGGGTATGGAAAGTGGGGTTTATTTTATATCTTTTAACTATGGCTGAGGCTTTTTTGGGTTATATTTTACCTTGACATCAAATGTCATATTGGGCTGCTACTGTTTTGACGGCTATTGCCGAGAGTATTCCCTTAGTTGGTCCTACGGTGTTTAAGTATTTGGTGGGGGGGTTTTCTGTAACTAAAGTAACTTTGGTTCGTGTATTTTCAGCTCATGTTTGTTTAGGTTTTGTAATTTTAGGTTTAATGATTTTGCATCTTTTTTATTTGCATTCTTCCGGGTCTAATAACCCTTTATTTTCTTCTTTTGGGTATGGAGATGTTGTTTATTTCCACTCTTATTTTACCACTAAGGATTTTTTTTGTTTGGTTGTTTTATGCTGTATTTTGGTTGGATTTATGTGGTTGGTTCCTGATTTGGTGGTAGATACAGAGGGTTATCTAGAGTCTGATCCTTTGGTGACTCCGGTGTCTATAAAGCCTGAGTGATATTTTTTGATTTATTATGCTATGCTTCGTTCTGTTGAGTCTAAGATAGGTGGTTTAGTGTTGGTGGCTAGATTATTATTTTTTATGTGGGTCCCAACTTTTAAAGACTCTAGTTCATATTTTGTTATTCGACAGGTGGTTTTTTGGGGTTTCGTTTGTCTTTTTGGTGGGTTGACTTATTTAGCCTCATGTCACCCTGAGTATCCTTATTTGGGTATTTGTCAGTTATTTTCGGTTGGTGCTGTAGCTTTTATGTTTATTTATAAGCTATTTTGATCGAGTTATACTAAGTTGGGTTTTAGTATTTTTTTAGGGTAAATGGCTAAGTTTAGTTTTTTTAGTTGGCTTTTTACGCTAGATCATAAGCGTGTGGGTATGATTTATACTTTAATTGGGATATGATCAGGTTTTGTTGGGTTGAGTTTTAGGGTAATGATACGTGTTAATTTCGTTGAGCCTTATTTTAATGTAATTTCTTCGGACTGTTATAAATTTTTGATAACTAATCATGGTATTATTATGATTTTTTTTTTTTTGATGCCGGGGTTGATGGGGGGTTTGGGAAATTATTTGATCCCTTTATTACCCGGTTTACCAAATTTAAATTTCCCCCGGTTGAAACCCTTGAAAGCAGGGTGGCTTTTTCCTTCTATTTTATTTCTGGGGCGAAGTATGGGTTGGGGGGCGGGAAAAGGGGGGACTTTTTACCCCCCTCTCTCCCCTTCCCTTTTTAGGGATAGCCGGGGGGTTAATCTTTTAATGTTTTCTTTACATTTGGCGGGTCTTTCAAGTTTGTTGGGTTCAATAAATTTTATATGTACTCTTTACTCAGCTTTTGTTGATAATTTTGTGTCTCGTAGTTCTATTTTGTTGTGGTCTTATCTATTTACTTCTATTCTTTTATTGTTAACTATTCCTGTGTGGGCTGCTGCCATTACAATGTTGTTGTTTGATCGTAAATTTGGTTCGGCTTTTTTTGATCCTTTGGGTGGTGGTGATCCTGTTTTGTTTCAACATATGTTTTGGTTTTTTGGGCATCCAGAAGTTTATGTATTGATTTTGCCTGGTTTTGGTATGGTAAGTCATGTGTGTAGTAAATTAGGTTGTTCATATGATACTTTTGGTTTTTATGGTTTATTATTTGCTATGTTTTCTATAGTGTGTTTAGGTAGCGTGGTTTGGGGTCACCACATGTTTACTGTGGGGTTGGACGTGAAGACTGCTGTTTTCTTTAGTTCTGTGACTATGATTATTGGGGTTCCCACGGGTATAAAGGTGTTTTCTTGGCTTTATATGATTTTAAATAGTCGTGTTTCGTTGCGTGAGCCTGTGTTTTGATGGGTTTTATCCTTTATTGTGTTGTTTACTATGGGTGGTGTTACTGGTATAATTCTTTCTGCTTGTGTGTTGGATAAAATTTTGCATGACACGTGGTTTGTGGTGGCTCATTTTCATTATGTTATGTCTTTGGGTTCTTATATTAGGGTTATTATATTTTTTGTTTGGTGATGGCCTGTTATCACAGGGGTTAGCTTGAATAAGTATTTGTTACAGTGTCATTGTATAGTATCAAATGTGGGCTTTAATTTGTGTTTTTTTCCTATGCATTATTTTGGTATTTGTGGTTTACCTCGGCGTGTTTGTGTGTATGAGTCAGGGTACGCTTGAGTTAATATGCTTTGTTCAATAGGTTCTTTTGTTTCTGCCTTTAGTGGTTGCTTTTTTATTTTTATTTTATGGGAGTCTTTAGCTAAAAAGAATGTTGTTATAGGTTATTATGGTAGTTCTTCAACTTTGCTTAATTTGTGTTGATCGCCAGTGCCTTACCACAGTAATTTTTTTGTGCGTGGATTATTTGTTGATTATTCTGTATTGGCTTTTTAG

>Hap_31

ATGGTTTTGTTGTTGCGTCGTAATGTGGTTGATTTGCCTACTAATTATTCTCTTAATTATTATTGGTGTAGCGGGTTTATGATTTCGGCTTTTATGGTAGTTCAGGTAATTACTGGTGTGATTCTTTCACTTTTGTATGTGGCTGATTCAAGATTAAGTTTTCGTTGTGTTATGGATTTGAGAAAAGATTCTTTTTTTACTTGGGGGGTGCGTTATTGACACATCTGGGGGGTTAGTATTTTGTTTGTCCTGTTTTTTGTTCATATGGGTCGTGCCTTATATTATTCAAGTTATACTAAGAAGGGGGTATGGAAAGTGGGGTTTATTTTATATCTTTTAACTATGGCTGAGGCTTTTTTGGGTTATATTTTACCTTGACATCAAATGTCATATTGGGCTGCTACTGTTTTGACGGCTATTGCCGAGAGTATTCCCTTAGTTGGTCCTACGGTGTTTAAGTATTTGGTGGGGGGGTTTTCTGTAACTAAAGTAACTTTGGTTCGTGTATTTTCAGCTCATGTTTGTTTAGGTTTTGTAATTTTAGGTTTAATGATTTTGCATCTTTTTTATTTGCATTCTTCCGGGTCTAATAACCCTTTATTTTCTTCTTTTGGGTATGGAGATGTTGTTTATTTCCACTCTTATTTTACCACTAAGGATTTTTTTTGTTTGGTTGTTTTATGCTGTATTTTGGTTGGATTTATGTGGTTGGTTCCTGATTTGGTGGTAGATACAGAGGGTTATCTAGAGTCTGATCCTTTGGTGACTCCGGTGTCTATAAAGCCTGAGTGATATTTTTTGATTTATTATGCTATGCTTCGTTCTGTTGAGTCTAAGATAGGTGGTTTAGTGTTGGTGGCTAGATTATTATTTTTTATGTGGGTCCCAACTTTTAAAGATTCTAGTTCATATTTTGTTATTCGACAGGTGGTTTTTTGGGGTTTCGTTTGTCTTTTTGTTGGGTTGACTTATTTAGCCTCATGTCACCCTGAGTATCCTTATTTGGGTATTTGTCAGTTATTTTCGGTTGGTGCTGTAGCTTTTATGTTTATTTATAAGCTATTTTGATCGAGTTATACTAAGTTGGGTTTTAGTATTTTTTTAGGGTAAATGGCTAAGTTTAGTTTTTTTAGTTGGCTTTTTACGCTAGATCATAAGCGTGTGGGTATGATTTATACTTTAATTGGGATATGATCAGGTTTTGTTGGGTTGAGTTTTAGGGTAATGATACGTGTTAATTTCGTTGAGCCTTATTTTAATGTAATTTCTTCGGACTGTTATAAATTTTTGATAACTAATCATGGTATTATTATGATTTTTTTTTTTTTGAGGCCGGGGTTGATTGGGGGTTTGGGAAATTATTTAATCCCTTTATTACCCGGTTTACCAAATTTAAATTTACCCCGGTTGAAACCCTTGAAAGCAGGGTGGCTTTTTCCTTCTATTTTATTTCTGGGGCTAAAAATGGGTTGGGGTGCTGGAAAAGGGGGAACTTTTTACCCCCCTCTCTCCCCTTCCCTTTTTAGGGATAGCCGGGGGGTTAATCTTTTAATGTTTTCTTTACATTTGGCGGGTCTTTCAAGTTTGTTGGGTTCAATAAATTTTATATGTACTCTTTACTCAGCTTTTGTTGATAATTTTGTGTCTCGTAGTTCTATTTTGTTGTGGTCTTATCTATTTACTTCTATTCTTTTATTGTTAACTATTCCTGTGTGGGCTGCTGCCATTACAATGTTGTTGTTTGATCGTAAATTTGGTTCGGCTTTTTTTGATCCTTTGGGTGGTGGTGATCCTGTTTTGTTTCAACATATGTTTTGGTTTTTTGGGCATCCAGAAGTTTATGTATTGATTTTGCCTGGTTTTGGTATGGTAAGTCATGTGTGTAGTAAATTAGGTTGTTCATATGATACTTTTGGTTTTTATGGTTTATTATTTGCTATGTTTTCTATAGTGTGTTTAGGTAGCGTGGTTTGGGGTCACCACATGTTTACTGTGGGGTTGGACGTGAAGACTGCTGTTTTCTTTAGTTCTGTGACTATGATTATTGGGGTTCCCACGGGTATAAAGGTGTTTTCTTGGCTTTATATGATTTTAAATAGTCGTGTTTCGTTGCGTGAGCCTGTGTTTTGATGGGTTTTATCCTTTATTGTGTTGTTTACTATGGGTGGTGTTACTGGTATAATTCTTTCTGCTTGTGTGTTGGATAAAATTTTGCATGACACGTGGTTTGTGGTGGCTCATTTTCATTATGTTATGTCTTTGGGTTCTTATATTAGGGTTATTATATTTTTTGTTTGGTGATGGCCTGTTATCACAGGGGTTAGCTTGAATAAGTATTTGTTACAGTGTCATTGTATAGTATCAAATGTGGGCTTTAATTTGTGTTTTTTTCCTATGCATTATTTTGGTATTTGTGGTTTACCTCGGCGTGTTTGTGTGTATGAGTCAGGGTACGCTTGAGTTAATATGCTTTGTTCAATAGGTTCTTTTGTTTCTGCCTTTAGTGGTTGCTTTTTTATTTTTATTTTATGGGAGTCTTTAGCTAAAAAGAATGTTGTTATAGGTTATTATGGTAGTTCTTCAACTTTGCTTAATTTGTGTTGATCGCCAGTGCCTTACCACAGTAATTTTTTTGTGCGTGGATTATTTGTTGATTATTCTGTATTGGCTTTTTAG

>Hap_32

ATGGTTTTGTTGTTGCGTCGTAATGTGGTTGATTTGCCTACTAATTATTCTCTTAATTATTATTGGTGTAGCGGGTTTATGATTTCGGCTTTTATGGTAGTTCAGGTAATTACTGGTGTGATTCTTTCACTTTTGTATGTGGCTGATTCAAGATTAAGTTTTCGTTGTGTTATGGATTTGAGAAAAGATTCTTTTTTTACTTGGGGGGTGCGTTATTGACACATCTGGGGGGTTAGTATTTTGTTTGTCCTGTTTTTTGTTCATATGGGTCGTGCCTTATATTATTCAAGTTATACTAAGAAGGGGGTATGGAAAGTGGGGTTTATTTTATATCTTTTAACTATGGCTGAGGCTTTTTTGGGTTATATTTTACCTTGACATCAAATGTCATATTGGGCTGCTACTGTTTTGACGGCTATTGCCGAGAGTATTCCCTTAGTTGGTCCTACGGTGTTTAAGTATTTGGTGGGGGGGTTTTCTGTAACTAAAGTAACTTTGGTTCGTGTATTTTCAGCTCATGTTTGTTTAGGTTTTGTAATTTTAGGTTTAATGATTTTGCATCTTTTTTATTTGCATTCTTCCGGGTCTAATAACCCTTTATTTTCTTCTTTTGGGTATGGAGATGTTGTTTATTTCCACTCTTATTTTACCACTAAGGATTTTTTTTGTTTGGTTGTTTTATGCTGTATTTTGGTTGGATTTATGTGGTTGGTTCCTGATTTGGTGGTAGATACAGAGGGTTATCTAGAGTCTGATCCTTTGGTGACTCCGGTGTCTATAAAGCCTGAGTGATATTTTTTGATTTATTATGCTATGCTTCGTTCTGTTGAGTCTAAGATAGGTGGTTTAGTGTTGGTGGCTAGATTATTATTTTTTATGTGGGTCCCAACTTTTAAAGACTCTAGTTCATATTTTGTTATTCGACAGGTGGTTTTTTGGGGTTTCGTTTGTCTTTTTGTTGGGTTGACTTATTTAGCCTCATGTCACCCTGAGTATCCTTATTTGGGTATTTGTCAGTTATTTTCGGTTGGTGCTGTAGCTTTTATGTTTATTTATAAGCTATTTTGATCGAGTTATACTAAGTTGGGTTTTAGTATTTTTTTAGGGTAAATGGCTAAGTTTAGTTTTTTTAGTTGGCTTTTTACGCTAGATCATAAGCGTGTGGGTATGATTTATACTTTAATTGGGATATGATCAGGTTTTGTTGGGTTGAGTTTTAGGGTAATGATACGTGTTAATTTCGTTGAGCCTTATTTTAATGTAATTTCTTCGGACTGTTATAAATTTTTGATAACTAATCATGGTATTATTATGATTTTTTTTTTTTTGATCCCGGGGTTGATGGGGGGTTTTGGAAATTATTTGATCCCTTTATTACCCGGTTTACCAAATTTAAATTTCCCCCGGTTGAAACCCTTGAAAGCAGGGTGGCTTTTTCCTTCTATTTTATTTCTGGGGCTAAAAATGGGTTGGGGGGCGGGAAAAGGGGGAACTTTTTACCCCCCTCTCTCCCCTTCCCTTTTTAGGGATAGCCGGGGGGTTAATCTTTTGATGTTTTCTTTACATTTGGCGGGTCTTTCAAGTTTGTTGGGTTCTATAAATTTTATATGTACTCTTTACTCAGCTTTTGTTGATAATTTTGTGTCTCGTAGTTCTATTTTGTTGTGGTCTTATCTATTTACTTCTATTCTTTTATTGTTAACTATTCCTGTGTGGGCTGCTGCCATTACAATGTTGTTGTTTGATCGTAAATTTGGTTCGGCTTTTTTTGATCCTTTGGGTGGTGGTGATCCTGTTTTGTTTCAACATATGTTTTGGTTTTTTGGGCATCCAGAAGTTTATGTATTGATTTTGCCTGGTTTTGGTATGGTAAGTCATGTGTGTAGTAAATTAGGTTGTTCATATGATACTTTTGGTTTTTATGGTTTATTATTTGCTATGTTTTCTATAGTGTGTTTAGGTAGCGTGGTTTGGGGTCACCACATGTTTACTGTGGGGTTGGACGTGAAGACTGCTGTTTTCTTTAGTTCTGTGACTATGATTATTGGGGTTCCCACGGGTATAAAGGTGTTTTCTTGGCTTTATATGATTTTAAATAGTCGTGTTTCGTTGCGTGAGCCTGTGTTTTGATGGGTTTTATCCTTTATTGTGTTGTTTACTATGGGTGGTGTTACTGGTATAATTCTTTCTGCTTGTGTGTTGGATAAAATTTTGCATGACACGTGGTTTGTGGTGGCTCATTTTCATTATGTTATGTCTTTGGGTTCTTATATTAGGGTTATTATATTTTTTGTTTGGTGATGGCCTGTTATCACAGGGGTTAGCTTGAATAAGTATTTGTTACAGTGTCATTGTATAGTATCAAATGTGGGCTTTAATTTGTGTTTTTTTCCTATGCATTATTTTGGTATTTGTGGTTTACCTCGGCGTGTTTGTGTGTATGAGTCAGGGTACGCTTGAGTTAATATGCTTTGTTCAATAGGTTCTTTTGTTTCTGCCTTTAGTGGTTGCTTTTTTATTTTTATTTTATGGGAGTCTTTAGCTAAAAAGAATGTTGTTATAGGTTATTATGGTAGTTCTTCAACTTTGCTTAATTTGTGTTGATCGCCAGTGCCTTACCACAGTAATTTTTTTGTGCGTGGATTATTTGTTGATTATTCTGTATTGGCTTTTTAG

>Hap_33

ATGGTTTTGTTGTTGCGTCGTAATGTGGTTGATTTGCCTACTAATTATTCTCTTAATTATTATTGGTGTAGCGGGTTTATGATTTCGGCTTTTATGGTAGTTCAGGTAATTACTGGTGTGATTCTTTCACTTTTGTATGTGGCTGATTCAAGATTAAGTTTTCGTTGTGTTATGGATTTGAGAAAAGATTCTTTTTTTACTTGGGGGGTGCGTTATTGACACATCTGGGGGGTTAGTATTTTGTTTGTCCTGTTTTTTGTTCATATGGGTCGTGCCTTATATTATTCAAGTTATACTAAGAAGGGGGTATGGAAAGTGGGGTTTATTTTATATCTTTTAACTATGGCTGAGGCTTTTTTGGGTTATATTTTACCTTGACATCAAATGTCATATTGGGCTGCTACTGTTTTGACGGCTATTGCCGAGAGTATTCCCTTAGTTGGTCCTACGGTGTTTAAGTATTTGGTGGGGGGGTTTTCTGTAACTAAAGTAACTTTGGTTCGTGTATTTTCAGCTCATGTTTGTTTAGGTTTTGTAATTTTAGGTTTAATGATTTTGCATCTTTTTTATTTGCATTCTTCCGGGTCTAATAACCCTTTATTTTCTTCTTTTGGGTATGGAGATGTTGTTTATTTCCACTCTTATTTTACCACTAAGGATTTTTTTTGTTTGGTTGTTTTATGCTGTATTTTGGTTGGATTTATGTGGTTGGTTCCTGATTTGGTGGTAGATACAGAGGGTTATCTAGAGTCTGATCCTTTGGTGACTCCGGTGTCTATAAAGCCTGAGTGATATTTTTTGATTTATTATGCTATGCTTCGTTCTGTTGAGTCTAAGATAGGTGGTTTAGTGTTGGTTGCTAGATTATTATTTTTTATGTGGGTCCCAACTTTTAAAGACTCTAGTTCATATTTTGTTATTCGACAGGTGGTTTTTTGGGGTTTCGTTTGTCTTTTTGTTGGGTTGACTTATTTAGGCTCATGTCACCCTGAGTATCCTTATTTGGGTATTTGTCAGTTATTTTCGGTTGGTGCTGTAGCTTTTATGTTTATTTATAAGCTATTTTGATCGAGTTATACTAAGTTGGGTTTTAGTATTTTTTTAGGGTAAATGGCTAAGTTTAGTTTTTTTAGTTGGCTTTTTACGCTAGATCATAAGCGTGTGGGTATGATTTATACTTTAATTGGGATATGATCAGGTTTTGTTGGGTTGAGTTTTAGGGTAATGATACGTGTTAATTTCGTTGAGCCTTATTTTAATGTAATTTCTTCGGACTGTTATAAATTTTTGATAACTAATCATGGTATTATTATGATTTTTTTTTTTTTGAGGCCGGGGTTGATTGGGGGTTTGGGAAATTATTTAATCCCTTTATTACCCGGTTTACCAAATTTAAATTTCCCCCGGTTGAAACCCTTGAAAGCAGGGTGGCTTTTTCCTTCTATTTTATTTCTGGGGCGAAGTATGGGTTGGGGGGCGGGAAAAGGGGGAACTTTTTACCCCCCTCTCTCCCCTTCCCTTTTTAGGGATAGCCGGGGGGTTAATCTTTTAATGTTTTCTTTACATTTGGCGGGTCTTTCAAGTTTGTTGGGTTCAATAAATTTTATATGTACTCTTTACTCAGCTTTTGTTGATAATTTTGTGTCTCGTAGTTCTATTTTGTTGTGGTCTTATCTATTTACTTCTATTCTTTTATTGTTAACTATTCCTGTGTGGGCTGCTGCCATTACAATGTTGTTGTTTGATCGTAAATTTGGTTCGGCTTTTTTTGATCCTTTGGGTGGTGGTGATCCTGTTTTGTTTCAACATATGTTTTGGTTTTTTGGGCATCCAGAAGTTTATGTATTGATTTTGCCTGGTTTTGGTATGGTAAGTCATGTGTGTAGTAAATTAGGTTGTTCATATGATACTTTTGGTTTTTATGGTTTATTATTTGCTATGTTTTCTATAGTGTGTTTAGGTAGCGTGGTTTGGGGTCACCACATGTTTACTGTGGGGTTGGATGTGAAGACTGCTGTTTTCTTTAGTTCTGTGACTATGATTATTGGGGTTCCCACGGGTATAAAGGTGTTTTCTTGGCTTTATATGATTTTAAATAGTCGTGTTTCGTTGCGTGAGCCTGTGTTTTGATGGGTTTTATCTTTTATTGTGTTGTTTACTATGGGTGGTGTTACTGGTATAATTCTTTCTGCTTGTGTGTTGGATAAAATTTTGCATGACACGTGGTTTGTGGTGGCTCATTTTCATTATGTTATGTCTTTGGGTTCTTATATTAGGATTATTATATTTTTTGTTTGGTGATGGCCTGTTATCACAGGGGTTAGCCTGAATAAGTATTTGTTACAGTGTCATTGTATAGTATCAAATGTGGGTTTTAATTTGTGTTTTTTCCCTATGCATTATTTTGGTATTTGTGGTTTACCTCGGCGTGTTTGTGTGTATGAGTCAGGGTACGCTTGAGTTAATATGCTTTGTTCAATAGGTTCTTTTGTTTCTGCCTTTAGTGGTTGCTTTTTTATTTTTATTTTATGGGAGTCTTTAGCTAAAAAGAATGTTGTTATAGGTTATTATGGTAGTTCTTCAACTTTGCTTAATTTGTGTTGATCGCCAGTGCCTTACCACAGTAATTTTTTTGTGCGTGGATTATTTGTTGATTATTCTGTATTGGCTTTTTAG

>Hap_34

ATGGTTTTGTTGTTGCGTCGTAATGTGGTTGATTTGCCTACTAATTATTCTCTTAATTATTATTGGTGTAGCGGGTTTATGATTTCGGCTTTTATGGTAGTTCAGGTAATTACTGGTGTGATTCTTTCACTTTTGTATGTGGCTGATTCAAGATTAAGTTTTCGTTGTGTTATGGATTTGAGAAAAGATTCTTTTTTTACTTGGGGGGTGCGTTATTGACACATCTGGGGGGTTAGTATTTTGTTTGTCCTGTTTTTTGTTCATATGGGTCGTGCCTTATATTATTCAAGTTATACTAAGAAGGGGGTATGGAAAGTGGGGTTTATTTTATATCTTTTAACTATGGCTGAGGCTTTTTTGGGTTATATTTTACCTTGACATCAAATGTCATATTGGGCTGCTACTGTTTTGACGGCTATTGCCGAGAGTATTCCCTTAGTTGGTCCTACGGTGTTTAAGTATTTGGTGGGGGGGTTTTCTGTAACTAAAGTAACTTTGGTTCGTGTATTTTCAGCTCATGTTTGTTTAGGTTTTGTAATTTTAGGTTTAATGATTTTGCATCTTTTTTATTTGCATTCTTCCGGGTCTAATAACCCTTTATTTTCTTCTTTTGGGTATGGAGATGTTGTTTATTTCCACTCTTATTTTACCACTAAGGATTTTTTTTGTTTGGTTGTTTTATGCTGTATTTTGGTTGGATTTATGTGGTTGGTTCCTGATTTGGTGGTAGATACAGAGGGTTATCTAGAGTCTGATCCTTTGGTGACTCCGGTGTCTATAAAGCCTGAGTGATATTTTTTGATTTATTATGCTATGCTTCGTTCTGTTGAGTCTAAGATAGGTGGTTTAGTGTTGGTGGCTAGATTATTATTTTTTATGTGGGTCCCAACTTTTAAAGATTCTAGTTCATATTTTGTTATTCGACAGGTGGTTTTTTGGGGTTTCGTTTGTCTTTTTGTTGGGTTGACTTATTTAGGCTCATGTCACCCTGAGTATCCTTATTTGGGTATTTGTCAGTTATTTTCGGTTGGTGCTGTAGCTTTTATGTTTATTTATAAGCTATTTTGATCGAGTTATACTAAGTTGGGTTTTAGTATTTTTTTAGGGTAAATGGCTAAGTTTAGTTTTTTTAGTTGGCTTTTTACGCTAGATCATAAGCGTGTGGGTATGATTTATACTTTAATTGGGATATGATCAGGTTTTGTTGGGTTGAGTTTTAGGGTAATGATACGTGTTAATTTCGTTGAGCCTTATTTTAATGTAATTTCTTCGGACTGTTATAAATTTTTGATAACTAATCATGGTATTATTATGATTTTTTTTTTTTTGAGGCCGGGGTTGATTGGGGGTTTGGGAAATTATTTAATCCCTTTATTACCCGGTTTACCAAATTTAAATTTCCCCCGGTTGAAACCCTTGAAAGCAGGGTGGCTTTTTCCTTCTATTTTATTTCTGGGGCGAAGTATGGGTTGGGGGGCGGGAAAAGGGGGAACTTTTTACCCCCCTCTCTCCCCTTCCCTTTTTAGGGATAGCCGGGGGGTTAATCTTTTAATGTTTTCTTTACATTTGGCGGGTCTTTCAAGTTTGTTGGGTTCAATAAATTTTATATGTACTCTTTACTCAGCTTTTGTTGATAATTTTGTGTCTCGTAGTTCTATTTTGTTGTGGTCTTATCTATTTACTTCTATTCTTTTATTGTTAACTATTCCTGTGTGGGCTGCTGCCATTACAATGTTGTTGTTTGATCGTAAATTTGGTTCGGCTTTTTTTGATCCTTTGGGTGGTGGTGATCCTGTTTTGTTTCAACATATGTTTTGGTTTTTTGGGCATCCAGAAGTTTATGTATTGATTTTGCCTGGTTTTGGTATGGTAAGTCATGTGTGTAGTAAATTAGGTTGTTCATATGATACTTTTGGTTTTTATGGTTTATTATTTGCTATGTTTTCTATAGTGTGTTTAGGTAGCGTGGTTTGGGGTCACCACATGTTTACTGTGGGGTTGGATGTGAAGACTGCTGTTTTCTTTAGTTCTGTGACTATGATTATTGGGGTTCCCACGGGTATAAAGGTGTTTTCTTGGCTTTATATGATTTTAAATAGTCGTGTTTCGTTGCGTGAGCCTGTGTTTTGATGGGTTTTATCTTTTATTGTGTTGTTTACTATGGGTGGTGTTACTGGTATAATTCTTTCTGCTTGTGTGTTGGATAAAATTTTGCATGACACGTGGTTTGTGGTGGCTCATTTTCATTATGTTATGTCTTTGGGTTCTTATATTAGGATTATTATATTTTTTGTTTGGTGATGGCCTGTTATCACAGGGGTTAGCCTGAATAAGTATTTGTTACAGTGTCATTGTATAGTATCAAATGTGGGTTTTAATTTGTGTTTTTTCCCTATGCATTATTTTGGTATTTGTGGTTTACCTCGGCGTGTTTGTGTGTATGAGTCAGGGTACGCTTGAGTTAATATGCTTTGTTCAATAGGTTCTTTTGTTTCTGCCTTTAGTGGTTGCTTTTTTATTTTTATTTTATGGGAGTCTTTAGCTAAAAAGAATGTTGTTATAGGTTATTATGGTAGTTCTTCAACTTTGCTTAATTTGTGTTGATCGCCAGTGCCTTACCACAGTAATTTTTTTGTGCGTGGATTATTTGTTGATTATTCTGTATTGGCTTTTTAG

>Hap_35

ATGGTTTTGTTGTTGCGTCGTAATGTGGTTGATTTGCCTACTAATTATTCTCTTAATTATTATTGGTGTAGCGGGTTTATGATTTCGGCTTTTATGGTAGTTCAGGTAATTACTGGTGTGATTCTTTCACTTTTGTATGTGGCTGATTCAAGATTAAGTTTTCGTTGTGTTATGGATTTGAGAAAAGATTCTTTTTTTACTTGGGGGGTGCGTTATTGACACATCTGGGGGGTTAGTATTTTGTTTGTCCTGTTTTTTGTTCATATGGGTCGTGCCTTATATTATTCAAGTTATACTAAGAAGGGGGTATGGAAAGTGGGGTTTATTTTATATCTTTTAACTATGGCTGAGGCTTTTTTGGGTTATATTTTACCTTGACATCAAATGTCATATTGGGCTGCTACTGTTTTGACGGCTATTGCCGAGAGTATTCCCTTAGTTGGTCCTACGGTGTTTAAGTATTTGGTGGGGGGGTTTTCTGTAACTAAAGTAACTTTGGTTCGTGTATTTTCAGCTCATGTTTGTTTAGGTTTTGTAATTTTAGGTTTAATGATTTTGCATCTTTTTTATTTGCATTCTTCCGGGTCTAATAACCCTTTATTTTCTTCTTTTGGGTATGGAGATGTTGTTTATTTCCACTCTTATTTTACCACTAAGGATTTTTTTTGTTTGGTTGTTTTATGCTGTATTTTGGTTGGATTTATGTGGTTGGTTCCTGATTTGGTGGTAGATACAGAGGGTTATCTAGAGTCTGATCCTTTGGTGACTCCGGTGTCTATAAAGCCTGAGTGATATTTTTTGATTTATTATGCTATGCTTCGTTCTGTTGAGTCTAAGATAGGTGGTTTAGTGTTGGTGGCTAGATTATTATTTTTTATGTGGGTCCCAACTTTTAAAGACTCTAGTTCATATTTTGTTATTCGACAGGTGGTTTTTTGGGGTTTCGTTTGTCTTTTTGTTGGGTTGACTTATTTAGGCTCATGTCACCCTGAGTATCCTTATTTGGGTATTTGTCAGTTATTTTCGGTTGGTGCTGTAGCTTTTATGTTTATTTATAAGCTATTTTGATCGAGTTATACTAAGTTGGGTTTTAGTATTTTTTTAGGGTAAATGGCTAAGTTTAGTTTTTTTAGTTGGCTTTTTACGCTAGATCATAAGCGTGTGGGTATGATTTATACTTTAATTGGGATATGATCAGGTTTTGTTGGGTTGAGTTTTAGGGTAATGATACGTGTTAATTTCGTTGAGCCTTATTTTAATGTAATTTCTTCGGACTGTTATAAATTTTTGATAACTAATCATGGTATTATTATGATTTTTTTTTTTTTGATCCCGGGGTTGATGGGGGGTTTGGGAAATTATTTAATCCCTTTATTACCCGGTTTACCAAATTTAAATTTCCCCCGGTTGAAACCCTTGAAAGCAGGGTGGCTTTTTCCTTCTATTTTATTTCTGGGGCTAAGAATGGGTTGGGGGGCGGGAAAAGGGGGAACTTTTTACCCCCCTCTCTCCCCTTCCCTTTTTAGGGATAGCCGGGGGGTTAACCTTTTAAGGTTTTCTTTACATTTGGCTGGTCTTTCAAGTTTGTTGGGTTCTATAAATTTTATATGTACTCTTTACTCAGCTTTTGTTGATAATTTTGTGTCTCGTAGTTCTATTTTGTTGGGGTCTTATCTATTTACTTCTATTCTTTTATTGTTAACTATTCCTGTGTTGGCTGCTGCCATTACAATGTTGTTGTTTGATCGTAAATTTGGTTCGGCTTTTTTTGATCCTTTGGGTGGTGGTGATCCTGTTTTGTTTCAACATATGTTTTGGTTTTTTGGGCATCCAGAAGTTTATGTATTGATTTTGCCTGGTTTTGGTATGGTAAGTCATGTGTGTAGTAAATTAGGTTGTTCATATGATACTTTTGGTTTTTATGGTTTATTATTTGCTATGTTTTCTATAGTGTGTTTAGGTAGCGTGGTTTGGGGTCACCACATGTTTACTGTGGGGTTGGACGTGAAGACTGCTGTTTTCTTTAGTTCTGTGACTATGATTATTGGGGTTCCCACGGGTATAAAGGTGTTTTCTTGGCTTTATATGATTTTAAATAGTCGTGTTTCGTTGCGTGAGCCTGTGTTTTGATGGGTTTTATCCTTTATTGTGTTGTTTACTATGGGTGGTGTTACTGGTATAATTCTTTCTGCTTGTGTGTTGGATAAAATTTTGCATGACACGTGGTTTGTGGTGGCTCATTTTCATTATGTTATGTCTTTGGGTTCTTATATTAGGGTTATTATATTTTTTGTTTGGTGATGGCCTGTTATCACAGGGGTTAGCTTGAATAAGTATTTGTTACAGTGTCATTGTATAGTATCAAATGTGGGCTTTAATTTGTGTTTTTTTCCTATGCATTATTTTGGTATTTGTGGTTTACCTCGGCGTGTTTGTGTGTATGAGTCAGGGTACGCTTGAGTTAATATGCTTTGTTCAATAGGTTCTTTTGTTTCTGCCTTTAGTGGTTGCTTTTTTATTTTTATTTTATGGGAGTCTTTAGCTAAAAAGAATGTTGTTATAGGTTATTATGGTAGTTCTTCAACTTTGCTTAATTTGTGTTGATCGCCAGTGCCTTACCACAGTAATTTTTTTGTGCGTGGATTATTTGTTGATTATTCTGTATTGGCTTTTTAG

>Hap_36

ATGGTTTTGTTGTTGCGTCGTAATGTGGTTGATTTGCCTACTAATTATTCTCTTAATTATTATTGGTGTAGCGGGTTTATGATTTCGGCTTTTATGGTAGTTCAGGTAATTACTGGTGTGATTCTTTCACTTTTGTATGTGGCTGATTCAAGATTAAGTTTTCGTTGTGTTATGGATTTGAGAAAAGATTCTTTTTTTACTTGGGGGGTGCGTTATTGACACATCTGGGGGGTTAGTATTTTGTTTGTCCTGTTTTTTGTTCATATGGGTCGTGCCTTATATTATTCAAGTTATACTAAGAAGGGGGTATGGAAAGTGGGGTTTATTTTATATCTTTTAACTATGGCTGAGGCTTTTTTGGGTTATATTTTACCTTGACATCAAATGTCATATTGGGCTGCTACTGTTTTGACGGCTATTGCCGAGAGTATTCCCTTAGTTGGTCCTACGGTGTTTAAGTATTTGGTGGGGGGGTTTTCTGTAACTAAAGTAACTTTGGTTCGTGTATTTTCAGCTCATGTTTGTTTAGGTTTTGTAATTTTAGGTTTAATGATTTTGCATCTTTTTTATTTGCATTCTTCCGGGTCTAATAACCCTTTATTTTCTTCTTTTGGGTATGGAGATGTTGTTTATTTCCACTCTTATTTTACCACTAAGGATTTTTTTTGTTTGGTTGTTTTATGCTGTATTTTGGTTGGATTTATGTGGTTGGTTCCTGATTTGGTGGTAGATACAGAGGGTTATCTAGAGTCTGATCCTTTGGTGACTCCGGTGTCTATAAAGCCTGAGTGATATTTTTTGATTTATTATGCTATGCTTCGTTCTGTTGAGTCTAAGATAGGTGGTTTAGTGTTGGTGGCTAGATTATTATTTTTTATGTGGGTCCCAACTTTTAAAGACTCTAGTTCATATTTTGTTATTCGACAGGTGGTTTTTTGGGGTTTCGTTTGTCTTTTTGTTGGGTTGACTTATTTACGCTCATGTCACCCTGAGTATCCTTATTTGGGTATTTGTCAGTTATTTTCGGTTGGTGCTGTAGCTTTTATGTTTATTTATAAGCTATTTTGATCGAGTTATACTAAGTTGGGTTTTAGTATTTTTTTAGGGTAAATGGCTAAGTTTAGTTTTTTTAGTTGGCTTTTTACGCTAGATCATAAGCGTGTGGGTATGATTTATACTTTAATTGGGATATGATCAGGTTTTGTTGGGTTGAGTTTTAGGGTAATGATACGTGTTAATTTCGTTGAGCCTTATTTTAATGTAATTTCTTCGGACTGTTATAAATTTTTGATAACTAATCATGGTATTATTATGATTTTTTTTTTTTTGATCCCGGGGTTGATGGGGGGTTTTGGAAATTATTTAATTCCTTTATTACCCGGTTTACCAAATTTAAATTTACCCCGGTTGAAACCCTTGAAAGCAGGGTTGCTTTTTCCTTCTATTTTATTTCTGGGGCTAAATATGGGTTTGGGGGCGGGTAAAGGGGGAACTTTTTACCCCCCTCTCTCCCCTTCCCTTTTTAGGGATAGCCGGGGGGTTAACCTTTTAATGTTTTCTTTACATTTGGCTGGTCTTTCAAGTTTGTTGGGTTCTATAAATTTTATATGTACTCTTTACTCAGCTTTTGTTGATAATTTTGTGTCTCGTAGTTCTATTTTGTTGGGGTCTTATCTATTTACTTCTATCCTTTTATTGTTAACTATTCCTGTGTTGGCTGCTGCCATTACAATGTTGTTGTTTGATCGTAAATTTGGTTCGGCTTTTTTTGATCCTTTGGGTGGTGGTGATCCTGTTTTGTTTCAACATATGTTTTGGTTTTTTGGGCATCCAGAAGTTTATGTATTGATTTTGCCTGGTTTTGGTATGGTAAGTCATGTGTGTAGTAAATTAGGTTGTTCATATGATACTTTTGGTTTTTATGGTTTATTATTTGCTATGTTTTCTATAGTGTGTTTAGGTAGCGTGGTTTGGGGTCACCACATGTTTACTGTGGGGTTGGACGTGAAGACTGCTGTTTTCTTTAGTTCTGTGACTATGATTATTGGGGTTCCCACGGGTATAAAGGTGTTTTCTTGGCTTTATATGATTTTAAATAGTCGTGTTTCGTTGCGTGAGCCTGTGTTTTGATGGGTTTTATCCTTTATTGTGTTGTTTACTATGGGTGGTGTTACTGGTATAATTCTTTCTGCTTGTGTGTTGGATAAAATTTTGCATGACACGTGGTTTGTGGTGGCTCATTTTCATTATGTTATGTCTTTGGGTTCTTATATTAGGGTTATTATATTTTTTGTTTGGTGATGGCCTGTTATCACAGGGGTTAGCTTGAATAAGTATTTGTTACAGTGTCATTGTATAGTATCAAATGTGGGCTTTAATTTGTGTTTTTTTCCTATGCATTATTTTGGTATTTGTGGTTTACCTCGGCGTGTTTGTGTGTATGAGTCAGGGTACGCTTGAGTTAATATGCTTTGTTCAATAGGTTCTTTTGTTTCTGCCTTTAGTGGTTGCTTTTTTATTTTTATTTTATGGGAGTCTTTAGCTAAAAAGAATGTTGTTATAGGTTATTATGGTAGTTCTTCAACTTTGCTTAATTTGTGTTGATCGCCAGTGCCTTACCACAGTAATTTTTTTGTGCGTGGATTATTTGTTGATTATTCTGTATTGGCTTTTTAG

>Hap_37

ATGGTTTTGTTGTTGCGTCGTAATGTGGTTGATTTGCCTACTAATTATTCTCTTAATTATTATTGGTGTAGCGGGTTTATGATTTCGGCTTTTATGGTAGTTCAGGTAATTACTGGTGTGATTCTTTCACTTTTGTATGTGGCTGATTCAAGATTAAGTTTTCGTTGTGTTATGGATTTGAGAAAAGATTCTTTTTTTACTTGGGGGGTGCGTTATTGACACATCTGGGGGGTTAGTATTTTGTTTGTCCTGTTTTTTGTTCATATGGGTCGTGCCTTATATTATTCAAGTTATACTAAGAAGGGGGTATGGAAAGTGGGGTTTATTTTATATCTTTTAACTATGGCTGAGGCTTTTTTGGGTTATATTTTACCTTGACATCAAATGTCATATTGGGCTGCTACTGTTTTGACGGCTATTGCCGAGAGTATTCCCTTAGTTGGTCCTACGGTGTTTAAGTATTTGGTGGGGGGGTTTTCTGTAACTAAAGTAACTTTGGTTCGTGTATTTTCAGCTCATGTTTGTTTAGGTTTTGTAATTTTAGGTTTAATGATTTTGCATCTTTTTTATTTGCATTCTTCCGGGTCTAATAACCCTTTATTTTCTTCTTTTGGGTATGGAGATGTTGTTTATTTCCACTCTTATTTTACCACTAAGGATTTTTTTTGTTTGGTTGTTTTATGCTGTATTTTGGTTGGATTTATGTGGTTGGTTCCTGATTTGGTGGTAGATACAGAGGGTTATCTAGAGTCTGATCCTTTGGTGACTCCGGTGTCTATAAAGCCTGAGTGATATTTTTTGATTTATTATGCTATGCTTCGTTCTGTTGAGTCTAAGATAGGTGGTTTAGTGTTGGTGGCTAGATTATTATTTTTTATGTGGGTCCCAACTTTTAAAGACTCTAGTTCATATTTTGTTATTCGACAGGTGGTTTTTTGGGGTTTCGTTTGTCTTTTTGTTGGGTTGACTTATTTAGCCTCATGTCACCCTGAGTATCCTTATTTGGGTATTTGTCAGTTATTTTCGGTTGGTGCTGTAGCTTTTATGTTTATTTATAAGCTATTTTGATCGAGTTATACTAAGTTGGGTTTTAGTATTTTTTTAGGGTAAATGGCTAAGTTTAGTTTTTTTAGTTGGCTTTTTACGCTAGATCATAAGCGTGTGGGTATGATTTATACTTTAATTGGGATATGATCAGGTTTTGTTGGGTTGAGTTTTAGGGTAATGATACGTGTTAATTTCGTTGAGCCTTATTTTAATGTAATTTCTTCGGACTGTTATAAATTTTTGATAACTAATCATGGTATTATTATGATTTTTTTTTTTTTGATCCCGGGGTTGATGGGGGGTTTGGGAAATTATTTAATCCCTTTTTTACCCGGTTTACCAAATTTAAATTTCCCCCGGTTGAAACCCTTGAAAGCAGGGTGGCTTTTTCCTTCTATTTTATTTCTGGGGCTAAAAATGGGTTGGGGGGCGGGAAAAGGGGGAACTTTTTACCCCCCTCTCTCCCCTTCCCTTTTTAGGGATAGCCGGGGGGTTAACCTTTTAAGGTTTTCTTTACATTTGGCTGGTCTTTCAAGTTTGTTGGGTTCTATAAATTTTATATGTACTCTTTACTCAGCTTTTGTTGATAATTTTGTGTCTCGTAGTTCTATTTTGTTGGGGTCTTATCTATTTACTTCTATTCTTTTATTGTTAACTATTCCTGTGTTGGCTGCTGCCATTACAATGTTGTTGTTTGATCGTAAATTTGGTTCGGCTTTTTTTGATCCTTTGGGTGGTGGTGATCCTGTTTTGTTTCAACATATGTTTTGGTTTTTTGGGCATCCAGAAGTTTATGTATTGATTTTGCCTGGTTTTGGTATGGTAAGTCATGTGTGTAGTAAATTAGGTTGTTCATATGATACTTTTGGTTTTTATGGTTTATTATTTGCTATGTTTTCTATAGTGTGTTTAGGTAGCGTGGTTTGGGGTCACCACATGTTTACTGTGGGGTTGGACGTGAAGACTGCTGTTTTCTTTAGTTCTGTGACTATGATTATTGGGGTTCCCACGGGTATAAAGGTGTTTTCTTGGCTTTATATGATTTTAAATAGTCGTGTTTCGTTGCGTGAGCCTGTGTTTTGATGGGTTTTATCCTTTATTGTGTTGTTTACTATGGGTGGTGTTACTGGTATAATTCTTTCTGCTTGTGTGTTGGATAAAATTTTGCATGACACGTGGTTTGTGGTGGCTCATTTTCATTATGTTATGTCTTTGGGTTCTTATATTAGGGTTATTATATTTTTTGTTTGGTGATGGCCTGTTATCACAGGGGTTAGCTTGAATAAGTATTTGTTACAGTGTCATTGTATAGTATCAAATGTGGGCTTTAATTTGTGTTTTTTTCCTATGCATTATTTTGGTATTTGTGGTTTACCTCGGCGTGTTTGTGTGTATGAGTCAGGGTACGCTTGAGTTAATATGCTTTGTTCAATAGGTTCTTTTGTTTCTGCCTTTAGTGGTTGCTTTTTTATTTTTATTTTATGGGAGTCTTTAGCTAAAAAGAATGTTGTTATAGGTTATTATGGTAGTTCTTCAACTTTGCTTAATTTGTGTTGATCGCCAGTGCCTTACCACAGTAATTTTTTTGTGCGTGGATTATTTGTTGATTATTCTGTATTGGCTTTTTAG

>Hap_38

ATGGTTTTGTTGTTGCGTCGTAATGTGGTTGATTTGCCTACTAATTATTCTCTTAATTATTATTGGTGTAGCGGGTTTATGATTTCGGCTTTTATGGTAGTTCAGGTAATTACTGGTGTGATTCTTTCACTTTTGTATGTGGCTGATTCAAGATTAAGTTTTCGTTGTGTTATGGATTTGAGAAAAGATTCTTTTTTTACTTGGGGGGTGCGTTATTGACACATCTGGGGGGTTAGTATTTTGTTTGTCCTGTTTTTTGTTCATATGGGTCGTGCCTTATATTATTCAAGTTATACTAAGAAGGGGGTATGGAAAGTGGGGTTTATTTTATATCTTTTAACTATGGCTGAGGCTTTTTTGGGTTATATTTTACCTTGACATCAAATGTCATATTGGGCTGCTACTGTTTTGACGGCTATTGCCGAGAGTATTCCCTTAGTTGGTCCTACGGTGTTTAAGTATTTGGTGGGGGGGTTTTCTGTAACTAAAGTAACTTTGGTTCGTGTATTTTCAGCTCATGTTTGTTTAGGTTTTGTAATTTTAGGTTTAATGATTTTGCATCTTTTTTATTTGCATTCTTCCGGGTCTAATAACCCTTTATTTTCTTCTTTTGGGTATGGAGATGTTGTTTATTTCCACTCTTATTTTACCACTAAGGATTTTTTTTGTTTGGTTGTTTTATGCTGTATTTTGGTTGGATTTATGTGGTTGGTTCCTGATTTGGTGGTAGATACAGAGGGTTATCTAGAGTCTGATCCTTTGGTGACTCCGGTGTCTATAAAGCCTGAGTGATATTTTTTGATTTATTATGCTATGCTTCGTTCTGTTGAGTCTAAGATAGGTGGTTTAGTGTTGGTGGCTAGATTATTATTTTTTATGTGGGTCCCAACTTTTAAAGACTCTAGTTCATATTTTGTTATTCGACAGGTGGTTTTTTGGGGTTTCGTTTGTCTTTTTGGTGGGTTGACTTATTTAGGCTCATGTCACCCTGAGTATCCTTATTTGGGTATTTGTCAGTTATTTTCGGTTGGTGCTGTAGCTTTTATGTTTATTTATAAGCTATTTTGATCGAGTTATACTAAGTTGGGTTTTAGTATTTTTTTAGGGTAAATGGCTAAGTTTAGTTTTTTTAGTTGGCTTTTTACGCTAGATCATAAGCGTGTGGGTATGATTTATACTTTAATTGGGATATGATCAGGTTTTGTTGGGTTGAGTTTTAGGGTAATGATACGTGTTAATTTCGTTGAGCCTTATTTTAATGTAATTTCTTCGGACTGTTATAAATTTTTGATAACTAATCATGGTATTATTATGATTTTTTTTTTTTTGATCCCGGGGTTGATGGGGGGTTTTGGAAATTATTTAATCCCTTTTTTACCCGGTTTACCAAATTTAAATTTCCCCCGGTTGAAACCCTTGAAAGCAGGGTGGCTTTTTCCTTCTATTTTATTTCTGGGGCTAAAAATGGGTTGGGGGGCGGGAAAAGGGGGAACTTTTTACCCCCCTCTCTCCCCTTCCCTTTTTAGGGATAGCCGGGGGGTTAACCTTTTGAGGTTTTCTTTACATTTGGCTGGTCTTTCAAGTTTGTTGGGTTCTATAAATTTTATATGTACTCTTTACTCAGCTTTTGTTGATAATTTTGTGTCTCGTAGTTCTATTTTGTTGGGGTCTTATCTATTTACTTCTATCCTTTTATTGTTAACTATTCCTGTGTTGGCTGCTGCCATTACAATGTTGTTGTTTGATCGTAAATTTGGTTCGGCTTTTTTTGATCCTTTGGGTGGTGGTGATCCTGTTTTGTTTCAACATATGTTTTGGTTTTTTGGGCATCCAGAAGTTTATGTATTGATTTTGCCTGGTTTTGGTATGGTAAGTCATGTGTGTAGTAAATTAGGTTGTTCATATGATACTTTTGGTTTTTATGGTTTATTATTTGCTATGTTTTCTATAGTGTGTTTAGGTAGCGTGGTTTGGGGTCACCACATGTTTACTGTGGGGTTGGACGTGAAGACTGCTGTTTTCTTTAGTTCTGTGACTATGATTATTGGGGTTCCCACGGGTATAAAGGTGTTTTCTTGGCTTTATATGATTTTAAATAGTCGTGTTTCGTTGCGTGAGCCTGTGTTTTGATGGGTTTTATCCTTTATTGTGTTGTTTACTATGGGTGGTGTTACTGGTATAATTCTTTCTGCTTGTGTGTTGGATAAAATTTTGCATGACACGTGGTTTGTGGTGGCTCATTTTCATTATGTTATGTCTTTGGGTTCTTATATTAGGGTTATTATATTTTTTGTTTGGTGATGGCCTGTTATCACAGGGGTTAGCTTGAATAAGTATTTGTTACAGTGTCATTGTATAGTATCAAATGTGGGCTTTAATTTGTGTTTTTTTCCTATGCATTATTTTGGTATTTGTGGTTTACCTCGGCGTGTTTGTGTGTATGAGTCAGGGTACGCTTGAGTTAATATGCTTTGTTCAATAGGTTCTTTTGTTTCTGCCTTTAGTGGTTGCTTTTTTATTTTTATTTTATGGGAGTCTTTAGCTAAAAAGAATGTTGTTATAGGTTATTATGGTAGTTCTTCAACTTTGCTTAATTTGTGTTGATCGCCAGTGCCTTACCACAGTAATTTTTTTGTGCGTGGATTATTTGTTGATTATTCTGTATTGGCTTTTTAG

>Hap_39

ATGGTTTTGTTGTTGCGTCGTAATGTGGTTGATTTGCCTACTAATTATTCTCTTAATTATTATTGGTGTAGCGGGTTTATGATTTCGGCTTTTATGGTAGTTCAGGTAATTACTGGTGTGATTCTTTCACTTTTGTATGTGGCTGATTCAAGATTAAGTTTTCGTTGTGTTATGGATTTGAGAAAAGATTCTTTTTTTACTTGGGGGGTGCGTTATTGACACATCTGGGGGGTTAGTATTTTGTTTGTCCTGTTTTTTGTTCATATGGGTCGTGCCTTATATTATTCAAGTTATACTAAGAAGGGGGTATGGAAAGTGGGGTTTATTTTATATCTTTTAACTATGGCTGAGGCTTTTTTGGGTTATATTTTACCTTGACATCAAATGTCATATTGGGCTGCTACTGTTTTGACGGCTATTGCCGAGAGTATTCCCTTAGTTGGTCCTACGGTGTTTAAGTATTTGGTGGGGGGGTTTTCTGTAACTAAAGTAACTTTGGTTCGTGTATTTTCAGCTCATGTTTGTTTAGGTTTTGTAATTTTAGGTTTAATGATTTTGCATCTTTTTTATTTGCATTCTTCCGGGTCTAATAACCCTTTATTTTCTTCTTTTGGGTATGGAGATGTTGTTTATTTCCACTCTTATTTTACCACTAAGGATTTTTTTTGTTTGGTTGTTTTATGCTGTATTTTGGTTGGATTTATGTGGTTGGTTCCTGATTTGGTGGTAGATACAGAGGGTTATCTAGAGTCTGATCCTTTGGTGACTCCGGTGTCTATAAAGCCTGAGTGATATTTTTTGATTTATTATGCTATGCTTCGTTCTGTTGAGTCTAAGATAGGTGGTTTAGTGTTGGTGGCTAGATTATTATTTTTTATGTGGGTCCCAACTTTTAAAGACTCTAGTTCATATTTTGTTATTCGACAGGTGGTTTTTTGGGGTTTCGTTTGTCTTTTTGTTGGGTTGACTTATTTAGGCTCATGTCACCCTGAGTATCCTTATTTGGGTATTTGTCAGTTATTTTCGCTTGGTGCTGTAGCTTTTATGTTTATTTATAAGCTATTTTGATCGAGTTATACTAAGTTGGGTTTTAGTATTTTTTTAGGGTAAATGGCTAAGTTTAGTTTTTTTAGTTGGCTTTTTACGCTAGATCATAAGCGTGTGGGTATGATTTATACTTTAATTGGGATATGATCAGGTTTTGTTGGGTTGAGTTTTAGGGTAATGATACGTGTTAATTTCGTTGAGCCTTATTTTAATGTAATTTCTTCGGACTGTTATAAATTTTTGATAACTAATCATGGTATTATTATGATTTTTTTTTTTTTGAGGCCGGGGTTGATGGGGGGTTTGGGAAATTATTTGATCCCTTTATTACCCGGTTTACCAAATTTAAATTTCCCCCGGTTGAAACCCTTGAAAGCAGGGTGGCTTTTTCCTCCAATTTTATTTCTGGGGCGAAAAAGGGGTTGGGGGGCGGGAAAAGGGGGAACTTTTTACCCCCCTCTCTCCCCTTCCCTTTTTAGGGATAGCCGGGGGGTTGACCTTTTGAGGTTTTCTTTACATTTGGCTGGTCTTTCAAGTTTGTTGGGTTCTATAAATTTTATATGTACTCTTTACTCAGCTTTTGTTGATAATTTTGTGTCTCGTAGTTCTATTTTGTTGGGGTCTTATCTATTTACTTCAATCCTTTTATTGTTAACTATTCCTGTGTTGGCTGCTGCCATTACAATGTTGTTGTTTGATCGTAAATTTGGTTCGGCTTTTTTTGATCCTTTGGGTGGTGGTGATCCTGTTTTGTTTCAACATATGTTTTGGTTTTTTGGGCATCCAGAAGTTTATGTATTGATTTTGCCTGGTTTTGGTATGGTAAGTCATGTGTGTAGTAAATTAGGTTGTTCATATGATACTTTTGGTTTTTATGGTTTATTATTTGCTATGTTTTCTATAGTGTGTTTAGGTAGCGTGGTTTGGGGTCACCACATGTTTACTGTGGGGTTGGACGTGAAGACTGCTGTTTTCTTTAGTTCTGTGACTATGATTATTGGGGTTCCCACGGGTATAAAGGTGTTTTCTTGGCTTTATATGATTTTAAATAGTCGTGTTTCGTTGCGTGAGCCTGTGTTTTGATGGGTTTTATCCTTTATTGTGTTGTTTACTATGGGTGGTGTTACTGGTATAATTCTTTCTGCTTGTGTGTTGGATAAAATTTTGCATGACACGTGGTTTGTGGTGGCTCATTTTCATTATGTTATGTCTTTGGGTTCTTATATTAGGGTTATTATATTTTTTGTTTGGTGATGGCCTGTTATCACAGGGGTTAGCTTGAATAAGTATTTGTTACAGTGTCATTGTATAGTATCAAATGTGGGCTTTAATTTGTGTTTTTTTCCTATGCATTATTTTGGTATTTGTGGTTTACCTCGGCGTGTTTGTGTGTATGAGTCAGGGTACGCTTGAGTTAATATGCTTTGTTCAATAGGTTCTTTTGTTTCTGCCTTTAGTGGTTGCTTTTTTATTTTTATTTTATGGGAGTCTTTAGCTAAAAAGAATGTTGTTATAGGTTATTATGGTAGTTCTTCAACTTTGCTTAATTTGTGTTGATCGCCAGTGCCTTACCACAGTAATTTTTTTGTGCGTGGATTATTTGTTGATTATTCTGTATTGGCTTTTTAG

>Hap_40

ATGGTTTTGTTGTTGCGTCGTAATGTGGTTGATTTGCCTACTAATTATTCTCTTAATTATTATTGGTGTAGCGGGTTTATGATTTCGGCTTTTATGGTAGTTCAGGTAATTACTGGTGTGATTCTTTCACTTTTGTATGTGGCTGATTCAAGATTAAGTTTTCGTTGTGTTATGGATTTGAGAAAAGATTCTTTTTTTACTTGGGGGGTGCGTTATTGACACATCTGGGGGGTTAGTATTTTGTTTGTCCTGTTTTTTGTTCATATGGGTCGTGCCTTATATTATTCAAGTTATACTAAGAAGGGGGTATGGAAAGTGGGGTTTATTTTATATCTTTTAACTATGGCTGAGGCTTTTTTGGGTTATATTTTACCTTGACATCAAATGTCATATTGGGCTGCTACTGTTTTGACGGCTATTGCCGAGAGTATTCCCTTAGTTGGTCCTACGGTGTTTAAGTATTTGGTGGGGGGGTTTTCTGTAACTAAAGTAACTTTGGTTCGTGTATTTTCAGCTCATGTTTGTTTAGGTTTTGTAATTTTAGGTTTAATGATTTTGCATCTTTTTTATTTGCATTCTTCCGGGTCTAATAACCCTTTATTTTCTTCTTTTGGGTATGGAGATGTTGTTTATTTCCACTCTTATTTTACCACTAAGGATTTTTTTTGTTTGGTTGTTTTATGCTGTATTTTGGTTGGATTTATGTGGTTGGTTCCTGATTTGGTGGTAGATACAGAGGGTTATCTAGAGTCTGATCCTTTGGTGACTCCGGTGTCTATAAAGCCTGAGTGATATTTTTTGATTTATTATGCTATGCTTCGTTCTGTTGAGTCTAAGATAGGTGGTTTAGTGTTGGTGGCTAGATTATTATTTTTTATGTGGGTCCCAACTTTTAAAGACTCTAGTTCATATTTTGTTATTCGACAGGTGGTTTTTTGGGGTTTCGTTTGTCTTTTTGTTGGGTTGACTTATTTAGGCTCATGTCACCCTGAGTATCCTTATTTGGGTATTTGTCAGTTATTTTCGCTTGGTGCTGTAGCTTTTATGTTTATTTATAAGCTATTTTGATCGAGTTATACTAAGTTGGGTTTTAGTATTTTTTTAGGGTAAATGGCTAAGTTTAGTTTTTTTAGTTGGCTTTTTACGCTAGATCATAAGCGTGTGGGTATGATTTATACTTTAATTGGGATATGATCAGGTTTTGTTGGGTTGAGTTTTAGGGTAATGATACGTGTTAATTTCGTTGAGCCTTATTTTAATGTAATTTCTTCGGACTGTTATAAATTTTTGATAACTAATCATGGTATTATTATGATTTTTTTTTTTTTGAGGCCGGGGTTGATGGGGGGTTTGGGAAATTATTTGATCCCTTTATTACCCGGTTTACCAAATTTAAATTTCCCCCGGTTAAAACCCTTGAAAGCAGGGTGGCTTTTTCCTCCAATTTTATTCCTGGGGCGAAAAAGGGGTTGGGGGGCGGGAAAAGGGGGAACTTTTTACCCCCCTCTCTCCCCTTCCCTTTTTAGGGATAGCCGGGGGGTTGACCTTTTGAGGTTTTCTTTACATTTGGCTGGTCTTTCAAGTTTGTTGGGTTCTATAAATTTTATATGTACTCTTTACTCAGCTTTTGTTGATAATTTTGTGTCTCGTAGTTCTATTTTGTTGGGGTCTTATCTATTTACTTCAATCCTTTTATTGTTAACTATTCCTGTGTTGGCTGCTGCCATTACAATGTTGTTGTTTGATCGTAAATTTGGTTCGGCTTTTTTTGATCCTTTGGGTGGTGGTGATCCTGTTTTGTTTCAACATATGTTTTGGTTTTTTGGGCATCCAGAAGTTTATGTATTGATTTTGCCTGGTTTTGGTATGGTAAGTCATGTGTGTAGTAAATTAGGTTGTTCATATGATACTTTTGGTTTTTATGGTTTATTATTTGCTATGTTTTCTATAGTGTGTTTAGGTAGCGTGGTTTGGGGTCACCACATGTTTACTGTGGGGTTGGACGTGAAGACTGCTGTTTTCTTTAGTTCTGTGACTATGATTATTGGGGTTCCCACGGGTATAAAGGTGTTTTCTTGGCTTTATATGATTTTAAATAGTCGTGTTTCGTTGCGTGAGCCTGTGTTTTGATGGGTTTTATCCTTTATTGTGTTGTTTACTATGGGTGGTGTTACTGGTATAATTCTTTCTGCTTGTGTGTTGGATAAAATTTTGCATGACACGTGGTTTGTGGTGGCTCATTTTCATTATGTTATGTCTTTGGGTTCTTATATTAGGGTTATTATATTTTTTGTTTGGTGATGGCCTGTTATCACAGGGGTTAGCTTGAATAAGTATTTGTTACAGTGTCATTGTATAGTATCAAATGTGGGCTTTAATTTGTGTTTTTTTCCTATGCATTATTTTGGTATTTGTGGTTTACCTCGGCGTGTTTGTGTGTATGAGTCAGGGTACGCTTGAGTTAATATGCTTTGTTCAATAGGTTCTTTTGTTTCTGCCTTTAGTGGTTGCTTTTTTATTTTTATTTTATGGGAGTCTTTAGCTAAAAAGAATGTTGTTATAGGTTATTATGGTAGTTCTTCAACTTTGCTTAATTTGTGTTGATCGCCAGTGCCTTACCACAGTAATTTTTTTGTGCGTGGATTATTTGTTGATTATTCTGTATTGGCTTTTTAG

>Hap_41

ATGGTTTTGTTGTTGCGTCGTAATGTGGTTGATTTGCCTACTAATTATTCTCTTAATTATTATTGGTGTAGCGGGTTTATGATTTCGGCTTTTATGGTAGTTCAGGTAATTACTGGTGTGATTCTTTCACTTTTGTATGTGGCTGATTCAAGATTAAGTTTTCGTTGTGTTATGGATTTGAGAAAAGATTCTTTTTTTACTTGGGGGGTGCGTTATTGACACATCTGGGGGGTTAGTATTTTGTTTGTCCTGTTTTTTGTTCATATGGGTCGTGCCTTATATTATTCAAGTTATACTAAGAAGGGGGTATGGAAAGTGGGGTTTATTTTATATCTTTTAACTATGGCTGAGGCTTTTTTGGGTTATATTTTACCTTGACATCAAATGTCATATTGGGCTGCTACTGTTTTGACGGCTATTGCCGAGAGTATTCCCTTAGTTGGTCCTACGGTGTTTAAGTATTTGGTGGGGGGGTTTTCTGTAACTAAAGTAACTTTGGTTCGTGTATTTTCAGCTCATGTTTGTTTAGGTTTTGTAATTTTAGGTTTAATGATTTTGCATCTTTTTTATTTGCATTCTTCCGGGTCTAATAACCCTTTATTTTCTTCTTTTGGGTATGGAGATGTTGTTTATTTCCACTCTTATTTTACCACTAAGGATTTTTTTTGTTTGGTTGTTTTATGCTGTATTTTGGTTGGATTTATGTGGTTGGTTCCTGATTTGGTGGTAGATACAGAGGGTTATCTAGAGTCTGATCCTTTGGTGACTCCGGTGTCTATAAAGCCTGAGTGATATTTTTTGATTTATTATGCTATGCTTCGTTCTGTTGAGTCTAAGATAGGTGGTTTAGTGTTGGTGGCTAGATCATTATTTTTTATGTGGGTCCCAACTTTTAAAGACTCTAGTTCATATTTTGTTATTCGACAGGTGGTTTTTTGGGGTTTCGTTTGTCTTTTTGTTGGGTTGACTTATTTAGGCTCATGTCACCCTGAGTATCCTTATTTGGGTATTTGTCAGTTATTTTCGCTTGGTGCTGTAGCTTTTATGTTTATTTATAAGCTATTTTGATCGAGTTATACTAAGTTGGGTTTTAGTATTTTTTTAGGGTAAATGGCTAAGTTTAGTTTTTTTAGTTGGCTTTTTACGCTAGATCATAAGCGTGTGGGTATGATTTATACTTTAATTGGGATATGATCAGGTTTTGTTGGGTTGAGTTTTAGGGTAATGATACGTGTTAATTTCGTTGAGCCTTATTTTAATGTAATTTCTTCGGACTGTTATAAATTTTTGATAACTAATCATGGTATTATTATGATTTTTTTTTTTTTGAGGCCGGGGTTGATTGGGGGTTTGGGAAATTATTTGATCCCTTTATTACCCGGTTTACCAAATTTAAATTTACCCCGGTTAAAACCCTTGAAAGCAGGGTGGCTTTTTCCTCCAATTTTATTCCTGGGGCGAAGAAGGGGTTGGGGGGCGGGAAAAGGGGGAACTTTTTACCCCCCTCTCTCCCCTTCCCTTTTTAGGGATAGCCGGGGGGTTGACCTTTTGAGGTTTTCTTTACATTTGGCTGGTCTTTCAAGTTTGTTGGGTTCTATAAATTTTATATGTACTCTTTACTCAGCTTTTGTTGATAATTTTGTGTCTCGTAGTTCTATTTTGTTGGGGTCTTATCTATTTACTTCAATCCTTTTATTGTTAACTATTCCTGTGTTGGCTGCTGCCATTACAATGTTGTTGTTTGATCGTAAATTTGGTTCGGCTTTTTTTGATCCTTTGGGTGGTGGTGATCCTGTTTTGTTTCAACATATGTTTTGGTTTTTTGGGCATCCAGAAGTTTATGTATTGATTTTGCCTGGTTTTGGTATGGTAAGTCATGTGTGTAGTAAATTAGGTTGTTCATATGATACTTTTGGTTTTTATGGTTTATTATTTGCTATGTTTTCTATAGTGTGTTTAGGTAGCGTGGTTTGGGGTCACCACATGTTTACTGTGGGGTTGGACGTGAAGACTGCTGTTTTCTTTAGTTCTGTGACTATGATTATTGGGGTTCCCACGGGTATAAAGGTGTTTTCTTGGCTTTATATGATTTTAAATAGTCGTGTTTCGTTGCGTGAGCCTGTGTTTTGATGGGTTTTATCCTTTATTGTGTTGTTTACTATGGGTGGTGTTACTGGTATAATTCTTTCTGCTTGTGTGTTGGATAAAATTTTGCATGACACGTGGTTTGTGGTGGCTCATTTTCATTATGTTATGTCTTTGGGTTCTTATATTAGGGTTATTATATTTTTTGTTTGGTGATGGCCTGTTATCACAGGGGTTAGCTTGAATAAGTATTTGTTACAGTGTCATTGTATAGTATCAAATGTGGGCTTTAATTTGTGTTTTTTTCCTATGCATTATTTTGGTATTTGTGGTTTACCTCGGCGTGTTTGTGTGTATGAGTCAGGGTACGCTTGAGTTAATATGCTTTGTTCAATAGGTTCTTTTGTTTCTGCCTTTAGTGGTTGCTTTTTTATTTTTATTTTATGGGAGTCTTTAGCTAAAAAGAATGTTGTTATAGGTTATTATGGTAGTTCTTCAACTTTGCTTAATTTGTGTTGATCGCCAGTGCCTTACCACAGTAATTTTTTTGTGCGTGGATTATTTGTTGATTATTCTGTATTGGCTTTTTAG

>Hap_42

ATGGTTTTGTTGTTGCGTCGTAATGTGGTTGATTTGCCTACTAATTATTCTCTTAATTATTATTGGTGTAGCGGGTTTATGATTTCGGCTTTTATGGTAGTTCAGGTAATTACTGGTGTGATTCTTTCACTTTTGTATGTGGCTGATTCAAGATTAAGTTTTCGTTGTGTTATGGATTTGAGAAAAGATTCTTTTTTTACTTGGGGGGTGCGTTATTGACACATCTGGGGGGTTAGTATTTTGTTTGTCCTGTTTTTTGTTCATATGGGTCGTGCCTTATATTATTCAAGTTATACTAAGAAGGGGGTATGGAAAGTGGGGTTTATTTTATATCTTTTAACTATGGCTGAGGCTTTTTTGGGTTATATTTTACCTTGACATCAAATGTCATATTGGGCTGCTACTGTTTTGACGGCTATTGCCGAGAGTATTCCCTTAGTTGGTCCTACGGTGTTTAAGTATTTGGTGGGGGGGTTTTCTGTAACTAAAGTAACTTTGGTTCGTGTATTTTCAGCTCATGTTTGTTTAGGTTTTGTAATTTTAGGTTTAATGATTTTGCATCTTTTTTATTTGCATTCTTCCGGGTCTAATAACCCTTTATTTTCTTCTTTTGGGTATGGAGATGTTGTTTATTTCCACTCTTATTTTACCACTAAGGATTTTTTTTGTTTGGTTGTTTTATGCTGTATTTTGGTTGGATTTATGTGGTTGGTTCCTGATTTGGTGGTAGATACAGAGGGTTATCTACAGTCTGATCCTTTGGTGACTCCGGTGTCTATAAAGCCTGAGTGATATTTTTTGATTTATTATGCTATGCTTCGTTCTGTTGAGTCTAAGATAGGTGGTTTAGTGTTGGTGGCTAGATCATTATTTTTTATGTGGGTCCCAACTTTTAAAGACTCTAGTTCATATTTTGTTATTCGACAGGTGGTTTTTTGGGGTTTCGTTTGTCTTTTTGTTGGGTTGACTTATTTAGGCTCATGTCACCCTGAGTATCCTTATTTGGGTATTTGTCAGTTATTTTCGCTTGGTGCTGTAGCTTTTATGTTTATTTATAAGCTATTTTGATCGAGTTATACTAAGTTGGGTTTTAGTATTTTTTTAGGGTAAATGGCTAAGTTTAGTTTTTTTAGTTGGCTTTTTACGCTAGATCATAAGCGTGTGGGTATGATTTATACTTTAATTGGGATATGATCAGGTTTTGTTGGGTTGAGTTTTAGGGTAATGATACGTGTTAATTTCGTTGAGCCTTATTTTAATGTAATTTCTTCGGACTGTTATAAATTTTTGATAACTAATCATGGTATTATTATGATTTTTTTTTTTTTGAGGCCGGGGTTGATTGGGGGTTTGGGAAATTATTTGATCCCTTTATTACCCGGTTTACCAAATTTAAATTTACCCCGGTTAAAACCCTTGAAAGCAGGGTGGCTTTTTCCTCCAATTTTATTCCTGGGGCGAAAAAGGGGTTGGGGGGCGGGAAAAGGGGGAACTTTTTACCCCCCTCTCTCCCCTTCCCTTTTTAGGGATAGCCGGGGGGTTGACCTTTTGAGGTTTTCTTTACATTTGGCTGGTCTTTCAAGTTTGTTGGGTTCTATAAATTTTATATGTACTCTTTACTCAGCTTTTGTTGATAATTTTGTGTCTCGTAGTTCTATTTTGTTGGGGTCTTATCTATTTACTTCAATCCTTTTATTGTTAACTATTCCTGTGTTGGCTGCTGCCATTACAATGTTGTTGTTTGATCGTAAATTTGGTTCGGCTTTTTTTGATCCTTTGGGTGGTGGTGATCCTGTTTTGTTTCAACATATGTTTTGGTTTTTTGGGCATCCAGAAGTTTATGTATTGATTTTGCCTGGTTTTGGTATGGTAAGTCATGTGTGTAGTAAATTAGGTTGTTCATATGATACTTTTGGTTTTTATGGTTTATTATTTGCTATGTTTTCTATAGTGTGTTTAGGTAGCGTGGTTTGGGGTCACCACATGTTTACTGTGGGGTTGGACGTGAAGACTGCTGTTTTCTTTAGTTCTGTGACTATGATTATTGGGGTTCCCACGGGTATAAAGGTGTTTTCTTGGCTTTATATGATTTTAAATAGTCGTGTTTCGTTGCGTGAGCCTGTGTTTTGATGGGTTTTATCCTTTATTGTGTTGTTTACTATGGGTGGTGTTACTGGTATAATTCTTTCTGCTTGTGTGTTGGATAAAATTTTGCATGACACGTGGTTTGTGGTGGCTCATTTTCATTATGTTATGTCTTTGGGTTCTTATATTAGGGTTATTATATTTTTTGTTTGGTGATGGCCTGTTATCACAGGGGTTAGCTTGAATAAGTATTTGTTACAGTGTCATTGTATAGTATCAAATGTGGGCTTTAATTTGTGTTTTTTTCCTATGCATTATTTTGGTATTTGTGGTTTACCTCGGCGTGTTTGTGTGTATGAGTCAGGGTACGCTTGAGTTAATATGCTTTGTTCAATAGGTTCTTTTGTTTCTGCCTTTAGTGGTTGCTTTTTTATTTTTATTTTATGGGAGTCTTTAGCTAAAAAGAATGTTGTTATAGGTTATTATGGTAGTTCTTCAACTTTGCTTAATTTGTGTTGATCGCCAGTGCCTTACCACAGTAATTTTTTTGTGCGTGGATTATTTGTTGATTATTCTGTATTGGCTTTTTAG

>Hap_43

ATGGTTTTGTTGTTGCGTCGTAATGTGGTTGATTTGCCTACTAATTATTCTCTTAATTATTATTGGTGTAGCGGGTTTATGATTTCGGCTTTTATGGTAGTTCAGGTAATTACTGGTGTGATTCTTTCACTTTTGTATGTGGCTGATTCAAGATTAAGTTTTCGTTGTGTTATGGATTTGAGAAAAGATTCTTTTTTTACTTGGGGGGTGCGTTATTGACACATCTGGGGGGTTAGTATTTTGTTTGTCCTGTTTTTTGTTCATATGGGTCGTGCCTTATATTATTCAAGTTATACTAAGAAGGGGGTATGGAAAGTGGGGTTTATTTTATATCTTTTAACTATGGCTGAGGCTTTTTTGGGTTATATTTTACCTTGACATCAAATGTCATATTGGGCTGCTACTGTTTTGACGGCTATTGCCGAGAGTATTCCCTTAGTTGGTCCTACGGTGTTTAAGTATTTGGTGGGGGGGTTTTCTGTAACTAAAGTAACTTTGGTTCGTGTATTTTCAGCTCATGTTTGTTTAGGTTTTGTAATTTTAGGTTTAATGATTTTGCATCTTTTTTATTTGCATTCTTCCGGGTCTAATAACCCTTTATTTTCTTCTTTTGGGTATGGAGATGTTGTTTATTTCCACTCTTATTTTACCACTAAGGATTTTTTTTGTTTGGTTGTTTTATGCTGTATTTTGGTTGGATTTATGTGGTTGGTTCCTGATTTGGTGGTAGATACAGAGGGTTATCTAGAGTCTGATCCTTTGGTGACTCCGGTGTCTATAAAGCCTGAGTGATATTTTTTGATTTATTATGCTATGCTTCGTTCTGTTGAGTCTAAGATAGGTGGTTTAGTGTTGGTGGCTAGATTATTATTTTTTATGTGGGTCCCAACTTTTAAAGACTCTAGTTCATATTTTGTTATTCGACAGGTGGTTTTTTGGGGTTTCGTTTGTCTTTTTGGTGGGTTGACTTATTTAGGCTCATGTCACCCTGAGTATCCTTATTTGGGTATTTGTCAGTTATTTTCGGTTGGTGCTGTAGCTTTTATGTTTATTTATAAGCTATTTTGATCGAGTTATACTAAGTTGGGTTTTAGTATTTTTTTAGGGTAAATGGCTAAGTTTAGTTTTTTTAGTTGGCTTTTTACGCTAGATCATAAGCGTGTGGGTATGATTTATACTTTAATTGGGATATGATCAGGTTTTGTTGGGTTGAGTTTTAGGGTAATGATACGTGTTAATTTCGTTGAGCCTTATTTTAATGTAATTTCTTCGGACTGTTATAAATTTTTGATAACTAATCATGGTATTATTATGATTTTTTTTTTTTTGATCCCGGGGTTGATGGGGGGTTTTGGAAATTATTTAATCCCTTTATTACCCGGTTTACCAAATTTAAATTTCCCCCGGTTGAAACCCTTGAAAGCAGGGTGGCTTTTTCCTTCTATTTTATTTCTGGGGCTAAAAATGGGTTGGGGGGCGGGAAAAGGGGGAACTTTTTACCCCCCTCTCTCCCCTTCCCTTTTTAGGGATAGCCGGGGGGTTAACCTTTTGAGGTTTTCTTTACATTTGGCTGGTCTTTCAAGTTTGTTGGGTTCTATAAATTTTATATGTACTCTTTACTCAGCTTTTGTTGATAATTTTGTGTCTCGTAGTTCTATTTTGTTGGGGTCTTATCTATTTACTTCTATCCTTTTATTGTTAACTATTCCTGTGTTGGCTGCTGCCATTACAATGTTGTTGTTTGATCGTAAATTTGGTTCGGCTTTTTTTGATCCTTTGGGTGGTGGTGATCCTGTTTTGTTTCAACATATGTTTTGGTTTTTTGGGCATCCAGAAGTTTATGTATTGATTTTGCCTGGTTTTGGTATGGTAAGTCATGTGTGTAGTAAATTAGGTTGTTCATATGATACTTTTGGTTTTTATGGTTTATTATTTGCTATGTTTTCTATAGTGTGTTTAGGTAGCGTGGTTTGGGGTCACCACATGTTTACTGTGGGGTTGGACGTGAAGACTGCTGTTTTCTTTAGTTCTGTGACTATGATTATTGGGGTTCCCACGGGTATAAAGGTGTTTTCTTGGCTTTATATGATTTTAAATAGTCGTGTTTCGTTGCGTGAGCCTGTGTTTTGATGGGTTTTATCCTTTATTGTGTTGTTTACTATGGGTGGTGTTACTGGTATAATTCTTTCTGCTTGTGTGTTGGATAAAATTTTGCATGACACGTGGTTTGTGGTGGCTCATTTTCATTATGTTATGTCTTTGGGTTCTTATATTAGGGTTATTATATTTTTTGTTTGGTGATGGCCTGTTATCACAGGGGTTAGCTTGAATAAGTATTTGTTACAGTGTCATTGTATAGTATCAAATGTGGGCTTTAATTTGTGTTTTTTTCCTATGCATTATTTTGGTATTTGTGGTTTACCTCGGCGTGTTTGTGTGTATGAGTCAGGGTACGCTTGAGTTAATATGCTTTGTTCAATAGGTTCTTTTGTTTCTGCCTTTAGTGGTTGCTTTTTTATTTTTATTTTATGGGAGTCTTTAGCTAAAAAGAATGTTGTTATAGGTTATTATGGTAGTTCTTCAACTTTGCTTAATTTGTGTTGATCGCCAGTGCCTTACCACAGTAATTTTTTTGTGCGTGGATTATTTGTTGATTATTCTGTATTGGCTTTTTAG

>Hap_44

ATGGTTTTGTTGTTGCGTCGTAATGTGGTTGATTTGCCTACTAATTATTCTCTTAATTATTATTGGTGTAGCGGGTTTATGATTTCGGCTTTTATGGTAGTTCAGGTAATTACTGGTGTGATTCTTTCACTTTTGTATGTGGCTGATTCAAGATTAAGTTTTCGTTGTGTTATGGATTTGAGAAAAGATTCTTTTTTTACTTGGGGGGTGCGTTATTGACACATCTGGGGGGTTAGTATTTTGTTTGTCCTGTTTTTTGTTCATATGGGTCGTGCCTTATATTATTCAAGTTATACTAAGAAGGGGGTATGGAAAGTGGGGTTTATTTTATATCTTTTAACTATGGCTGAGGCTTTTTTGGGTTATATTTTACCTTGACATCAAATGTCATATTGGGCTGCTACTGTTTTGACGGCTATTGCCGAGAGTATTCCCTTAGTTGGTCCTACGGTGTTTAAGTATTTGGTGGGGGGGTTTTCTGTAACTAAAGTAACTTTGGTTCGTGTATTTTCAGCTCATGTTTGTTTAGGTTTTGTAATTTTAGGTTTAATGATTTTGCATCTTTTTTATTTGCATTCTTCCGGGTCTAATAACCCTTTATTTTCTTCTTTTGGGTATGGAGATGTTGTTTATTTCCACTCTTATTTTACCACTAAGGATTTTTTTTGTTTGGTTGTTTTATGCTGTATTTTGGTTGGATTTATGTGGTTGGTTCCTGATTTGGTGGTAGATACAGAGGGTTATCTAGAGTCTGATCCTTTGGTGACTCCGGTGTCTATAAAGCCTGAGTGATATTTTTTGATTTATTATGCTATGCTTCGTTCTGTTGAGTCTAAGATAGGTGGTTTAGTGTTGGTGGCTAGATTATTATTTTTTATGTGGGTCCCAACTTTTAAAGACTCTAGTTCATATTTTGTTATTCGACAGGTGGTTTTTTGGGGTTTCGTTTGTCTTTTTGTTGGGTTGACTTATTTAGGCTCATGTCACCCTGAGTATCCTTATTTGGGTATTTGTCAGTTATTTTCGGTTGGTGCTGTAGCTTTTATGTTTATTTATAAGCTATTTTGATCGAGTTATACTAAGTTGGGTTTTAGTATTTTTTTAGGGTAAATGGCTAAGTTTAGTTTTTTTAGTTGGCTTTTTACGCTAGATCATAAGCGTGTGGGTATGATTTATACTTTAATTGGGATATGATCAGGTTTTGTTGGGTTGAGTTTTAGGGTAATGATACGTGTTAATTTCGTTGAGCCTTATTTTAATGTAATTTCTTCGGACTGTTATAAATTTTTGATAACTAATCATGGTATTATTATGATTTTTTTTTTTTTGATCCCGGGGTTGATGGGGGGTTTTGGAAATTATTTAATCCCTTTATTACCCGGTTTACCAAATTTAAATTTCCCCCGGTTGAAACCCTTGAAAGCAGGGTGGCTTTTTCCTTCTATTTTATTTCTGGGGCTAAAAATGGGTTGGGGGGCGGGAAAAGGGGGAACTTTTTACCCCCCTCTCTCCCCTTCCCTTTTTAGGGATAGCCGGGGGGTTAACCTTTTGAGGTTTTCTTTACATTTGGCTGGTCTTTCAAGTTTGTTGGGTTCTATAAATTTTATATGTACTCTTTACTCAGCTTTTGTTGATAATTTTGTGTCTCGTAGTTCTATTTTGTTGGGGTCTTATCTATTTACTTCTATCCTTTTATTGTTAACTATTCCTGTGTTGGCTGCTGCCATTACAATGTTGTTGTTTGATCGTAAATTTGGTTCGGCTTTTTTTGATCCTTTGGGTGGTGGTGATCCTGTTTTGTTTCAACATATGTTTTGGTTTTTTGGGCATCCAGAAGTTTATGTATTGATTTTGCCTGGTTTTGGTATGGTAAGTCATGTGTGTAGTAAATTAGGTTGTTCATATGATACTTTTGGTTTTTATGGTTTATTATTTGCTATGTTTTCTATAGTGTGTTTAGGTAGCGTGGTTTGGGGTCACCACATGTTTACTGTGGGGTTGGACGTGAAGACTGCTGTTTTCTTTAGTTCTGTGACTATGATTATTGGGGTTCCCACGGGTATAAAGGTGTTTTCTTGGCTTTATATGATTTTAAATAGTCGTGTTTCGTTGCGTGAGCCTGTGTTTTGATGGGTTTTATCCTTTATTGTGTTGTTTACTATGGGTGGTGTTACTGGTATAATTCTTTCTGCTTGTGTGTTGGATAAAATTTTGCATGACACGTGGTTTGTGGTGGCTCATTTTCATTATGTTATGTCTTTGGGTTCTTATATTAGGGTTATTATATTTTTTGTTTGGTGATGGCCTGTTATCACAGGGGTTAGCTTGAATAAGTATTTGTTACAGTGTCATTGTATAGTATCAAATGTGGGCTTTAATTTGTGTTTTTTTCCTATGCATTATTTTGGTATTTGTGGTTTACCTCGGCGTGTTTGTGTGTATGAGTCAGGGTACGCTTGAGTTAATATGCTTTGTTCAATAGGTTCTTTTGTTTCTGCCTTTAGTGGTTGCTTTTTTATTTTTATTTTATGGGAGTCTTTAGCTAAAAAGAATGTTGTTATAGGTTATTATGGTAGTTCTTCAACTTTGCTTAATTTGTGTTGATCGCCAGTGCCTTACCACAGTAATTTTTTTGTGCGTGGATTATTTGTTGATTATTCTGTATTGGCTTTTTAG

>Hap_45

ATGGTTTTGTTGTTGCGTCGTAATGTGGTTGATTTGCCTACTAATTATTCTCTTAATTATTATTGGTGTAGCGGGTTTATGATTTCGGCTTTTATGGTAGTTCAGGTAATTACTGGTGTGATTCTTTCACTTTTGTATGTGGCTGATTCAAGATTAAGTTTTCGTTGTGTTATGGATTTGAGAAAAGATTCTTTTTTTACTTGGGGGGTGCGTTATTGACACATCTGGGGGGTTAGTATTTTGTTTGTCCTGTTTTTTGTTCATATGGGTCGTGCCTTATATTATTCAAGTTATACTAAGAAGGGGGTATGGAAAGTGGGGTTTATTTTATATCTTTTAACTATGGCTGAGGCTTTTTTGGGTTATATTTTACCTTGACATCAAATGTCATATTGGGCTGCTACTGTTTTGACGGCTATTGCCGAGAGTATTCCCTTAGTTGGTCCTACGGTGTTTAAGTATTTGGTGGGGGGGTTTTCTGTAACTAAAGTAACTTTGGTTCGTGTATTTTCAGCTCATGTTTGTTTAGGTTTTGTAATTTTAGGTTTAATGATTTTGCATCTTTTTTATTTGCATTCTTCCGGGTCTAATAACCCTTTATTTTCTTCTTTTGGGTATGGAGATGTTGTTTATTTCCACTCTTATTTTACCACTAAGGATTTTTTTTGTTTGGTTGTTTTATGCTGTATTTTGGTTGGATTTATGTGGTTGGTTCCTGATTTGGTGGTAGATACAGAGGGTTATCTAGAGTCTGATCCTTTGGTGACTCCGGTGTCTATAAAGCCTGAGTGATATTTTTTGATTTATTATGCTATGCTTCGTTCTGTTGAGTCTAAGATAGGTGGTTTAGTGTTGGTGGCTAGATTATTATTTTTTATGTGGGTCCCAACTTTTAAAGACTCTAGTTCATATTTTGTTATTCGACAGGTGGTTTTTTGGGGTTTCGTTTGTCTTTTTGGTGGGTTGACTTATTTACGCTCATGTCACCCTGAGTATCCTTATTTGGGTATTTGTCAGTTATTTTCGCTTGGTGCTGTAGCTTTTATGTTTATTTATAAGCTATTTTGATCGAGTTATACTAAGTTGGGTTTTAGTATTTTTTTAGGGTAAATGGCTAAGTTTAGTTTTTTTAGTTGGCTTTTTACGCTAGATCATAAGCGTGTGGGTATGATTTATACTTTAATTGGGATATGATCAGGTTTTGTTGGGTTGAGTTTTAGGGTAATGATACGTGTTAATTTCGTTGAGCCTTATTTTAATGTAATTTCTTCGGACTGTTATAAATTTTTGATAACTAATCATGGTATTATTATGATTTTTTTTTTTTTGAGGCCGGGGTTGATGGGGGGTTTGGGAAATTATTTGATCCCTTTATTACCCGGTTTACCAAATTTAAATTTCCCCCGGTTGAAACCCTTGAAAGCAGGGTTGCTTTTTCCTTCTATTTTATTTCTGGGGCTAAGAATGTGTTTGGGGGCTGGAATAGGGGGAACTTTTTACCCCCCTCTCTCCCCTTCTCTTTTTAGGGATAGCCGGGGGGTTGATCTTTTGATGTTTTCTTTACATTTGGCTGGTCTTTCAAGTTTGTTGGGTTCTATAAATTTTATATGTACTCTTTACTCAGCTTTTGTTGATAATTTTGTGTCTCGTAGTTCTATTTTGTTGGGGTCTTATCTATTTACTTCTATCCTTTTATTGTTAACTATTCCTGTGTTGGCTGCTGCCATTACAATGTTGTTGTTTGATCGTAAATTTGGTTCGGCTTTTTTTGATCCTTTGGGTGGTGGTGATCCTGTTTTGTTTCAACATATGTTTTGGTTTTTTGGGCATCCAGAAGTTTATGTATTGATTTTGCCTGGTTTTGGTATGGTAAGTCATGTGTGTAGTAAATTAGGTTGTTCATATGATACTTTTGGTTTTTATGGTTTATTATTTGCTATGTTTTCTATAGTGTGTTTAGGTAGCGTGGTTTGGGGTCACCACATGTTTACTGTGGGGTTGGACGTGAAGACTGCTGTTTTCTTTAGTTCTGTGACTATGATTATTGGGGTTCCCACGGGTATAAAGGTGTTTTCTTGGCTTTATATGATTTTAAATAGTCGTGTTTCGTTGCGTGAGCCTGTGTTTTGATGGGTTTTATCCTTTATTGTGTTGTTTACTATGGGTGGTGTTACTGGTATAATTCTTTCTGCTTGTGTGTTGGATAAAATTTTGCATGACACGTGGTTTGTGGTGGCTCATTTTCATTATGTTATGTCTTTGGGTTCTTATATTAGGGTTATTATATTTTTTGTTTGGTGATGGCCTGTTATCACAGGGGTTAGCTTGAATAAGTATTTGTTACAGTGTCATTGTATAGTATCAAATGTGGGCTTTAATTTGTGTTTTTTTCCTATGCATTATTTTGGTATTTGTGGTTTACCTCGGCGTGTTTGTGTGTATGAGTCAGGGTACGCTTGAGTTAATATGCTTTGTTCAATAGGTTCTTTTGTTTCTGCCTTTAGTGGTTGCTTTTTTATTTTTATTTTATGGGAGTCTTTAGCTAAAAAGAATGTTGTTATAGGTTATTATGGTAGTTCTTCAACTTTGCTTAATTTGTGTTGATCGCCAGTGCCTTACCACAGTAATTTTTTTGTGCGTGGATTATTTGTTGATTATTCTGTATTGGCTTTTTAG

>Hap_46

ATGGTTTTGTTGTTGCGTCGTAATGTGGTTGATTTGCCTACTAATTATTCTCTTAATTATTATTGGTGTAGCGGGTTTATGATTTCGGCTTTTATGGTAGTTCAGGTAATTACTGGTGTGATTCTTTCACTTTTGTATGTGGCTGATTCAAGATTAAGTTTTCGTTGTGTTATGGATTTGAGAAAAGATTCTTTTTTTACTTGGGGGGTGCGTTATTGACACATCTGGGGGGTTAGTATTTTGTTTGTCCTGTTTTTTGTTCATATGGGTCGTGCCTTATATTATTCAAGTTATACTAAGAAGGGGGTATGGAAAGTGGGGTTTATTTTATATCTTTTAACTATGGCTGAGGCTTTTTTGGGTTATATTTTACCTTGACATCAAATGTCATATTGGGCTGCTACTGTTTTGACGGCTATTGCCGAGAGTATTCCCTTAGTTGGTCCTACGGTGTTTAAGTATTTGGTGGGGGGGTTTTCTGTAACTAAAGTAACTTTGGTTCGTGTATTTTCAGCTCATGTTTGTTTAGGTTTTGTAATTTTAGGTTTAATGATTTTGCATCTTTTTTATTTGCATTCTTCCGGGTCTAATAACCCTTTATTTTCTTCTTTTGGGTATGGAGATGTTGTTTATTTCCACTCTTATTTTACCACTAAGGATTTTTTTTGTTTGGTTGTTTTATGCTGTATTTTGGTTGGATTTATGTGGTTGGTTCCTGATTTGGTGGTAGATACAGAGGGTTATCTAGAGTCTGATCCTTTGGTGACTCCGGTGTCTATAAAGCCTGAGTGATATTTTTTGATTTATTATGCTATGCTTCGTTCTGTTGAGTCTAAGATAGGTGGTTTAGTGTTGGTGGCTAGATTATTATTTTTTATGTGGGTCCCAACTTTTAAAGACTCTAGTTCATATTTTGTTATTCGACAGGTGGTTTTTTGGGGTTTCGTTTGTCTTTTTGGTGGGTTGACTTATTTAGCCTCATGTCACCCTGAGTATCCTTATTTGGGTATTTGTCAGTTATTTTCGGTTGGTGCTGTAGCTTTTATGTTTATTTATAAGCTATTTTGATCGAGTTATACTAAGTTGGGTTTTAGTATTTTTTTAGGGTAAATGGCTAAGTTTAGTTTTTTTAGTTGGCTTTTTACGCTAGATCATAAGCGTGTGGGTATGATTTATACTTTAATTGGGATATGATCAGGTTTTGTTGGGTTGAGTTTTAGGGTAATGATACGTGTTAATTTCGTTGAGCCTTATTTTAATGTAATTTCTTCGGACTGTTATAAATTTTTGATAACTAATCATGGTATTATTATGATTTTTTTTTTTTTGATCCCGGGGTTGATTGGGGGTTTTGGAAATTATTTAATTCCTTTATTACCCGGTTTACCAAATTTAATTTTACCCCGGTTGAAACCCTTGAAAGCAGGGTGGCTTTTTCCTTCAATTTTATTTCTGGGGCGGAGAATGGGTTGGGGGGCGGGAAAAGGGGGAACTTTTTACCCCCCCCTCTCCCCTTCCCTTTTTAGGGATAGCCGGGGGGTTAATCTTTTGAGGTTTTCTTTACATTTGGCTGGTCTTTCAAGTTTGTTGGGTTCTATAAATTTTATATGTACTCTTTACTCAGCTTTTGTTGATAATTTTGTGTCTCGTAGTTCTATTTTGTTGGGGTCTTATCTATTTACTTCTATCTTTTTATTGTTAACTATTCCTGTGTTGGCTGCTGCCATTACAATGTTGTTGTTTGATCGTAAATTTGGTTCGGCTTTTTTTGATCCTTTGGGTGGTGGTGATCCTGTTTTGTTTCAACATATGTTTTGGTTTTTTGGGCATCCAGAAGTTTATGTATTGATTTTGCCTGGTTTTGGTATGGTAAGTCATGTGTGTAGTAAATTAGGTTGTTCATATGATACTTTTGGTTTTTATGGTTTATTATTTGCTATGTTTTCTATAGTGTGTTTAGGTAGCGTGGTTTGGGGTCACCACATGTTTACTGTGGGGTTGGACGTGAAGACTGCTGTTTTCTTTAGTTCTGTGACTATGATTATTGGGGTTCCCACGGGTATAAAGGTGTTTTCTTGGCTTTATATGATTTTAAATAGTCGTGTTTCGTTGCGTGAGCCTGTGTTTTGATGGGTTTTATCCTTTATTGTGTTGTTTACTATGGGTGGTGTTACTGGTATAATTCTTTCTGCTTGTGTGTTGGATAAAATTTTGCATGACACGTGGTTTGTGGTGGCTCATTTTCATTATGTTATGTCTTTGGGTTCTTATATTAGGGTTATTATATTTTTTGTTTGGTGATGGCCTGTTATCACAGGGGTTAGCTTGAATAAGTATTTGTTACAGTGTCATTGTATAGTATCAAATGTGGGCTTTAATTTGTGTTTTTTTCCTATGCATTATTTTGGTATTTGTGGTTTACCTCGGCGTGTTTGTGTGTATGAGTCAGGGTACGCTTGAGTTAATATGCTTTGTTCAATAGGTTCTTTTGTTTCTGCCTTTAGTGGTTGCTTTTTTATTTTTATTTTATGGGAGTCTTTAGCTAAAAAGAATGTTGTTATAGGTTATTATGGTAGTTCTTCAACTTTGCTTAATTTGTGTTGATCGCCAGTGCCTTACCACAGTAATTTTTTTGTGCGTGGATTATTTGTTGATTATTCTGTATTGGCTTTTTAG

>Hap_47

ATGGTTTTGTTGTTGCGTCGTAATGTGGTTGATTTGCCTACTAATTATTCTCTTAATTATTATTGGTGTAGCGGGTTTATGATTTCGGCTTTTATGGTAGTTCAGGTAATTACTGGTGTGATTCTTTCACTTTTGTATGTGGCTGATTCAAGATTAAGTTTTCGTTGTGTTATGGATTTGAGAAAAGATTCTTTTTTTACTTGGGGGGTGCGTTATTGACACATCTGGGGGGTTAGTATTTTGTTTGTCCTGTTTTTTGTTCATATGGGTCGTGCCTTATATTATTCAAGTTATACTAAGAAGGGGGTATGGAAAGTGGGGTTTATTTTATATCTTTTAACTATGGCTGAGGCTTTTTTGGGTTATATTTTACCTTGACATCAAATGTCATATTGGGCTGCTACTGTTTTGACGGCTATTGCCGAGAGTATTCCCTTAGTTGGTCCTACGGTGTTTAAGTATTTGGTGGGGGGGTTTTCTGTAACTAAAGTAACTTTGGTTCGTGTATTTTCAGCTCATGTTTGTTTAGGTTTTGTAATTTTAGGTTTAATGATTTTGCATCTTTTTTATTTGCATTCTTCCGGGTCTAATAACCCTTTATTTTCTTCTTTTGGGTATGGAGATGTTGTTTATTTCCACTCTTATTTTACCACTAAGGATTTTTTTTGTTTGGTTGTTTTATGCTGTATTTTGGTTGGATTTATGTGGTTGGTTCCTGATTTGGTGGTAGATACAGAGGGTTATCTAGAGTCTGATCCTTTGGTGACTCCGGTGTCTATAAAGCCTGAGTGATATTTTTTGATTTATTATGCTATGCTTCGTTCTGTTGAGTCTAAGATAGGTGGTTTAGTGTTGGTGGCTAGATTATTATTTTTTATGTGGGTCCCAACTTTTAAAGACTCTAGTTCATATTTTGTTATTCGACAGGTGGTTTTTTGGGGTTTCGTTTGTCTTTTTGTTGGGTTGACTTATTTAGGCTCATGTCACCCTGAGTATCCTTATTTGGGTATTTGTCAGTTATTTTCGGTTGGTGCTGTAGCTTTTATGTTTATTTATAAGCTATTTTGATCGAGTTATACTAAGTTGGGTTTTAGTATTTTTTTAGGGTAAATGGCTAAGTTTAGTTTTTTTAGTTGGCTTTTTACGCTAGATCATAAGCGTGTGGGTATGATTTATACTTTAATTGGGATATGATCAGGTTTTGTTGGGTTGAGTTTTAGGGTAATGATACGTGTTAATTTCGTTGAGCCTTATTTTAATGTAATTTCTTCGGACTGTTATAAATTTTTGATAACTAATCATGGTATTATTATGATTTTTTTTTTTTTGATCCCGGGGTTGATGGGGGGTTTGGGAAATTATTTAATCCCTTTATTACCCGGTTTACCAAATTTAAATTTCCCCCGGTTGAAACCCTTGAAAGCAGGGTGGCTTTTTCCTTCTATTTTATTTCTGGGGCTAAGAATGGGTTGGGGGGCGGGAAAAGGGGGAACTTTTTACCCCCCCCTCTCCCCTTCCCTTTTTAGGGATAGCCGGGGGGTTAACCTTTTAAGGTTTTCTTTACATTTGGCTGGTCTTTCAAGTTTGTTGGGTTCTATAAATTTTATATGTACTCTTTACTCAGCTTTTGTTGATAATTTTGTGTCTCGTAGTTCTATTTTGTTGGGGTCTTATCTATTTACTTCTATTCTTTTATTGTTAACTATTCCTGTGTTGGCTGCTGCCATTACAATGTTGTTGTTTGATCGTAAATTTGGTTCGGCTTTTTTTGATCCTTTGGGTGGTGGTGATCCTGTTTTGTTTCAACATATGTTTTGGTTTTTTGGGCATCCAGAAGTTTATGTATTGATTTTGCCTGGTTTTGGTATGGTAAGTCATGTGTGTAGTAAATTAGGTTGTTCATATGATACTTTTGGTTTTTATGGTTTATTATTTGCTATGTTTTCTATAGTGTGTTTAGGTAGCGTGGTTTGGGGTCACCACATGTTTACTGTGGGGTTGGACGTGAAGACTGCTGTTTTCTTTAGTTCTGTGACTATGATTATTGGGGTTCCCACGGGTATAAAGGTGTTTTCTTGGCTTTATATGATTTTAAATAGTCGTGTTTCGTTGCGTGAGCCTGTGTTTTGATGGGTTTTATCCTTTATTGTGTTGTTTACTATGGGTGGTGTTACTGGTATAATTCTTTCTGCTTGTGTGTTGGATAAAATTTTGCATGACACGTGGTTTGTGGTGGCTCATTTTCATTATGTTATGTCTTTGGGTTCTTATATTAGGGTTATTATATTTTTTGTTTGGTGATGGCCTGTTATCACAGGGGTTAGCTTGAATAAGTATTTGTTACAGTGTCATTGTATAGTATCAAATGTGGGCTTTAATTTGTGTTTTTTTCCTATGCATTATTTTGGTATTTGTGGTTTACCTCGGCGTGTTTGTGTGTATGAGTCAGGGTACGCTTGAGTTAATATGCTTTGTTCAATAGGTTCTTTTGTTTCTGCCTTTAGTGGTTGCTTTTTTATTTTTATTTTATGGGAGTCTTTAGCTAAAAAGAATGTTGTTATAGGTTATTATGGTAGTTCTTCAACTTTGCTTAATTTGTGTTGATCGCCAGTGCCTTACCACAGTAATTTTTTTGTGCGTGGATTATTTGTTGATTATTCTGTATTGGCTTTTTAG

>Hap_48

ATGGTTTTGTTGTTGCGTCGTAATGTGGTTGATTTGCCTACTAATTATTCTCTTAATTATTATTGGTGTAGCGGGTTTATGATTTCGGCTTTTATGGTAGTTCAGGTAATTACTGGTGTGATTCTTTCACTTTTGTATGTGGCTGATTCAAGATTAAGTTTTCGTTGTGTTATGGATTTGAGAAAAGATTCTTTTTTTACTTGGGGGGTGCGTTATTGACACATCTGGGGGGTTAGTATTTTGTTTGTCCTGTTTTTTGTTCATATGGGTCGTGCCTTATATTATTCAAGTTATACTAAGAAGGGGGTATGGAAAGTGGGGTTTATTTTATATCTTTTAACTATGGCTGAGGCTTTTTTGGGTTATATTTTACCTTGACATCAAATGTCATATTGGGCTGCTACTGTTTTGACGGCTATTGCCGAGAGTATTCCCTTAGTTGGTCCTACGGTGTTTAAGTATTTGGTGGGGGGGTTTTCTGTAACTAAAGTAACTTTGGTTCGTGTATTTTCAGCTCATGTTTGTTTAGGTTTTGTAATTTTAGGTTTAATGATTTTGCATCTTTTTTATTTGCATTCTTCCGGGTCTAATAACCCTTTATTTTCTTCTTTTGGGTATGGAGATGTTGTTTATTTCCACTCTTATTTTACCACTAAGGATTTTTTTTGTTTGGTTGTTTTATGCTGTATTTTGGTTGGATTTATGTGGTTGGTTCCTGATTTGGTGGTAGATACAGAGGGTTATCTACAGTCTGATCCTTTGGTGACTCCGGTGTCTATAAAGCCTGAGTGATATTTTTTGATTTATTATGCTATGCTTCGTTCTGTTGAGTCTAAGATAGGTGGTTTAGTGTTGGTGGCTAGATTATTATTTTTTATGTGGGTCCCAACTTTTAAAGACTCTAGTTCATATTTTGTTATTCGACAGGTGGTTTTTTGGGGTTTCGTTTGTCTTTTTGTTGGGTTGACTTATTTAGGCTCATGTCACCCTGAGTATCCTTATTTGGGTATTTGTCAGTTATTTTCGGTTGGTGCTGTAGCTTTTATGTTTATTTATAAGCTATTTTGATCGAGTTATACTAAGTTGGGTTTTAGTATTTTTTTAGGGTAAATGGCTAAGTTTAGTTTTTTTAGTTGGCTTTTTACGCTAGATCATAAGCGTGTGGGTATGATTTATACTTTAATTGGGATATGATCAGGTTTTGTTGGGTTGAGTTTTAGGGTAATGATACGTGTTAATTTCGTTGAGCCTTATTTTAATGTAATTTCTTCGGACTGTTATAAATTTTTGATAACTAATCATGGTATTATTATGATTTTTTTTTTTTTGATGCCGGGGTTGATGGGGGGTTTGGGAAATTATTTGATTCCTTTATTACCCGGTTTACCAAATTTAAATTTCCCCCGGTTGAAACCCTTGAAAGCAGGGTGGCTTTTTCCTTCAATTTTATTTCTGGGGCGGAGAATGGGTTGGGGGGCGGGAAAAGGGGGAACTTTTTACCCCCCTCTCTCCCCTTCCCTTTTTAGGGATAGCCGGGGGGTTAATCTTTTGAGGTTTTCTTTACATTTGGCTGGTCTTTCAAGTTTGTTGGGTTCTATAAATTTTATATGTACTCTTTACTCAGCTTTTGTTGATAATTTTGTGTCTCGTAGTTCTATTTTGTTGGGGTCTTATCTATTTACTTCTATCTTTTTATTGTTAACTATTCCTGTGTTGGCTGCTGCCATTACAATGTTGTTGTTTGATCGTAAATTTGGTTCGGCTTTTTTTGATCCTTTGGGTGGTGGTGATCCTGTTTTGTTTCAACATATGTTTTGGTTTTTTGGGCATCCAGAAGTTTATGTATTGATTTTGCCTGGTTTTGGTATGGTAAGTCATGTGTGTAGTAAATTAGGTTGTTCATATGATACTTTTGGTTTTTATGGTTTATTATTTGCTATGTTTTCTATAGTGTGTTTAGGTAGCGTGGTTTGGGGTCACCACATGTTTACTGTGGGGTTGGACGTGAAGACTGCTGTTTTCTTTAGTTCTGTGACTATGATTATTGGGGTTCCCACGGGTATAAAGGTGTTTTCTTGGCTTTATATGATTTTAAATAGTCGTGTTTCGTTGCGTGAGCCTGTGTTTTGATGGGTTTTATCCTTTATTGTGTTGTTTACTATGGGTGGTGTTACTGGTATAATTCTTTCTGCTTGTGTGTTGGATAAAATTTTGCATGACACGTGGTTTGTGGTGGCTCATTTTCATTATGTTATGTCTTTGGGTTCTTATATTAGGGTTATTATATTTTTTGTTTGGTGATGGCCTGTTATCACAGGGGTTAGCTTGAATAAGTATTTGTTACAGTGTCATTGTATAGTATCAAATGTGGGCTTTAATTTGTGTTTTTTTCCTATGCATTATTTTGGTATTTGTGGTTTACCTCGGCGTGTTTGTGTGTATGAGTCAGGGTACGCTTGAGTTAATATGCTTTGTTCAATAGGTTCTTTTGTTTCTGCCTTTAGTGGTTGCTTTTTTATTTTTATTTTATGGGAGTCTTTAGCTAAAAAGAATGTTGTTATAGGTTATTATGGTAGTTCTTCAACTTTGCTTAATTTGTGTTGATCGCCAGTGCCTTACCACAGTAATTTTTTTGTGCGTGGATTATTTGTTGATTATTCTGTATTGGCTTTTTAG

>Hap_49

ATGGTTTTGTTGTTGCGTCGTAATGTGGTTGATTTGCCTACTAATTATTCTCTTAATTATTATTGGTGTAGCGGGTTTATGATTTCGGCTTTTATGGTAGTTCAGGTAATTACTGGTGTGATTCTTTCACTTTTGTATGTGGCTGATTCAAGATTAAGTTTTCGTTGTGTTATGGATTTGAGAAAAGATTCTTTTTTTACTTGGGGGGTGCGTTATTGACACATCTGGGGGGTTAGTATTTTGTTTGTCCTGTTTTTTGTTCATATGGGTCGTGCCTTATATTATTCAAGTTATACTAAGAAGGGGGTATGGAAAGTGGGGTTTATTTTATATCTTTTAACTATGGCTGAGGCTTTTTTGGGTTATATTTTACCTTGACATCAAATGTCATATTGGGCTGCTACTGTTTTGACGGCTATTGCCGAGAGTATTCCCTTAGTTGGTCCTACGGTGTTTAAGTATTTGGTGGGGGGGTTTTCTGTAACTAAAGTAACTTTGGTTCGTGTATTTTCAGCTCATGTTTGTTTAGGTTTTGTAATTTTAGGTTTAATGATTTTGCATCTTTTTTATTTGCATTCTTCCGGGTCTAATAACCCTTTATTTTCTTCTTTTGGGTATGGAGATGTTGTTTATTTCCACTCTTATTTTACCACTAAGGATTTTTTTTGTTTGGTTGTTTTATGCTGTATTTTGGTTGGATTTATGTGGTTGGTTCCTGATTTGGTGGTAGATACAGAGGGTTATCTAGAGTCTGATCCTTTGGTGACTCCGGTGTCTATAAAGCCTGAGTGATATTTTTTGATTTATTATGCTATGCTTCGTTCTGTTGAGTCTAAGATAGGTGGTTTAGTGTTGGTGGCTAGATTATTATTTTTTATGTGGGTCCCAACTTTTAAAGACTCTAGTTCATATTTTGTTATTCGACAGGTGGTTTTTTGGGGTTTCGTTTGTCTTTTTGTTGGGTTGACTTATTTAGGCTCATGTCACCCTGAGTATCCTTATTTGGGTATTTGTCAGTTATTTTCGGTTGGTGCTGTAGCTTTTATGTTTATTTATAAGCTATTTTGATCGAGTTATACTAAGTTGGGTTTTAGTATTTTTTTAGGGTAAATGGCTAAGTTTAGTTTTTTTAGTTGGCTTTTTACGCTAGATCATAAGCGTGTGGGTATGATTTATACTTTAATTGGGATATGATCAGGTTTTGTTGGGTTGAGTTTTAGGGTAATGATACGTGTTAATTTCGTTGAGCCTTATTTTAATGTAATTTCTTCGGACTGTTATAAATTTTTGATAACTAATCATGGTATTATTATGATTTTTTTTTTTTTGATCCCGGGGTTGATGGGGGGTTTTGGAAATTATTTAATTCCTTTATTACCCGGTTTACCAAATTTAAATTTACCCCGGTTGAAACCCTTGAAAGCAGGGTTGCTTTTTCCTTCTATTTTATTTCTGGGGCTAAATATGGGTTTGGGGGCGGGTAAAGGGGGAACTTTTTACCCCCCTCTCTCCCCTTCCCTTTTTAGGGATAGCCGGGGGGTTAACCTTTTAATGTTTTCTTTACATTTGGCTGGTCTTTCAAGTTTGTTGGGTTCTATAAATTTTATATGTACTCTTTACTCAGCTTTTGTTGATAATTTTGTGTCTCGTAGTTCTATTTTGTTGGGGTCTTATCTATTTACTTCTATCCTTTTATTGTTAACTATTCCTGTGTTGGCTGCTGCCATTACAATGTTGTTGTTTGATCGTAAATTTGGTTCGGCTTTTTTTGATCCTTTGGGTGGTGGTGATCCTGTTTTGTTTCAACATATGTTTTGGTTTTTTGGGCATCCAGAAGTTTATGTATTGATTTTGCCTGGTTTTGGTATGGTAAGTCATGTGTGTAGTAAATTAGGTTGTTCATATGATACTTTTGGTTTTTATGGTTTATTATTTGCTATGTTTTCTATAGTGTGTTTAGGTAGCGTGGTTTGGGGTCACCACATGTTTACTGTGGGGTTGGACGTGAAGACTGCTGTTTTCTTTAGTTCTGTGACTATGATTATTGGGGTTCCCACGGGTATAAAGGTGTTTTCTTGGCTTTATATGATTTTAAATAGTCGTGTTTCGTTGCGTGAGCCTGTGTTTTGATGGGTTTTATCCTTTATTGTGTTGTTTACTATGGGTGGTGTTACTGGTATAATTCTTTCTGCTTGTGTGTTGGATAAAATTTTGCATGACACGTGGTTTGTGGTGGCTCATTTTCATTATGTTATGTCTTTGGGTTCTTATATTAGGGTTATTATATTTTTTGTTTGGTGATGGCCTGTTATCACAGGGGTTAGCTTGAATAAGTATTTGTTACAGTGTCATTGTATAGTATCAAATGTGGGCTTTAATTTGTGTTTTTTTCCTATGCATTATTTTGGTATTTGTGGTTTACCTCGGCGTGTTTGTGTGTATGAGTCAGGGTACGCTTGAGTTAATATGCTTTGTTCAATAGGTTCTTTTGTTTCTGCCTTTAGTGGTTGCTTTTTTATTTTTATTTTATGGGAGTCTTTAGCTAAAAAGAATGTTGTTATAGGTTATTATGGTAGTTCTTCAACTTTGCTTAATTTGTGTTGATCGCCAGTGCCTTACCACAGTAATTTTTTTGTGCGTGGATTATTTGTTGATTATTCTGTATTGGCTTTTTAG

>Hap_50

ATGGTTTTGTTGTTGCGTCGTAATGTGGTTGATTTGCCTACTAATTATTCTCTTAATTATTATTGGTGTAGCGGGTTTATGATTTCGGCTTTTATGGTAGTTCAGGTAATTACTGGTGTGATTCTTTCACTTTTGTATGTGGCTGATTCAAGATTAAGTTTTCGTTGTGTTATGGATTTGAGAAAAGATTCTTTTTTTACTTGGGGGGTGCGTTATTGACACATCTGGGGGGTTAGTATTTTGTTTGTCCTGTTTTTTGTTCATATGGGTCGTGCCTTATATTATTCAAGTTATACTAAGAAGGGGGTATGGAAAGTGGGGTTTATTTTATATCTTTTAACTATGGCTGAGGCTTTTTTGGGTTATATTTTACCTTGACATCAAATGTCATATTGGGCTGCTACTGTTTTGACGGCTATTGCCGAGAGTATTCCCTTAGTTGGTCCTACGGTGTTTAAGTATTTGGTGGGGGGGTTTTCTGTAACTAAAGTAACTTTGGTTCGTGTATTTTCAGCTCATGTTTGTTTAGGTTTTGTAATTTTAGGTTTAATGATTTTGCATCTTTTTTATTTGCATTCTTCCGGGTCTAATAACCCTTTATTTTCTTCTTTTGGGTATGGAGATGTTGTTTATTTCCACTCTTATTTTACCACTAAGGATTTTTTTTGTTTGGTTGTTTTATGCTGTATTTTGGTTGGATTTATGTGGTTGGTTCCTGATTTGGTGGTAGATACAGAGGGTTATCTAGAGTCTGATCCTTTGGTGACTCCGGTGTCTATAAAGCCTGAGTGATATTTTTTGATTTATTATGCTATGCTTCGTTCTGTTGAGTCTAAGATAGGTGGTTTAGTGTTGGTGGCTAGATTATTATTTTTTATGTGGGTCCCAACTTTTAAAGACTCTAGTTCATATTTTGTTATTCGACAGGTGGTTTTTTGGGGTTTCGTTTGTCTTTTTGTTGGGTTGACTTATTTAGGCTCATGTCACCCTGAGTATCCTTATTTGGGTATTTGTCAGTTATTTTCGGTTGGTGCTGTAGCTTTTATGTTTATTTATAAGCTATTTTGATCGAGTTATACTAAGTTGGGTTTTAGTATTTTTTTAGGGTAAATGGCTAAGTTTAGTTTTTTTAGTTGGCTTTTTACGCTAGATCATAAGCGTGTGGGTATGATTTATACTTTAATTGGGATATGATCAGGTTTTGTTGGGTTGAGTTTTAGGGTAATGATACGTGTTAATTTTGTTGAGCCTTATTTTAATGTAATTTCTTCGGACTGTTATAAATTTTTGATAACTAATCATGGTATTATTATGATTTTTTTTTTTTTGATGCCGGGGTTGATGGGGGGTTTTGGAAATTATTTAATTCCTTTATTACCCGGTTTACCAAATTTAAATTTACCCCGGTTGAAACCCTTGAAAGCAGGGTTGCTTTTTCCTTCTATTTTATTTCTGGGGCTAAATATGGGTTTGGGGGCGGGTAAAGGGGGAACTTTTTACCCCCCTCTCTCCCCTTCCCTTTTTAGGGATAGCCGGGGGGTTAACCTTTTAATGTTTTCTTTACATTTGGCTGGTCTTTCAAGTTTGTTGGGTTCTATAAATTTTATATGTACTCTTTACTCAGCTTTTGTTGATAATTTTGTGTCTCGTAGTTCTATTTTGTTGGGGTCTTATCTATTTACTTCTATCCTTTTATTGTTAACTATTCCTGTGTTGGCTGCTGCCATTACAATGTTGTTGTTTGATCGTAAATTTGGTTCGGCTTTTTTTGATCCTTTGGGTGGTGGTGATCCTGTTTTGTTTCAACATATGTTTTGGTTTTTTGGGCATCCAGAAGTTTATGTATTGATTTTGCCTGGTTTTGGTATGGTAAGTCATGTGTGTAGTAAATTAGGTTGTTCATATGATACTTTTGGTTTTTATGGTTTATTATTTGCTATGTTTTCTATAGTGTGTTTAGGTAGCGTGGTTTGGGGTCACCACATGTTTACTGTGGGGTTGGACGTGAAGACTGCTGTTTTCTTTAGTTCTGTGACTATGATTATTGGGGTTCCCACGGGTATAAAGGTGTTTTCTTGGCTTTATATGATTTTAAATAGTCGTGTTTCGTTGCGTGAGCCTGTGTTTTGATGGGTTTTATCCTTTATTGTGTTGTTTACTATGGGTGGTGTTACTGGTATAATTCTTTCTGCTTGTGTGTTGGATAAAATTTTGCATGACACGTGGTTTGTGGTGGCTCATTTTCATTATGTTATGTCTTTGGGTTCTTATATTAGGGTTATTATATTTTTTGTTTGGTGATGGCCTGTTATCACAGGGGTTAGCTTGAATAAGTATTTGTTACAGTGTCATTGTATAGTATCAAATGTGGGCTTTAATTTGTGTTTTTTTCCTATGCATTATTTTGGTATTTGTGGTTTACCTCGGCGTGTTTGTGTGTATGAGTCAGGGTACGCTTGAGTTAATATGCTTTGTTCAATAGGTTCTTTTGTTTCTGCCTTTAGTGGTTGCTTTTTTATTTTTATTTTATGGGAGTCTTTAGCTAAAAAGAATGTTGTTATAGGTTATTATGGTAGTTCTTCAACTTTGCTTAATTTGTGTTGATCGCCAGTGCCTTACCACAGTAATTTTTTTGTGCGTGGATTATTTGTTGATTATTCTGTATTGGCTTTTTAG

>Hap_51

ATGGTTTTGTTGTTGCGTCGTAATGTGGTTGATTTGCCTACTAATTATTCTCTTAATTATTATTGGTGTAGCGGGTTTATGATTTCGGCTTTTATGGTAGTTCAGGTAATTACTGGTGTGATTCTTTCACTTTTGTATGTGGCTGATTCAAGATTAAGTTTTCGTTGTGTTATGGATTTGAGAAAAGATTCTTTTTTTACTTGGGGGGTGCGTTATTGACACATCTGGGGGGTTAGTATTTTGTTTGTCCTGTTTTTTGTTCATATGGGTCGTGCCTTATATTATTCAAGTTATACTAAGAAGGGGGTATGGAAAGTGGGGTTTATTTTATATCTTTTAACTATGGCTGAGGCTTTTTTGGGTTATATTTTACCTTGACATCAAATGTCATATTGGGCTGCTACTGTTTTGACGGCTATTGCCGAGAGTATTCCCTTAGTTGGTCCTACGGTGTTTAAGTATTTGGTGGGGGGGTTTTCTGTAACTAAAGTAACTTTGGTTCGTGTATTTTCAGCTCATGTTTGTTTAGGTTTTGTAATTTTAGGTTTAATGATTTTGCATCTTTTTTATTTGCATTCTTCCGGGTCTAATAACCCTTTATTTTCTTCTTTTGGGTATGGAGATGTTGTTTATTTCCACTCTTATTTTACCACTAAGGATTTTTTTTGTTTGGTTGTTTTATGCTGTATTTTGGTTGGATTTATGTGGTTGGTTCCTGATTTGGTGGTAGATACAGAGGGTTATCTACAGTCTGATCCTTTGGTGACTCCGGTGTCTATAAAGCCTGAGTGATATTTTTTGATTTATTATGCTATGCTTCCTTCTGTTGAGTCTAAGATAGGTGGTTTAGTGTTGGTGGCTAGATTATTATTTTTTATGTGGGTCCCAACTTTTAAAGACTCTAGTTCATATTTTGTTATTCGACAGGTGGTTTTTTGGGGTTTCGTTTGTCTTTTTGTTGGGTTGACTTATTTAGGCTCATGTCACCCTGAGTATCCTTATTTGGGTATTTGTCAGTTATTTTCGGTTGGTGCTGTAGCTTTTATGTTTATTTATAAGCTATTTTGATCGAGTTATACTAAGTTGGGTTTTAGTATTTTTTTAGGGTAAATGGCTAAGTTTAGTTTTTTTAGTTGGCTTTTTACGCTAGATCATAAGCGTGTGGGTATGATTTATACTTTAATTGGGATATGATCAGGTTTTGTTGGGTTGAGTTTTAGGGTAATGATACGTGTTAATTTCGTTGAGCCTTATTTTAATGTAATTTCTTCGGACTGTTATAAATTTTTGATAACTAATCATGGTATTATTATGATTTTTTTTTTTTTGATGCCGGTGTTGATGGGGGGTTTTGGAAATTATTTGATTCCTTTATTACCCGGTTTACCAAATTTAAATTTACCCCGGTTGAAACCCTTGAAAGCAGGGTTGCTTTTTCCTTCTATTTTATTTCTGGGGCTGAGTATGTGTTTGGGGGCGGGTATAGGGGGAACTTTTTATCCCCCCCTCTCCTCTTCCCTTTTTAGGAATAGCCGGGGTGTTAATCTTTTGATGTTTTCTTTACTTTTGGCTGGCCTTTCAAGTTTGTTGGGTTCTATAAATTTTATATGTACTCTTTACTCAGCTTTTGTTGATAATTTTGTGTCTCGTAGTTCTATTTTGTTGGGGTCTTATCTATTTACTTCTATTCTTTTATTGTTAACTATTCCTGTGTTGGCTGCTGCCATTACAATGTTGTTGTTTGATCGTAAATTTGGTTCGGCTTTTTTTGATCCTTTGGGTGGTGGTGATCCTGTTTTGTTTCAACATATGTTTTGGTTTTTTGGGCATCCAGAAGTTTATGTATTGATTTTGCCTGGTTTTGGTATGGTAAGTCATGTGTGTAGTAAATTAGGTTGTTCATATGATACTTTTGGTTTTTATGGTTTATTATTTGCTATGTTTTCTATAGTGTGTTTAGGTAGCGTGGTTTGGGGTCACCACATGTTTACTGTGGGGTTGGACGTGAAGACTGCTGTTTTCTTTAGTTCTGTGACTATGATTATTGGGGTTCCCACGGGTATAAAGGTGTTTTCTTGGCTTTATATGATTTTAAATAGTCGTGTTTCGTTGCGTGAGCCTGTGTTTTGATGGGTTTTATCCTTTATTGTGTTGTTTACTATGGGTGGTGTTACTGGTATAATTCTTTCTGCTTGTGTGTTGGATAAAATTTTGCATGACACGTGGTTTGTGGTGGCTCATTTTCATTATGTTATGTCTTTGGGTTCTTATATTAGGGTTATTATATTTTTTGTTTGGTGATGGCCTGTTATCACAGGGGTTAGCTTGAATAAGTATTTGTTACAGTGTCATTGTATAGTATCAAATGTGGGCTTTAATTTGTGTTTTTTTCCTATGCATTATTTTGGTATTTGTGGTTTACCTCGGCGTGTTTGTGTGTATGAGTCAGGGTACGCTTGAGTTAATATGCTTTGTTCAATAGGTTCTTTTGTTTCTGCCTTTAGTGGTTGCTTTTTTATTTTTATTTTATGGGAGTCTTTAGCTAAAAAGAATGTTGTTATAGGTTATTATGGTAGTTCTTCAACTTTGCTTAATTTGTGTTGATCGCCAGTGCCTTACCACAGTAATTTTTTTGTGCGTGGATTATTTGTTGATTATTCTGTATTGGCTTTTTAG

>Hap_52

ATGGTTTTGTTGTTGCGTCGTAATGTGGTTGATTTGCCTACTAATTATTCTCTTAATTATTATTGGTGTAGCGGGTTTATGATTTCGGCTTTTATGGTAGTTCAGGTAATTACTGGTGTGATTCTTTCACTTTTGTATGTGGCTGATTCAAGATTAAGTTTTCGTTGTGTTATGGATTTGAGAAAAGATTCTTTTTTTACTTGGGGGGTGCGTTATTGACACATCTGGGGGGTTAGTATTTTGTTTGTCCTGTTTTTTGTTCATATGGGTCGTGCCTTATATTATTCAAGTTATACTAAGAAGGGGGTATGGAAAGTGGGGTTTATTTTATATCTTTTAACTATGGCTGAGGCTTTTTTGGGTTATATTTTACCTTGACATCAAATGTCATATTGGGCTGCTACTGTTTTGACGGCTATTGCCGAGAGTATTCCCTTAGTTGGTCCTACGGTGTTTAAGTATTTGGTGGGGGGGTTTTCTGTAACTAAAGTAACTTTGGTTCGTGTATTTTCAGCTCATGTTTGTTTAGGTTTTGTAATTTTAGGTTTAATGATTTTGCATCTTTTTTATTTGCATTCTTCCGGGTCTAATAACCCTTTATTTTCTTCTTTTGGGTATGGAGATGTTGTTTATTTCCACTCTTATTTTACCACTAAGGATTTTTTTTGTTTGGTTGTTTTATGCTGTATTTTGGTTGGATTTATGTGGTTGGTTCCTGATTTGGTGGTAGATACAGAGGGTTATCTAGAGTCTGATCCTTTGGTGACTCCGGTGTCTATAAAGCCTGAGTGATATTTTTTGATTTATTATGCTATGCTTCGTTCTGTTGAGTCTAAGATAGGTGGTTTAGTGTTGGTGGCTAGATTATTATTTTTTATGTGGGTCCCAACTTTTAAAGACTCTAGTTCATATTTTGTTATTCGACAGGTGGTTTTTTGGGGTTTCGTTTGTCTTTTTGTTGGGTTGACTTATTTAGGCTCATGTCACCCTGAGTACCCTTATTTGGGTATTTGTCAGTTATTTTCGGTTGGTGCTGTAGCTTTTATGTTTATTTATAAGCTATTTTGATCGAGTTATACTAAGTTGGGTTTTAGTATTTTTTTAGGGTAAATGGCTAAGTTTAGTTTTTTTAGTTGGCTTTTTACGCTAGATCATAAGCGTGTGGGTATGATTTATACTTTAATTGGGATATGATCAGGTTTTGTTGGGTTGAGTTTTAGGGTAATGATACGTGTTAATTTCGTTGAGCCTTATTTTAATGTAATTTCTTCGGACTGTTATAAATTTTTGATAACTAATCATGGTATTATTATGATTTTTTTTTTTTTGAGGCCGGGGTTGATGGGGGGTTTGGGAAATTATTTGATTCCTTTATTACCCGGTTTACCAAATTTAAATTTCCCCCGGTTAAAACCCTTGAAAGCAGGGTGGCTTTTTCCTTCTATTTTATTTCTGGGGCTAAAAATGTGTTGGGGGGCGGGAAAAGGGGGAACTTTTTATCCCCCTCTCTCCCCTTCCCTTTTTAGGGATAGCCGGGGGGTTAATCTTTTGAGGTTTTCTTTACTTTTGGCTGGCCTTTCTAGTTTGTTGGGTTCTATAAATTTTATATGTACTCTTTACTCAGCTTTTGTTGATAATTTTGTGTCTCGTAGTTCTATTTTGTTGGGGTCTTATCTATTTACTTCTATTCTTTTATTGTTAACTATTCCTGTGTTGGCTGCTGCCATTACAATGTTGTTGTTTGATCGTAAATTTGGTTCGGCTTTTTTTGATCCTTTGGGTGGTGGTGATCCTGTTTTGTTTCAACATATGTTTTGGTTTTTTGGGCATCCAGAAGTTTATGTATTGATTTTGCCTGGTTTTGGTATGGTAAGTCATGTGTGTAGTAAATTAGGTTGTTCATATGATACTTTTGGTTTTTATGGTTTATTATTTGCTATGTTTTCTATAGTGTGTTTAGGTAGCGTGGTTTGGGGTCACCACATGTTTACTGTGGGGTTGGACGTGAAGACTGCTGTTTTCTTTAGTTCTGTGACTATGATTATTGGGGTTCCCACGGGTATAAAGGTGTTTTCTTGGCTTTATATGATTTTAAATAGTCGTGTTTCGTTGCGTGAGCCTGTGTTTTGATGGGTTTTATCCTTTATTGTGTTGTTTACTATGGGTGGTGTTACTGGTATAATTCTTTCTGCTTGTGTGTTGGATAAAATTTTGCATGACACGTGGTTTGTGGTGGCTCATTTTCATTATGTTATGTCTTTGGGTTCTTATATTAGGGTTATTATATTTTTTGTTTGGTGATGGCCTGTTATCACAGGGGTTAGCTTGAATAAGTATTTGTTACAGTGTCATTGTATAGTATCAAATGTGGGCTTTAATTTGTGTTTTTTTCCTATGCATTATTTTGGTATTTGTGGTTTACCTCGGCGTGTTTGTGTGTATGAGTCAGGGTACGCTTGAGTTAATATGCTTTGTTCAATAGGTTCTTTTGTTTCTGCCTTTAGTGGTTGCTTTTTTATTTTTATTTTATGGGAGTCTTTAGCTAAAAAGAATGTTGTTATAGGTTATTATGGTAGTTCTTCAACTTTGCTTAATTTGTGTTGATCGCCAGTGCCTTACCACAGTAATTTTTTTGTGCGTGGATTATTTGTTGATTATTCTGTATTGGCTTTTTAG

>Hap_53

ATGGTTTTGTTGTTGCGTCGTAATGTGGTTGATTTGCCTACTAATTATTCTCTTAATTATTATTGGTGTAGCGGGTTTATGATTTCGGCTTTTATGGTAGTTCAGGTAATTACTGGTGTGATTCTTTCACTTTTGTATGTGGCTGATTCAAGATTAAGTTTTCGTTGTGTTATGGATTTGAGAAAAGATTCTTTTTTTACTTGGGGGGTGCGTTATTGACACATCTGGGGGGTTAGTATTTTGTTTGTCCTGTTTTTTGTTCATATGGGTCGTGCCTTATATTATTCAAGTTATACTAAGAAGGGGGTATGGAAAGTGGGGTTTATTTTATATCTTTTAACTATGGCTGAGGCTTTTTTGGGTTATATTTTACCTTGACATCAAATGTCATATTGGGCTGCTACTGTTTTGACGGCTATTGCCGAGAGTATTCCCTTAGTTGGTCCTACGGTGTTTAAGTATTTGGTGGGGGGGTTTTCTGTAACTAAAGTAACTTTGGTTCGTGTATTTTCAGCTCATGTTTGTTTAGGTTTTGTAATTTTAGGTTTAATGATTTTGCATCTTTTTTATTTGCATTCTTCCGGGTCTAATAACCCTTTATTTTCTTCTTTTGGGTATGGAGATGTTGTTTATTTCCACTCTTATTTTACCACTAAGGATTTTTTTTGTTTGGTTGTTTTATGCTGTATTTTGGTTGGATTTATGTGGTTGGTTCCTGATTTGGTGGTAGATACAGAGGGTTATCTAGAGTCTGATCCTTTGGTGACTCCGGTGTCTATAAAGCCTGAGTGATATTTTTTGATTTATTATGCTATGCTTCGTTCTGTTGAGTCTAAGATAGGTGGTTTAGTGTTGGTGGCTAGATTATTATTTTTTATGTGGGTCCCAACTTTTAAAGACTCTAGTTCATATTTTGTTATTCGACAGGTGGTTTTTTGGGGTTTCGTTTGTCTTTTTGTTGGGTTGACTTATTTAGGCTCATGTCACCCTGAGTATCCTTATTTGGGTATTTGTCAGTTATTTTCGGTTGGTGCTGTAGCTTTTATGTTTATTTATAAGCTATTTTGATCGAGTTATACTAAGTTGGGTTTTAGTATTTTTTTAGGGTAAATGGCTAAGTTTAGTTTTTTTAGTTGGCTTTTTACGCTAGATCATAAGCGTGTGGGTATGATTTATACTTTAATTGGGATATGATCAGGTTTTGTTGGGTTGAGTTTTAGGGTAATGATACGTGTTAATTTTGTTGAGCCTTATTTTAATGTAATTTCTTCGGACTGTTATAAATTTTTGATAACTAATCATGGTATTATTATGATTTTTTTTTTTTTGATGCCGGGGTTGATGGGGGGTTTTGGAAATTATTTAATTCCTTTATTACCCGGTTTACCAAATTTAAATTTACCCCGGTTGAAACCCTTGAAAGCAGGGTTGCTTTTTCCTTCTATTTTATTTCTGGGGCTAAATATGGGTTTGGGGGCGGGTAAAGGGGGAACTTTTTACCCCCCTCTCTCCCCTTCCCTTTTTAGGGATAGCCGGGGGGTTAACCTTTTAATGTTTTCTTTACATTTGGCTGGTCTTTCAAGTTTGTTGGGTTCTATAAATTTTATATGTACTCTTTACTCAGCTTTTGTTGATAATTTTGTGTCTCGTAGTTCTATTTTGTTGGGGTCTTATCTATTTACTTCTATTCTTTTATTGTTAACTATTCCTGTGTTGGCTGCTGCCATTACAATGTTGTTGTTTGATCGTAAATTTGGTTCGGCTTTTTTTGATCCTTTGGGTGGTGGTGATCCTGTTTTGTTTCAACATATGTTTTGGTTTTTTGGGCATCCAGAAGTTTATGTATTGATTTTGCCTGGTTTTGGTATGGTAAGTCATGTGTGTAGTAAATTAGGTTGTTCATATGATACTTTTGGTTTTTATGGTTTATTATTTGCTATGTTTTCTATAGTGTGTTTAGGTAGCGTGGTTTGGGGTCACCACATGTTTACTGTGGGGTTGGACGTGAAGACTGCTGTTTTCTTTAGTTCTGTGACTATGATTATTGGGGTTCCCACGGGTATAAAGGTGTTTTCTTGGCTTTATATGATTTTAAATAGTCGTGTTTCGTTGCGTGAGCCTGTGTTTTGATGGGTTTTATCCTTTATTGTGTTGTTTACTATGGGTGGTGTTACTGGTATAATTCTTTCTGCTTGTGTGTTGGATAAAATTTTGCATGACACGTGGTTTGTGGTGGCTCATTTTCATTATGTTATGTCTTTGGGTTCTTATATTAGGGTTATTATATTTTTTGTTTGGTGATGGCCTGTTATCACAGGGGTTAGCTTGAATAAGTATTTGTTACAGTGTCATTGTATAGTATCAAATGTGGGCTTTAATTTGTGTTTTTTTCCTATGCATTATTTTGGTATTTGTGGTTTACCTCGGCGTGTTTGTGTGTATGAGTCAGGGTACGCTTGAGTTAATATGCTTTGTTCAATAGGTTCTTTTGTTTCTGCCTTTAGTGGTTGCTTTTTTATTTTTATTTTATGGGAGTCTTTAGCTAAAAAGAATGTTGTTATAGGTTATTATGGTAGTTCTTCAACTTTGCTTAATTTGTGTTGATCGCCAGTGCCTTACCACAGTAATTTTTTTGTGCGTGGATTATTTGTTGATTATTCTGTATTGGCTTTTTAG

>Hap_54

ATGGTTTTGTTGTTGCGTCGTAATGTGGTTGATTTGCCTACTAATTATTCTCTTAATTATTATTGGTGTAGCGGGTTTATGATTTCGGCTTTTATGGTAGTTCAGGTAATTACTGGTGTGATTCTTTCACTTTTGTATGTGGCTGATTCAAGATTAAGTTTTCGTTGTGTTATGGATTTGAGAAAAGATTCTTTTTTTACTTGGGGGGTGCGTTATTGACACATCTGGGGGGTTAGTATTTTGTTTGTCCTGTTTTTTGTTCATATGGGTCGTGCCTTATATTATTCAAGTTATACTAAGAAGGGGGTATGGAAAGTGGGGTTTATTTTATATCTTTTAACTATGGCTGAGGCTTTTTTGGGTTATATTTTACCTTGACATCAAATGTCATATTGGGCTGCTACTGTTTTGACGGCTATTGCCGAGAGTATTCCCTTAGTTGGTCCTACGGTGTTTAAGTATTTGGTGGGGGGGTTTTCTGTAACTAAAGTAACTTTGGTTCGTGTATTTTCAGCTCATGTTTGTTTAGGTTTTGTAATTTTAGGTTTAATGATTTTGCATCTTTTTTATTTGCATTCTTCCGGGTCTAATAACCCTTTATTTTCTTCTTTTGGGTATGGAGATGTTGTTTATTTCCACTCTTATTTTACCACTAAGGATTTTTTTTGTTTGGTTGTTTTATGCTGTATTTTGGTTGGATTTATGTGGTTGGTTCCTGATTTGGTGGTAGATACAGAGGGTTATCTAGAGTCTGATCCTTTGGTGACTCCGGTGTCTATAAAGGCTGACTGATATTTTTTGATTTATTATGCTATGCTTCGTTCTGTTGAGTCTAAGATAGGTGGTTTAGTGTTGGTGGCTAGATCATTATTTTTTATGTGGGTCCCAACTTTTAAAGACTCTAGTTCATATTTTGTTATTCGACAGGTGGTTTTTTGGGGTTTCGTTTGTCTTTTTGTTGGGTTGACTTATTTAGGCTCATGTCACCCTGAGTATCCTTATTTGGGTATTTGTCAGTTATTTTCGGTTGGTGCTGTAGCTTTTATGTTTATTTATAAGCTATTTTGATCGAGTTATACTAAGTTGGGTTTTAGTATTTTTTTAGGGTAAATGGCTAAGTTTAGTTTTTTTAGTTGGCTTTTTACGCTAGATCATAAGCGTGTGGGTATGATTTATACTTTAATTGGGATATGATCAGGTTTTGTTGGGTTGAGTTTTAGGGTAATGATACGTGTTAATTTCGTTGAGCCTTATTTTAATGTAATTTCTTCGGACTGTTATAAATTTTTGATAACTAATCATGGTATTATTATGATTTTTTTTTTTTTGAGGCCGGGGTTGATGGGGGGTTTGGGAAATTATTTGATTCCTTTATTACCCGGTTTACCAAATTTAAATTTCCCCCGGTTAAAACCCTTGAAAGCAGGGTGGCTTTTTCCTTCTATTTTATTTCTGGGGCTAAAAATGTGTTGGGGGGCGGGAAAAGGGGGAACTTTTTATCCCCCTCTCTCCCCTTCCCTTTTTAGGGATAGCCGGGGGGTTAATCTTTTGAGGTTTTCTTTACTTTTGGCTGGCCTTTCTAGTTTGTTGGGTTCTATAAATTTTATATGTACTCTTTACTCAGCTTTTGTTGATAATTTTGTGTCTCGTAGTTCTATTTTGTTGGGGTCTTATCTATTTACTTCTATTCTTTTATTGTTAACTATTCCTGTGTTGGCTGCTGCCATTACAATGTTGTTGTTTGATCGTAAATTTGGTTCGGCTTTTTTTGATCCTTTGGGTGGTGGTGATCCTGTTTTGTTTCAACATATGTTTTGGTTTTTTGGGCATCCAGAAGTTTATGTATTGATTTTGCCTGGTTTTGGTATGGTAAGTCATGTGTGTAGTAAATTAGGTTGTTCATATGATACTTTTGGTTTTTATGGTTTATTATTTGCTATGTTTTCTATAGTGTGTTTAGGTAGCGTGGTTTGGGGTCACCACATGTTTACTGTGGGGTTGGACGTGAAGACTGCTGTTTTCTTTAGTTCTGTGACTATGATTATTGGGGTTCCCACGGGTATAAAGGTGTTTTCTTGGCTTTATATGATTTTAAATAGTCGTGTTTCGTTGCGTGAGCCTGTGTTTTGATGGGTTTTATCCTTTATTGTGTTGTTTACTATGGGTGGTGTTACTGGTATAATTCTTTCTGCTTGTGTGTTGGATAAAATTTTGCATGACACGTGGTTTGTGGTGGCTCATTTTCATTATGTTATGTCTTTGGGTTCTTATATTAGGGTTATTATATTTTTTGTTTGGTGATGGCCTGTTATCACAGGGGTTAGCTTGAATAAGTATTTGTTACAGTGTCATTGTATAGTATCAAATGTGGGCTTTAATTTGTGTTTTTTTCCTATGCATTATTTTGGTATTTGTGGTTTACCTCGGCGTGTTTGTGTGTATGAGTCAGGGTACGCTTGAGTTAATATGCTTTGTTCAATAGGTTCTTTTGTTTCTGCCTTTAGTGGTTGCTTTTTTATTTTTATTTTATGGGAGTCTTTAGCTAAAAAGAATGTTGTTATAGGTTATTATGGTAGTTCTTCAACTTTGCTTAATTTGTGTTGATCGCCAGTGCCTTACCACAGTAATTTTTTTGTGCGTGGATTATTTGTTGATTATTCTGTATTGGCTTTTTAG

>Hap_55

ATGGTTTTGTTGTTGCGTCGTAATGTGGTTGATTTGCCTACTAATTATTCTCTTAATTATTATTGGTGTAGCGGGTTTATGATTTCGGCTTTTATGGTAGTTCAGGTAATTACTGGTGTGATTCTTTCACTTTTGTATGTGGCTGATTCAAGATTAAGTTTTCGTTGTGTTATGGATTTGAGAAAAGATTCTTTTTTTACTTGGGGGGTGCGTTATTGACACATCTGGGGGGTTAGTATTTTGTTTGTCCTGTTTTTTGTTCATATGGGTCGTGCCTTATATTATTCAAGTTATACTAAGAAGGGGGTATGGAAAGTGGGGTTTATTTTATATCTTTTAACTATGGCTGAGGCTTTTTTGGGTTATATTTTACCTTGACATCAAATGTCATATTGGGCTGCTACTGTTTTGACGGCTATTGCCGAGAGTATTCCCTTAGTTGGTCCTACGGTGTTTAAGTATTTGGTGGGGGGGTTTTCTGTAACTAAAGTAACTTTGGTTCGTGTATTTTCAGCTCATGTTTGTTTAGGTTTTGTAATTTTAGGTTTAATGATTTTGCATCTTTTTTATTTGCATTCTTCCGGGTCTAATAACCCTTTATTTTCTTCTTTTGGGTATGGAGATGTTGTTTATTTCCACTCTTATTTTACCACTAAGGATTTTTTTTGTTTGGTTGTTTTATGCTGTATTTTGGTTGGATTTATGTGGTTGGTTCCTGATTTGGTGGTAGATACAGAGGGTTATCTAGAGTCTGATCCTTTGGTGACTCCGGTGTCTATAAAGCCTGAGTGATATTTTTTGATTTATTATGCTATGCTTCGTTCTGTTGAGTCTAAGATAGGTGGTTTAGTGTTGGTGGCTAGATTATTATTTTTTATGTGGGTCCCAACTTTTAAAGACTCTAGTTCATATTTTGTTATTCGACAGGTGGTTTTTTGGGGTTTCGTTTGTCTTTTTGTTGGGTTGACTTATTTAGGCTCATGTCACCCTGAGTATCCTTATTTGGGTATTTGTCAGTTATTTTCGGTTGGTGCTGTAGCTTTTATGTTTATTTATAAGCTATTTTGATCGAGTTATACTAAGTTGGGTTTTAGTATTTTTTTAGGGTAAATGGCTAAGTTTAGTTTTTTTAGTTGGCTTTTTACGCTAGATCATAAGCGTGTGGGTATGATTTATACTTTAATTGGGATATGATCAGGTTTTGTTGGGTTGAGTTTTAGGGTAATGATACGTGTTAATTTCGTTGAGCCTTATTTTAATGTAATTTCTTCGGACTGTTATAAATTTTTGATAACTAATCATGGTATTATTATGATTTTTTTTTTTTTGATCCCGGGGTTGATGGGGGGTTTTGGAAATTATTTAATTCCTTTATTACCCGGTTTACCAAATTTAAATTTACCCCGGTTGAAACCCTTGAAAGCAGGGTTGCTTTTTCCTTCTATTTTATTTCTGGGGCTAAATATGGGTTTGGGGGCGGGTAAAGGGGGAACTTTTTACCCCCCTCTCTCCCCTTCCCTTTTTAGGGATAGCCGGGGGGTTAACCTTTTAATGTTTTCTTTACATTTGGCTGGTCTTTCAAGTTTGTTGGGTTCTATAAATTTTATATGTACTCTTTACTCAGCTTTTGTTGATAATTTTGTGTCTCGTAGTTCTATTTTGTTGGGGTCTTATCTATTTACTTCTATTCTTTTATTGTTAACTATTCCTGTGTTGGCTGCTGCCATTACAATGTTGTTGTTTGATCGTAAATTTGGTTCGGCTTTTTTTGATCCTTTGGGTGGTGGTGATCCTGTTTTGTTTCAACATATGTTTTGGTTTTTTGGGCATCCAGAAGTTTATGTATTGATTTTGCCTGGTTTTGGTATGGTAAGTCATGTGTGTAGTAAATTAGGTTGTTCATATGATACTTTTGGTTTTTATGGTTTATTATTTGCTATGTTTTCTATAGTGTGTTTAGGTAGCGTGGTTTGGGGTCACCACATGTTTACTGTGGGGTTGGACGTGAAGACTGCTGTTTTCTTTAGTTCTGTGACTATGATTATTGGGGTTCCCACGGGTATAAAGGTGTTTTCTTGGCTTTATATGATTTTAAATAGTCGTGTTTCGTTGCGTGAGCCTGTGTTTTGATGGGTTTTATCCTTTATTGTGTTGTTTACTATGGGTGGTGTTACTGGTATAATTCTTTCTGCTTGTGTGTTGGATAAAATTTTGCATGACACGTGGTTTGTGGTGGCTCATTTTCATTATGTTATGTCTTTGGGTTCTTATATTAGGGTTATTATATTTTTTGTTTGGTGATGGCCTGTTATCACAGGGGTTAGCTTGAATAAGTATTTGTTACAGTGTCATTGTATAGTATCAAATGTGGGCTTTAATTTGTGTTTTTTTCCTATGCATTATTTTGGTATTTGTGGTTTACCTCGGCGTGTTTGTGTGTATGAGTCAGGGTACGCTTGAGTTAATATGCTTTGTTCAATAGGTTCTTTTGTTTCTGCCTTTAGTGGTTGCTTTTTTATTTTTATTTTATGGGAGTCTTTAGCTAAAAAGAATGTTGTTATAGGTTATTATGGTAGTTCTTCAACTTTGCTTAATTTGTGTTGATCGCCAGTGCCTTACCACAGTAATTTTTTTGTGCGTGGATTATTTGTTGATTATTCTGTATTGGCTTTTTAG

>Hap_56

ATGGTTTTGTTGTTGCGTCGTAATGTGGTTGATTTGCCTACTAATTATTCTCTTAATTATTATTGGTGTAGCGGGTTTATGATTTCGGCTTTTATGGTAGTTCAGGTAATTACTGGTGTGATTCTTTCACTTTTGTATGTGGCTGATTCAAGATTAAGTTTTCGTTGTGTTATGGATTTGAGAAAAGATTCTTTTTTTACTTGGGGGGTGCGTTATTGACACATCTGGGGGGTTAGTATTTTGTTTGTCCTGTTTTTTGTTCATATGGGTCGTGCCTTATATTATTCAAGTTATACTAAGAAGGGGGTATGGAAAGTGGGGTTTATTTTATATCTTTTAACTATGGCTGAGGCTTTTTTGGGTTATATTTTACCTTGACATCAAATGTCATATTGGGCTGCTACTGTTTTGACGGCTATTGCCGAGAGTATTCCCTTAGTTGGTCCTACGGTGTTTAAGTATTTGGTGGGGGGGTTTTCTGTAACTAAAGTAACTTTGGTTCGTGTATTTTCAGCTCATGTTTGTTTAGGTTTTGTAATTTTAGGTTTAATGATTTTGCATCTTTTTTATTTGCATTCTTCCGGGTCTAATAACCCTTTATTTTCTTCTTTTGGGTATGGAGATGTTGTTTATTTCCACTCTTATTTTACCACTAAGGATTTTTTTTGTTTGGTTGTTTTATGCTGTATTTTGGTTGGATTTATGTGGTTGGTTCCTGATTTGGTGGTAGATACAGAGGGTTATCTAGAGTCTGATCCTTTGGTGACTCCCGTGTCTATAAAGCCTGAGTGATATTTTTTGATTTATTATGCTATGCTTCGTTCTGTTGAGTCTAAGATAGGTGGTTTAGTGTTGGTGGCTAGATTATTATTTTTTATGTGGGTCCCAACTTTTAAAGACTCTAGTTCATATTTTGTTATTCGACAGGTGGTTTTTTGGGGTTTCGTTTGTCTTTTTGTTGGGTTGACTTATTTAGGCTCATGTCACCCTGAGTATCCTTATTTGGGTATTTGTCAGTTATTTTCGGTTGGTGCTGTAGCTTTTATGTTTATTTATAAGCTATTTTGATCGAGTTATACTAAGTTGGGTTTTAGTATTTTTTTAGGGTAAATGGCTAAGTTTAGTTTTTTTAGTTGGCTTTTTACGCTAGATCATAAGCGTGTGGGTATGATTTATACTTTAATTGGGATATGATCAGGTTTTGTTGGGTTGAGTTTTAGGGTAATGATACGTGTTAATTTCGTTGAGCCTTATTTTAATGTAATTTCTTCGGACTGTTATAAATTTTTGATAACTAATCATGGTATTATTATGATTTTTTTTTTTTTGAGGCCGGGGTTGATGGGGGGTTTTGGAAATTATTTGATCCCTTTATTACCCGGTTTACCAAATTTAATTTTCCCCCGGTTAAAACCCTTGAAAGCAGGGTGGCTTTTTCCTCCAATTTTATTTCTGGGGCTAAAAATGGGTTTGGGGGCGGGAAAAGGGGGAACTTTTTACCCCCCTCTCTCCTCTTCCCTTTTTAGGGATAGCCGGGGGGTTAACCTTTTAATGTTTTCTTTACTTTTGGCTGGCCTTTCAAGTTTGTTGGGTTCAATAAATTTTATATGTACTCTTTACTCAGCTTTTGTTGATAATTTTGTGTGTCGTAGTTCTATTTTGTTGGGGTCTTATCTATTTACTTCTATTCTTTTATTGTTAACTATTCCTGTGTTGGCTGCTGCCATTACAATGTTGTTGTTTGATCGTAAATTTGGTTCGGCTTTTTTTGATCCTTTGGGTGGTGGTGATCCTGTTTTGTTTCAACATATGTTTTGGTTTTTTGGGCATCCAGAAGTTTATGTATTGATTTTGCCTGGTTTTGGTATGGTAAGTCATGTGTGTAGTAAATTAGGTTGTTCATATGATACTTTTGGTTTTTATGGTTTATTATTTGCTATGTTTTCTATAGTGTGTTTAGGTAGCGTGGTTTGGGGTCACCACATGTTTACTGTGGGGTTGGACGTGAAGACTGCTGTTTTCTTTAGTTCTGTGACTATGATTATTGGGGTTCCCACGGGTATAAAGGTGTTTTCTTGGCTTTATATGATTTTAAATAGTCGTGTTTCGTTGCGTGAGCCTGTGTTTTGATGGGTTTTATCCTTTATTGTGTTGTTTACTATGGGTGGTGTTACTGGTATAATTCTTTCTGCTTGTGTGTTGGATAAAATTTTGCATGACACGTGGTTTGTGGTGGCTCATTTTCATTATGTTATGTCTTTGGGTTCTTATATTAGGGTTATTATATTTTTTGTTTGGTGATGGCCTGTTATCACAGGGGTTAGCTTGAATAAGTATTTGTTACAGTGTCATTGTATAGTATCAAATGTGGGCTTTAATTTGTGTTTTTTTCCTATGCATTATTTTGGTATTTGTGGTTTACCTCGGCGTGTTTGTGTGTATGAGTCAGGGTACGCTTGAGTTAATATGCTTTGTTCAATAGGTTCTTTTGTTTCTGCCTTTAGTGGTTGCTTTTTTATTTTTATTTTATGGGAGTCTTTAGCTAAAAAGAATGTTGTTATAGGTTATTATGGTAGTTCTTCAACTTTGCTTAATTTGTGTTGATCGCCAGTGCCTTACCACAGTAATTTTTTTGTGCGTGGATTATTTGTTGATTATTCTGTATTGGCTTTTTAG

>Hap_57

ATGGTTTTGTTGTTGCGTCGTAATGTGGTTGATTTGCCTACTAATTATTCTCTTAATTATTATTGGTGTAGCGGGTTTATGATTTCGGCTTTTATGGTAGTTCAGGTAATTACTGGTGTGATTCTTTCACTTTTGTATGTGGCTGATTCAAGATTAAGTTTTCGTTGTGTTATGGATTTGAGAAAAGATTCTTTTTTTACTTGGGGGGTGCGTTATTGACACATCTGGGGGGTTAGTATTTTGTTTGTCCTGTTTTTTGTTCATATGGGTCGTGCCTTATATTATTCAAGTTATACTAAGAAGGGGGTATGGAAAGTGGGGTTTATTTTATATCTTTTAACTATGGCTGAGGCTTTTTTGGGTTATATTTTACCTTGACATCAAATGTCATATTGGGCTGCTACTGTTTTGACGGCTATTGCCGAGAGTATTCCCTTAGTTGGTCCTACGGTGTTTAAGTATTTGGTGGGGGGGTTTTCTGTAACTAAAGTAACTTTGGTTCGTGTATTTTCAGCTCATGTTTGTTTAGGTTTTGTAATTTTAGGTTTAATGATTTTGCATCTTTTTTATTTGCATTCTTCCGGGTCTAATAACCCTTTATTTTCTTCTTTTGGGTATGGAGATGTTGTTTATTTCCACTCTTATTTTACCACTAAGGATTTTTTTTGTTTGGTTGTTTTATGCTGTATTTTGGTTGGATTTATGTGGTTGGTTCCTGATTTGGTGGTAGATACAGAGGGTTATCTAGAGTCTGATCCTTTGGTGACTCCGGTGTCTATAAAGCCTGAGTGATATTTTTTGATTTATTATGCTATGCTTCGTTCTGTTGAGTCTAAGATAGGTGGTTTAGTGTTGGTGGCTAGATTATTATTTTTTATGTGGGTCCCAACTTTTAAAGACTCTAGTTCATATTTTGTTATTCGACAGGTGGTTTTTTGGGGTTTCGTTTGTCTTTTTGGTGGGTTGACTTATTTAGGCTCATGTCACCCTGAGTATCCTTATTTGGGTATTTGTCAGTTATTTTCGGTTGGTGCTGTAGCTTTTATGTTTATTTATAAGCTATTTTGATCGAGTTATACTAAGTTGGGTTTTAGTATTTTTTTAGGGTAAATGGCTAAGTTTAGTTTTTTTAGTTGGCTTTTTACGCTAGATCATAAGCGTGTGGGTATGATTTATACTTTAATTGGGATATGATCAGGTTTTGTTGGGTTGAGTTTTAGGGTAATGATACGTGTTAATTTCGTTGAGCCTTATTTTAATGTAATTTCTTCGGACTGTTATAAATTTTTGATAACTAATCATGGTATTATTATGATTTTTTTTTTTTTGATCCCGGGGTTGATGGGGGGTTTTGGAAATTATTTAATCCCTTTTTTACCCGGTTTACCAAATTTAAATTTCCCCCGGTTGAAACCCTTGAAAGCAGGGTGGCTTTTTCCTTCTATTTTATTTCTGGGGCTAAAAATGGGTTGGGGGGCGGGAAAAGGGGGAACTTTTTACCCCCCTCTCTCCCCTTCCCTTTTTAGGGATAGCCGGGGGGTTAACCTTTTGAGGTTTTCTTTACATTTGGCTGGTCTTTCAAGTTTGTTGGGTTCTATAAATTTTATATGTACTCTTTACTCAGCTTTTGTTGATAATTTTGTGTCTCGTAGTTCTATTTTGTTGGGGTCTTATCTATTTACTTCTATTCTTTTATTGTTAACTATTCCTGTGTTGGCTGCTGCCATTACAATGTTGTTGTTTGATCGTAAATTTGGTTCGGCTTTTTTTGATCCTTTGGGTGGTGGTGATCCTGTTTTGTTTCAACATATGTTTTGGTTTTTTGGGCATCCAGAAGTTTATGTATTGATTTTGCCTGGTTTTGGTATGGTAAGTCATGTGTGTAGTAAATTAGGTTGTTCATATGATACTTTTGGTTTTTATGGTTTATTATTTGCTATGTTTTCTATAGTGTGTTTAGGTAGCGTGGTTTGGGGTCACCACATGTTTACTGTGGGGTTGGACGTGAAGACTGCTGTTTTCTTTAGTTCTGTGACTATGATTATTGGGGTTCCCACGGGTATAAAGGTGTTTTCTTGGCTTTATATGATTTTAAATAGTCGTGTTTCGTTGCGTGAGCCTGTGTTTTGATGGGTTTTATCCTTTATTGTGTTGTTTACTATGGGTGGTGTTACTGGTATAATTCTTTCTGCTTGTGTGTTGGATAAAATTTTGCATGACACGTGGTTTGTGGTGGCTCATTTTCATTATGTTATGTCTTTGGGTTCTTATATTAGGGTTATTATATTTTTTGTTTGGTGATGGCCTGTTATCACAGGGGTTAGCTTGAATAAGTATTTGTTACAGTGTCATTGTATAGTATCAAATGTGGGCTTTAATTTGTGTTTTTTTCCTATGCATTATTTTGGTATTTGTGGTTTACCTCGGCGTGTTTGTGTGTATGAGTCAGGGTACGCTTGAGTTAATATGCTTTGTTCAATAGGTTCTTTTGTTTCTGCCTTTAGTGGTTGCTTTTTTATTTTTATTTTATGGGAGTCTTTAGCTAAAAAGAATGTTGTTATAGGTTATTATGGTAGTTCTTCAACTTTGCTTAATTTGTGTTGATCGCCAGTGCCTTACCACAGTAATTTTTTTGTGCGTGGATTATTTGTTGATTATTCTGTATTGGCTTTTTAG

>Hap_58

ATGGTTTTGTTGTTGCGTCGTAATGTGGTTGATTTGCCTACTAATTATTCTCTTAATTATTATTGGTGTAGCGGGTTTATGATTTCGGCTTTTATGGTAGTTCAGGTAATTACTGGTGTGATTCTTTCACTTTTGTATGTGGCTGATTCAAGATTAAGTTTTCGTTGTGTTATGGATTTGAGAAAAGATTCTTTTTTTACTTGGGGGGTGCGTTATTGACACATCTGGGGGGTTAGTATTTTGTTTGTCCTGTTTTTTGTTCATATGGGTCGTGCCTTATATTATTCAAGTTATACTAAGAAGGGGGTATGGAAAGTGGGGTTTATTTTATATCTTTTAACTATGGCTGAGGCTTTTTTGGGTTATATTTTACCTTGACATCAAATGTCATATTGGGCTGCTACTGTTTTGACGGCTATTGCCGAGAGTATTCCCTTAGTTGGTCCTACGGTGTTTAAGTATTTGGTGGGGGGGTTTTCTGTAACTAAAGTAACTTTGGTTCGTGTATTTTCAGCTCATGTTTGTTTAGGTTTTGTAATTTTAGGTTTAATGATTTTGCATCTTTTTTATTTGCATTCTTCCGGGTCTAATAACCCTTTATTTTCTTCTTTTGGGTATGGAGATGTTGTTTATTTCCACTCTTATTTTACCACTAAGGATTTTTTTTGTTTGGTTGTTTTATGCTGTATTTTGGTTGGATTTATGTGGTTGGTTCCTGATTTGGTGGTAGATACAGAGGGTTATCTAGAGTCTGATCCTTTGGTGACTCCGGTGTCTATAAAGCCTGAGTGATATTTTTTGATTTATTATGCTATGCTTCGTTCTGTTGAGTCTAAGATAGGTGGTTTAGTGCTGGTGGCTAGATTATTATTTTTTATGTGGGTCCCAACTTTTAAAGACTCTAGTTCATATTTTGTTATTCGACAGGTGGTTTTTTGGGGTTTCGTTTGTCTTTTTGTTGGGTTGACTTATTTAGGCTCATGTCACCCTGAGTATCCTTATTTGGGTATTTGTCAGTTATTTTCGGTTGGTGCTGTAGCTTTTATGTTTATTTATAAGCTATTTTGATCGAGTTATACTAAGTTGGGTTTTAGTATTTTTTTAGGGTAAATGGCTAAGTTTAGTTTTTTTAGTTGGCTTTTTACGCTAGATCATAAGCGTGTGGGTATGATTTATACTTTAATTGGGATATGATCAGGTTTTGTTGGGTTGAGTTTTAGGGTAATGATACGTGTTAATTTCGTTGAGCCTTATTTTAATGTAATTTCTTCGGACTGTTATAAATTTTTGATAACTAATCATGGTATTATTATGATTTTTTTTTTTTTGATGCCGGGGTTGATGGGGGGTTTGGGAAATTATTTGATTCCTTTATTACCCGGTTTACCAAATTTAAATTTACCCCGGTTGAAACCCTTGAAAGCAGGGTGGCTTTTTCCTCCTATTTTATTCCTGGGGCTAATAATGTGTTGGGGTGCGGGAATAGGGGGAACTTTTTACCCCCCTCTCTCCTCTTCCCTTTTTAGGGATAGCCGGGGTGTTAATCTTTTGATGTTTTCTTTACATTTGGCTGGTCTTTCAAGTTTGTTGGGTTCAATAAATTTTATATGTACTCTTTACTCAGCTTTTGTTGATAATTTTGTGTCTCGTAGTTCTATTTTGTTGGGGTCTTATCTATTTACTTCTATTCTTTTATTGTTAACTATTCCTGTGTTGGCTGCTGCCATTACAATGTTGTTGTTTGATCGTAAATTTGGTTCGGCTTTTTTTGATCCTTTGGGTGGTGGTGATCCTGTTTTGTTTCAACATATGTTTTGGTTTTTTGGGCATCCAGAAGTTTATGTATTGATTTTGCCTGGTTTTGGTATGGTAAGTCATGTGTGTAGTAAATTAGGTTGTTCATATGATACTTTTGGTTTTTATGGTTTATTATTTGCTATGTTTTCTATAGTGTGTTTAGGTAGCGTGGTTTGGGGTCACCACATGTTTACTGTGGGGTTGGACGTGAAGACTGCTGTTTTCTTTAGTTCTGTGACTATGATTATTGGGGTTCCCACGGGTATAAAGGTGTTTTCTTGGCTTTATATGATTTTAAATAGTCGTGTTTCGTTGCGTGAGCCTGTGTTTTGATGGGTTTTATCCTTTATTGTGTTGTTTACTATGGGTGGTGTTACTGGTATAATTCTTTCTGCTTGTGTGTTGGATAAAATTTTGCATGACACGTGGTTTGTGGTGGCTCATTTTCATTATGTTATGTCTTTGGGTTCTTATATTAGGGTTATTATATTTTTTGTTTGGTGATGGCCTGTTATCACAGGGGTTAGCTTGAATAAGTATTTGTTACAGTGTCATTGTATAGTATCAAATGTGGGCTTTAATTTGTGTTTTTTTCCTATGCATTATTTTGGTATTTGTGGTTTACCTCGGCGTGTTTGTGTGTATGAGTCAGGGTACGCTTGAGTTAATATGCTTTGTTCAATAGGTTCTTTTGTTTCTGCCTTTAGTGGTTGCTTTTTTATTTTTATTTTATGGGAGTCTTTAGCTAAAAAGAATGTTGTTATAGGTTATTATGGTAGTTCTTCAACTTTGCTTAATTTGTGTTGATCGCCAGTGCCTTACCACAGTAATTTTTTTGTGCGTGGATTATTTGTTGATTATTCTGTATTGGCTTTTTAG

>Hap_59

ATGGTTTTGTTGTTGCGTCGTAATGTGGTTGATTTGCCTACTAATTATTCTCTTAATTATTATTGGTGTAGCGGGTTTATGATTTCGGCTTTTATGGTAGTTCAGGTAATTACTGGTGTGATTCTTTCACTTTTGTATGTGGCTGATTCAAGATTAAGTTTTCGTTGTGTTATGGATTTGAGAAAAGATTCTTTTTTTACTTGGGGGGTGCGTTATTGACACATCTGGGGGGTTAGTATTTTGTTTGTCCTGTTTTTTGTTCATATGGGTCGCGCCTTATATTATTCAAGTTATACTAAGAAGGGGGTATGGAAAGTGGGGTTTATTTTATATCTTTTAACTATGGCTGAGGCTTTTTTGGGTTATATTTTACCTTGACATCAAATGTCATATTGGGCTGCTACTGTTTTGACGGCTATTGCCGAGAGTATTCCCTTAGTTGGTCCTACGGTGTTTAAGTATTTGGTGGGGGGGTTTTCTGTAACTAAAGTAACTTTGGTTCGTGTATTTTCAGCTCATGTTTGTTTAGGTTTTGTAATTTTAGGTTTAATGATTTTGCATCTTTTTTATTTGCATTCTTCCGGGTCTAATAACCCTTTATTTTCTTCTTTTGGGTATGGAGATGTTGTTTATTTCCACTCTTATTTTACCACTAAGGATTTTTTTTGTTTGGTTGTTTTATGCTGTATTTTGGTTGGATTTATGTGGTTGGTTCCTGATTTGGTGGTAGATACAGAGGGTTATCTAGAGTCTGATCCTTTGGTGACTCCGGTGTCTATAAAGCCTGAGTGATATTTTTTGATTTATTATGCTATGCTTCGTTCTGTTGAGTCTAAGATAGGTGGTTTAGTGTTGGTGGCTAGATTATTATTTTTTATGTGGGTCCCAACTTTTAAAGACTCTAGTTCATATTTTGTTATTCGACAGGTGGTTTTTTGGGGTTTCGTTTGTCTTTTTGTTGGGTTGACTTATTTAGGCTCATGTCACCCTGAGTATCCTTATTTGGGTATTTGTCAGTTATTTTCGGTTGGTGCTGTAGCTTTTATGTTTATTTATAAGCTATTTTGATCGAGTTATACTAAGTTGGGTTTTAGTATTTTTTTAGGGTAAATGGCTAAGTTTAGTTTTTTTAGTTGGCTTTTTACGCTAGATCATAAGCGTGTGGGTATGATTTATACTTTAATTGGGATATGATCAGGTTTTGTTGGGTTGAGTTTTAGGGTAATGATACGTGTTAATTTCGTTGAGCCTTATTTTAATGTAATTTCTTCGGACTGTTATAAATTTTTGATAACTAATCATGGTATTATTATGATTTTTTTTTTTTTGATGCCGGGGTTGATGGGGGGTTTGGGAAATTATTTGATTCCTTTATTACCCGGTTTACCAAATTTAAATTTACCCCGGTTGAAACCCTTGAAAGCAGGGTGGCTTTTTCCTCCTATTTTATTCCTGGGGCTAATAATGTGTTGGGGTGCGGGAATAGGGGGAACTTTTTACCCCCCTCTCTCCTCTTCCCTTTTTAGGGATAGCCGGGGTGTTAATCTTTTGATGTTTTCTTTACATTTGGCTGGTCTTTCAAGTTTGTTGGGTTCAATAAATTTTATATGTACTCTTTACTCAGCTTTTGTTGATAATTTTGTGTCTCGTAGTTCTATTTTGTTGGGGTCTTATCTATTTACTTCTATTCTTTTATTGTTAACTATTCCTGTGTTGGCTGCTGCCATTACAATGTTGTTGTTTGATCGTAAATTTGGTTCGGCTTTTTTTGATCCTTTGGGTGGTGGTGATCCTGTTTTGTTTCAACATATGTTTTGGTTTTTTGGGCATCCAGAAGTTTATGTATTGATTTTGCCTGGTTTTGGTATGGTAAGTCATGTGTGTAGTAAATTAGGTTGTTCATATGATACTTTTGGTTTTTATGGTTTATTATTTGCTATGTTTTCTATAGTGTGTTTAGGTAGCGTGGTTTGGGGTCACCACATGTTTACTGTGGGGTTGGACGTGAAGACTGCTGTTTTCTTTAGTTCTGTGACTATGATTATTGGGGTTCCCACGGGTATAAAGGTGTTTTCTTGGCTTTATATGATTTTAAATAGTCGTGTTTCGTTGCGTGAGCCTGTGTTTTGATGGGTTTTATCCTTTATTGTGTTGTTTACTATGGGTGGTGTTACTGGTATAATTCTTTCTGCTTGTGTGTTGGATAAAATTTTGCATGACACGTGGTTTGTGGTGGCTCATTTTCATTATGTTATGTCTTTGGGTTCTTATATTAGGGTTATTATATTTTTTGTTTGGTGATGGCCTGTTATCACAGGGGTTAGCTTGAATAAGTATTTGTTACAGTGTCATTGTATAGTATCAAATGTGGGCTTTAATTTGTGTTTTTTTCCTATGCATTATTTTGGTATTTGTGGTTTACCTCGGCGTGTTTGTGTGTATGAGTCAGGGTACGCTTGAGTTAATATGCTTTGTTCAATAGGTTCTTTTGTTTCTGCCTTTAGTGGTTGCTTTTTTATTTTTATTTTATGGGAGTCTTTAGCTAAAAAGAATGTTGTTATAGGTTATTATGGTAGTTCTTCAACTTTGCTTAATTTGTGTTGATCGCCAGTGCCTTACCACAGTAATTTTTTTGTGCGTGGATTATTTGTTGATTATTCTGTATTGGCTTTTTAG

>Hap_60

ATGGTTTTGTTGTTGCGTCGTAATGTGGTTGATTTGCCTACTAATTATTCTCTTAATTATTATTGGTGTAGCGGGTTTATGATTTCGGCTTTTATGGTAGTTCAGGTAATTACTGGTGTGATTCTTTCACTTTTGTATGTGGCTGATTCAAGATTAAGTTTTCGTTGTGTTATGGATTTGAGAAAAGATTCTTTTTTTACTTGGGGGGTGCGTTATTGACACATCTGGGGGGTTAGTATTTTGTTTGTCCTGTTTTTTGTTCATATGGGTCGCGCCTTATATTATTCAAGTTATACTAAGAAGGGGGTATGGAAAGTGGGGTTTATTTTATATCTTTTAACTATGGCTGAGGCTTTTTTGGGTTATATTTTACCTTGACATCAAATGTCATATTGGGCTGCTACTGTTTTGACGGCTATTGCCGAGAGTATTCCCTTAGTCGGTCCTACGGTGTTTAAGTATTTGGTGGGGGGGTTTTCTGTAACTAAAGTAACTTTGGTTCGTGTATTTTCAGCTCATGTTTGTTTAGGTTTTGTAATTTTAGGTTTAATGATTTTGCATCTTTTTTATTTGCATTCTTCCGGGTCTAATAACCCTTTATTTTCTTCTTTTGGGTATGGAGATGTTGTTTATTTCCACTCTTATTTTACCACTAAGGATTTTTTTTGTTTGGTTGTTTTATGCTGTATTTTGGTTGGATTTATGTGGTTGGTTCCTGATTTGGTGGTAGATACAGAGGGTTATCTAGAGTCTGATCCTTTGGTGACTCCGGTGTCTATAAAGCCTGAGTGATATTTTTTGATTTATTATGCTATGCTTCGTTCTGTTGAGTCTAAGATAGGTGGTTTAGTGTTGGTGGCTAGATTATTATTTTTTATGTGGGTCCCAACTTTTAAAGACTCTAGTTCATATTTTGTTATTCGACAGGTGGTTTTTTGGGGTTTCGTTTGTCTTTTTGTTGGGTTGACTTATTTAGGCTCATGTCACCCTGAGTATCCTTATTTGGGTATTTGTCAGTTATTTTCGGTTGGTGCTGTAGCTTTTATGTTTATTTATAAGCTATTTTGATCGAGTTATACTAAGTTGGGTTTTAGTATTTTTTTAGGGTAAATGGCTAAGTTTAGTTTTTTTAGTTGGCTTTTTACGCTAGATCATAAGCGTGTGGGTATGATTTATACTTTAATTGGGATATGATCAGGTTTTGTTGGGTTGAGTTTTAGGGTAATGATACGTGTTAATTTCGTTGAGCCTTATTTTAATGTAATTTCTTCGGACTGTTATAAATTTTTGATAACTAATCATGGTATTATTATGATTTTTTTTTTTTTGATGCCGGGGTTGATGGGGGGTTTTGGAAATTATTTGATTCCTTTATTACCCGGTTTACCAAATTTAAATTTCCCCCGGTTGAAACCCTTGAAAGCAGGGTGGCTTTTTCCTTCTATTTTATTTCTGGGGCTAATAATGTGTTTGGGTGCGGGAAAAGGGGGAACTTTTTACCCCCCTCTCTCCCCTTCCCTTTTTAGGGATAGCCGGGGGGTTAATCTTTTGATGTTTTCTTTACATTTGGCTGGTCTTTCAAGTTTGTTGGGTTCAATAAATTTTATATGTACTCTTTACTCAGCTTTTGTTGATAATTTTGTGTCTCGTAGTTCTATTTTGTTGGGGTCTTATCTATTTACTTCTATTCTTTTATTGTTAACTATTCCTGTGTTGGCTGCTGCCATTACAATGTTGTTGTTTGATCGTAAATTTGGTTCGGCTTTTTTTGATCCTTTGGGTGGTGGTGATCCTGTTTTGTTTCAACATATGTTTTGGTTTTTTGGGCATCCAGAAGTTTATGTATTGATTTTGCCTGGTTTTGGTATGGTAAGTCATGTGTGTAGTAAATTAGGTTGTTCATATGATACTTTTGGTTTTTATGGTTTATTATTTGCTATGTTTTCTATAGTGTGTTTAGGTAGCGTGGTTTGGGGTCACCACATGTTTACTGTGGGGTTGGACGTGAAGACTGCTGTTTTCTTTAGTTCTGTGACTATGATTATTGGGGTTCCCACGGGTATAAAGGTGTTTTCTTGGCTTTATATGATTTTAAATAGTCGTGTTTCGTTGCGTGAGCCTGTGTTTTGATGGGTTTTATCCTTTATTGTGTTGTTTACTATGGGTGGTGTTACTGGTATAATTCTTTCTGCTTGTGTGTTGGATAAAATTTTGCATGACACGTGGTTTGTGGTGGCTCATTTTCATTATGTTATGTCTTTGGGTTCTTATATTAGGGTTATTATATTTTTTGTTTGGTGATGGCCTGTTATCACAGGGGTTAGCTTGAATAAGTATTTGTTACAGTGTCATTGTATAGTATCAAATGTGGGCTTTAATTTGTGTTTTTTTCCTATGCATTATTTTGGTATTTGTGGTTTACCTCGGCGTGTTTGTGTGTATGAGTCAGGGTACGCTTGAGTTAATATGCTTTGTTCAATAGGTTCTTTTGTTTCTGCCTTTAGTGGTTGCTTTTTTATTTTTATTTTATGGGAGTCTTTAGCTAAAAAGAATGTTGTTATAGGTTATTATGGTAGTTCTTCAACTTTGCTTAATTTGTGTTGATCGCCAGTGCCTTACCACAGTAATTTTTTTGTGCGTGGATTATTTGTTGATTATTCTGTATTGGCTTTTTAG

>Hap_61

ATGGTTTTGTTGTTGCGTCGTAATGTGGTTGATTTGCCTACTAATTATTCTCTTAATTATTATTGGTGTAGCGGGTTTATGATTTCGGCTTTTATGGTAGTTCAGGTAATTACTGGTGTGATTCTTTCACTTTTGTATGTGGCTGATTCAAGATTAAGTTTTCGTTGTGTTATGGATTTGAGAAAAGATTCTTTTTTTACTTGGGGGGTGCGTTATTGACACATCTGGGGGGTTAGTATTTTGTTTGTCCTGTTTTTTGTTCATATGGGTCGCGCCTTATATTATTCAAGTTATACTAAGAAGGGGGTATGGAAAGTGGGGTTTATTTTATATCTTTTAACTATGGCTGAGGCTTTTTTGGGTTATATTTTACCTTGACATCAAATGTCATATTGGGCTGCTACTGTTTTGACGGCTATTGCCGAGAGTATTCCCTTAGTCGGTCCTACGGTGTTTAAGTATTTGGTGGGGGGGTTTTCTGTAACTAAAGTAACTTTGGTTCGTGTATTTTCAGCTCATGTTTGTTTAGGTTTTGTAATTTTAGGTTTAATGATTTTGCATCTTTTTTATTTGCATTCTTCCGGGTCTAATAACCCTTTATTTTCTTCTTTTGGGTATGGAGATGTTGTTTATTTCCACTCTTATTTTACCACTAAGGATTTTTTTTGTTTGGTTGTTTTATGCTGTATTTTGGTTGGATTTATGTGGTTGGTTCCTGATTTGGTGGTAGATACAGAGGGTTATCTAGAGTCTGATCCTTTGGTGACTCCGGTGTCTATAAAGCCTGAGTGATATTTTTTGATTTATTATGCTATGCTTCGTTCTGTTGAGTCTAAGATAGGTGGTTTAGTGTTGGTGGCTAGATTATTATTTTTTATGTGGGTCCCAACTTTTAAAGACTCTAGTTCATATTTTGTTATTCGACAGGTGGTTTTTTGGGGTTTCGTTTGTCTTTTTGTTGGGTTGACTTATTTAGGCTCATGTCACCCTGAGTATCCTTATTTGGGTATTTGTCAGTTATTTTCGGTTGGTGCTGTAGCTTTTATGTTTATTTATAAGCTATTTTGATCGAGTTATACTAAGTTGGGTTTTAGTATTTTTTTAGGGTAAATGGCTAAGTTTAGTTTTTTTAGTTGGCTTTTTACGCTAGATCATAAGCGTGTGGGTATGATTTATACTTTAATTGGGATATGATCAGGTTTTGTTGGGTTGAGTTTTAGGGTAATGATACGTGTTAATTTCGTTGAGCCTTATTTTAATGTAATTTCTTCGGACTGTTATAAATTTTTGATAACTAATCATGGTATTATTATGATTTTTTTTTTTTTGATCCCGGGGTTGATGGGGGGTTTTGGAAATTATTTGATCCCTTTATTACCCGGTTTACCAAATTTAAATTTCCCCCGGTTAAAACCCTTGAAAGCAGGGTGGCTTTTTCCTCCTATTTTATTTCTGGGGCTAATAATGGGTTGGGGGGCGGGAAAAGGGGGAACTTTTTACCCCCCTCTCTCCCCTTCCCTTTTTAGGGATAGCCGGGGGGTTAATCTTTTAAGGTTTTCTTTACATTTGGCTGGTCTTTCAAGTTTGTTGGGTTCAATAAATTTTATATGTACTCTTTACTCAGCTTTTGTTGATAATTTTGTGTCTCGTAGTTCTATTTTGTTGGGGTCTTATCTATTTACTTCTATTCTTTTATTGTTAACTATTCCTGTGTTGGCTGCTGCCATTACAATGTTGTTGTTTGATCGTAAATTTGGTTCGGCTTTTTTTGATCCTTTGGGTGGTGGTGATCCTGTTTTGTTTCAACATATGTTTTGGTTTTTTGGGCATCCAGAAGTTTATGTATTGATTTTGCCTGGTTTTGGTATGGTAAGTCATGTGTGTAGTAAATTAGGTTGTTCATATGATACTTTTGGTTTTTATGGTTTATTATTTGCTATGTTTTCTATAGTGTGTTTAGGTAGCGTGGTTTGGGGTCACCACATGTTTACTGTGGGGTTGGACGTGAAGACTGCTGTTTTCTTTAGTTCTGTGACTATGATTATTGGGGTTCCCACGGGTATAAAGGTGTTTTCTTGGCTTTATATGATTTTAAATAGTCGTGTTTCGTTGCGTGAGCCTGTGTTTTGATGGGTTTTATCCTTTATTGTGTTGTTTACTATGGGTGGTGTTACTGGTATAATTCTTTCTGCTTGTGTGTTGGATAAAATTTTGCATGACACGTGGTTTGTGGTGGCTCATTTTCATTATGTTATGTCTTTGGGTTCTTATATTAGGGTTATTATATTTTTTGTTTGGTGATGGCCTGTTATCACAGGGGTTAGCTTGAATAAGTATTTGTTACAGTGTCATTGTATAGTATCAAATGTGGGCTTTAATTTGTGTTTTTTTCCTATGCATTATTTTGGTATTTGTGGTTTACCTCGGCGTGTTTGTGTGTATGAGTCAGGGTACGCTTGAGTTAATATGCTTTGTTCAATAGGTTCTTTTGTTTCTGCCTTTAGTGGTTGCTTTTTTATTTTTATTTTATGGGAGTCTTTAGCTAAAAAGAATGTTGTTATAGGTTATTATGGTAGTTCTTCAACTTTGCTTAATTTGTGTTGATCGCCAGTGCCTTACCACAGTAATTTTTTTGTGCGTGGATTATTTGTTGATTATTCTGTATTGGCTTTTTAG
