# Supplementary figures and images for: Genetic Structure Analysis of Spirometra erinaceieuropaei Isolates from Central and Southern China
Source: PLoS One. 2015 Mar 20;10(3):e0119295. doi: 10.1371/journal.pone.0119295 (PMC4368571; doi:10.1371/journal.pone.0119295)

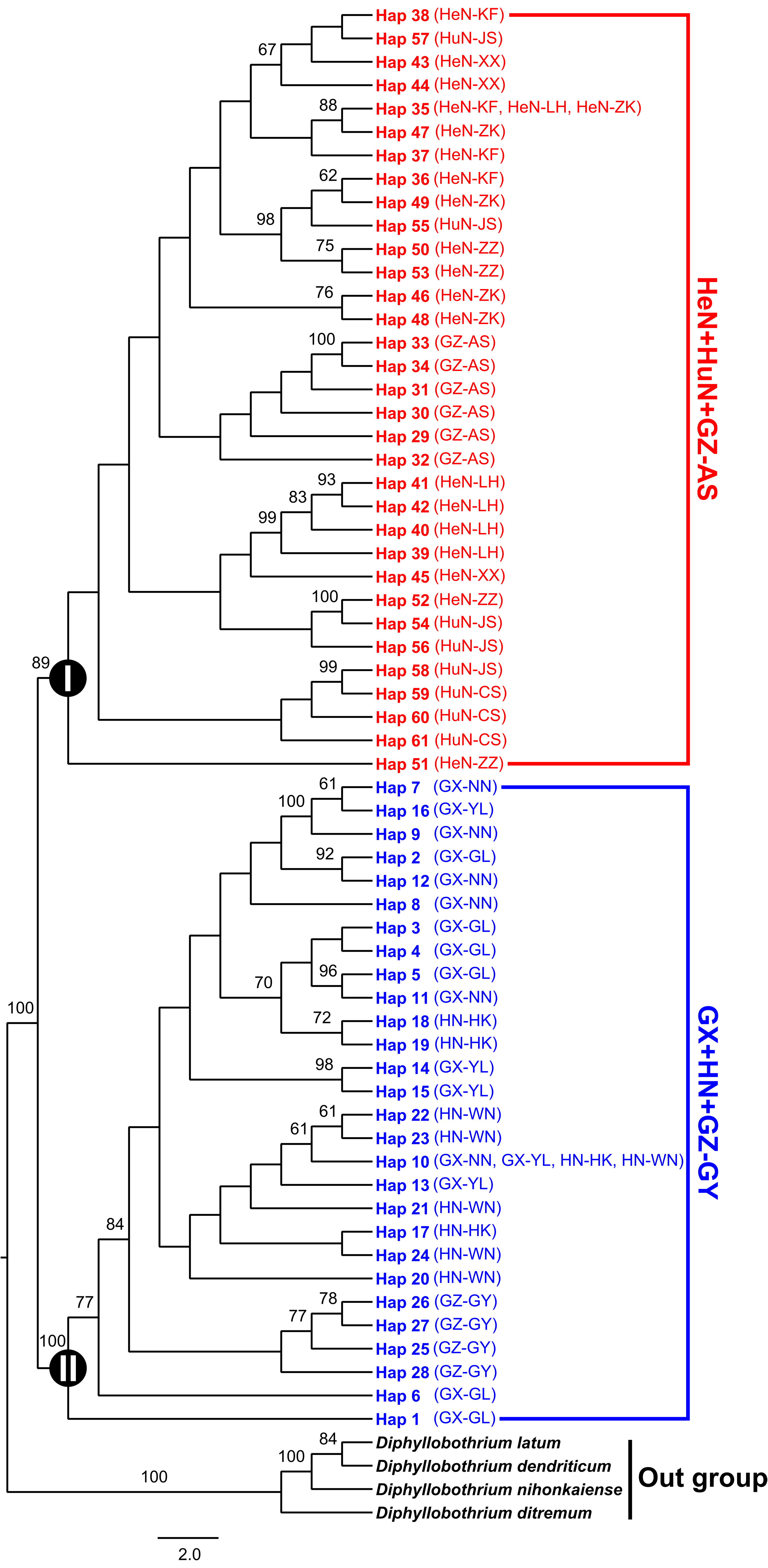

Supplement: S1 Fig — Numbers above branches represent the bootstrap values. Only bootstrap values above 60 are shown. Circled Roman numbers ‘I’ and ‘II’ refer to two main clades discussed in the text. (TIF) [file pone.0119295.s002.tif]

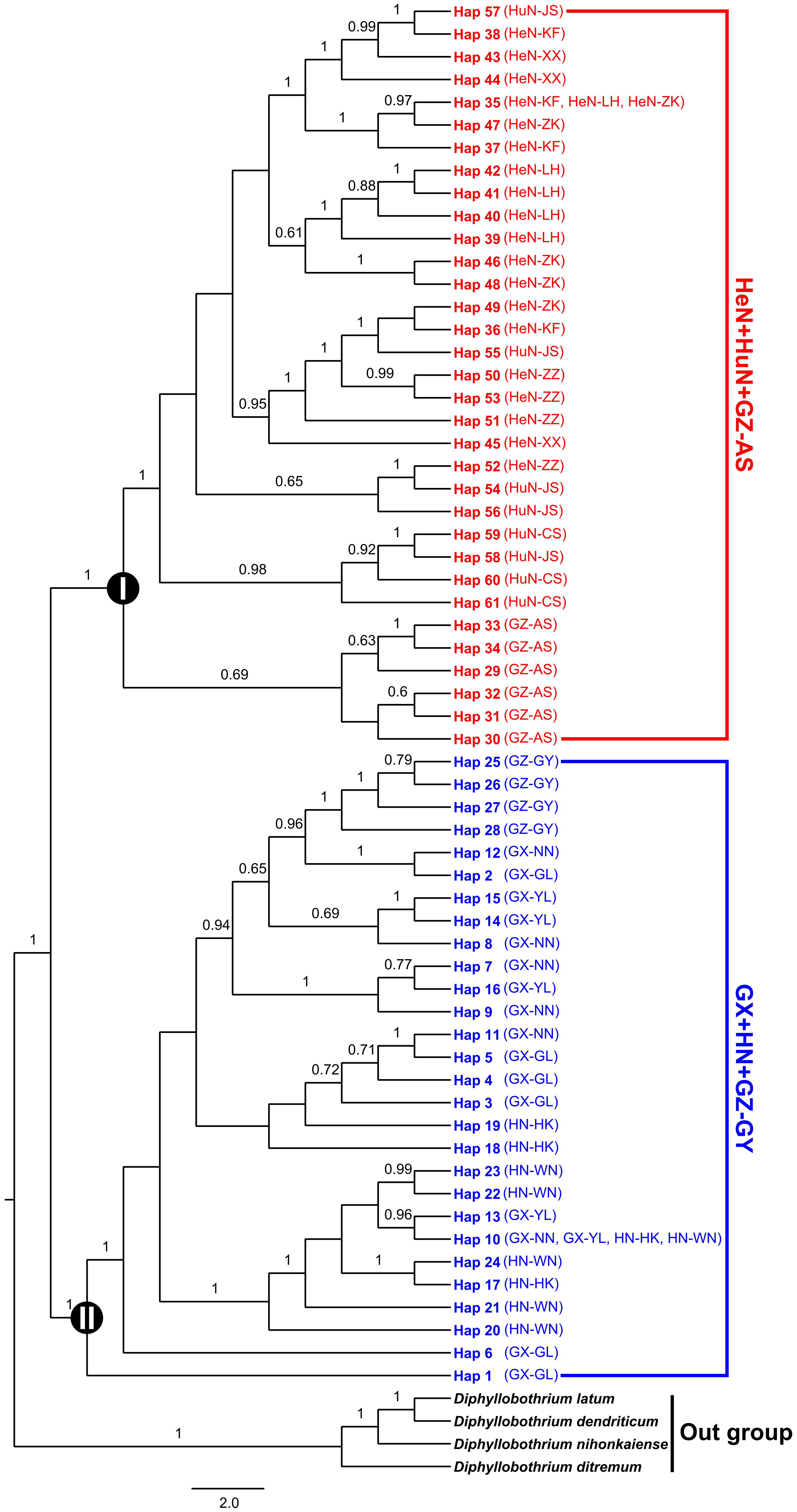

Supplement: S2 Fig — Numbers above branches represent the Bayesian posterior probabilities. Only posterior probabilities above 0.6 are shown. Circled Roman numbers ‘I’ and ‘II’ refer to two main clades discussed in the text. (TIF) [file pone.0119295.s003.tif]
