# Supplementary material for: Genetic Structure Analysis of Spirometra erinaceieuropaei Isolates from Central and Southern China
Source: PLoS One. 2015 Mar 20;10(3):e0119295. doi: 10.1371/journal.pone.0119295 (PMC4368571; doi:10.1371/journal.pone.0119295)
Supplement: S1 Table — (DOC) [file pone.0119295.s004.doc]

**Table S1.** *Spirometra erinaceieuropaei* sampling and data summary for this study.

| Sample codes | Host | Longitude | Latitude | Year | Accession number | |
| --- | --- | --- | --- | --- | --- | --- |
|  |  |  |  |  | *cytb* | *cox1* |
| GX-GL-1 | *R. rugulosa* | 110.2 E | 25.23 N | 2013 | KM605343 | KM605255 |
| GX-GL-2 | *R. rugulosa* | 110.2 E | 25.23 N | 2013 | KM605344 | KM605256 |
| GX-GL-3 | *R. rugulosa* | 110.2 E | 25.23 N | 2013 | KM605345 | KM605257 |
| GX-GL-4 | *R. rugulosa* | 110.2 E | 25.23 N | 2013 | KM605346 | KM605258 |
| GX-GL-5 | *R. rugulosa* | 110.2 E | 25.23 N | 2013 | KM605347 | KM605259 |
| GX-GL-6 | *R. rugulosa* | 110.2 E | 25.23 N | 2013 | KM605348 | KM605260 |
| GX-GL-7 | *R. nigromaculata* | 110.2 E | 25.23 N | 2013 | KM605349 | KM605261 |
| GX-GL-8 | *R. nigromaculata* | 110.2 E | 25.23 N | 2013 | KM605350 | KM605262 |
| GX-NN-1 | *E. chinensis* | 108.48 E | 22.75 N | 2013 | KM605351 | KM605263 |
| GX-NN-2 | *E. chinensis* | 108.48 E | 22.75 N | 2013 | KM605352 | KM605264 |
| GX-NN-3 | *R. rugulosa* | 108.48 E | 22.75 N | 2013 | KM605353 | KM605265 |
| GX-NN-4 | *R. rugulosa* | 108.48 E | 22.75 N | 2013 | KM605354 | KM605266 |
| GX-NN-5 | *R. rugulosa* | 108.48 E | 22.75 N | 2013 | KM605355 | KM605267 |
| GX-NN-6 | *R. rugulosa* | 108.48 E | 22.75 N | 2013 | KM605356 | KM605268 |
| GX-NN-7 | *R. nigromaculata* | 108.48 E | 22.75 N | 2013 | KM605357 | KM605269 |
| GX-NN-8 | *R. nigromaculata* | 108.48 E | 22.75 N | 2013 | KM605358 | KM605270 |
| GX-YL-1 | *R. rugulosa* | 110.27 E | 22.33 N | 2013 | KM605359 | KM605271 |
| GX-YL-2 | *R. rugulosa* | 110.27 E | 22.33 N | 2013 | KM605360 | KM605272 |
| GX-YL-3 | *R. rugulosa* | 110.27 E | 22.33 N | 2013 | KM605361 | KM605273 |
| GX-YL-4 | *R. rugulosa* | 110.27 E | 22.33 N | 2013 | KM605362 | KM605274 |
| GX-YL-5 | *R. nigromaculata* | 110.27 E | 22.33 N | 2013 | KM605363 | KM605275 |
| GX-YL-6 | *R. nigromaculata* | 110.27 E | 22.33 N | 2013 | KM605364 | KM605276 |
| HN-HK-1 | *R. rugulosa* | 110.32 E | 20.03 N | 2013 | KM605365 | KM605277 |
| HN-HK-2 | *R. rugulosa* | 110.32 E | 20.03 N | 2013 | KM605366 | KM605278 |
| HN-HK-3 | *R. nigromaculata* | 110.32 E | 20.03 N | 2013 | KM605367 | KM605279 |
| HN-HK-4 | *R. nigromaculata* | 110.32 E | 20.03 N | 2013 | KM605368 | KM605280 |
| HN-HK-5 | *R. nigromaculata* | 110.32 E | 20.03 N | 2013 | KM605369 | KM605281 |
| HN-WZS-1 | *R. rugulosa* | 110.4 E | 18.8 N | 2013 | KM605370 | KM605282 |
| HN-WZS-2 | *R. rugulosa* | 110.4 E | 18.8 N | 2013 | KM605371 | KM605283 |
| HN-WZS-3 | *R. rugulosa* | 110.4 E | 18.8 N | 2013 | KM605372 | KM605284 |
| HN-WZS-4 | *R. nigromaculata* | 110.4 E | 18.8 N | 2013 | KM605373 | KM605285 |
| HN-WZS-5 | *R. nigromaculata* | 110.4 E | 18.8 N | 2013 | KM605374 | KM605286 |
| HN-WZS-6 | *R. nigromaculata* | 110.4 E | 18.8 N | 2013 | KM605375 | KM605287 |
| HN-WZS-7 | *R. nigromaculata* | 110.4 E | 18.8 N | 2013 | KM605376 | KM605288 |
| HN-WZS-8 | *R. nigromaculata* | 110.4 E | 18.8 N | 2013 | KM605377 | KM605289 |
| HN-WZS-9 | *R. nigromaculata* | 110.4 E | 18.8 N | 2013 | KM605378 | KM605290 |
| GZ-GY-1 | *Enhydris chinensis* | 106.63 E | 26.65 N | 2012 | KM605379 | KM605291 |
| GZ-GY-2 | *E. chinensis* | 106.63 E | 26.65 N | 2012 | KM605380 | KM605292 |
| GZ-GY-3 | *R. nigromaculata* | 106.63 E | 26.65 N | 2012 | KM605381 | KM605293 |
| GZ-GY-4 | *R. nigromaculata* | 106.63 E | 26.65 N | 2012 | KM605382 | KM605294 |
| GZ-GY-5 | *R. nigromaculata* | 106.63 E | 26.65 N | 2012 | KM605383 | KM605295 |
| GZ-GY-6 | *R. nigromaculata* | 106.63 E | 26.65 N | 2012 | KM605384 | KM605296 |
| GZ-AS-1 | *R. nigromaculata* | 105.95 E | 26.25 N | 2013 | KM605385 | KM605297 |
| GZ-AS-2 | *R. nigromaculata* | 105.95 E | 26.25 N | 2013 | KM605386 | KM605298 |
| GZ-AS-3 | *R. nigromaculata* | 105.95 E | 26.25 N | 2013 | KM605387 | KM605299 |
| GZ-AS-4 | *R. nigromaculata* | 105.95 E | 26.25 N | 2013 | KM605388 | KM605300 |
| GZ-AS-5 | *R. nigromaculata* | 105.95 E | 26.25 N | 2013 | KM605389 | KM605301 |
| GZ-AS-6 | *R. nigromaculata* | 105.95 E | 26.25 N | 2013 | KM605390 | KM605302 |
| HeN-KF-1 | *R. nigromaculata* | 114.47 E | 34.48 N | 2012 | KM605391 | KM605303 |
| HeN-KF-2 | *R. nigromaculata* | 114.47 E | 34.48 N | 2012 | KM605392 | KM605304 |
| HeN-KF-3 | *R. nigromaculata* | 114.47 E | 34.48 N | 2012 | KM605393 | KM605305 |
| HeN-KF-4 | *R. nigromaculata* | 114.47 E | 34.48 N | 2012 | KM605394 | KM605306 |
| HeN-KF-5 | *R. nigromaculata* | 114.47 E | 34.48 N | 2012 | KM605395 | KM605307 |
| HeN-KF-6 | *R. limnocharis* | 114.47 E | 34.48 N | 2013 | KM605396 | KM605308 |
| HeN-KF-7 | *R. limnocharis* | 114.47 E | 34.48 N | 2013 | KM605397 | KM605309 |
| HeN-KF-8 | *R. limnocharis* | 114.47 E | 34.48 N | 2013 | KM605398 | KM605310 |
| HeN-LH-1 | *R. limnocharis* | 114.02 E | 33.58 N | 2012 | KM605399 | KM605311 |
| HeN-LH-2 | *R. limnocharis* | 114.02 E | 33.58 N | 2012 | KM605400 | KM605312 |
| HeN-LH-3 | *R. limnocharis* | 114.02 E | 33.58 N | 2012 | KM605401 | KM605313 |
| HeN-LH-4 | *R. limnocharis* | 114.02 E | 33.58 N | 2012 | KM605402 | KM605314 |
| HeN-LH-5 | *R. limnocharis* | 114.02 E | 33.58 N | 2012 | KM605403 | KM605315 |
| HeN-LH-6 | *R. nigromaculata* | 114.02 E | 33.58 N | 2013 | KM605404 | KM605316 |
| HeN-LH-7 | *R. nigromaculata* | 114.02 E | 33.58 N | 2013 | KM605405 | KM605317 |
| HeN-LH-8 | *R. nigromaculata* | 114.02 E | 33.58 N | 2013 | KM605406 | KM605318 |
| HeN-XX-1 | *R. limnocharis* | 113.87 E | 35.3 N | 2013 | KM605407 | KM605319 |
| HeN-XX-2 | *R. limnocharis* | 113.87 E | 35.3 N | 2013 | KM605408 | KM605320 |
| HeN-XX-3 | *R. limnocharis* | 113.87 E | 35.3 N | 2013 | KM605409 | KM605321 |
| HeN-XX-4 | *R. limnocharis* | 113.87 E | 35.3 N | 2013 | KM605410 | KM605322 |
| HeN-ZK-1 | *R. nigromaculata* | 114.38 E | 34.07 N | 2013 | KM605411 | KM605323 |
| HeN-ZK-2 | *R. nigromaculata* | 114.38 E | 34.07 N | 2013 | KM605412 | KM605324 |
| HeN-ZK-3 | *R. nigromaculata* | 114.38 E | 34.07 N | 2013 | KM605413 | KM605325 |
| HeN-ZK-4 | *R. nigromaculata* | 114.38 E | 34.07 N | 2013 | KM605414 | KM605326 |
| HeN-ZK-5 | *R. nigromaculata* | 114.38 E | 34.07 N | 2013 | KM605415 | KM605327 |
| HeN-ZZ-1 | *R. nigromaculata* | 113.65 E | 34.73 N | 2012 | KM605416 | KM605328 |
| HeN-ZZ-2 | *R. nigromaculata* | 113.65 E | 34.73 N | 2012 | KM605417 | KM605329 |
| HeN-ZZ-3 | *R. temporaria* | 113.65 E | 34.73 N | 2012 | KM605418 | KM605330 |
| HeN-ZZ-4 | *R. temporaria* | 113.65 E | 34.73 N | 2012 | KM605419 | KM605331 |
| HeN-ZZ-5 | *R. nigromaculata* | 113.65 E | 34.73 N | 2012 | KM605420 | KM605332 |
| HuN-JS-1 | *R. nigromaculata* | 109.73 E | 28.32 N | 2012 | KM605421 | KM605333 |
| HuN-JS-2 | *R. nigromaculata* | 109.73 E | 28.32 N | 2012 | KM605422 | KM605334 |
| HuN-JS-3 | *R. nigromaculata* | 109.73 E | 28.32 N | 2012 | KM605423 | KM605335 |
| HuN-JS-4 | *R. nigromaculata* | 109.73 E | 28.32 N | 2012 | KM605424 | KM605336 |
| HuN-JS-5 | *R. nigromaculata* | 109.73 E | 28.32 N | 2012 | KM605425 | KM605337 |
| HuN-JS-6 | *R. nigromaculata* | 109.73 E | 28.32 N | 2012 | KM605426 | KM605338 |
| HuN-CS-1 | *R. nigromaculata* | 112.93 E | 28.23 N | 2013 | KM605427 | KM605339 |
| HuN-CS-2 | *R. nigromaculata* | 112.93 E | 28.23 N | 2013 | KM605428 | KM605340 |
| HuN-CS-3 | *R. nigromaculata* | 112.93 E | 28.23 N | 2013 | KM605429 | KM605341 |
| HuN-CS-4 | *R. nigromaculata* | 112.93 E | 28.23 N | 2013 | KM605430 | KM605342 |
